# A directing group switch in copper-catalyzed electrophilic C–H amination/migratory annulation cascade: divergent access to benzimidazolone/benzimidazole†

**DOI:** 10.1039/d2sc01420c

**Published:** 2022-04-14

**Authors:** Hasina Mamataj Begam, Shantanu Nandi, Ranjan Jana

**Affiliations:** Organic and Medicinal Chemistry Division, CSIR-Indian Institute of Chemical Biology 4 Raja S. C. Mullick Road, Jadavpur Kolkata-700032 West Bengal India rjana@iicb.res.in

## Abstract

We present here a copper-catalyzed electrophilic *ortho* C–H amination of protected naphthylamines with *N*-(benzoyloxy)amines, cyclization with the pendant amide, and carbon to nitrogen 1,2-directing group migration cascade to access *N*,*N*-disubstituted 2-benzimidazolinones. Remarkably, this highly atom-economic tandem reaction proceeds through a C–H and C–C bond cleavage and three new C–N bond formations in a single operation. Intriguingly, the reaction cascade was altered by the subtle tuning of the directing group from picolinamide to thiopicolinamide furnishing 2-heteroaryl-imidazoles *via* the extrusion of hydrogen sulfide. This strategy provided a series of benzimidazolones and benzimidazoles in moderate to high yields with low catalyst loading (66 substrates with yields up to 99%). From the control experiments, it was observed that after the C–H amination an incipient tetrahedral oxyanion or thiolate intermediate is formed *via* an intramolecular attack of the primary amine to the amide/thioamide carbonyl. It undergoes either a 1,2-pyridyl shift with the retention of the carbonyl moiety or H_2_S elimination for scaffold diversification. Remarkably, inspite of a positive influence of copper in the reaction outcome, from our preliminary investigations, the benzimidazolone product was obtained in good to moderate yields in two steps under metal-free conditions. The *N*-pyridyl moiety of the benzimidazolone was removed for further manipulation of the free NH group.

## Abstract 2

A novel directing group switch strategy is explored in a copper-catalyzed divergent synthesis of benzimidazolone *via* electrophilic C–H amination/cyclization/1,2-C → N directing group migration cascade and benzimidazole through the extrusion of H_2_S.

## Graphical abstract

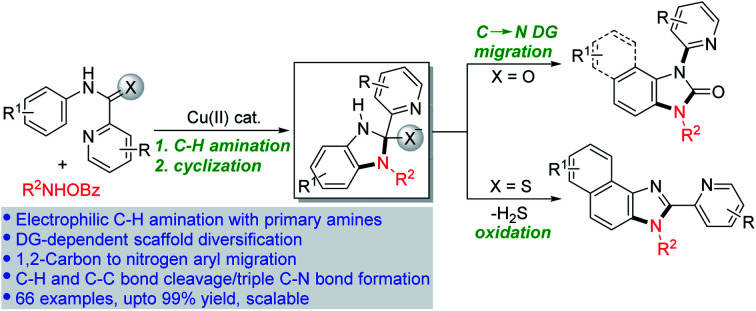

## Introduction

**Fig. 1:**
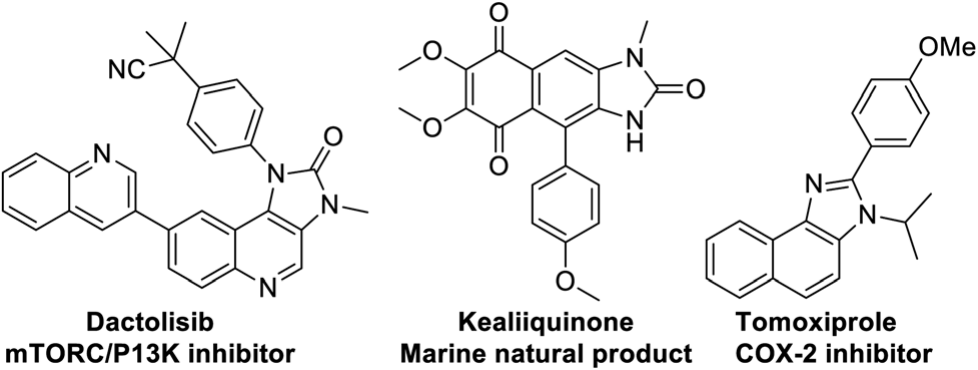
Biologically important imidazolone/imidazole core.

**Scheme 1:**
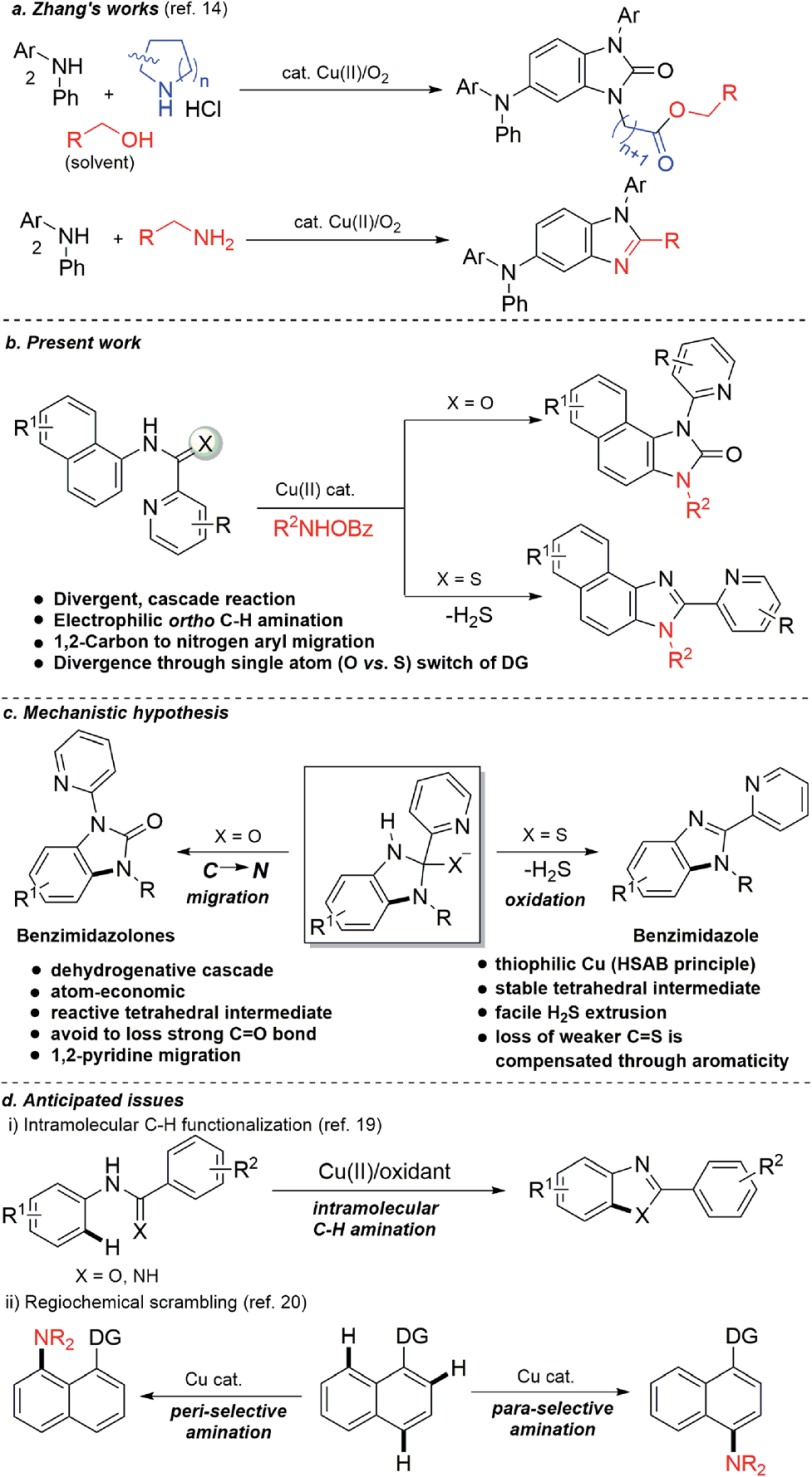
Cascade synthesis of benzimidazole and benzimidazolones.

At the threshold of an era of automated organic synthesis, the cascade reaction encompassing C–H activation is emerging as a powerful tool for the rapid construction and late-stage diversification of functional molecules in a step- and atom-economic manner.^1^ In this cascade process, a command to control chemoselectivity using an inherent chemical reporter enables to achieve rapid molecular diversity.^2^ However, the introduction and removal of directing groups is one of the major drawbacks in chelation-assisted C–H functionalization.^3^ To circumvent, transient, traceless, and inherent directing group strategies were unveiled.^4^ Very recently, the migration and incorporation of a directing group into the target molecule has emerged as a practical strategy in transition metal-catalyzed C–H functionalization to access rapid molecular complexity maximizing atom-economy.^5^ In this direction, cobalt and rhodium-catalyzed stereoselective syntheses of tetra-substituted alkenes were reported through the migration of the directing group.^6^ The Ackermann group reported a cobalt-catalyzed pyridine-directing group migration/annulation cascade in C–H activation.^7^ The Wang group reported manganese-catalyzed C–H activation/enaminylation/DG migration cascade in an *N*-pyrimidyl protected indole system.^8^ The Huang group demonstrated a multitasking directing group strategy for structural diversification in C–H activation cascade.^9^ Very recently, the Li group reported a Pd-catalyzed domino C4-arylation/3,2-carbonyl directing group migration in indoles.^10^ To the best of our knowledge, there is no report of a single atom of a directing group (O *vs.* S) switch for scaffold diversification exploiting their innate reactivity in the C–H functionalization/annulation cascade. Benzimidazolone and benzimidazole are prevalent in pharmaceuticals, agrochemicals, pigments, herbicides, and fine chemicals (Fig. 1).^11^ The synthesis of these privileged scaffolds primarily relies on *ortho*-phenylenediamine or *ortho*-haloanilines.^12^ Recently, the oxidative annulation of anilides/aniline is emerging as an attractive strategy for expanding the substrate scope.^13^ The Zhang group reported the copper-catalyzed synthesis of benzimidazolones and benzimidazoles exploiting the SOMO-stabilized radical cation of diarylamines (Scheme 1a).^14^ The electrophilic C–H amination has emerged as a benign alternative to construct *N*-heterocycles under mild conditions.^15^ Although a Ru(ii)-catalyzed O → N migration during benzimidazole to benzimidazolone conversion is reported,^16^ direct C-2 aryl migration is not reported. We disclose here a novel single atom of a directing group switch strategy for the chemoselective synthesis of 1,3-dihydro-2*H*-naphtho[1,2-*d*]imidazole-2-one *via* electrophilic *ortho* C–H amination, intramolecular cyclization, C → N 1,2-pyridyl shift or 2-(pyridin-2-yl)-3*H*-naphtho[1,2-*d*]imidazole derivatives through an electrophilic C–H amination/annulation cascade (Scheme 1b).

Contrary to the previous procedures, we observed an excellent reactivity of the bicyclic system towards electrophilic *ortho* C–H amination with secondary amines under our optimized reaction condition.^17^ We hypothesized that C–H amination with primary amine may initiate an annulation cascade *via* subsequent addition to the picolinamide directing group. The incipient oxyanion may trigger the 1,2-migration of the directing group to provide benzimidazolone. Instead, thiophilic copper may endorse the extrusion of hydrogen sulfide to provide benzimidazole from the corresponding thioamide directing group (Scheme 1c). However, (1) copper-catalyzed C–H amination with a primary amine is a formidable challenge due to the catalyst inhibition;^18^ (2) a deleterious intramolecular oxidative cyclization of the corresponding directing group may impede the cascade process^19^ (Scheme 1d(i)); (3) competing *peri*- and *para*-C–H amination of naphthalene should be suppressed^20^ (Scheme 1d(ii)). Furthermore, C–H amination with aliphatic primary amines is challenging and less explored.^21^

## Results and discussion

We hypothesized that C–H amination with electrophilic primary amine surrogates may overcome the catalyst inhibition problem. To test our hypothesis, picolinamide-protected 1-naphthylamine 1a, and 1.0 equiv. of *O*-benzoylhydroxylamine 2a was subjected to our previous reaction condition to afford the expected imidazolone product 3a in 44% yield. In the course of further optimization, DMSO was found to be the optimal solvent and excellent yields of this tandem reaction, *e.g.*, 93% and 87% were obtained with just 5.0 and 1.0 mol% catalyst loading, respectively (Table 1). Other copper catalysts, lower reaction temperature, and 1.2 equiv. of 2a provided inferior results. Free primary amines along with external oxidants such as PIDA, K_2_S_2_O_8_ or Bz_2_O_2_ were able to furnish the desired product in <5%, 12% and 32% yields, respectively. Using 1.2 equiv. of 2a along with 1.0 equiv. Bz_2_O_2_ the yield was dropped to 58%. Other metal catalysts such as Pd(OAc)_2_, Co(OAc)_2_, Ni(OAc)_2_, Mn(OAc)_2_, FeCl_2_, and FeBr_2_ were less or ineffective for this transformation (for detailed optimization, see the ESI†). Other aminating reagents such as anthranil, benzisoxazole, or oxime esters under the same condition could not afford any desired product.

**Table 1:** Optimization of the reaction conditions^a^^,^^b^

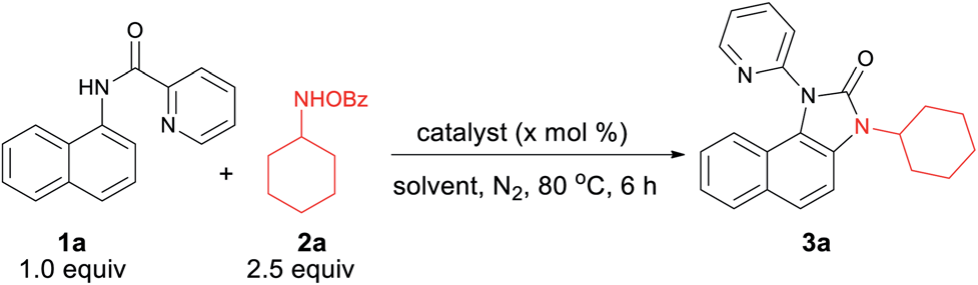					
Entry	Catalyst	X	Oxidant	Solvent	Yield^b^ (3a)
1^c^	Cu(OAc)_2_·H_2_O	10	—	DMSO	44
2	Cu(OAc)_2_·H_2_O	10	—	H_2_O	0
3	Cu(OAc)_2_·H_2_O	10	—	MeCN	39
4	Cu(OAc)_2_·H_2_O	10	—	Dioxane	62
5	Cu(OAc)_2_·H_2_O	10	—	DMSO	90
6	Cu(OAc)_2_·H_2_O	5	—	DMSO	93
7	Cu(OAc)_2_·H_2_O	2.5	—	DMSO	90
8	Cu(OAc)_2_·H_2_O	1.0	—	DMSO	87
9	Cu(OAc)_2_·H_2_O	0.5	—	DMSO	72
10^d^	Cu(OAc)_2_·H_2_O	0	—	DMSO	0
11^e^	Cu(OAc)_2_·H_2_O	2.5	—	DMSO	56
12	Cu powder	5	—	DMSO	83
13^f^	Cu(OAc)_2_·H_2_O	10	PIDA	DMSO	>5
14^g^	Cu(OAc)_2_·H_2_O	10	Bz_2_O_2_	DMSO	58
15	Pd(OAc)_2_	10	—	DMSO	>5
16	Co(OAc)_2_	10	—	DMSO	20

a All reactions were carried out on a 0.2 mmol scale.

b Yields refer to here are overall isolated yields.

c 1.0 equiv. 2a was used.

d 25% *ortho* aminated product was obtained.

e Reaction was performed at room temperature.

f Free amine was used as amine source and 2.5 equiv. PIDA as oxidant.

g 1.0 equiv. Bz_2_O_2_ and 1.2 equiv. of 2a were used.

Under the optimized reaction condition, cyclic as well as acyclic primary amines furnished the desired products in good to high yields (Table 2). Cyclohexylamine provided an excellent 93% yield of the desired product (3a), whereas cyclopentyl, cycloheptyl, and cyclooctylamines afforded slightly lower 78%, 65%, and 46% yields, respectively (3b–3d). In the case of acyclic amines, yields increased with the increase in chain length (3e–3g) and steric bulk. Surprisingly, sterically hindered *sec*-butyl (3i), *tert*-butyl (3j), adamantylamine (3m) provided almost quantitative yields exhibiting a positive influence of steric bulk of amines on the rearrangement cascade. Competition experiment of 1a with 2m and 2n afforded 3m and 3n in 70% and 28%, respectively. Several substituted 1-naphthylamines including methyl substitution at the C8 position provided high to excellent yields of the desired products (3q–3y). Heterocyclic amino quinoline and isoquinoline provided the rearranged product in excellent yields (3z, 3aa). Delightedly, diverse (2-*N*-heteroaryl) anilides serve as excellent directing groups and are incorporated into the benzimidazolones (3ac–3ah). Interestingly, 3-methyl picolinamide provided the corresponding *N*-pyridyl product (3ac) *via ipso*-substitution. Similarly, the amides of 5-methyl pyrazinoic (3ad, CCDC 2025269), quinaldic (3ae), and pyrazinoic acid (3af, 3ag) also furnished in comparable yields. Isoquinoline-1-carbamide provided a mixture of benzimidazolone 3ah and C–H amination product 3ah′. This may be the result of an unfavourable tetrahedral intermediate due to steric reasons. 6-Aminoquinoline provided the C–H amination at the C5 position. Furthermore, sterically less hindered methyl amine provided a corresponding 2-pyridylbenzimidazole (3aj, CCDC 2025266), imparting the role of steric and trajectory of the C–H amination on the subsequent rearrangement reaction.

**Table 2:** Substrate scope of benzimidazolones^a^^,^^b^^,^^g^

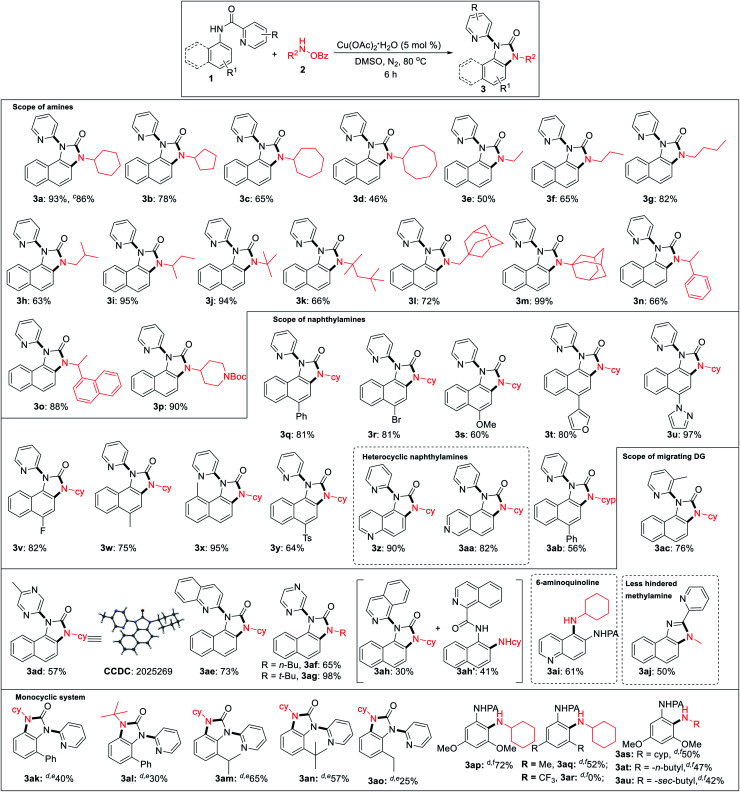

a All reactions were carried out in 0.2 mmol scale.

b Yields refer to the overall isolated yields with respect to 1.

c 2.0 mmol scale reaction.

d 10 mol% Cu(OAc)_2_·H_2_O is used.

e Reaction temperature was 100 °C.

f Additional 2.0 equiv. LiO^*t*^Bu is used and reaction temperature was 90 °C. In the competition experiment: 1a (0.20 mmol), 2m (0.50 mmol), 2n (0.50 mmol), Cu(OAc)_2_·H_2_O (0.01 mmol), dry DMSO (2.0 mL), N_2_, 80 °C, and 6 h.

g Reaction conditions: 1 (0.20 mmol), 2 (0.50 mmol), Cu(OAc)_2_·H_2_O (0.01 mmol), Dry DMSO (2.0 mL), N_2_, 80 °C, and 6 h.

We performed the reaction with various *ortho*, *meta*, *para*-substituted anilines under the same condition and successfully we got 30% rearranged product in 2-phenyl aniline and *ortho* amination product (35%) in a 3,5-dimethoxy aniline system. After getting these results, we turned our attention to optimize these reactions (for detailed optimization; see the ESI†). After several screenings of solvents, base or acid additives, ligands there was no significant improvement in the rearrangement reaction of 2-phenyl aniline picolinamide. Increasing temperature to 100 °C product yield increased to 40%. Then, we performed the reaction in different *ortho*-substituted anilines (electron-donating bulky groups) to obtain rearranged benzimidazolones in moderate to good yields (3ak–3ao). Finally, we were able to get an *ortho* C–H amination product in 3,5-dimethoxy aniline with 72% yield at 90 °C using 2.0 equiv. LiO^*t*^Bu (3ap). While 3,5-dimethyl substituted picolinamide furnished the C–H amination product in 52% yield, the corresponding 3,5-bis(trifluoromethyl)aniline substrate remained unreactive indicating a profound role of electronic nature (3aq, 3ar). Besides cyclohexylamine, other amines also afforded the *ortho* C–H amination products in moderate yields (3as–3au). However, no benzimidazolones were formed even at a low catalyst loading or additives, suggesting that an *ortho* substitution w.r.t. picolinamide is crucial for the subsequent rearrangement reaction.

2-Pyridylbenzimidazoles are an important class of compounds generally used as excellent ligands for metal complexation and glukokinase activator.^22^ We turned our attention to optimize the reaction for benzimidazole. Initially, we started the optimization of benzimidazole with 1a and 2a. All our efforts using different acidic solvents such as AcOH or TFE, acid additives such as BzOH, TsOH, and high temperature (120 °C) to facilitate dehydration after cyclization were in vain. Gratifyingly, the corresponding picolinimidamide afforded the imidazole product in 30% yield in 12 h. Since thioamides have been proved as excellent directing groups in Pd- or Co-catalyzed C–H functionalizations,^23^ we hypothesized that thiophilic copper^24^ may trigger the liberation of hydrogen sulfide from thioamide instead of 1,2-pyridyl migration to furnish the desired benzimidazole product. Hence, examining thiopicolinamide 4a under the reaction condition resulted in the desired 2-pyridylbenzimidazole exclusively albeit in a low yield. After several screenings (for detailed optimization, see the ESI†), using 3.0 equiv. of 2a, 20 mol% Cu(OAc)_2_·H_2_O and 1.2 equiv. of K_2_CO_3_ in DMSO at 90 °C under O_2_ atmosphere the desired product was obtained in 88% yield. During the optimization, we found K_2_CO_3_ and O_2_ to be crucial otherwise yield dropped significantly.

During the substrate scope studies, we observed a positive influence of the increasing chain length of the linear amines (5c–5d, Table 3). Unlike imidazolones, the yields of isomeric acyclic amines decreased with the increase in α-substitution (5d–5g). Benzylamine, furylamine, adamantanemethylamine and 4-amino-1-boc piperidine afforded the products in moderate yields (5h–5k). Thioamides of 5-methyl pyrazine-2-carboxylic acid and 2-quinaldic acids also provided the imidazole product in moderate yields (5l, 5m). Various substituted 1-naphthylamines at different positions also underwent the reaction providing the desired product in medium to good yields (5n–5s). The reaction is also reproducible on a 2.0 mmol scale demonstrating the practicability of this methodology. The thio-picolinamide of monocyclic anilines under the same condition mostly oxidized to picolinamide.

**Table 3:** Substrate scope of benzimidazoles^a^^,^^b^^,^^e^

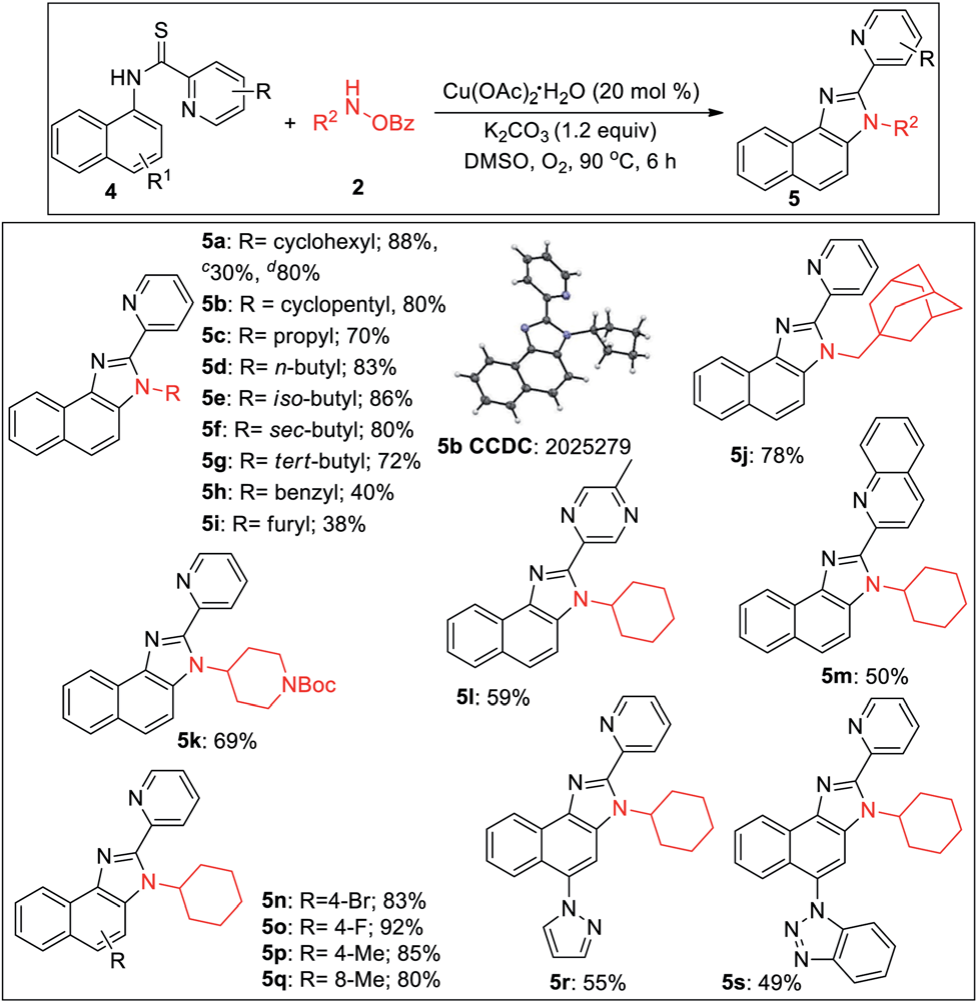

a All reactions were carried out in 0.2 mmol scale.

b Yields refer to overall isolated yields with respect to 4.

c Picolinimidamide 4a′ used as substrate.

d Reaction in 2.0 mmol scale.

e Reaction conditions: 4 (0.20 mmol), 2 (0.60 mmol), Cu(OAc)_2_·H_2_O (0.04 mmol), K_2_CO_3_ (0.24 mmol), dry DMSO (2.0 mL), O_2_, 90 °C, and 6 h.

Intrigued by the initial C–H amination product formation under the metal-free conditions (entry 10, Table 1), we wondered whether it can be re-optimized to achieve the desired benzimidazolones. Initially, a mixture of C–H amination product 1a′, imidazolone 3a and unreacted starting material was obtained, which was subjected to the subsequent cyclization reaction (Table 4). However, the work-up of the reaction mixture and the subsequent addition of benzoyl peroxide converted the C–H amination product to the desired annulation product. Increasing the amine source 2a to 4.0 equiv. or temperature to 90 °C did not improve the yield. Gratifyingly, varying different substituents in the phenyl ring of 2, we observed that 4-NMe_2_ substitution furnished the product in 65% yield. Protected cyclopentylamine afforded the desired product in 44% yield in 12 h. Monocyclic substrates were unreactive under metal-free conditions. Notably, the possibility of metal contamination was excluded by using all new glassware, freshly distilled solvents, and repeating the reaction at least four times. Although, this metal-free, two-step process has inherent limitations, we anticipate that it has profound mechanistic implications.

**Table 4:** Optimization of the metal-free condition^a^^,^^b^^,^^e^

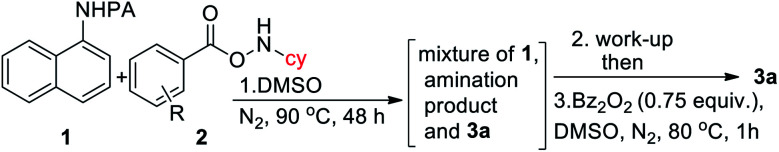			
Entry	Amine source	Equiv. of 2	Yield^*b*^ (3)
1	2a: R = H	2.5	25
2	2a: R = H	4.0	25
**4**	2a_2_**: R = 4-NMe**_**2**_	**4.0**	**65**
5	2a_3_: R = 4-CF_3_	4.0	10
6	2a_4_: R = 4-^*t*^Bu	4.0	20
7	2a_5_: R = 4-OMe	4.0	41
8	2a_6_: R = 3,5-di Cl	4.0	0
9	2a_7_: R = pentafluoro	4.0	25
10^c^	2b_2_**: R = 4-NMe**_**2**_	4.0	**44**
11^d^	2a_2_**: R = 4-NMe**_**2**_	4.0	**70**

a All reactions were carried out on a 0.1 mmol scale.

b Yields refer to here are isolated yields after two steps with respect to 1.

c Protected cyclopentylamine was used and reaction time was 12 h.

d Substrate 1r was used.

e Reaction conditions: 1st step: 1 (0.10 mmol), 2 (0.40 mmol), dry DMSO (1.0 mL), N_2_, 90 °C, and 48 h. 2nd step: Bz_2_O_2_ (0.75 equiv.), DMSO, N_2_, 80 °C, and 1 h.

**Scheme 2:**
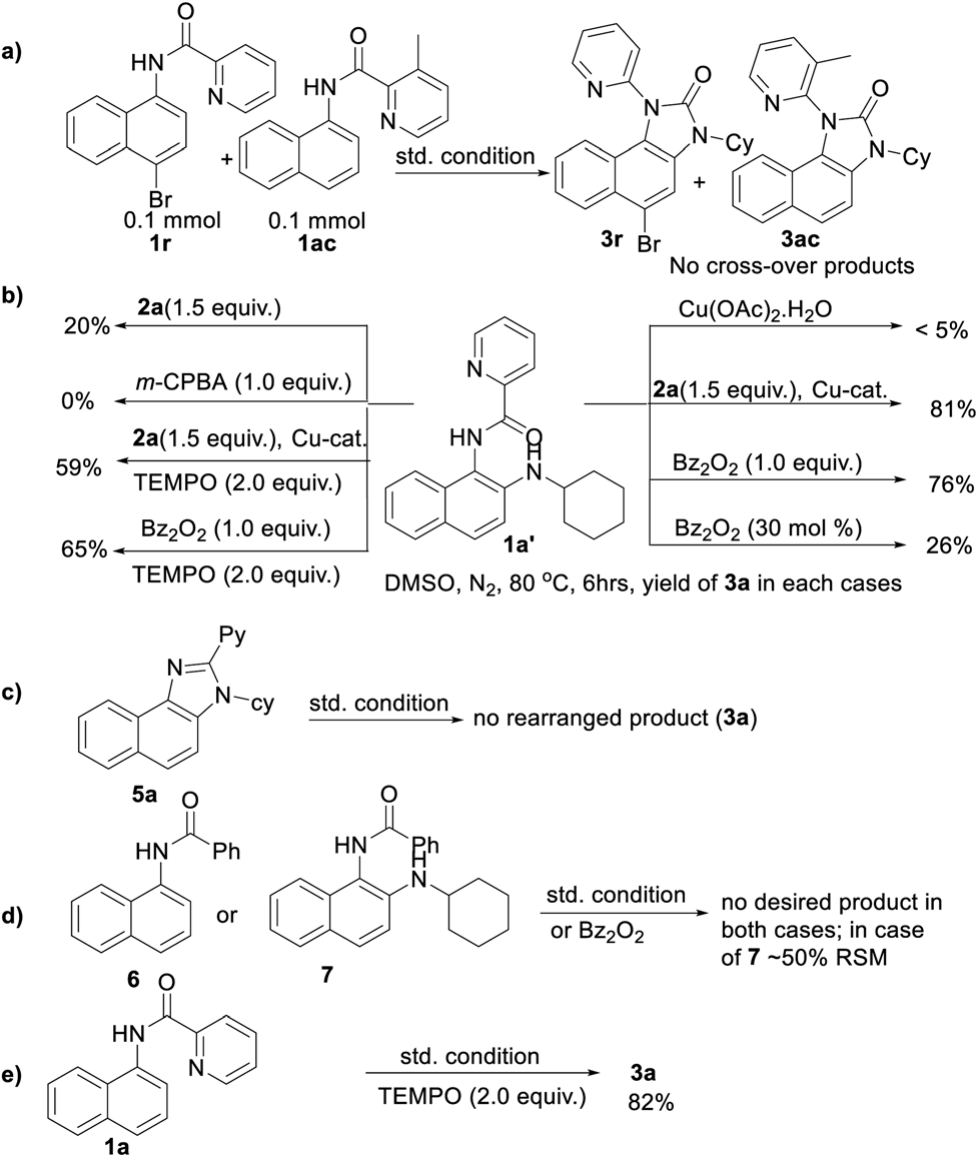
Control experiments.

**Fig. 2:**
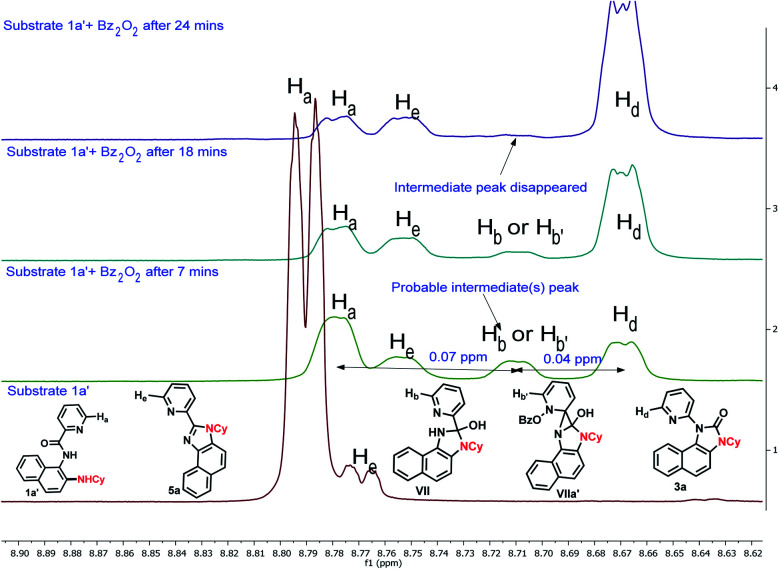
Time-dependent ^1^H NMR experiment.

To gain insight into the reaction mechanism, when an equimolar mixture of 1r and 1ac was treated in our reaction condition, 3r and 3ac were formed and no cross-over product was isolated suggesting an intramolecular 1,2-pyridyl migration (Scheme 2a). Unsymmetrically-substituted 5-methyl pyrazine anilide 1ad afforded the imidazolone product 3ad exclusively through *ipso* C–N bond formation (Table 2). Either performing the reaction at room temperature for a shorter time or quenching the reaction after 1.5 h, a mixture of the starting material, C–H amination product 1a′, and rearranged product were observed. Heating a preformed C–H amination product 1a′ in absence of 2a <5% product was formed. However, a combination of copper catalyst and *O*-benzoylhydroxylamine 2a (1.5 equiv.) or even benzoylperoxide alone furnished the desired 3a in high yields (Scheme 2b). Therefore, besides an aminating agent, 2a also acts as an oxidant for catalytic turnover. Furthermore, the imidazole product 5a was not converted into the corresponding imidazolone 3a under the reaction condition, suggesting a plausible concerted cyclization/1,2-pyridyl migration pathway (Scheme 2c). In place of picolinamide 1a, the corresponding benzanilide 6 or 7 did not furnish any desired product (Scheme 2d). 7 does not undergo any rearrangement even in the presence of Bz_2_O_2_, suggesting that the pyridyl-nitrogen is not only involved in the directed C–H amination but also in the subsequent steps. In both conditions, 7 underwent decomposition with 50% starting material recovery. To examine the involvement of radicals in this C–H amination/migratory annulation cascade, we performed the reaction in the presence of 2.0 equiv. of TEMPO. The yield was slightly decreased from 93% to 82% (Scheme 2e). Further, cyclization from a preformed amination product 1a′ furnished an almost similar yield in the presence of TEMPO. Hence, the single electron transfer (SET) process may not be involved in the migratory cyclization step. Then, we performed the time-dependent ^1^H NMR experiment to get a clue about the intermediate of the rearrangement reaction. We took 1a′ in a NMR tube in DMSO-d_6_ and 1.0 equiv. of Bz_2_O_2_ was added to it for recording the time-dependent ^1^H NMR spectra at room temperature (Fig. 2). After 5–7 min, a new peak corresponding to H_b_ or H_b′_ appeared at 8.71, which was slowly decreased and finally diminished while the desired product was formed. In this study, the chemical shift of the *ortho* proton of pyridine (H_a_) in 1a′ is indicative of the formation of intermediate VII or VIIa′, which gradually shifted to more upfield in the intermediates and then in the product (H_d_). It indicates that the electron-withdrawing effect of the amide-carbonyl is no longer persists in the intermediate pointing either of the intermediates VII or VIIa′ (H_b_ or H_b′_). Since the chemical shift value in the intermediate is closer to the product than the starting material, we assume that the new C–N bond may already have been formed, which is in favor of the intermediate VIIa′ (for full spectrum, see the ESI†).

**Scheme 3:**
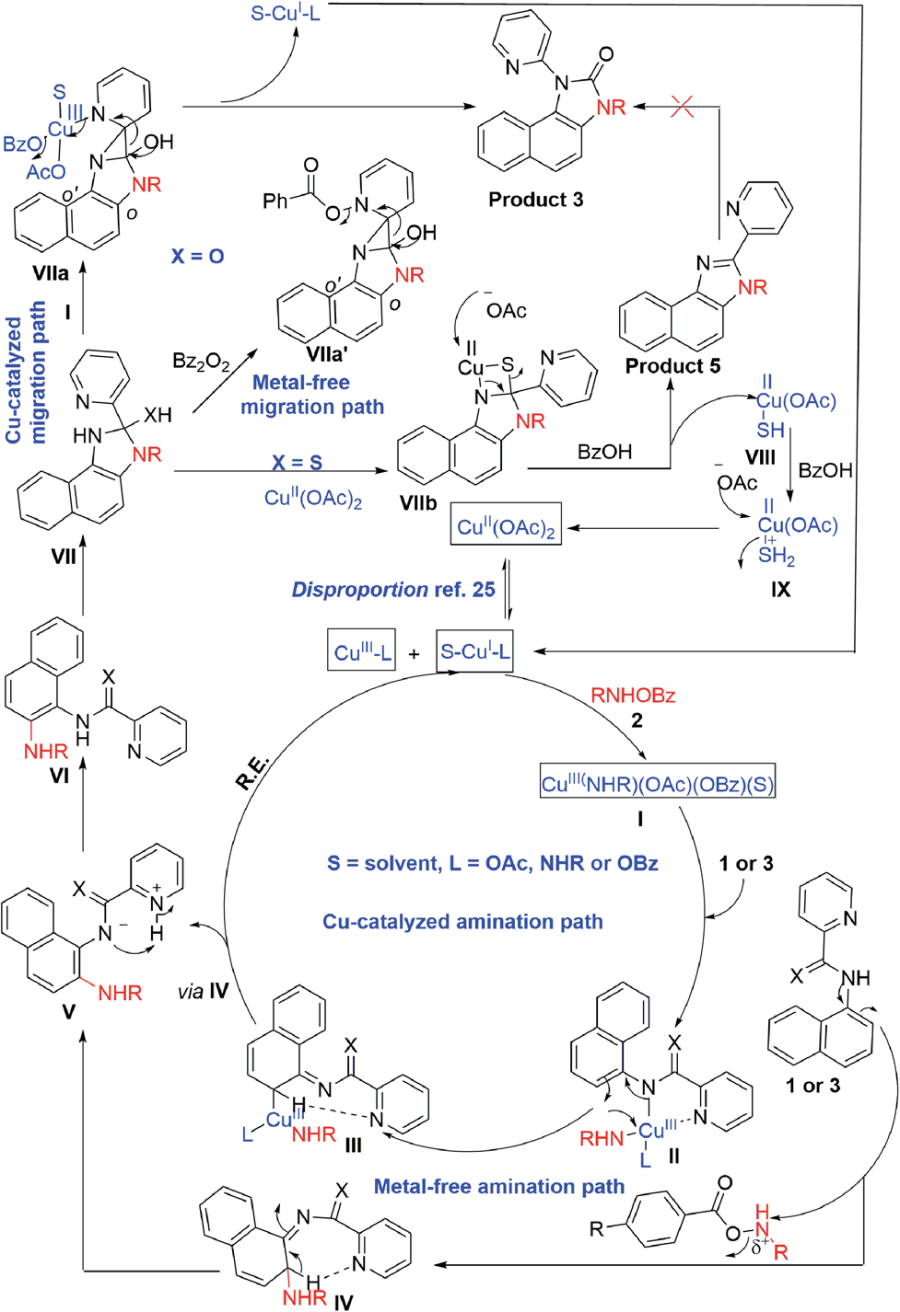
Proposed reaction mechanism.

A Cu^I^/Cu^III^ cycle may be involved in this cascade reaction as reported by the Li, Gaunt, Stahl, and other groups.^25^ Based on control experiments and the above literature reports, Cu(ii) salt first undergoes disproportionation to form an active Cu^I^ species, which undergoes oxidative addition to *O*-benzoylhydroxylamine 2 forming a Cu^III^ complex, I (Scheme 3). This complex chelates with the substrate 1 or 3 forming II, which then undergoes electrophilic aromatic substitution type reaction generating III. Similarly, intermediate IV may also form under metal-free conditions. The pyridine of the picolinamide may work as a proton shuttle that abstracts *ortho* proton and delivers to the amide nitrogen during rearomatization through V providing *ortho* amination products VI. Next, a tetrahedral intermediate VII may form through the intramolecular attack of the amine moiety to the amide/thioamide carbonyl group. The nucleophilic nitrogen may attack at the *ipso* position of the pyridine moiety in the presence of the second molecule of the Cu^III^-complex I, forming VIIa that explains the need for an excess (2.5 equiv.) amount of *O*-benzoylhydroxylamine.^26^ A similar kind of an intermediate, VIIa′ may generate in the presence of Bz_2_O_2_ in the metal-free pathway. This kind of a three-membered cyclic intermediate is claimed by the Feng group for aryl migration.^27^ A similar O → C pyridyl migration through the de-aromatization of the pyridine ring was reported.^28^ Then, a 1,2-pyridyl migration from C → N may take place to furnish the corresponding benzimidazolone product 3 and regenerates the active Cu^I^ species. VIIa′ also furnishes the product in a similar fashion. It is further supported by the fact that electron-donating substitution (*e.g.* ethyl, isopropyl in case of monocyclic anilines) at the *o*′-position has a positive influence in the reaction outcome. However, the *tert*-Bu group may hinder the reaction slightly due to steric inhibition. Owing to the distinct reactivity of thiol than the corresponding oxygen analogue,^29^ a putative 4-membered cyclic intermediate VIIb may form with thiophilic copper from the tetrahedral thiolate intermediate (Scheme 3). Subsequently, the elimination of hydrogen sulphide provides the thermodynamically favourable benzimidazoles (5) regenerating Cu(ii). However, the exact mechanism is not clear at this moment and warrants further studies.

**Scheme 4:**
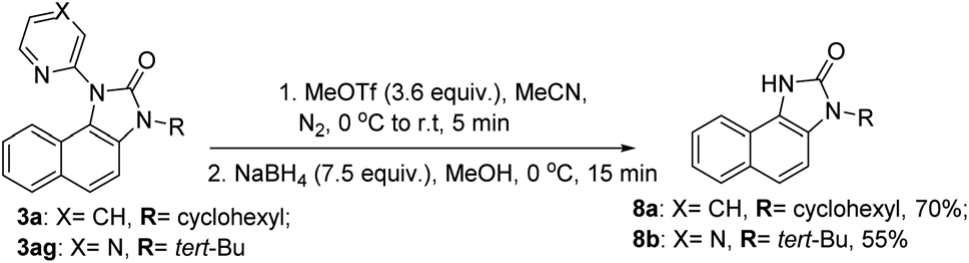
Deprotection of the *N*-heteroaryls of the benzimidazolone product.

Our efforts to utilize the pendant pyridine moiety for further C–H functionalization (thioarylation, alkynylation) at the *peri*-position remained unsuccessful at this moment. However, to expand the synthetic utility of this migratory annulation cascade, the pyridine and pyrazine moieties were deprotected from the representative benzimidazolones (3a, 3ag) using a reported condition providing 8a and 8b in 70% and 55% yield, respectively (Scheme 4).^30^ The free –NH moiety can be further manipulated to achieve further molecular diversity.

## Conclusions

In conclusion, we have demonstrated a directing group-dependent complete switch of product selectivity in copper-catalyzed electrophilic C–H amination with protected primary amines. The benzimidazolones are formed *via* an unprecedented electrophilic *ortho* C–H amination with primary amines, intramolecular cyclization, and 1,2-directing group migration from carbon to the nitrogen center. Remarkably, one C–H and C–C bond cleavage and three new C–N bond formations take place in a single operation in this migratory annulation cascade. Strikingly, by changing the picolinamide directing group to the corresponding thiopicolinamide, the chemoselectivity is switched to form 2-pyridylbenzimidazoles *via* the extrusion of H_2_S. Inexpensive copper catalyst, scale-up synthesis, low catalyst loading, and controlled scaffold diversification are some of the practical aspects of this tandem reaction. From the preliminary studies, the benzimidazolone product was also obtained in good to moderate yields in two steps under metal-free conditions. The in-depth mechanistic studies and investigation of metal-free conditions of this divergent tandem reaction is undergoing in our laboratory.

## Data availability

All experimental data is available in the ESI.† No computation component is present in this manuscript.

## Author contributions

R. J. conceptualized and supervised the project and wrote the manuscript. H. M. B. performed the experiments and wrote the manuscript. S. N. analyzed the crystal structure.

## Conflicts of interest

There are no conflicts to declare.

## Supplementary Material

SC-013-D2SC01420C-s001

SC-013-D2SC01420C-s002
