# Supplementary material for: A directing group switch in copper-catalyzed electrophilic C–H amination/migratory annulation cascade: divergent access to benzimidazolone/benzimidazole
Source: Chem Sci. 2022 Apr 14;13(19):5726–33. doi: 10.1039/d2sc01420c (PMC9116329; doi:10.1039/d2sc01420c)
Supplement: SC-013-D2SC01420C-s001 [file SC-013-D2SC01420C-s001.pdf]

Electronic Supplementary Material (ESI) for Chemical Science.
This journal is © The Royal Society of Chemistry 2022

Electronic Supplementary Information

Directing group switch in copper-catalyzed electrophilic C–H amination/migratory annulation cascade: divergent access to benzimidazolone/benzimidazole

Hasina Mamataj Begam,[a] Shantanu Nandi,[a] and Ranjan Jana*[a]

[a]Organic and Medicinal Chemistry Division, CSIR-Indian Institute of Chemical Biology

4 Raja S. C. Mullick Road, Jadavpur, Kolkata-700032, West Bengal, India

E-mail: rjana@iicb.res.in

Table of Contents

General information…………………………………………………………....................S2

Preparation of starting materials……………………………………………………..S3-S10

Optimization tables ……………………………………………………………………S10-S14

Representative procedures for the main reactions………………………………..S14-S16

Deprotection of the heteroaryl from benzimidazolone product…….……………S16-S17

^{1}H NMR spectra for mechanism…….………………………………………………S17-S18

Crystal structure…….……………………………………………...………S18-S27

Spectral data……. …….………………………………………………...…….S27-S65

References……………………………………………………………….................S66

^{1}H and ^{13}C NMR spectra………………………………………………...…...S67-S147

General Information: Air-sensitive reagents were handled under a dry nitrogen atmosphere. Unless otherwise stated, all commercial reagents were used without additional purification. Solvents were dried using standard methods and distilled before use. TLC was performed on silica gel plates (Merck silica gel 60, f_{254}), and the spots were visualized with UV light (254 and 365 nm) or by charring the plate dipped in $KMnO_4$ or vanillin charring solution. ^{1}H NMR spectra were recorded at 400 MHz (JEOL-JNM-ECZ400S/L1), ^{13}C NMR spectra were recorded at 100 MHz (JEOL-JNM-ECZ400S/L1) and ^{19}F NMR spectra were recorded at 376 MHz (JEOL-JNM-ECZ400S/L1) frequency in $CDCl_3$ solvent using TMS as the internal standard. Chemical shifts were measured in parts per million (ppm) referenced to 0.0 ppm for tetramethylsilane. The following abbreviations were used to explain multiplicities: s = singlet, d = doublet, t = triplet, q = quartet, m = multiplet, br. = broad, dt = doublet of triplet, td = triplet of doublet, dd = doublet of doublet. Coupling constants, *J* were reported in Hertz unit (Hz). HRMS (m/z) were measured using ESI technique (Q-Tof Micro mass spectrometer). Crystals were grown in dichloromethane and crystal data was recorded in (Bruker Kappa Apex-2, CCD Area Detector) instrument.

Preparation of starting materials:

Preparation of some 1-naphthylamines:

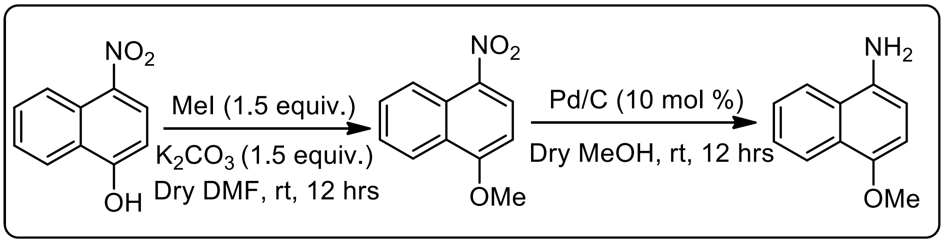

To a solution of 4-nitro-1-naphthol (106.0 mg, 0.4 mmol) in dry DMF (5.0 mL) were added K_2CO_3 (83.0 mg, 0.6 mmol) and MeI (58 µL, 0.6 mmol). The resulting reaction mixture was stirred at r.t. for 12 hrs. After completion of the reaction, the mixture was diluted with ethyl acetate and washed with chilled water, brine solution and dried over Na_2SO_4. The solvent was evaporated under reduced pressure and finally the crude product was purified by column chromatography to afford the desired product.

To a solution of nitro compound (50 mg) in 5.0 mL MeOH was added 10% Pd/C (15 mg) and stirred for 12 hrs under H_2 atmosphere. Then the mixture was diluted with EtOAc (5 mL), filtered through celite, the filtrate was concentrated under reduced pressure to afford the crude compound which was purified by column chromatography to give the corresponding as purple colored viscous liquid which was used for amidation following **procedure1**.

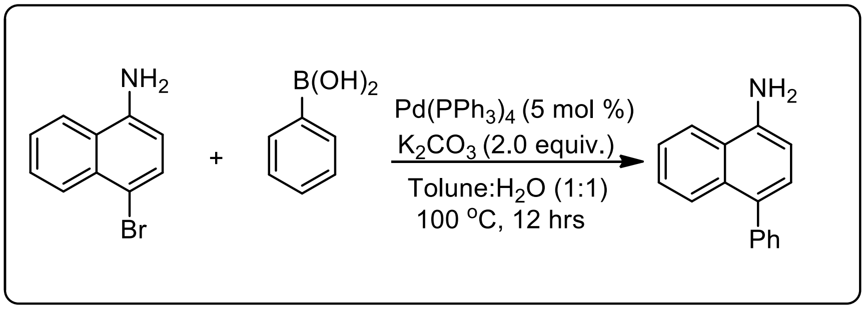

Phenylboronic acid or 3-Furanylboronic acid (1.5 mmol, 1.5 equiv.), 4-bromo-1-naphthylamine (1.0 equiv.), K_2CO_3 (1.0 equiv.) and $Pd(PPh_3)_4$ (5 mol %) were added to an oven-dried sealed tube (15 mL) and toluene (2 mL) and H_2O (2 mL) were added into the tube which was then purged with nitrogen three times. The resulted solution was stirred at 100 °C for 8 hrs. The reaction mixture was cooled to r.t. and then extracted with ethyl acetate (3 × 15 mL). The combined organic layer was washed with brine, dried over anhydrous Na_2SO_4 and concentrated under reduced pressure. This crude product was passed through short column.

These substituted 1-naphthylamine compounds were then used for amidation following **procedure1**.

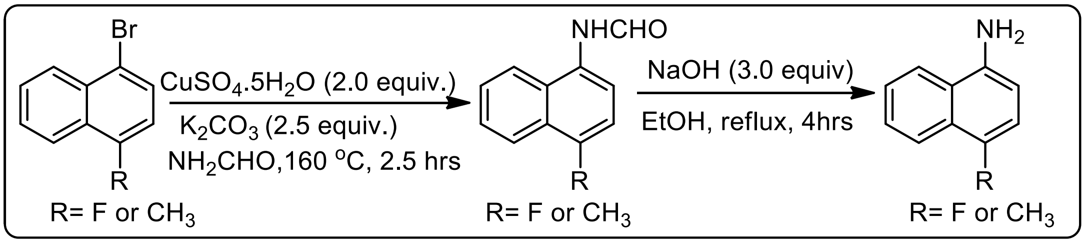

In an oven dried RB substituted 1-bromonaphthalene (2.5 mmol), K_2CO_3 (865 mg, 6.25 mmol), and copper(II) sulfate pentahydrate (750 mg, 5.0 mmol) was taken and to it formamide (10 mL)was added. The RB was fitted with reflux condenser and heated at 160 °C for 4 hrs. (**caution:** don't use pressure tube for this reaction). After cooling to room temperature the reaction mixture was mixed with ice cold water (50 mL) and extracted with ethyl acetate (3×20 mL). Combined organic layers were dried over Na_2SO_4 and evaporated. The crude product was used for the next step without further purification.

Aqueous (2 mL) sodium hydroxide (5.0 equiv.) was added to the ethanol (10 mL) solution of *N*-(naphthalen-1-yl)formamide. Resulting solution was heated to reflux for 6 hrs. Ethanol was evaporated and extracted with ethyl acetate (3×10 mL). The combined organic layer was dried over Na_2SO_4 and solvent was evaporated under reduced pressure. The crude mixture was purified by column chromatography to obtain naphthylamines. These substituted 1-naphthylamine compounds were then used for amidation following **procedure 1**.

General procedure for the preparation of picolinamides:

Procedure 1 for the preparation of picolinamides:

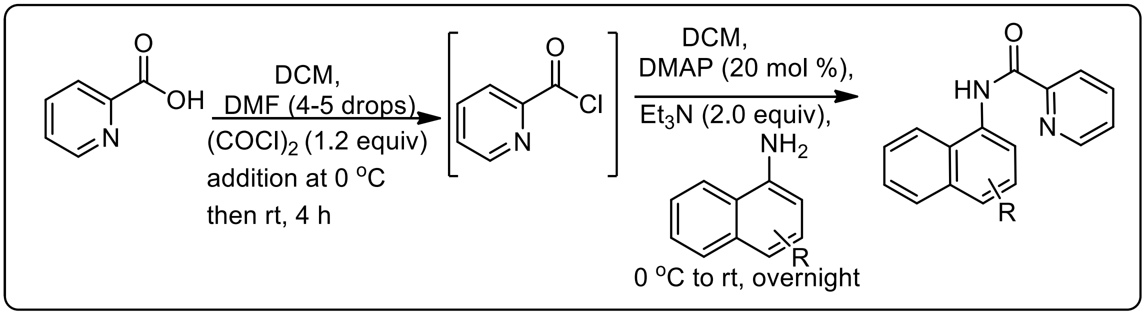

To an oven dried RB was charged with 2-picolinic acid or the corresponding acid (2.5 mmol) in dry DCM (5mL) was added 4-5 drops of DMF and the solution was stirred at 0 °C for 5 minutes. Then oxalyl chloride (0.26ml, 3 mmol) was added dropwise into the cooled solution of the acid which immediately formed rust-red color in case of 2-picolinic acid (other colors for other acids) with the gas bubbling and then stirred at 0 °C for 10 minutes. The reaction mixture was stirred at

room temperature for 4 hours. After completion, the excess oxalyl chloride was removed under *vacuum* to obtain crude acid chloride. Then, the crude pyridine-2-acid chloride was dissolved in 5mL dry DCM, and cooled to 0 °C and DMAP (61 mg, 0.5 mmol), Et_3N (0.7 mL, 5 mmol) and the naphthylamine (2.5 mmol) were successively added. Then the reaction mixture was stirred under room temperature for overnight. After completion as indicated by TLC, the mixture was diluted with 60 mL DCM and washed with 2N HCl (20 mL), brine solution (20 mL) and dried over Na_2SO_4. The solvent was evaporated under reduced pressure and finally the crude product was purified by column chromatography to afford the 2-picolinamide (50-85% yield). This method was used for the synthesis of most of the substrates.

Procedure 2 for the preparation of picolinamides:

A RB containing pyridine-2-carboxylic acid (203 mg, 1.65 mmol), HATU (627 mg, 1.65 mmol) in dry DMF was cooled to 0 °C. Then DIPEA (0.43 ml, 2.5 mmol) and 5-amino isoquinoline (216 mg, 1.5 mmol) were successively added. The reaction mixture was stirred at r.t for overnight. Then the mixture was diluted with ethyl acetate and washed with chilled water, brine solution and dried over Na_2SO_4. The solvent was evaporated under reduced pressure and finally the crude product was purified by column chromatography to afford **1aa**.

Procedure 3 for the preparation of picolinamides:

An oven dried RB charged with 5-methyl pyrazine-2- carboxylic acid (319 mg, 2.31 mmol), 1-naphthylamine (300 mg, 2.1 mmol), EDCI (443 mg, 2.31 mmol), HOBT (312 mg, 2.31 mmol)) in dry DCM was cooled to 0 °C and then DIPEA (1.1 ml, 6.3 mmol) was added. The reaction mixture was stirred at r.t for 12 hrs. Then the mixture was diluted with DCM and washed with water, brine solution and dried over Na_2SO_4. The solvent was evaporated under reduced pressure and finally the crude product was purified by column chromatography to afford **1ad**. This method is also used for the synthesis of **1s**.

Procedure for the synthesis of 1u:

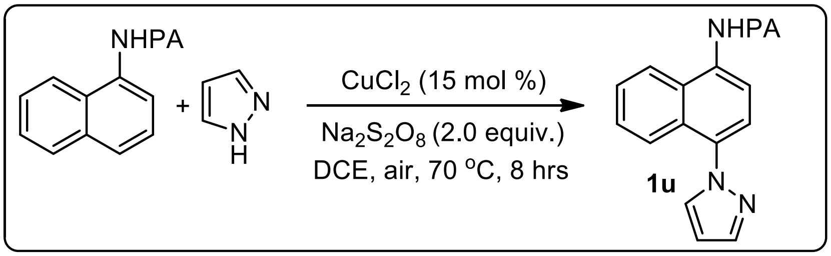

In an oven dried sealed tube charged with **1a** (496 mg, 2.0 mmol), pyrazole (544 mg, 4.0 equiv.), $CuCl_2$ (41 mg, 0.3 mmol), $Na_2S_2O_8$ (952 mg, 4.0 mmol) was added dry DCE (5ml) and heated at 70 °C for 8 hrs. Then the reaction mixture was cooled to r.t and diluted with DCM and washed with water, brine solution

and dried over Na_2SO_4. The solvent was evaporated under reduced pressure and finally the crude product was purified by column chromatography to afford **1u**. This method is also used for the synthesis of amide version of **4r**.

Procedure for the synthesis of 1y:

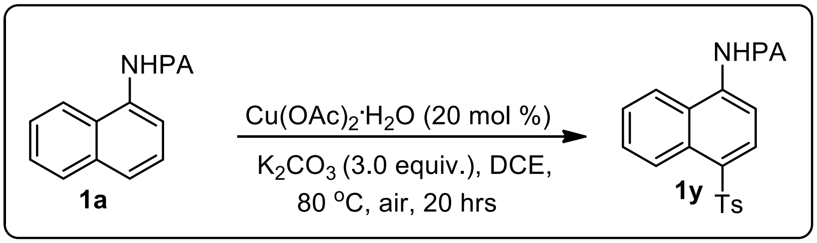

1a (248 mg, 1 mmol, 1.0 equiv.), K_2CO_3 (276.5 mg, 2.0 mmol), $Cu(OAc)_2 \cdot H_2O$ (40.0 mg, 0.2 mmol), TsCl (570 mg, 3.0 mmol) and DCE (5 mL) were successively added into a sealed tube. The mixture was stirred at 80 °C under air for 20 h. After cooling to ambient temperature, the resulting mixture was filtered through celite pad and washed with DCM. The filtrate was concentrated under vacuum and purified column chromatography using solvent mixtures of petroleum ether and ethyl acetate to give the product **1y.**

Procedure for the synthesis of 1x:

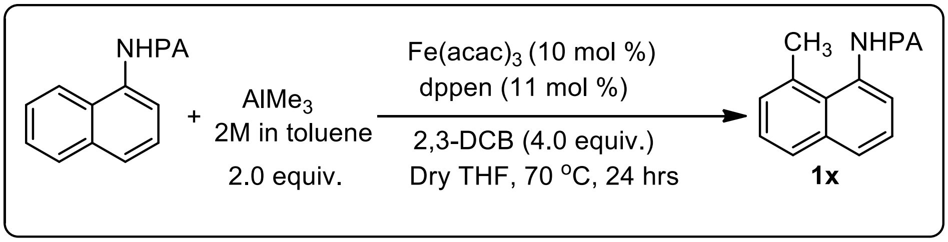

Under a nitrogen atmosphere, in an oven-dried two-necked RB a solution of $Fe(acac)_3$ (71 mg, 0.2 mmol) and (Z)-1,2-bis (diphenylphosphino)ethene (dppen) (88 mg, 0.22 mmol) in THF (1.0 ml) was injected into an anhydrous tetrahydrofuran (THF, 7 mL) solution of **1a** (496 mg, 2.0 mmol). Trimethylaluminum (2 ml, 4.0 mmol, 2.0 M in toluene) was slowly added. After stirring for 10 min to finish generating methane at r.t, 2,3-dichlorobutane (2,3-DCB) (0.9 ml, 8.0 mmol) was added via a syringe, and the RB was joined to reflux condenser and heated at 70 °C for 24 h. After cooling to r.t., the mixture was diluted with diethyl ether, and methanol (2.0 ml) was slowly added via a syringe at 0 °C to quench the aluminium reagent. Then the mixture was further diluted with diethyl ether and washed with water, brine solution and dried over Na_2SO_4. The solvent was evaporated under reduced pressure and finally the crude product was purified by column chromatography to afford **1x.**

General Procedure for the preparation of *O*-benzoylhydroxylamines:

.

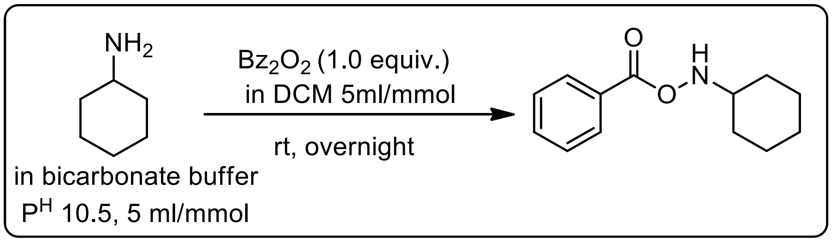

As reported in the literature [1-3] a solution of benzoyl peroxide (BPO, 1 equiv.) in CH_2Cl_2 (5 mL/mmol BPO) was added to a mixture of the amine (1 equiv.) and aqueous Na_2CO_3/$NaHCO_3$ buffer (pH 10.5) solution (5 mL/mmol amine) at room temperature overnight. (Note: The desired product in most cases had R_f values close to that of BPO). The aqueous layer was extracted three times with CH_2Cl_2. The organic layers were combined, dried over anhydrous Na_2SO_4, and concentrated. The crude residue was subjected to column chromatography for purification of the products. Using this protocol, *O*-benzoylhydroxylamine of other aliphatic primary amines were prepared. Most of the compounds are known in literature though ^{1}HNMR and ^{13}NMR spectral data of some compounds are given.

Preparation of P^H 10.5 bicarbonate buffer solution:

100 mL 0.1M $NaHCO_3$ solution = 840 mg $NaHCO_3$ in 100 mL water (solution A), 100 mL 0.1M Na_2CO_3 solution = 1.06 gm Na_2CO_3 in 100 mL water (solution B). For preparation 50 ml of P^H 10.5 = 5 mL solution A + 45 mL solution B.

$2a_4$, $2a_5$, $2a_6$, $2a_7$ were synthesized using corresponding substituted benzoyl peroxides.

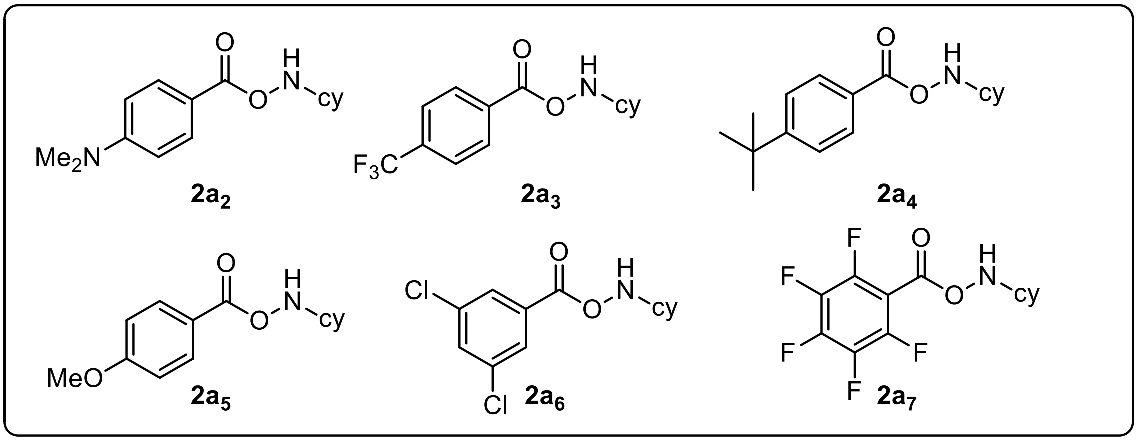

Synthesis of aryl acylperoxide

In a round-bottomed flask, the solution of acid chloride (5 mmol) in diethyl ether (2.5 mL) was cooled to 0 °C in an ice-bath. Then, hydrogen peroxide (0.294 g, 30 wt.% in H_2O, 2.86 mmol) was added dropwise over 10 minutes to the cold solution. This was followed by the dropwise addition of an aqueous solution of NaOH (0.252g, 6.32 mmol, 2 mL) over 20 minutes. The resulting white

precipitate was collected by filtration. After washing with water (3×5 mL) and diethyl ether (3×5 mL), the solid was air dried and used for the next step without any further purification. In some case if the compound was found to dissolve in ether, after evaporating ether the compound was extracted with DCM and dried over anhydrous Na_2SO_4 and after rotary evaporation of the solvent (<40 ºC) the solid compound was obtained.

Synthesis of 2a2 and 2a3

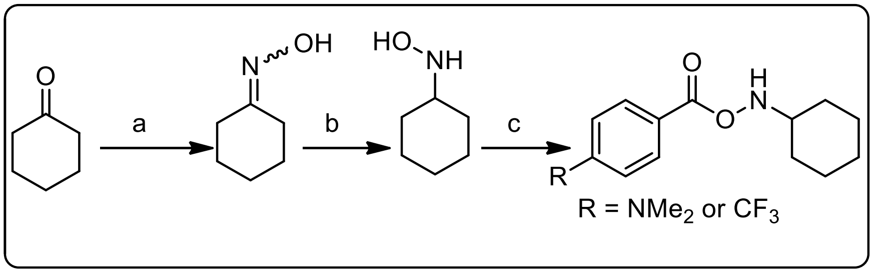

(a) A 100 ml round bottomed flask, equipped with a magnetic stirring bar, was charged with cyclohexanone (2.58 g, 25 mmol), hydroxylamine hydrochloride (2.57 g, 37.5 mmol), sodium acetate (5.13 g, 62.5 mmol), ethanol (10 mL) and water (30 mL). The reaction mixture was refluxed for 6 hrs and brought to room temperature. Thin layer chromatography in vanilline stain showed the complete conversion of cyclohexanone to cyclohexanone oxime. At this point, ethanol was evaporated using rotary evaporator. The residue was dissolved in 100 ml of EtOAc and washed sequentially with 20 mL of 1M HCl, 20 mL sat. $NaHCO_3$, 20 mL sat. NaCl, and organic layer was dried over Na_2SO_4.

(b) To a stirring solution of cyclohexanone oxime (2.86 g, 25 mmol, 1.0 equiv) in MeOH (30 mL) containing an altered pH strip were added solid $NaBH_3CN$ (2.32 g, 37.5 mmol, 1.5 equiv) and aqueous HCl (2.0 M, about 35 mL) over 15 min in such a way that the pH of the solution stayed within 2–3 during the duration of the addition. The reaction mixture was allowed to stir for an additional 3.5 h, and then was quenched with the addition of aqueous 15% NaOH (until pH = 10). MeOH was removed in vacuo. The remaining aqueous solution was extracted with CH_2Cl_2 (3 × 100 mL). The organic layers were combined, washed with brine (100 mL), dried over Na_2SO_4, and filtered. The filtrate was concentrated in vacuo. The crude residue was used for the next step.

(c) An oven-dried round bottom flask was charged with the corresponding benzoic acid (1.1 equiv) and CH_2Cl_2 (0.3 M). The flask was placed in an ice-bath, and 1,1'-carbonyldiimidazole (1.1 equiv) was added. The mixture was stirred at this temperature for 30 min, at which time N-alkyl hydroxylamine (1.0 equiv) solution in dry CH_2Cl_2 was added dropwise. The ice-bath was removed, and the reaction mixture was allowed to stir at room temperature for 1-2 h. The resulting mixture was passed through a pad of Celite, and rinsed with CH_2Cl_2. The filtrate was concentrated and purified by column chromatography to give the corresponding amine electrophile.

General procedure for the preparation of thioamides from corresponding picolinamides:

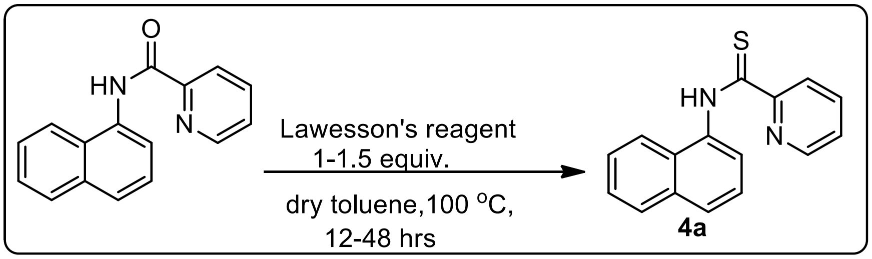

To a mixture of picolinamide (2.50 mmol) and Lawesson's reagent (0.52 g, 1.28 mmol) was added toluene (10 mL) and the solution refluxed for 12-48 hrs. The mixture was then filtered and solvent was removed under reduced pressure to afford a yellow solid material which was purified by column chromatography using solvent mixtures of petroleum ether and ethyl acetate to give the desired product (yellow solid). Using this method, thioamide of other amides were also prepared.

<u>General procedure for the preparation of picolinimidamide:</u>

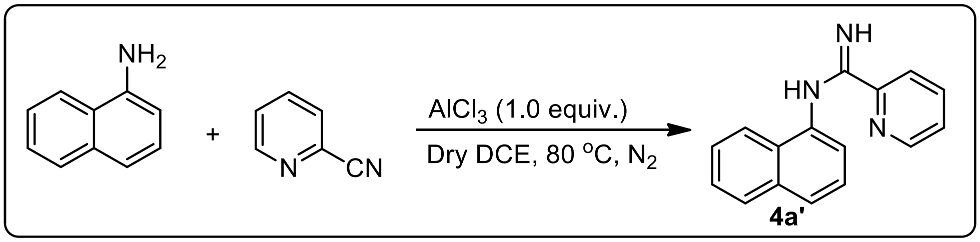

In an oven dried two-necked RB charged with 2-cyano pyridine (520 mg, 5.0 mmol) and 1-naphthylamine (715 mg, 5.0 mmol) under nitrogen atmosphere 10 ml dry DCE was added and cooled to 0 °C. Then $AlCl_3$ (665 mg, 5.0 mmol from glove box), was added portion wise to avoid overheating. The reaction mixture was stirred at this temperature for 10 minutes then at rt for 5 minutes. Then this mixture was heated to 80 °C for several hours under N_2 atmosphere. After consumtion of the starting materials. The reaction mixture was diluted with 20 mL dichloromethane and washed with 15 mL of 6N NaOH. The resulting organic layer was separated and dried over Na_2SO_4 and solvent was removed under reduced pressure. Then this crude product was washed with distilled hexane three times and dried under high vacuum to give the desired product which can be used without further purification.

<u>Procedure for the preparation of 6 and 7:</u>

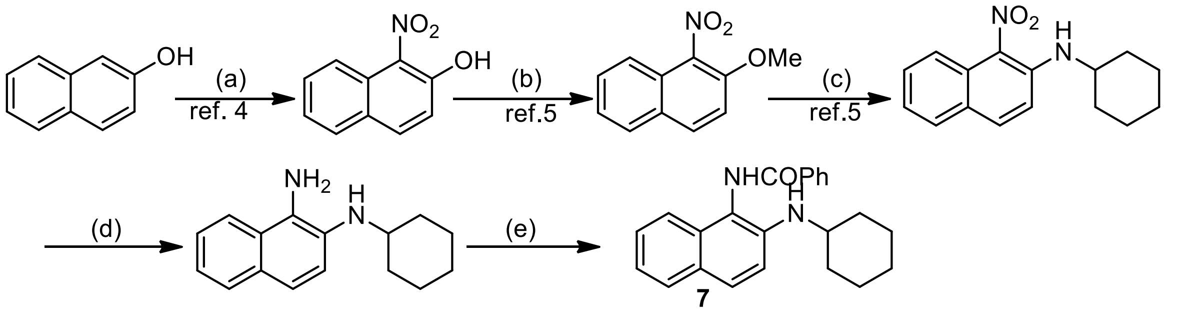

(a) $ZrO(NO_3)_2 .xH_2O$ (1.0 equiv.), acetone, r.t., 3hrs, filter,solvent evaporation, column chromatography;
(b) KOH (3.0 equiv.), MeI (2.0 equiv.), acetone, reflux, 12 hrs, cooled to r.t., work-up, column chromatography;
(c) K_2CO_3 (4.0 equiv.), $CyNH_2$ (3.0 equiv.), DMF, 80 ^oC, 16 hrs, cooled to r.t., work-up, column chromatography;
(d) Pd/C, EtOAc, H_2, r.t, 3 hrs, filter on celite bed, solvent evaporation, used for next step immidiately;
(e) Et_3N (2.0 equiv.), BzCl (1.0 equiv.), DCM, r.t., 30 min, work-up, solvent evaporation, column chromatography.

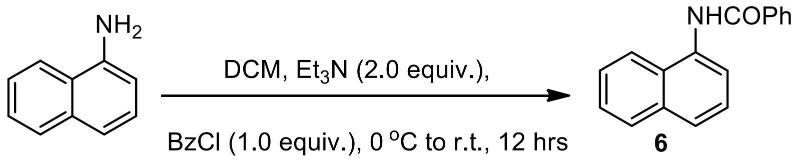

Optimization table for imidazolone product[a,b]:

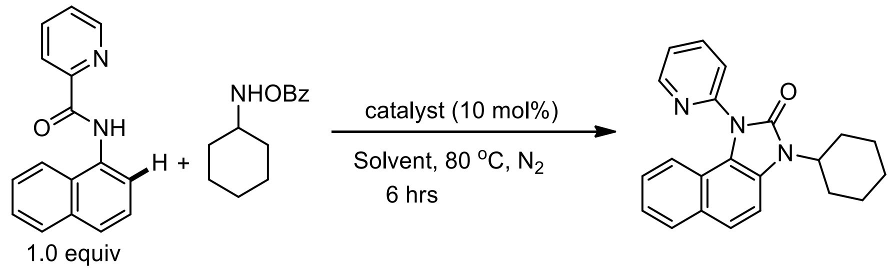

Entry	Catalyst	Amine source	additive	solvent	Yield (%)
1	$Cu(OAc)_2.H_2O$	1.0	-	DMSO	44
2	$Cu(OAc)_2.H_2O$	1.5		DMSO	50
3	$Cu(OAc)_2.H_2O$	2.5	-	H_2O	0
4	$Cu(OAc)_2.H_2O$	2.5	-	Toluene	20
5	$Cu(OAc)_2.H_2O$	2.5	-	MeCN	39
6	$Cu(OAc)_2.H_2O$	2.5	-	1,4-Dioxane	62
7	$Cu(OAc)_2.H_2O$	2.5	-	THF	52
**8**	**$Cu(OAc)_2.H_2O$**	**2.5**	**-**	**DMSO**	**90**
**10[c]**	**$Cu(OAc)_2.H_2O$**	**2.5**	**-**	**DMSO**	**93**
11[d]	$Cu(OAc)_2.H_2O$	2.5	-	DMSO	87
12[e]	$Cu(OAc)_2.H_2O$	2.5	-	DMSO	56
13[c]	Cu	2.5	-	DMSO	83
14[f]	$Cu(OAc)_2.H_2O$	2.5	PIDA	DMSO	5<
15[f]	$Cu(OAc)_2.H_2O$	2.5	$K_2S_2O_8$	DMSO	12
16[f]	$Cu(OAc)_2.H_2O$	2.5	Bz_2O_2	DMSO	32
17[g]	$Cu(OAc)_2.H_2O$	1.2	Bz_2O_2	DMSO	58
18[h]	$Cu(OAc)_2.H_2O$	2.5	-	DMSO	0
19	$Pd(OAc)_2$	2.5	-	DMSO	5<
20	$Co(OAc)_2$	2.5	-	DMSO	20<
21	$Ni(OAc)_2$	2.5	-	DMSO	n.r.
22	$Mn(OAc)_2$	2.5	-	DMSO	n.r.
23	$FeBr_2$	2.5	-	DMSO	n.r.
24	$FeCl_2$	2.5	-	DMSO	n.r.

[a]All reactions were carried out in 0.2 mmol scale. [b]Yields refer to here are overall isolated yields. [c]5.0 mol% catalyst was used. [d]1.0 mol % catalyst was used. [e]Reaction was performed at room temperature. [f]Free amine was used as amine source and oxidant 2.5 equiv. [g]1.0 equiv Bz_2O_2 was used. [h]25% *ortho* aminated product was obtained without any copper catalyst.

<u>Optimization table for imidazole product[a,b]:</u>

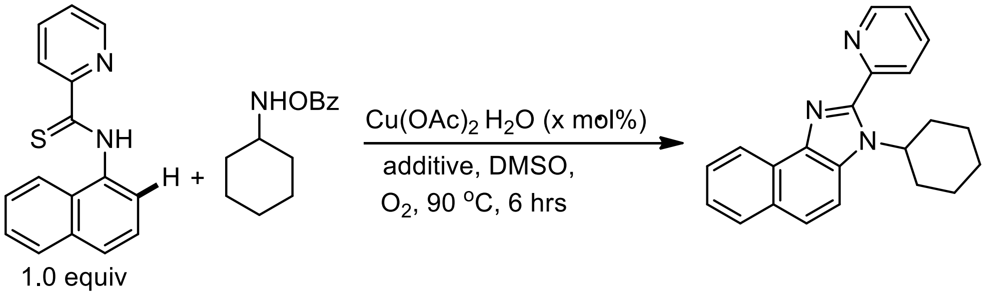

Entry	Amine source (equiv.)	$Cu(OAc)_2.H_2O$ (mol%)	Additive	Yield (%)
1[c]	2.5	10	-	38
2[d]	2.5	10	-	40
3	2.5	10	-	46

4	2.5	20	-	48
5	3.0	10	-	50
6	3.0	20	-	53
7[e]	3.0	20	-	34
8[f]	3.0	20	-	40
9[g]	3.0	20	-	40
10	3.0	20	1.2 equiv. K_2CO_3	58
11[h]	3.0	20	1.2 equiv. K_2CO_3	64
**12[i]**	**3.0**	**20**	**1.2 equiv. K_2CO_3**	**88**
13[i]	3.0	20	-	64
14[i]	3.0	10	1.2 equiv. K_2CO_3	70
15[i,j]	3.0	10	1.2 equiv. K_2CO_3	0

[a]All reactions were carried out in 0.2 mmol scale. [b]Yields refer to here are overall isolated yields. [c]Reaction was continued for 3 hrs. [d]Reaction was carried out at 100 °C. [e]Reaction was carried out in dry toluene. [f]Reaction was carried out in dry 1,4-dioxane. [g]Reaction was carried out in dry THF. [h]Reaction was carried out in air. [i]Reaction was carried under O_2 atm.[j]Reaction was performed with substrate **1a** instead of **4a**.

Optimization table for imidazolone product in 2-substituted aniline system[a,b]:

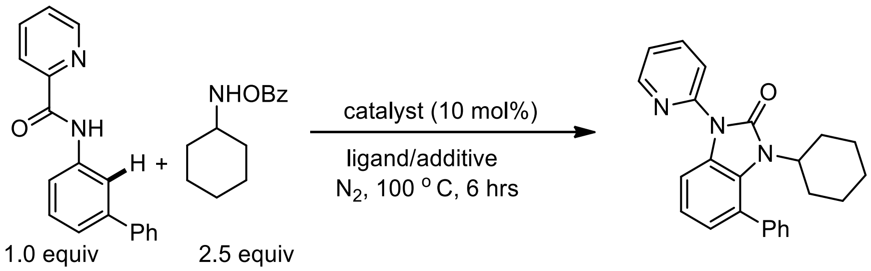

Entry	Catalyst	Additive (1.5 equiv.)	Ligand (20 mol %)	Solvent	Yield (%)
1[c]	$Cu(OAc)_2.H_2O$	-	-	DMSO	20
2[c]	$Pd(OAc)_2$	-	-	DMSO	nr
3[c]	$Co(OAc)_2.4H_2O$	-	-	DMSO	5 <
**4**	**$Cu(OAc)_2.H_2O$**	**-**	**-**	**DMSO**	**40**
5[d]	$Cu(OAc)_2.H_2O$	-	-	DMSO	27
6[e]	$Cu(OAc)_2.H_2O$	-	-	DMSO	27
7	$Cu(OAc)_2.H_2O$	-	-	Toluene	20
8	$Cu(OAc)_2.H_2O$	-	-	1,4-dioxane	32
9	$Cu(OAc)_2.H_2O$	-	-	MeCN	28
10	$Cu(OAc)_2.H_2O$	-	-	DCE	28
11	$Cu(OAc)_2.H_2O$	K_2CO_3	-	DMSO	15
12	$Cu(OAc)_2.H_2O$	$NaHCO_3$	-	DMSO	37

13	$Cu(OAc)_2.H_2O$	Li_2CO_3	-	DMSO	35
14	$Cu(OAc)_2.H_2O$	K_2CO_3	-	DMSO	32
15	$Cu(OAc)_2.H_2O$	Cs_2CO_3	-	DMSO	nr
16	$Cu(OAc)_2.H_2O$	$KHCO_3$	-	DMSO	30
17	$Cu(OAc)_2.H_2O$	LiO^tBu	-	DMSO	nr
18	$Cu(OAc)_2.H_2O$	$NaHCO_3$	dppb	DMSO	39
19	$Cu(OAc)_2.H_2O$	$NaHCO_3$	dppm	DMSO	27
20	$Cu(OAc)_2.H_2O$	$NaHCO_3$	dppe	DMSO	35
21	$Cu(OAc)_2.H_2O$	$NaHCO_3$	dppp	DMSO	27
22	$Cu(OAc)_2.H_2O$	$NaHCO_3$	dppf	DMSO	20
23	$Cu(OAc)_2.H_2O$	$NaHCO_3$	dppen	DMSO	27
24	$Cu(OAc)_2.H_2O$	-	1,10-phen	DMSO	18
25	$Cu(OAc)_2.H_2O$	-	PPh_3	DMSO	35
26	$Cu(OAc)_2.H_2O$	-	x-phos	DMSO	35
27	$Cu(OAc)_2.H_2O$	-	dtbbpy	DMSO	35
28	$Cu(OAc)_2.H_2O$	-	terpyridine	DMSO	35
29	$Cu(OTf)_2$	-	-	DMSO	35
30	Cu_2O	-	-	DMSO	33
31	$CuSO_4.5H_2O$	-	-	DMSO	35
32	CuCl	-	-	DMSO	28
33	$Cu(OAc)_2$	-	-	DMSO	35
34	$Cu(OAc)_2.H_2O$	Zn dust	-	DMSO	30
35	$Cu(OAc)_2.H_2O$	$Mn(OAc)_2$	-	DMSO	28
36	$Cu(OAc)_2.H_2O$	$Mn(OAc)_3$	-	DMSO	28

[a]All reactions were carried out in 0.2 mmol scale. [b]Yields refer to here are overall isolated yields. [c]Temperature was 80 °C. [d]20 mol % catalyst was used. [e]50 mol % catalyst was used.

Optimization table for amination product in aniline system[a,b]:

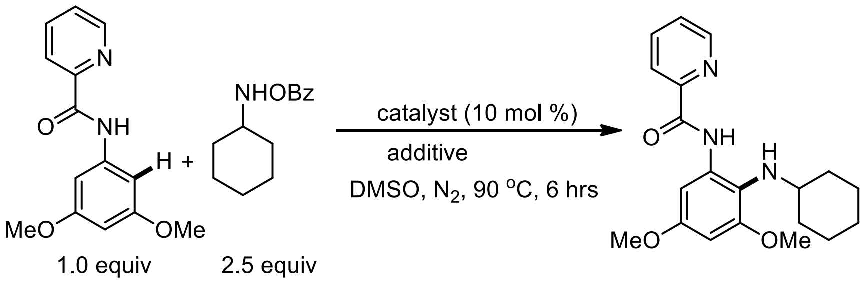

Entry	catalyst	additive	Yield (%)
1	$Cu(OAc)_2.H_2O$	-	35
2[c]	$Cu(OAc)_2.H_2O$	-	25
3	$Cu(OTf)_2$	-	10<
4	$Pd(OAc)_2$	-	nr
5	$Cu(OAc)_2.H_2O$	1,10-phen	15
6	$Cu(OAc)_2.H_2O$	K_2CO_3	58
7[d]	$Cu(OAc)_2.H_2O$	K_2CO_3	50

8[e]	$Cu(OAc)_2.H_2O$	K_2CO_3	54
9	$Cu(OAc)_2.H_2O$	Na_2CO_3	45
10	$Cu(OAc)_2.H_2O$	Cs_2CO_3	43
11	$Cu(OAc)_2.H_2O$	KOAc	30
**12**	**$Cu(OAc)_2.H_2O$**	**LiO^tBu**	**72**

[a]All reactions were carried out in 0.2 mmol scale. [b]Yields refer to here are overall isolated yields. [c]Temperature 100 °C and time 12 hrs. [d]Air. [e]O_2 atm.

Representative Procedure 1 for imidazolone:

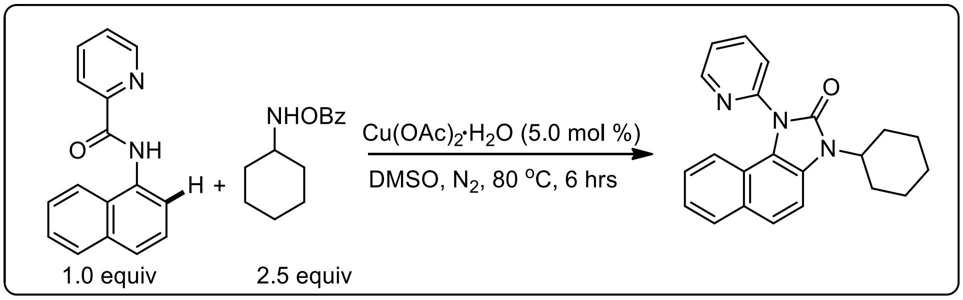

In an oven dried 15 mL sealed tube containing a stir bar was added corresponding picolinamide (0.2 mmol, 1.0 equiv), *O*-benzoylhydroxylamine (0.5 mmol, 2.5 equiv.) and $Cu(OAc)_2{\cdot}H_2O$ (0.01 mmol). Dry DMSO (2mL) was then added and N_2 gas was purged for 2 minutes. The mixture was stirred at 80 °C for 6 hrs. After allotted time the reaction mixture was cooled to room temperature. The mixture was diluted with EtOAc (15 mL) and washed with saturated aq. $NaHCO_3$ solution (25mL), followed by brine solution (25 mL) and dried over Na_2SO_4, and evaporated in *vacuo*. The crude mixture was loaded on a silica gel column chromatography and purified using (Hexane/EtOAc) to give the desired imidazolone product.

Representative Procedure for imidazole:

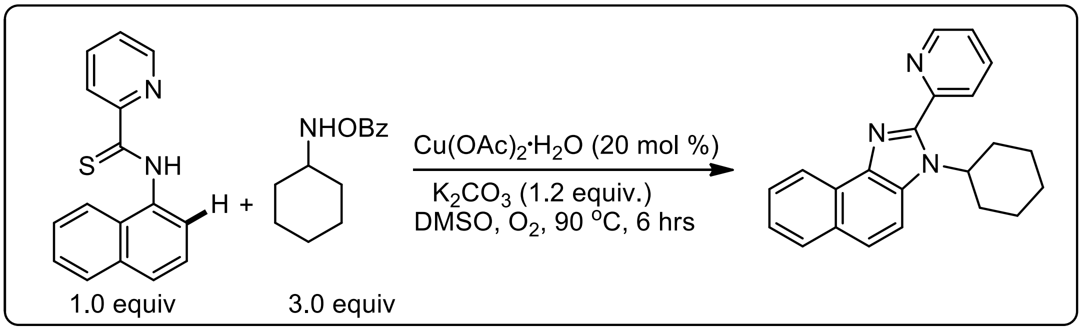

In an oven dried 15 mL sealed tube containing a stir bar was added corresponding thiopicolinamide (0.2 mmol, 1.0 equiv), *O*-benzoylhydroxylamine (0.5 mmol, 3.0 equiv.), $Cu(OAc)_2 \cdot H_2O$ (0.04 mmol) and K_2CO_3 (0.24 mmol). Dry DMSO (2mL) was then added and O_2 gas was purged for 2 minutes. **[Note**: amount of O_2 should be sufficient for better reaction and so large amount of empty space is required. For scale up reaction (2.0 mmol) 100 mL pressure tube was used**]**. The mixture was stirred at 90 °C for 6 hrs. After allotted time the reaction mixture was cooled to room temperature. The mixture was diluted with EtOAc (15 mL) and washed with saturated aq. $NaHCO_3$ solution (25mL), followed by brine solution (25 mL) and dried over Na_2SO_4, and evaporated in *vacuo*. The crude mixture was loaded on a silica gel column chromatography and purified using (Hexane/EtOAc) to give the desired imidazole product.

Representative Procedure 2 for imidazolone in 2-substituted aniline system:

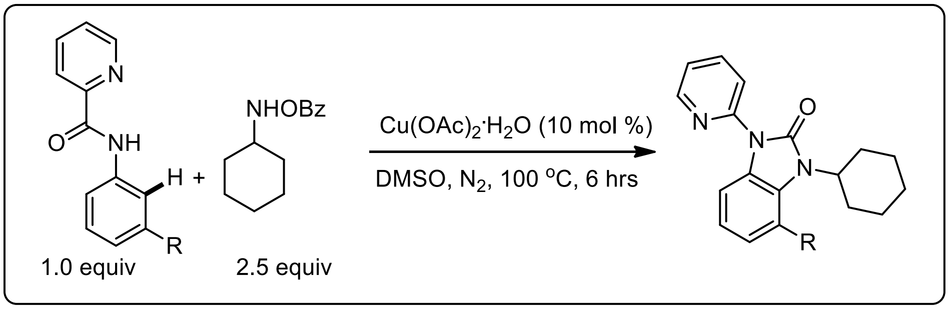

In an oven dried 15 mL sealed tube containing a stir bar was added corresponding picolinamide (0.2 mmol, 1.0 equiv), *O*-benzoylhydroxylamine (0.5 mmol, 2.5 equiv.) and $Cu(OAc)_2 \cdot H_2O$ (0.02 mmol). Dry DMSO (2mL) was then added and N_2 gas was purged for 2 minutes. The mixture was stirred at 100 °C for 6 hrs. After allotted time the reaction mixture was cooled to room temperature. The mixture was diluted with EtOAc (15 mL) and washed with saturated aq. $NaHCO_3$ solution (25mL), followed by brine solution (25 mL) and dried over Na_2SO_4, and evaporated in *vacuo*. The crude mixture was loaded on a silica gel column chromatography and purified using (Hexane/EtOAc) to give the desired imidazolone product.

Representative Procedure for amination in aniline system:

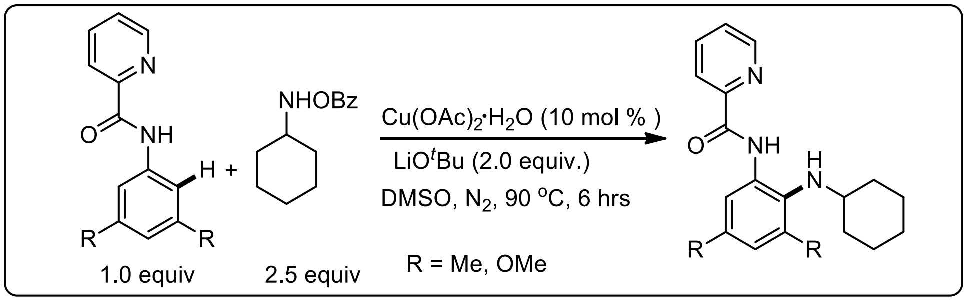

In an oven dried 15 mL sealed tube containing a stir bar was added corresponding picolinamide (0.2 mmol, 1.0 equiv), *O*-benzoylhydroxylamine (0.5 mmol, 2.5 equiv.), LiO^{t}Bu (0.4 mmol, 2.0 equiv.) and $Cu(OAc)_2 \cdot H_2O$ (0.02 mmol). Dry DMSO (2mL) was then added and N_2 gas was purged for 2 minutes. The mixture was stirred at 90 ^{o}C for 6 hrs. After allotted time the reaction mixture was cooled to room temperature. The mixture was diluted with EtOAc (15 mL) and washed with saturated aq. $NaHCO_3$ solution (25mL), followed by brine solution (25 mL) and dried over Na_2SO_4, and evaporated in *vacuo*. The crude mixture was loaded on a silica gel column chromatography and purified using (Hexane/EtOAc) to give the desired amination product.

<u>Procedure for synthesis of compound 1a'</u>:

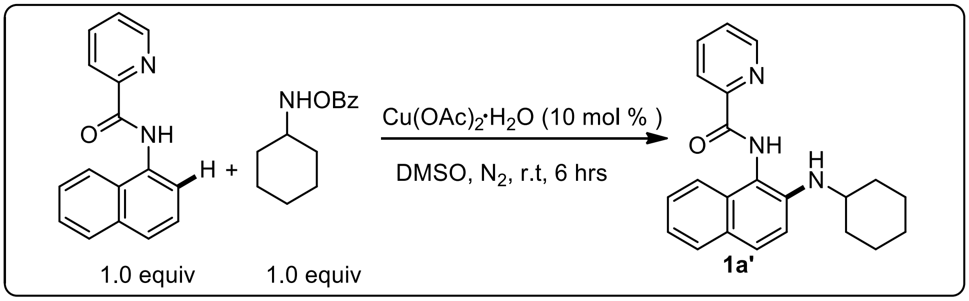

In an oven dried RB containing a stir bar was added picolinamide **1a** (2 mmol, 1.0 equiv), *O*-benzoylhydroxylamine (2 mmol, 1.0 equiv.) and $Cu(OAc)_2 \cdot H_2O$ (0.2 mmol). Dry DMSO (15 mL) was then added and N_2 gas was purged for 2 minutes. The mixture was stirred at room temperature for 6 hrs. The mixture was diluted with EtOAc (100 mL) and washed with saturated aq. $NaHCO_3$ solution (50 mL), followed by brine solution (50 mL) and dried over Na_2SO_4, and evaporated in *vacuo*. The crude mixture was loaded on a silica gel column chromatography and purified using (Hexane/EtOAc) to give the amination product.

<u>Deprotection of pyridyl group of Benzimidazolone product</u>:

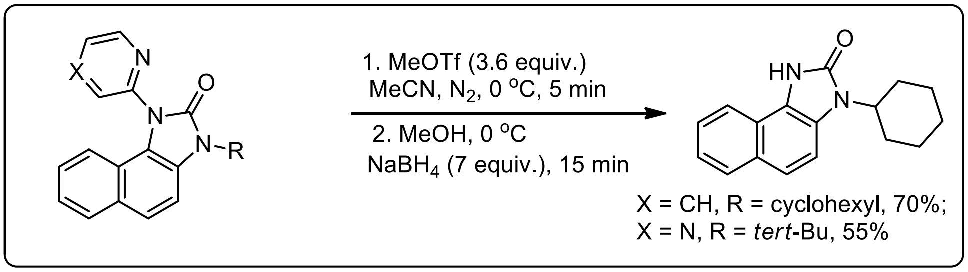

This deprotection was done according a condition for the deprotection of N-pyridyl group.[6] In an oven dried RB containing a stir bar was added the benzimidazolone and dry MeCN and it was cooled to 0 °C in an ice-bath under N_2 atmosphere. Ice-cooled MeOTf (3.6 equiv.) was added dropwise to this solution. After addition the mixture was stirred at r.t. for 10 minutes. Then to this mixture MeOH (4 mL) was added and further cooled to 0 °C in an ice-bath. $NaBH_4$ (7.0 equiv.) was added portion-wise and stirred at this temperature for 15 minutes. After that the solvent was evaporated and diluted with EtOAc, washed with water and dried over Na_2SO_4. This was concentrated under vacuum followed by column chromatography gave the desired product.

<u>Time dependent ^{1}H NMR experiment for mechanistic determination of intermediate</u>:

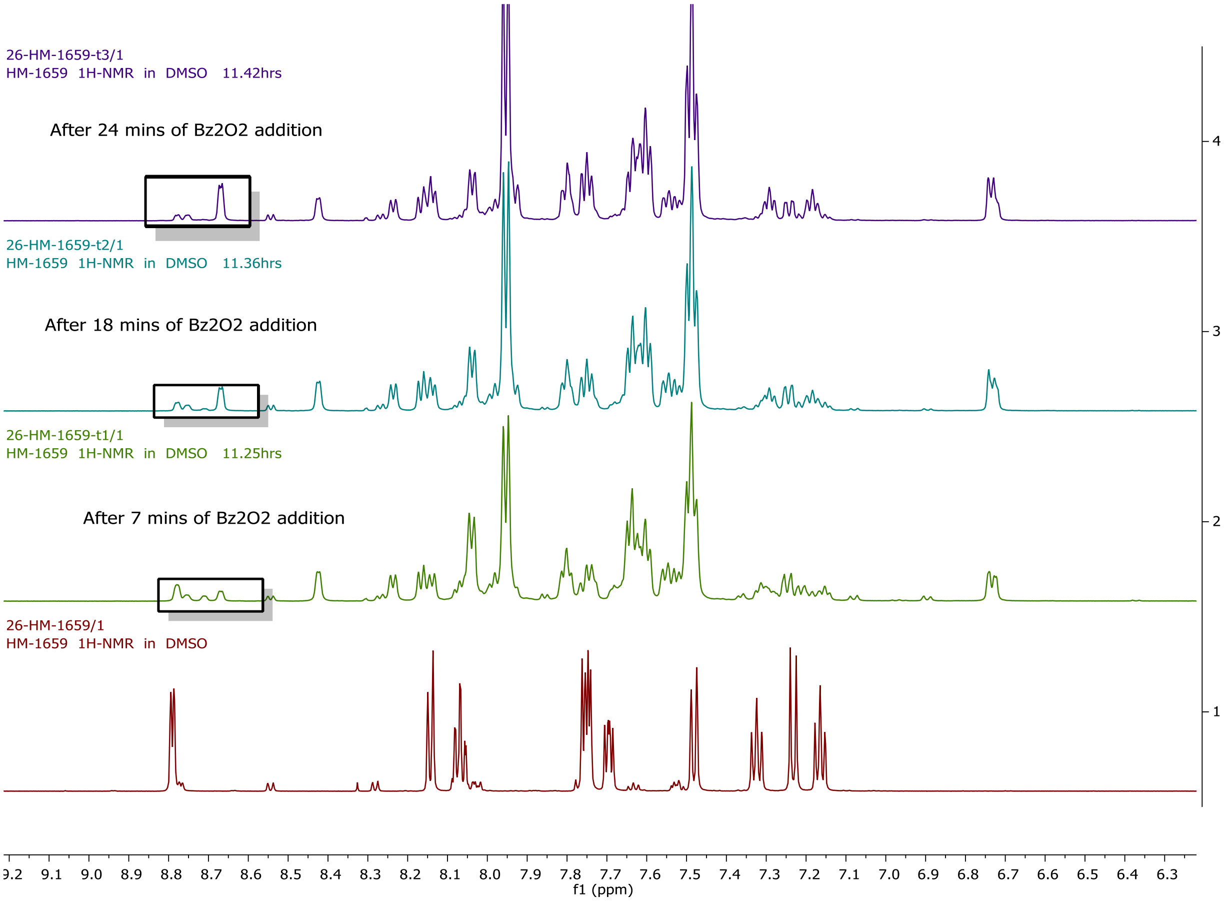

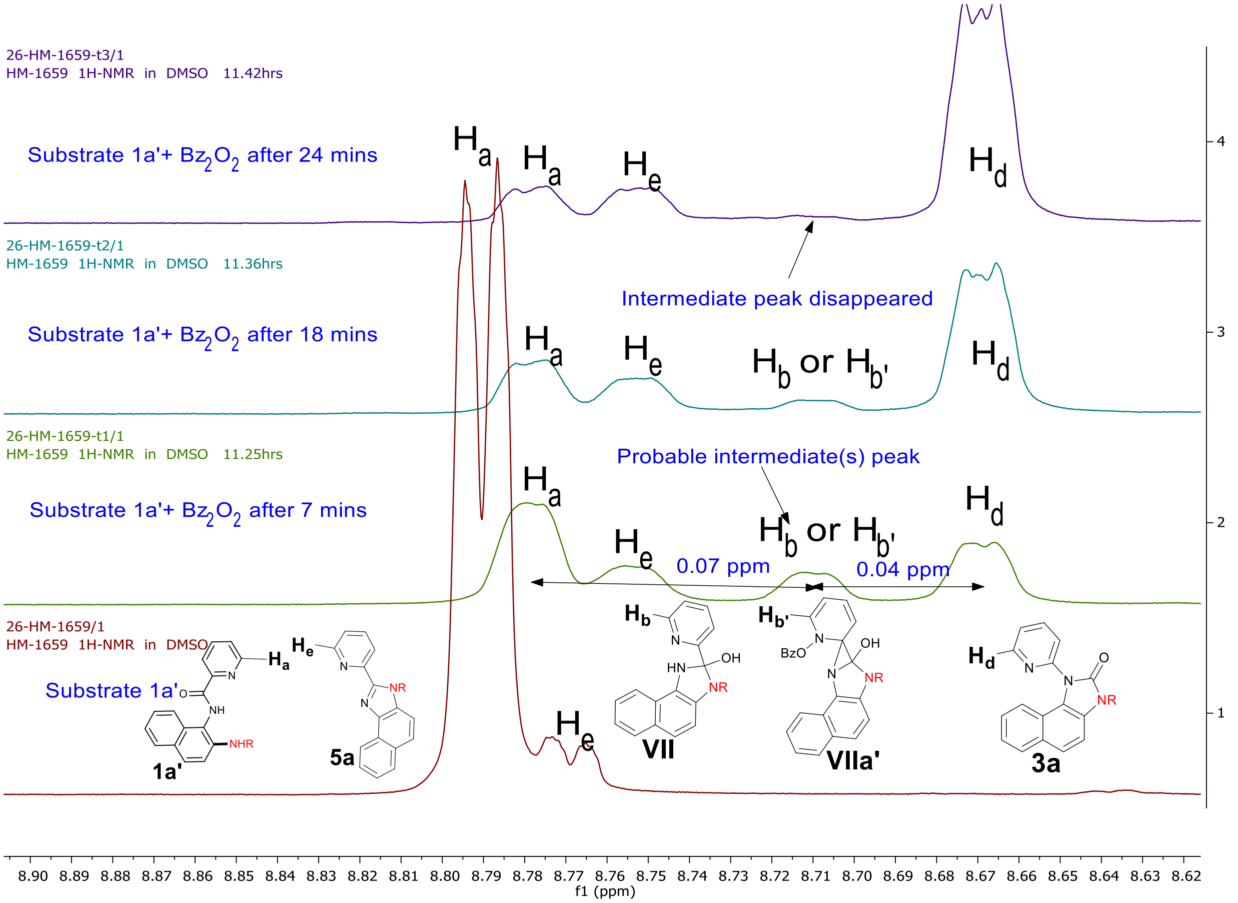

Crystal structure:

The crystals were grown in dichloromethane solvent. The pure compound was dissolved in dichloromethane slow evaporation led to the crystal **4a**. The crystal data was collected in X-ray spectroscopy (Bruker Kappa Apex-2, CCD Area Detector), and the data was analyzed using OLEX2 software. The structure is given below. The corresponding cif file has been uploaded separately as supporting information.

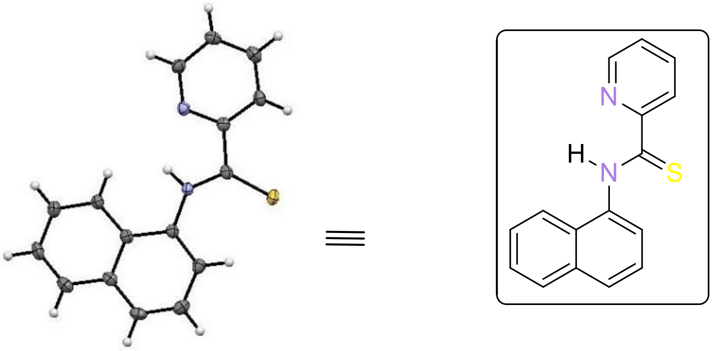

Thermal ellipsoid plot of **4a**. Ellipsoids are represented with 50% probability.

X-ray determined molecular structure of **4a,** CCDC: 2025280

Identification code	HM_643S_0m_a
Empirical formula	$C_{16}H_{12}N_2S$
Formula weight	264.34
Temperature/K	100.0
Crystal system	monoclinic
Space group	$P2_1/c$
a/Å	6.1282(2)
b/Å	14.1422(4)
c/Å	14.4603(4)
α/°	90
β/°	95.2550(10)
γ/°	90
Volume/Å^3	1247.95(6)
Z	4
ρ_{calc}g/cm^3	1.407
μ/mm^{-1}	2.167
F(000)	552.0
Crystal size/mm^3	0.2 × 0.2 × 0.2
Radiation	CuKα (λ = 1.54178)
2Θ range for data collection/°	8.764 to 133.626
Index ranges	$-7 \le h \le 7$, $-16 \le k \le 16$, $-17 \le l \le 17$
Reflections collected	19122
Independent reflections	2213 [R_{int} = 0.0469, R_{sigma} = 0.0251]
Data/restraints/parameters	2213/0/172
Goodness-of-fit on F^2	1.078

Final R indexes [I>=2σ (I)]	R_1 = 0.0361, wR_2 = 0.0921
Final R indexes [all data]	R_1 = 0.0373, wR_2 = 0.0929
Largest diff. peak/hole / e Å^{-3}	0.26/-0.32

The crystals were grown in dichloromethane solvent. The pure compound was dissolved in dichloromethane slow evaporation led to the crystal **3aj**. The crystal data was collected in X-ray spectroscopy (Bruker Kappa Apex-2, CCD Area Detector), and the data was analyzed using OLEX2 software. The structure is given below. The corresponding cif file has been uploaded separately as supporting information.

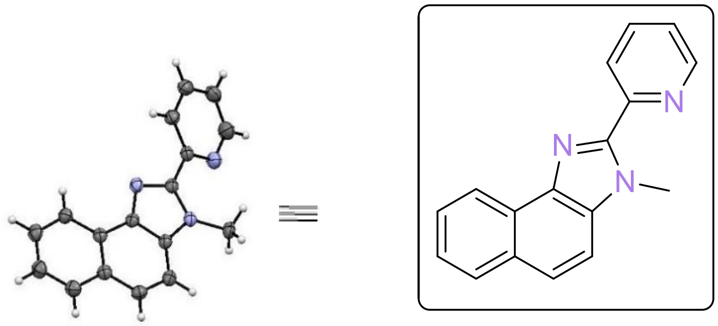

Thermal ellipsoid plot of **3aj**. Ellipsoids are represented with 50% probability.

X-ray determined molecular structure of **3aj,** CCDC: 2025266

Identification code	HM_430A_0m_a
Empirical formula	$C_{18}H_{15}N_2$
Formula weight	259.32
Temperature/K	100.0
Crystal system	monoclinic
Space group	$P2_1/n$
a/Å	10.9333(3)

b/Å	7.3400(2)
c/Å	16.2518(5)
α/°	90
β/°	98.6550(10)
γ/°	90
Volume/Å^3	1289.36(6)
Z	4
ρ_{calc}g/cm^3	1.336
μ/mm^{-1}	0.612
F(000)	548.0
Crystal size/mm^3	0.2 × 0.2 × 0.2
Radiation	CuKα (λ = 1.54178)
2Θ range for data collection/°	13.262 to 132.918
Index ranges	-12 ≤ h ≤ 12, -8 ≤ k ≤ 8, -19 ≤ l ≤ 17
Reflections collected	18364
Independent reflections	2259 [R_{int} = 0.0807, R_{sigma} = 0.0459]
Data/restraints/parameters	2259/0/183
Goodness-of-fit on F^2	1.056
Final R indexes [I>=2σ (I)]	R_1 = 0.0733, wR_2 = 0.2003
Final R indexes [all data]	R_1 = 0.0760, wR_2 = 0.2033
Largest diff. peak/hole / e Å^{-3}	0.55/-0.66

The crystals were grown in dichloromethane solvent. The pure compound was dissolved in dichloromethane slow evaporation led to the crystal **5h**. The crystal data was collected in X-ray spectroscopy (Bruker Kappa Apex-2, CCD Area Detector), and the data was analyzed using OLEX2 software. The structure is given below. The corresponding cif file has been uploaded separately as supporting information.

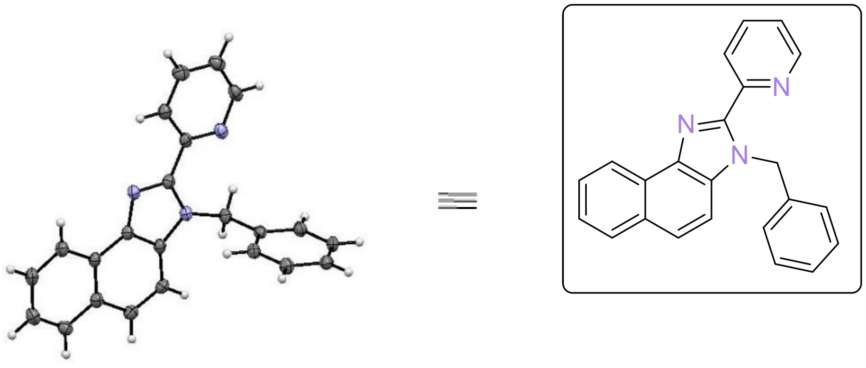

Thermal ellipsoid plot of **5h**. Ellipsoids are represented with 50% probability.

X-ray determined molecular structure of **5h,** CCDC: 2025275

Identification code	K_101_0m_a
Empirical formula	$C_{23}H_{18}N_3$
Formula weight	336.40
Temperature/K	100.0
Crystal system	monoclinic
Space group	$P2_1/n$
a/Å	11.8246(5)
b/Å	8.7668(4)
c/Å	16.2183(7)
α/°	90
β/°	90.457(2)
γ/°	90
Volume/Å^3	1681.20(13)
Z	4
ρ_{calc}g/cm^3	1.329
μ/mm^{-1}	0.618
F(000)	708.0
Crystal size/mm^3	0.35 × 0.29 × 0.28
Radiation	CuKα (λ = 1.54178)

2Θ range for data collection/°	10.91 to 133.22
Index ranges	$-14 \le h \le 13$, $-10 \le k \le 10$, $-18 \le l \le 19$
Reflections collected	16494
Independent reflections	2930 [R_{int} = 0.0772, R_{sigma} = 0.0536]
Data/restraints/parameters	2930/0/235
Goodness-of-fit on F^2	1.105
Final R indexes [I>=2σ (I)]	R_1 = 0.0624, wR_2 = 0.1540
Final R indexes [all data]	R_1 = 0.0667, wR_2 = 0.1575
Largest diff. peak/hole / e $Å^{-3}$	0.25/-0.66

The crystals were grown in dichloromethane solvent. The pure compound was dissolved in dichloromethane slow evaporation led to the crystal **3ad**. The crystal data was collected in X-ray spectroscopy (Bruker Kappa Apex-2, CCD Area Detector), and the data was analyzed using OLEX2 software. The structure is given below. The corresponding cif file has been uploaded separately as supporting information.

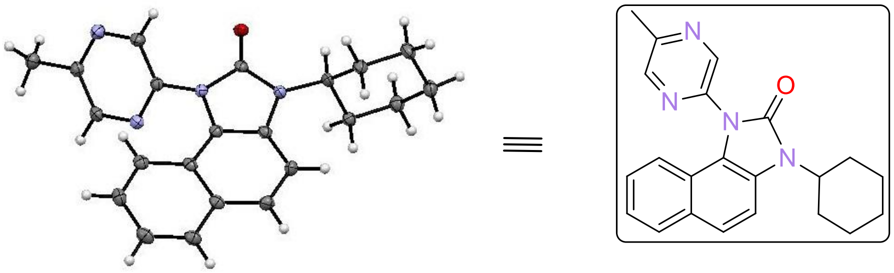

Thermal ellipsoid plot of **3ad**. Ellipsoids are represented with 50% probability.

X-ray determined molecular structure of **3ad,** CCDC: 2025269

Identification code	HM_583_0m_a
Empirical formula	$C_{2.44}H_{2.44}N_{0.44}O_{0.11}$
Formula weight	39.83
Temperature/K	100.0
Crystal system	triclinic
Space group	P-1
a/Å	7.9957(2)
b/Å	10.4307(2)
c/Å	12.5078(3)
α/°	67.7270(10)
β/°	89.0730(10)
γ/°	71.4000(10)
Volume/Å^3	908.36(4)
Z	18
ρ_{calc}g/cm^3	1.310
μ/mm^{-1}	0.658
F(000)	380.0
Crystal size/mm^3	0.80 × 0.28 × 0.27
Radiation	CuKα (λ = 1.54178)
2Θ range for data collection/°	7.692 to 133.402
Index ranges	-9 ≤ h ≤ 8, -12 ≤ k ≤ 12, -14 ≤ l ≤ 14
Reflections collected	28821
Independent reflections	3182 [R_{int} = 0.0634, R_{sigma} = 0.0334]
Data/restraints/parameters	3182/0/245

Goodness-of-fit on F^2	1.088
Final R indexes [I>=2σ (I)]	R_1 = 0.0424, wR_2 = 0.0985
Final R indexes [all data]	R_1 = 0.0438, wR_2 = 0.0994
Largest diff. peak/hole / e Å^{-3}	0.21/-0.25

The crystals were grown in dichloromethane solvent. The pure compound was dissolved in dichloromethane slow evaporation led to the crystal **5b**. The crystal data was collected in X-ray spectroscopy (Bruker Kappa Apex-2, CCD Area Detector), and the data was analyzed using OLEX2 software. The structure is given below. The corresponding cif file has been uploaded separately as supporting information.

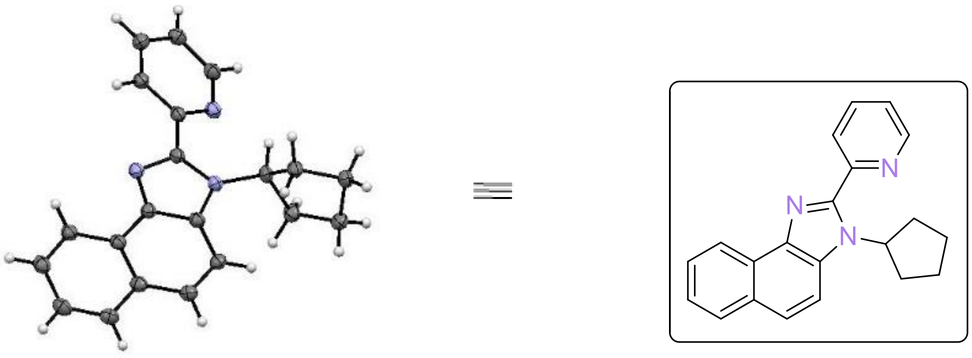

Thermal ellipsoid plot of **5b**. Ellipsoids are represented with 50% probability.

X-ray determined molecular structure of **5b,** CCDC: 2025279

Identification code	HM_788_1_0m_a
Empirical formula	$C_{21}H_{19}N_3$
Formula weight	313.41
Temperature/K	100.0
Crystal system	monoclinic
Space group	$P2_1/c$
a/Å	15.2933(10)
b/Å	5.6759(9)
c/Å	18.003(3)
α/°	90
β/°	99.217(8)
γ/°	90
Volume/Å^3	1542.5(4)
Z	4
ρ_{calc}g/cm^3	1.3495
μ/mm^{-1}	0.627
F(000)	665.9
Crystal size/mm^3	0.2 × 0.2 × 0.2
Radiation	Cu Kα (λ = 1.54178)
2Θ range for data collection/°	5.86 to 143.7
Index ranges	-18 ≤ h ≤ 18, -6 ≤ k ≤ 6, -21 ≤ l ≤ 22
Reflections collected	22846
Independent reflections	2934 [R_{int} = 0.0804, R_{sigma} = 0.0465]
Data/restraints/parameters	2934/0/218
Goodness-of-fit on F^2	1.048

Final R indexes [I>=2σ (I)]	R_1 = 0.0531, wR_2 = 0.1361
Final R indexes [all data]	R_1 = 0.0570, wR_2 = 0.1400
Largest diff. peak/hole / e $Å^{-3}$	0.22/-0.34

Spectral data:

***N*-(naphthalen-1-yl)pyridine-2-carbothioamide (4a)**

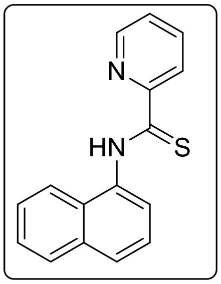

Column chromatography (SiO_2, eluting with 95:5 hexane/ethyl acetate) afforded the desired product as a yellow solid, mp 124-126 °C.

^{1}H NMR (400 MHz, $CDCl_3$): δ 12.36 (s, 1H), 8.84 (dt, J_1 = 8.0 Hz, J_2 = 0.8 Hz, 1H), 8.63-8.61 (m, 1H), 8.33 (d, J = 7.2 Hz, 1H), 8.03-7.99 (m, 1H), 7.95-7.89 (m, 2H), 7.85 (d, J = 8.4 Hz, 1H), 7.60-7.49 (m, 4H); ^{13}C NMR (100 MHz, $CDCl_3$): δ 190.2, 151.5, 146.8, 137.7, 134.4, 134.3, 128.9, 128.4, 127.7, 126.8, 126.5, 126.3, 125.5, 125.3, 122.9, 121.6; HRMS (ESI, m/z) calcd. For $C_{16}H_{13}N_2S$ $[M+H]^+$: 265.0799; found: 265.0802.

***N*-(naphthalen-1-yl)picolinimidamide (4a')**

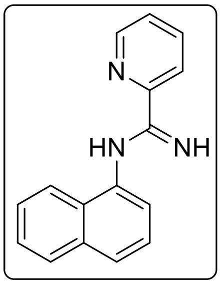

Washing with distilled hexane afforded the desired product as a violet solid, mp 118-120 °C.

^{1}H NMR (400 MHz, $CDCl_3$): δ 8.63 (d, J = 8.0 Hz, 1H), 8.61-8.59 (m, 1H), 8.10 (d, J = 8.0 Hz, 1H), 7.89-7.83 (m, 2H), 7.59 (d, J = 8.4 Hz, 1H), 7.49-7.38 (m, 4H), 7.09 (d, J = 7.6 Hz, 1H), 6.29-5.17 (br. S); ^{13}C NMR (100 MHz, $CDCl_3$): δ 152.9, 151.3, 148.1, 137.0, 134.8, 128.1, 127.4, 126.4, 126.2, 125.4, 123.9, 123.4, 122.0, 116.3; HRMS (ESI, m/z) calcd. For $C_{16}H_{14}N_3$ $[M+H]^+$: 248.1188; found: 248.1185.

***N*-(2-(cyclohexylamino)naphthalen-1-yl)picolinamide (1a')**

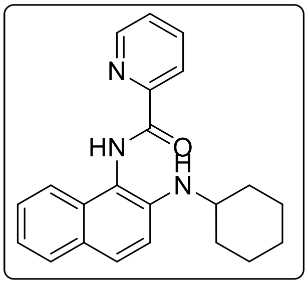

Column chromatography (SiO_2, eluting with 85:15 hexane/ethyl acetate) afforded the desired product as a brown gummy liquid.

^{1}H NMR (400 MHz, $CDCl_3$): δ 9.76 (s, 1H), 8.68-8.67 (m, 1H), 8.34 (dt, J_1 = 7.6 Hz, J_2 = 1.2 Hz, 1H), 7.91 (td, J_1 = 7.6 Hz, J_2 = 1.6 Hz, 1H), 7.73-7.68 (m, 3H), 7.51-7.48 (m, 1H), 7.41-7.37 (m, 1H), 7.23-7.19 (m, 2H), 3.51-3.44 (m, 1H), 2.12-2.08 (m, 2H), 1.79-1.74 (m, 2H), 1.65-1.61 (m, 1H), 1.43-1.21 (m, 5H); ^{13}C NMR (100 MHz, $CDCl_3$): δ 163.4, 149.8, 148.4, 141.5, 137.7, 131.5, 128.7, 128.3, 127.3, 127.0, 126.6, 122.8, 122.1, 120.5, 115.4, 113.0, 51.9, 33.8, 25.9, 25.1; HRMS (ESI, m/z) calcd. For $C_{22}H_{24}N_3O$ $[M+H]^+$: 346.1919; found: 346.1922.

***N*-(naphthalen-1-yl)benzamide (6)**

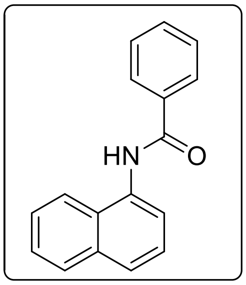

Column chromatography (SiO_2, eluting with 90:10 hexane/ethyl acetate) afforded the desired product as white fluffy solid.

^{1}H NMR (400 MHz, $CDCl_3$): δ 8.27 (s, 1H), 7.98-7.95 (m, 3H), 7.90-7.87 (m, 2H), 7.73 (d, J = 8.4 Hz, 1H), 7.58-7.55 (m, 1H), 7.51-7.46 (m, 5H); ^{13}C NMR (100 MHz, $CDCl_3$): δ 166.4, 134.9, 134.3, 132.5, 132.0, 128.94, 128.90, 127.6, 127.3, 126.5, 126.2, 126.1, 125.9, 121.5, 120.9.

***N*-(2-(cyclohexylamino)naphthalen-1-yl)benzamide (7)**

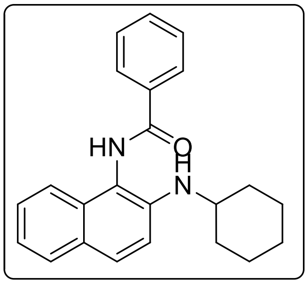

Column chromatography (SiO_2, eluting with 90:10 hexane/ethyl acetate) afforded the desired product as white fluffy solid.

^{1}H NMR (400 MHz, $CDCl_3$ + CD_3OD): δ 8.01-7.98 (m, 2H), 7.68-7.64 (m, 2H), 7.58-7.51 (m, 2H), 7.49-7.45 (m, 2H), 7.33-7.28 (m, 1H), 7.17-7.01 (m, 2H), 3.39-3.30 (m, 1H), 1.98 (d, *J* = 12.8 Hz, 2H), 1.69-1.61 (m, 2H), 1.58-1.54 (m, 1H), 1.35-1.12 (m, 5H); ^{13}C NMR (100 MHz, $CDCl_3$+ CD_3OD): δ 171.8, 137.7, 136.1, 135.6, 133.0, 132.8, 132.1, 132.0, 131.6, 131.0, 126.5, 124.8, 119.8, 56.8, 37.3, 33.6, 29.6, 28.9; HRMS (ESI, m/z) calcd. For $C_{23}H_{25}N_2O$ $[M+H]^+$: 345.1967; found: 345.1964.

***O*-benzoyl-*N*-cyclohexylhydroxylamine (2a)**

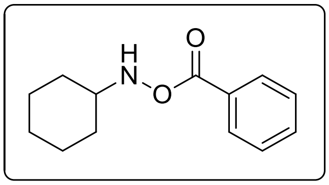

Column chromatography (SiO_2, eluting with 98:2 hexane/ethyl acetate) afforded the desired product as colourless liquid.
^{1}H NMR (400 MHz, $CDCl_3$): δ 8.02-7.99 (m, 2H), 7.58-7.53 (m, 1H), 7.45-7.41 (m, 2H), 3.07-2.99 (m, 1H), 1.98-1.94 (m, 2H), 1.81-1.74 (m, 2H), 1.64-1.59 (m, 1H), 1.31-1.17 (m, 5H); ^{13}C NMR (100 MHz, $CDCl_3$): δ 167.0, 133.3, 129.4, 128.6, 59.9, 30.4, 25.9, 24.1.

***O*-benzoyl-*N*-butylhydroxylamine (2g)**

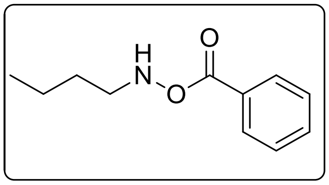

Column chromatography (SiO_2, eluting with 98:2 hexane/ethyl acetate) afforded the desired product as colourless liquid.

^{1}H NMR (400 MHz, $CDCl_3$): δ 8.03-7.99 (m, 2H), 7.86 (br. S, 1H), 7.58-7.54 (m, 1H), 7.46-7.42 (m, 2H), 3.13 (t, *J* = 7.2 Hz, 2H), 1.63-1.56 (m, 2H), 1.47-1.38 (m, 2H), 0.94 (t, *J* = 7.6 Hz, 3H); ^{13}C NMR (100 MHz, $CDCl_3$): δ 167.0, 133.4, 129.4, 128.6, 52.4, 29.3, 20.3, 13.9.

***O*-benzoyl-*N*-*iso*butylhydroxylamine (2h)**

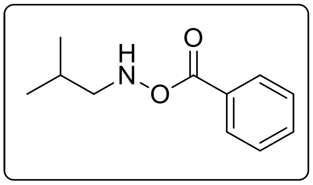

Column chromatography (SiO_2, eluting with 98:2 hexane/ethyl acetate) afforded the desired product as colourless liquid.
^{1}H NMR (400 MHz, $CDCl_3$): δ 8.02-7.99 (m, 2H), 7.96 (br. S, 1H), 7.58-7.54 (m, 1H), 7.46-7.41 (m, 2H), 2.95 (d, *J* = 6.8 Hz, 2H), 1.99-1.89 (m, 1H), 1.00 (d, *J* = 6.8 Hz, 6H); ^{13}C NMR (100 MHz, $CDCl_3$): δ 166.9, 133.4, 129.4, 128.6, 60.2, 26.5, 20.6.

***O*-benzoyl-*N*-(*sec*-butyl)hydroxylamine (2i)**

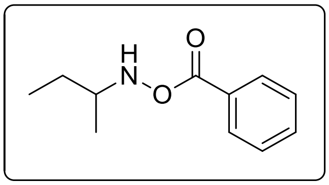

Column chromatography (SiO_2, eluting with 98:2 hexane/ethyl acetate) afforded the desired product as colourless liquid.
^{1}H NMR (400 MHz, $CDCl_3$): δ 8.03-7.99 (m, 2H), 7.58-7.54 (m, 1H), 7.46-7.42 (m, 2H), 3.11 (sextet, *J* = 6.4 Hz, 1H), 1.71-1.61 (m, 1H), 1.50-1.39 (m, 1H), 1.17 (d, *J* = 6.4 Hz, 3H), 0.98 (t, *J* = 7.2 Hz, 3H); ^{13}C NMR (100 MHz, $CDCl_3$): δ 167.0, 133.3, 129.4, 128.6, 58.2, 26.8, 17.6, 10.3.

***N*-((3s,5s,7s)-adamantan-1-yl)-*O*-benzoylhydroxylamine (2m)**

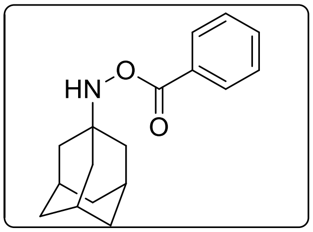

Column chromatography (SiO_2, eluting with 98:2 hexane/ethyl acetate) afforded the desired product as crystalline solid.

^{1}H NMR (400 MHz, $CDCl_3$): δ 8.03-8.01 (m, 2H), 7.59-7.55 (m, 1H), 7.47-7.43 (m, 2H), 2.11 (s, 3H), 1.76 (d, *J* = 2.8 Hz, 6H), 1.71-1.62 (m, 6H); ^{13}C NMR (100 MHz, $CDCl_3$): δ 167.0, 133.3, 129.4, 128.6, 56.4, 40.1, 36.5, 29.2.

***O*-benzoyl-*N*-(1-phenylethyl)hydroxylamine (2n)**

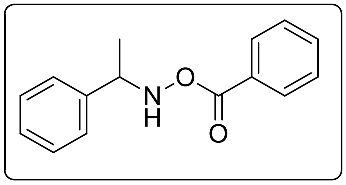

Column chromatography (SiO_2, eluting with 98:2 hexane/ethyl acetate) afforded the desired product as colourless liquid.
^{1}H NMR (400 MHz, $CDCl_3$): δ 7.97-7.94 (m, 2H), 7.56-7.51 (m, 1H), 7.45-7.35 (m, 6H), 7.32-7.28 (m, 1H), 4.34 (q, *J* = 6.8 Hz, 2H), 1.55 (d, *J* = 6.8 Hz, 3H); ^{13}C NMR (100 MHz, $CDCl_3$): δ 166.9, 141.3, 133.4, 129.4, 128.7, 128.6, 128.5, 127.9, 127.2, 61.0, 19.8.

3-cyclohexyl-1-(pyridin-2-yl)-1H-naphtho[1,2-d]imidazol-2(3H)-one (3a)

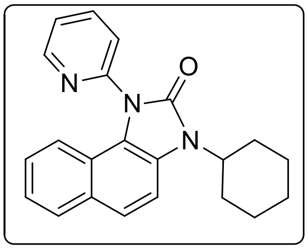

The general procedure 1 for imidazolone was followed. Column chromatography (SiO_2, eluting with 75:25 hexane/ethyl acetate) afforded the desired product as a light brown solid (63.8 mg, 93% yield), mp 142-144 °C.

^{1}H NMR (400 MHz, $CDCl_3$): δ 8.67-8.65 (m, 1H), 7.97 (td, J_1 = 8.0 Hz, J_2 = 2.0 Hz, 1H), 7.82 (d, *J* = 8.0 Hz, 1H), 7.70 (dt, J_1 = 8.0 Hz, J_2 = 0.8 Hz, 1H), 7.64 (d, *J* = 8.8 Hz, 1H), 7.51 (d, *J* = 8.8 Hz, 1H), 7.45-7.41 (m, 1H), 7.29-7.25 (m, 1H), 7.19-7.15 (m, 1H), 6.96 (d, *J* = 8.0 Hz, 1H), 4.42-4.34 (m, 1H), 2.33-2.22 (m, 2H), 1.99-1.92 (m, 4H), 1.77 (d, *J* = 12.8 Hz, 1H),1.53-1.42 (m, 2H), 1.36-1.29 (m, 1H); ^{13}C NMR (100 MHz, $CDCl_3$): δ 153.7, 150.2, 149.6, 138.8, 129.9, 129.2, 126.0, 125.8, 123.7, 123.5, 123.2, 121.8, 121.3, 120.4, 110.4, 53.7, 30.4, 26.2, 25.5; HRMS (ESI, m/z) calcd. For $C_{22}H_{22}N_3O$ $[M+H]^+$: 344.1763; found: 344.1760.

3-cyclopentyl-1-(pyridin-2-yl)-1H-naphtho[1,2-d]imidazol-2(3H)-one (3b)

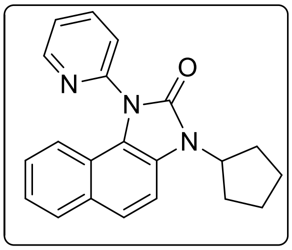

The general procedure 1 for imidazolone was followed. Column chromatography (SiO_2, eluting with 75:25 hexane/ethyl acetate) afforded the desired product as a light brown solid (51 mg, 78% yield), mp 138-140 °C.

^{1}H NMR (400 MHz, $CDCl_3$): δ 8.67-8.65 (m, 1H), 7.97 (td, J_1 = 8.0 Hz, J_2 = 2.0 Hz, 1H), 7.82 (dd, J_1 = 8.4 Hz, J_2 = 0.4 Hz, 1H), 7.69 (dt, J_1 = 8.0 Hz, J_2 = 0.8 Hz, 1H), 7.65 (d, J = 8.8 Hz, 1H), 7.45-7.40 (m, 2H), 7.29-7.24 (m, 1H), 7.19-7.15 (m, 1H), 6.97 (d, J = 8.8 Hz, 1H), 4.97 (quintet, J = 8.8 Hz, 1H), 2.29-2.20 (m, 2H), 2.14-1.97 (m, 4H), 1.80-1.73 (m, 2H); ^{13}C NMR(100 MHz, $CDCl_3$):δ 153.9, 150.2, 149.6, 138.8, 130.0, 129.2, 125.8, 125.6, 123.8, 123.6, 123.5, 123.3, 121.9, 121.2, 120.5, 110.1, 54.0, 29.3, 25.3; HRMS (ESI, m/z) calcd. For $C_{21}H_{20}N_3O$ $[M+H]^+$: 330.1606; found: 330.1644.

3-cycloheptyl-1-(pyridin-2-yl)-1H-naphtho[1,2-d]imidazol-2(3H)-one (3c)

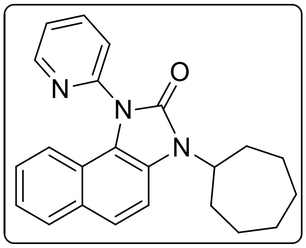

The general procedure 1 for imidazolone was followed. Column chromatography (SiO_2, eluting with 75:25 hexane/ethyl acetate) afforded the desired product as a light brown solid (46.4 mg, 65% yield), mp 154-156 °C.

^{1}H NMR (400 MHz, $CDCl_3$): δ 8.67-8.65 (m, 1H), 7.98 (td, J_1 = 8.0 Hz, J_2 = 2.0 Hz, 1H), 7.82 (d, J = 8.4 Hz, 1H), 7.70 (dt, J_1 = 8.0 Hz, J_2 = 0.8 Hz, 1H), 7.64 (d, J = 8.8 Hz, 1H), 7.47-7.42 (m, 2H), 7.29-7.25 (m, 1H), 7.19-7.15 (m, 1H), 6.97 (d, J = 8.8 Hz, 1H), 4.62-4.55 (m, 1H), 2.37-2.29 (m, 2H), 2.07-2.03 (m, 2H), 1.88-1.85 (m, 2H), 1.73-1.64 (m, 6H); ^{13}C NMR (100 MHz, $CDCl_3$): δ 153.5, 150. 2, 149.5, 138.8, 130.0, 129.2, 125.8, 125.7, 123.7, 123.49, 123.47, 123.3, 121.8, 121.3, 120.5, 110.5, 55.6, 32.9, 27.7, 25.9; HRMS (ESI, m/z) calcd. For $C_{23}H_{24}N_3O$ $[M+H]^+$: 358.1919; found: 358.1921.

3-cyclooctyl-1-(pyridin-2-yl)-1H-naphtho[1,2-d]imidazol-2(3H)-one (3d)

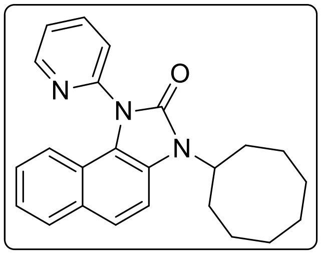

The general procedure 1 for imidazolone was followed. Column chromatography (SiO_2, eluting with 75:25 hexane/ethyl acetate) afforded the desired product as a light brown solid (34 mg, 46% yield), mp 156-158 °C.

^{1}H NMR (400 MHz, $CDCl_3$): δ 8.66-8.64 (m, 1H), 7.97 (td, J_1 = 8.0 Hz, J_2 = 2.0 Hz, 1H), 7.82 (d, J = 8.0 Hz, 1H), 7.71 (dt, J_1 = 8.0 Hz, J_2 = 0.8 Hz, 1H), 7.64 (d, J = 8.8 Hz, 1H), 7.45-7.41 (m, 2H), 7.29-7.25 (m, 1H), 7.19-7.15 (m, 1H), 6.99 (d, J = 8.8 Hz, 1H), 4.75-4.69 (m, 1H), 2.39-2.31 (m, 2H), 2.02-1.97 (m, 2H), 1.89-1.84 (m, 2H), 1.79-1.60 (m, 8H); ^{13}C NMR (100 MHz, $CDCl_3$): δ 153.5, 150.2, 149.5, 138.7, 130.0, 129.2, 125.8, 125.5, 123.6, 123.5, 123.4, 123.3, 121.9, 121.3, 120.5, 110.6, 54.1, 32.6, 26.4, 26.2, 25.5; HRMS (ESI, m/z) calcd. For $C_{24}H_{26}N_3O$ $[M+H]^+$: 372.2076; found: 372.2076.

3-ethyl-1-(pyridin-2-yl)-1H-naphtho[1,2-d]imidazol-2(3H)-one (3e)

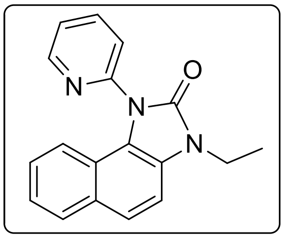

The general procedure 1 for imidazolone was followed. Column chromatography (SiO_2, eluting with 75:25 hexane/ethyl acetate) afforded the desired product as a light brown solid (29 mg, 50% yield), mp 96-98 °C.

^{1}H NMR (400 MHz, $CDCl_3$): δ 8.68-8.66 (m, 1H), 7.98 (td, J_1 = 8.0 Hz, J_2 = 2.0 Hz, 1H), 7.84 (d, J = 8.0 Hz, 1H), 7.72-7.68 (m, 2H), 7.46-7.42 (m, 1H), 7.34 (d, J = 8.4 Hz, 1H), 7.31-7.27 (m, 1H), 7.22-7.18 (m, 1H), 7.02 (d, J = 9.2 Hz, 1H), 4.09 (q, J = 7.2 Hz, 2H), 1.43 (t, J = 7.2 Hz, 3H); ^{13}C NMR (100 MHz, $CDCl_3$): δ 153.9, 150.1, 149.6, 138.9, 130.4, 129.3, 126.2, 125.9, 123.8, 123.7, 123.5, 123.4, 121.8, 121.2, 120.5, 108.9, 36.3, 13.9; HRMS (ESI, m/z) calcd. For $C_{18}H_{16}N_3O$ $[M+H]^+$: 290.1293; found: 290.1296.

3-propyl-1-(pyridin-2-yl)-1H-naphtho[1,2-d]imidazol-2(3H)-one (3f)

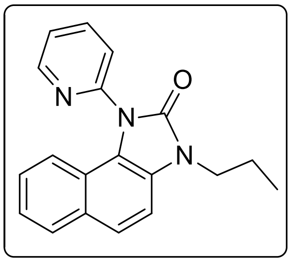

The general procedure 1 for imidazolone was followed. Column chromatography (SiO_2, eluting with 75:25 hexane/ethyl acetate) afforded the desired product as a light brown solid (39.4 mg, 65% yield), mp 134-136 °C.

^{1}H NMR (400 MHz, $CDCl_3$): δ 8.66 (d, *J* = 3.6 Hz, 1H), 7.97 (t, *J* = 7.6 Hz, 1H), 7.82 (d, *J* = 8.4 Hz, 1H), 7.71-7.66 (m, 2H), 7.45-7.41 (m, 1H), 7.33 (d, *J* = 8.4 Hz, 1H), 7.27 (t, *J* = 7.2 Hz, 1H), 7.19 (t, *J* = 8.0 Hz, 1H), 7.01 (d, *J* = 8.8 Hz, 1H), 3.98 (t, *J* = 7.2 Hz, 2H), 1.87 (sextet, *J* = 7.2 Hz, 2H), 1.02 (t, *J* = 7.2 Hz, 3H); ^{13}C NMR (100 MHz, $CDCl_3$):δ 154.2, 150.1, 149.6, 138.8, 130.4, 129.3, 126.6, 125.9, 123.8, 123.7, 123.5, 123.4, 121.7, 121.3, 120.5, 109.1, 43.1, 22.1, 11.5; HRMS (ESI, m/z) calcd. For $C_{19}H_{18}N_3O$ $[M+H]^+$: 304.1450; found: 304.1451.

3-butyl-1-(pyridin-2-yl)-1H-naphtho[1,2-d]imidazol-2(3H)-one (3g)

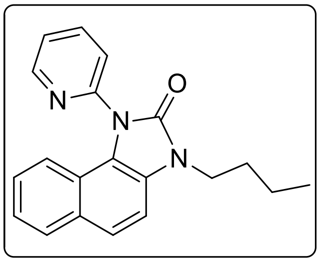

The general procedure 1 for imidazolone was followed. Column chromatography (SiO_2, eluting with 75:25 hexane/ethyl acetate) afforded the desired product as a creamy white solid (52 mg, 82% yield), mp 104-106 °C.

^{1}H NMR (400 MHz, $CDCl_3$): δ 8.68-8.67 (m, 1H), 7.99 (td, J_1 = 8.0 Hz, J_2 = 2.0 Hz, 1H), 7.85-7.83 (m, 1H), 7.72 (dt, J_1 = 8.0 Hz, J_2 = 0.8 Hz, 1H), 7.69 (d, *J* = 8.8 Hz, 1H), 7.47-7.43 (m, 1H), 7.34 (d, *J* = 8.8 Hz, 1H), 7.31-7.27 (m, 1H), 7.22-7.18 (m, 1H), 7.04-7.02 (m, 1H), 4.03 (t, *J* = 7.2 Hz, 2H), 1.86-1.79 (m, 2H), 1.51-1.41 (m, 2H), 0.97 (t, *J* = 7.6 Hz, 3H); ^{13}C NMR (100 MHz, $CDCl_3$): δ 154.2, 150.1, 149.5, 138.9, 130.4, 129.3, 126.6, 125.9, 123.8, 123.7, 123.5, 123.4, 121.7, 121.3, 120.5, 109.1, 41.3, 30.8, 20.2, 13.8; HRMS (ESI, m/z) calcd. For $C_{20}H_{20}N_3O$ $[M+H]^+$: 318.1606; found: 318.1657.

3-isobutyl-1-(pyridin-2-yl)-1H-naphtho[1,2-d]imidazol-2(3H)-one (3h)

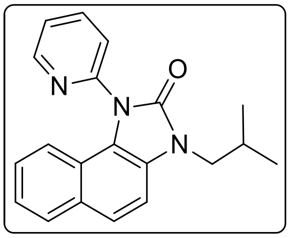

The general procedure 1 for imidazolone was followed. Column chromatography (SiO_2, eluting with 75:25 hexane/ethyl acetate) afforded the desired product as a light brown solid (40 mg, 63% yield). mp 112-114 °C.

^{1}H NMR (400 MHz, $CDCl_3$): δ 8.67 (s, 1H), 7.98 (td, J_1 = 7.6 Hz, J_2 = 1.6 Hz, 1H), 7.83 (d, J = 7.6 Hz, 1H), 7.72 (d, J = 8.0 Hz, 1H), 7.67 (d, J = 8.8 Hz, 1H), 7.46-7.43 (m, 1H), 7.34-7.26 (m, 2H), 7.22-7.18 (m, 1H), 7.03 (d, J = 8.4 Hz, 1H), 3.82 (d, J = 7.6 Hz, 2H), 2.34-2.24 (m, 1H), 1.02 (d, J = 6.8 Hz, 6H); ^{13}C NMR (100 MHz, $CDCl_3$): δ 154.5, 150.1, 149.4, 138.8, 130.4, 129.3, 126.9, 125.9, 123.7, 123.5, 123.4, 121.6, 121.3, 120.5, 109.4, 48.9, 28.3, 20.3; HRMS (ESI, m/z) calcd. For $C_{20}H_{20}N_3O$ [M+H]$^+$: 318.1606; found: 318.1608.

3-(sec-butyl)-1-(pyridin-2-yl)-1H-naphtho[1,2-d]imidazol-2(3H)-one (3i)

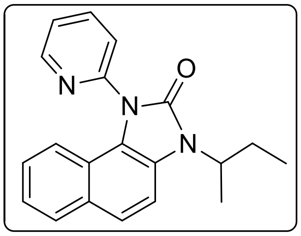

The general procedure 1 for imidazolone was followed. Column chromatography (SiO_2, eluting with 75:25 hexane/ethyl acetate) afforded the desired product as light brown solid (60 mg, 95% yield), mp 112-114 °C.

^{1}H NMR (400 MHz, $CDCl_3$):δ 8.68-8.66 (m, 1H), 7.98 (td, J_1 = 7.6 Hz, J_2 = 2.0 Hz, 1H), 7.82 (d, J = 8.0 Hz, 1H), 7.71 (dt, J_1 = 8.0 Hz, J_2 = 0.8 Hz, 1H), 7.65 (d, J = 8.4 Hz, 1H), 7.46-7.42 (m, 2H), 7.30-7.26 (m, 1H), 7.20-7.16 (m, 1H), 6.99 (dd, J_1 = 8.8 Hz, J_2 = 0.8 Hz, 1H), 4.61-4.51 (m, 1H), 2.24-2.13 (m, 1H), 1.98-1.87 (m, 1H), 1.61 (d, J = 6.8 Hz, 3H), 0.93 (t, J = 7.6 Hz, 3H),^{13}C NMR (100 MHz, $CDCl_3$): δ 153.9, 150.1, 149.5, 138.8, 130.0, 129.2, 126.0, 125.8, 123.7, 123.6, 123.5, 123.4, 121.8, 121.3, 120.5, 110.2, 51.8, 27.6, 18.8, 11.5; HRMS (ESI, m/z) calcd. For $C_{20}H_{20}N_3O$ [M+H]$^+$: 318.1606; found: 318.1610.

3-(tert-butyl)-1-(pyridin-2-yl)-1H-naphtho[1,2-d]imidazol-2(3H)-one (3j)

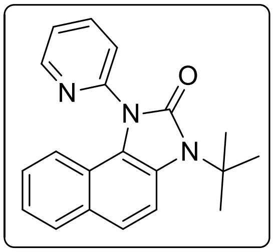

The general procedure 1 for imidazolone was followed. Column chromatography (SiO_2, eluting with 75:25 hexane/ethyl acetate) afforded the desired product as a creamy white powder (59.6 mg, 94% yield), mp 154−156 °C.

^{1}H NMR (400 MHz, $CDCl_3$): δ 8.67 (dd, J_1 = 4.8 Hz, J_2 = 1.2 Hz, 1H), 7.96 (td, J_1 = 8.0 Hz, J_2 = 2.0 Hz, 1H), 7.79 (d, J = 8.0 Hz, 1H), 7.75 (d, J = 9.2 Hz, 1H), 7.66 (d, J = 8.0 Hz, 1H), 7.58 (d, J = 9.2 Hz, 1H), 7.45- 7.42 (m, 1H), 7.28-7.24 (m, 1H), 7.15-7.11 (m, 1H), 6.85 (d, J = 8.8 Hz, 1H), 1.88 (s, 9H); ^{13}C NMR (100 MHz, $CDCl_3$): δ 154.1, 150.4, 149.7, 138.8, 129.7, 128.9, 126.6, 125.7, 124.0, 123.8, 123.7, 122.6, 122.4, 121.3, 120.0, 113.1, 58.8, 29.8; HRMS (ESI, m/z) calcd. For $C_{20}H_{20}N_3O$ $[M+H]^+$: 318.1606; found: 318.1606.

1-(pyridin-2-yl)-3-(2,4,4-trimethylpentan-2-yl)-1H-naphtho[1,2-d]imidazol-2(3H)-one (3k)

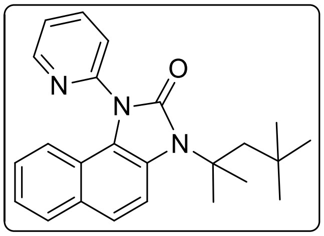

The general procedure 1 for imidazolone was followed. Column chromatography (SiO_2, eluting with 75:25 hexane/ethyl acetate) afforded the desired product as a creamy white solid (49 mg, 66% yield), mp 120−122 °C.

^{1}H NMR (400 MHz, $CDCl_3$): δ 8.68-8.66 (m, 1H), 7.95 (td, J_1 = 8.0 Hz, J_2 = 2.0 Hz, 1H), 7.81-7.78 (m, 2H), 7.62 (dt, J_1 = 8.0 Hz, J_2 = 0.4 Hz, 1H), 7.59 (d, J = 8.8 Hz, 1H), 7.45-7.41 (m, 1H), 7.29-7.25 (m, 1H), 7.16-7.12 (m, 1H), 6.86 (d, J = 8.8 Hz, 1H), 2.18 (s, 2H), 1.98 (s, 6H), 0.92 (s, 9H); ^{13}C NMR (100 MHz, $CDCl_3$): δ 154.4, 150.5, 149.8, 138.8, 129.6, 128.8, 127.1, 125.6, 124.0, 123.8, 123.7, 122.51, 122.49, 121.4, 120.0, 113.5, 62.4, 51.1, 31.9, 31.2, 31.0; HRMS (ESI, m/z) calcd. For $C_{24}H_{28}N_3O$ $[M+H]^+$: 374.2232; found: 374.2235.

3-((3s)-adamantan-1-ylmethyl)-1-(pyridin-2-yl)-1H-naphtho[1,2-d]imidazol-2(3H)-one (3l)

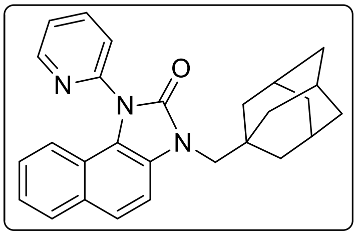

The general procedure 1 for imidazolone was followed. Column chromatography (SiO_2, eluting with 80:20 hexane/ethyl acetate) afforded the desired product as a light brown solid (59 mg, 72% yield), mp 198−200 °C.

^{1}H NMR (400 MHz, $CDCl_3$): δ 8.68 (d, *J* = 3.6 Hz, 1H), 7.98 (td, J_1 = 8.0 Hz, J_2 = 2.0 Hz, 1H), 7.84-7.82 (m, 1H), 7.73 (d, *J* = 8.0 Hz, 1H), 7.66 (d, *J* = 8.4 Hz, 1H), 7.46-7.43 (m, 1H), 7.38 (d, *J* = 8.8 Hz, 1H), 7.31-7.26 (m, 1H), 7.22-7.18 (m, 1H), 7.05-7.02 (m, 1H), 3.69 (s, 2H), 1.98 (s, 3H), 1.71-1.60 (m, 12H); ^{13}C NMR (100 MHz, $CDCl_3$): δ 155.2, 150.1, 149.4, 138.9, 130.3, 129.3, 128.2, 125.9, 123.67, 123.66, 123.4, 121.6, 121.3, 120.3, 110.3, 53.9, 41.2, 36.8, 36.4, 28.3; HRMS (ESI, m/z) calcd. For $C_{27}H_{28}N_3O$ $[M+H]^+$: 410.2232; found: 410.2231.

3-((3s,5s,7s)-adamantan-1-yl)-1-(pyridin-2-yl)-1H-naphtho[1,2-d]imidazol-2(3H)-one (3m)

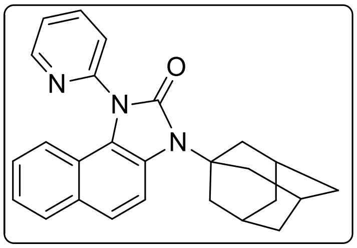

The general procedure 1 for imidazolone was followed. Column chromatography (SiO_2, eluting with 80:20 hexane/ethyl acetate) afforded the desired product as a light brown solid (77 mg, 98% yield), mp 164-166 °C.

^{1}H NMR (400 MHz, $CDCl_3$): δ 8.67-8.65 (m, 1H), 7.98-7.93 (m, 1H), 7.85 (dd, J_1 = 8.8 Hz, J_2 = 1.2 Hz, 1H), 7.78 (d, *J* = 8.0 Hz, 1H), 7.66-7.63 (m, 1H), 7.54 (d, *J* = 9.2 Hz, 1H), 7.44-7.41 (m, 1H), 7.28-7.23 (m, 1H), 7.14-7.10 (m, 1H), 6.85 (d, *J* = 9.2 Hz, 1H), 2.68 (s, 6H), 2.25 (s, 3H), 1.80 (dd, J_1 = 32.4 Hz, J_2 = 12.0 Hz, 6H); ^{13}C NMR (100 MHz, $CDCl_3$): δ 154.0, 150.5, 149.7, 138.7, 129.5, 128.8, 126.4, 125.6, 124.0, 123.72, 123.67, 122.6, 122.2, 121.3, 120.0, 113.6, 61.1, 40.9, 36.3, 30.1; HRMS (ESI, m/z) calcd. For $C_{26}H_{26}N_3O$ $[M+H]^+$: 396.2076; found: 396.2076

3-(1-phenylethyl)-1-(pyridin-2-yl)-1H-naphtho[1,2-d]imidazol-2(3H)-one (3n)

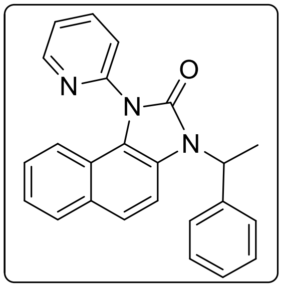

The general procedure 1 for imidazolone was followed. Column chromatography (SiO_2, eluting with 75:25 hexane/ethyl acetate) afforded the desired product as a pale yellow solid (48.2 mg, 66% yield), mp 136-138 °C.

^{1}H NMR (400 MHz, $CDCl_3$): δ 8.70-8.69 (m, 1H), 8.01 (td, J_1 = 8.0 Hz, J_2 = 2.0 Hz, 1H), 7.78-7.75 (m, 2H), 7.49-7.44 (m, 4H), 7.36-7.32 (m, 2H), 7.29-7.24 (m, 2H), 7.19-7.16 (m, 1H), 7.02-6.98 (m, 2H), 6.01 (q, J = 7.2 Hz, 1H), 1.99 (d, J = 7.2 Hz, 3H); ^{13}C NMR (100 MHz, $CDCl_3$): δ 154.2, 150.1, 149.6, 139.8, 138.9, 130.1, 129.2, 128.8, 127.7, 126.9, 125.8, 125.4, 123.8, 123.7, 123.5, 123.4, 121.9, 121.2, 120.4, 110.8, 51.2, 17.7; HRMS (ESI, m/z) calcd. For $C_{24}H_{20}N_3O$ $[M+H]^+$: 366.1606; found: 366.1606.

3-(1-(naphthalen-1-yl)ethyl)-1-(pyridin-2-yl)-1H-naphtho[1,2-d]imidazol-2(3H)-one (3o)

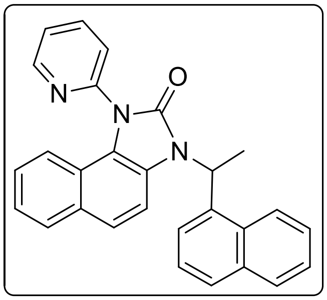

The general procedure 1 for imidazolone was followed. Column chromatography (SiO_2, eluting with 75:25 hexane/ethyl acetate) afforded the desired product as a light brown solid (73 mg, 88% yield), mp 222-224 °C.

^{1}H NMR (400 MHz, $CDCl_3$): δ 8.69 (d, J = 4.8 Hz, 1H), 8.25 (d, J = 8.4 Hz, 1H), 8.01 (td, J_1 = 7.6 Hz, J_2 = 1.6 Hz, 1H), 7.96 (d, J = 7.2 Hz, 1H), 7.86 (d, J = 8.4 Hz, 1H), 7.81 (d, J = 8.0 Hz, 1H), 7.77 (d, J = 8.0 Hz, 1H), 7.68 (d, J = 8.4 Hz, 1H), 7.58 (t, J = 8.0 Hz, 1H), 7.51- 7.40 (m, 3H), 7.36 (d, J = 8.8 Hz, 1H), 7.22-7.18 (m, 1H), 7.14-7.10 (m, 2H), 6.93 (d, J = 8.8 Hz, 1H), 6.63 (q, J = 6.8 Hz, 1H), 2.11 (d, J = 6.8 Hz, 3H); ^{13}C NMR (100 MHz, $CDCl_3$): δ 153.9, 150.1, 149.7, 138.9, 134.9, 134.0, 131.8, 129.9, 129.4, 129.1, 128.9, 127.1, 126.1, 125.7, 125.6, 125.0, 124.8, 123.8, 123.59, 123.57, 123.51, 123.3, 122.0, 121.2, 120.3, 110.6, 48.9, 18.1; HRMS (ESI, m/z) calcd. For $C_{28}H_{22}N_3O$ $[M+H]^+$: 416.1763; found: 416.1766.

tert-butyl 4-(2-oxo-1-(pyridin-2-yl)-1H-naphtho[1,2-d]imidazol-3(2H)-yl)piperidine-1-carboxylate (3p)

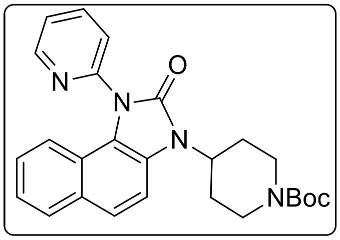

The general procedure 1 for imidazolone was followed. Column chromatography (SiO_2, eluting with 70:30 hexane/ethyl acetate) afforded the desired product as a creamy white solid (80 mg, 90% yield), mp 174-176 °C.

^{1}H NMR (400 MHz, $CDCl_3$): δ 8.68-8.66 (m, 1H), 7.99 (td, J_1 = 8.0 Hz, J_2 = 2.0 Hz, 1H), 7.83 (d, J = 8.0 Hz, 1H), 7.69 (dt, J_1 = 8.0 Hz, J_2 = 0.8Hz, 1H), 7.65 (d, J = 8.8 Hz, 1H), 7.48-7.45 (m, 2H), 7.31-7.27 (m, 1H), 7.20-7.16 (m, 1H), 6.94 (d, J = 8.8 Hz, 1H), 4.62-4,54 (m, 1H), 4.35-4.33 (br. S, 2H), 2.90 (t, J = 12.4 Hz, 2H), 2.50-2.40 (m, 2H), 1.92 (d, J = 12.4 Hz, 2H), 1.50 (s, 9H); ^{13}C NMR (100 MHz, $CDCl_3$): δ 154.8, 153.6, 150.0, 149.7, 138.9, 130.1, 129.2, 125.9, 125.4, 123.9, 123.8, 123.52, 123.50, 121.9, 121.2, 120.4, 110.2, 80.0, 51.8, 29.5, 28.6; HRMS (ESI, m/z) calcd. For $C_{26}H_{29}N_4O_3$ $[M+H]^+$: 445.2240; found: 445.2237.

3-cyclohexyl-5-phenyl-1-(pyridin-2-yl)-1H-naphtho[1,2-d]imidazol-2(3H)-one (3q)

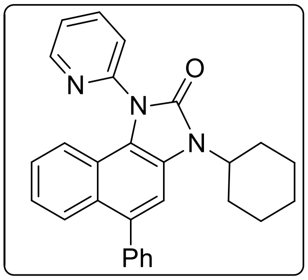

The general procedure 1 for imidazolone was followed. Column chromatography (SiO_2, eluting with 75:25 hexane/ethyl acetate) afforded the desired product as a creamy white solid (67.8 mg, 81% yield), mp 186-188 °C.

^{1}H NMR (400 MHz, $CDCl_3$): δ 8.68-8.66 (m, 1H), 7.99 (td, J_1 = 7.6 Hz, J_2 = 2.0 Hz, 1H), 7.83-7.80 (m, 1H), 7.74 (dt, J_1 = 8.0 Hz, J_2 = 0.8 Hz, 1H), 7.53-7.48 (m, 4H), 7.47-7.42 (m, 3H), 7.23-7.16 (m, 2H), 7.05-7.03 (m, 1H), 4.41-4.33 (m, 1H), 2.33-2.23 (m, 2H), 1.99-1.90 (m, 4H), 1.73 (d, J = 13.2 Hz, 1H), 1.50-1.40 (m, 2H), 1.32-1.26 (m, 1H); ^{13}C NMR (100 MHz, $CDCl_3$): δ 153.9, 150.3, 149.6, 141.1, 138.8, 136.0, 130.5, 128.4, 128.2, 127.46, 127.44, 125.71, 125.67, 123.63,

123.61, 123.4, 121.51, 121.46, 120.6, 111.4, 53.8, 30.4, 26.1, 25.4; HRMS (ESI, m/z) calcd. For $C_{28}H_{26}N_3O$ $[M+H]^+$: 420.2076; found: 420.2075.

5-bromo-3-cyclohexyl-1-(pyridin-2-yl)-1H-naphtho[1,2-d]imidazol-2(3H)-one (3r)

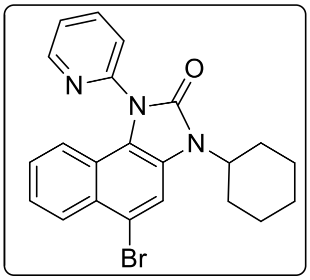

The general procedure 1 for imidazolone was followed. Column chromatography (SiO_2, eluting with 75:25 hexane/ethyl acetate) afforded the desired product as a creamy white solid (68 mg, 81% yield), mp 168-170 °C.

^{1}H NMR (400 MHz, $CDCl_3$): δ 8.65-8.63 (m, 1H), 8.25-8.22 (m, 1H), 7.98 (td, J_1 = 7.6 Hz, J_2 = 2.0 Hz, 1H), 7.83 (s, 1H), 7.69 (dt, J_1 = 8.0 Hz, J_2 = 0.8 Hz, 1H), 7.46-7.43 (m, 1H), 7.40-7.35 (m, 1H), 7.40-7.35(m, 1H), 7.22-7.18 (m, 1H), 6.99-6.96 (m, 1H), 4.36-4.28 (m, 1H), 2.29-2.19 (m, 2H), 1.95 (d, J = 11.2 Hz, 4H), 1.77 (d, J = 12.8 Hz, 1H),1.52-1.42 (m, 2H), 1.38-1.30 (m, 1H);^{13}C NMR (100 MHz, $CDCl_3$): δ 153.6, 150.0, 149.7, 138.9, 128.5, 127.8, 126.5, 126.2, 124.9, 123.9, 123.5, 121.9, 121.7, 121.2, 116.7, 114.4, 53.9, 30.4, 26.1, 25.4; HRMS (ESI, m/z) calcd. For $C_{22}H_{21}BrN_3O$ $[M+H]^+$: 422.0868; found: 422.0886.

3-cyclohexyl-5-methoxy-1-(pyridin-2-yl)-1H-naphtho[1,2-d]imidazol-2(3H)-one (3s)

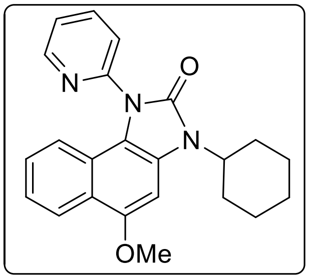

The general procedure 1 for imidazolone was followed. Column chromatography (SiO_2, eluting with 70:30 hexane/ethyl acetate) afforded the desired product as a brown solid (44.7 mg, 60% yield), mp 166-168 °C.

^{1}H NMR (400 MHz, $CDCl_3$): δ 8.64-8.62 (m, 1H), 8.27-8.25 (m, 1H), 7.95 (td, J_1 = 8.0 Hz, J_2 = 2.0 Hz, 1H), 7.70 (dt, J_1 = 8.0 Hz, J_2 = 0.8 Hz, 1H), 7.41-7.37 (m, 1H), 7.29-7.25 (m, 1H), 7.22-7.18 (m, 1H), 6.98-6.96 (m, 1H), 6.87 (s, 1H), 4.35-4.27 (m, 1H), 4.05 (s, 3H), 2.34-2.24 (m, 2H), 1.99-1.93 (m, 4H), 1.78 (d, J = 12.8 Hz, 1H), 1.54-1.43 (m, 2H), 1.37-1.28 (m, 1H); ^{13}C NMR (100 MHz, $CDCl_3$):δ 153.9, 152.6, 150.3, 149.4, 138.6, 126.5, 126.1, 123.3, 123.21, 123.18, 122.8,

121.8, 121.3, 121.0, 115.4, 90.8, 56.3, 53.8, 30.4, 26.2, 25.6; HRMS (ESI, m/z) calcd. For $C_{23}H_{24}N_3O_2$ $[M+H]^+$: 374.1869; found: 374.1874.

3-cyclohexyl-5-(furan-3-yl)-1-(pyridin-2-yl)-1H-naphtho[1,2-d]imidazol-2(3H)-one (3t)

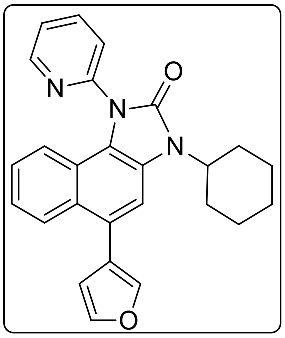

The general procedure 1 for imidazolone was followed. Column chromatography (SiO_2, eluting with 75:25 hexane/ethyl acetate) afforded the desired product as a light brown solid (65.4 mg, 80% yield), mp 190-192 °C.

^{1}H NMR (400 MHz, $CDCl_3$): δ 8.67-8.66 (m, 1H), 8.07-8.05 (m, 1H), 7.99 (td, J_1 = 8.0 Hz, J_2 = 2.0 Hz, 1H), 7.72 (dt, J_1 = 8.0 Hz, J_2 = 0.8 Hz, 1H), 7.65 (dd, J_1 = 1.6 Hz, J_2 = 0.8 Hz, 1H), 7.59 (t, J = 1.6 Hz, 1H), 7.47-7.43 (m, 2H), 7.29-7.25 (m, 1H), 7.21-7.17 (m, 1H), 7.04-7.01 (m, 1H), 6.68 (dd, J_1 = 2.0 Hz, J_2 = 0.8 Hz, 1H), 4.40-4.32 (m, 1H), 2.33-2.22 (m, 2H), 1.99-1.91 (m, 4H), 1.76 (d, J = 12.8 Hz, 1H), 1.53-1.42 (m, 2H), 1.36-1.28 (m, 1H); ^{13}C NMR (100 MHz, $CDCl_3$): δ 153.8, 150.2, 149.5, 143.0, 140.6, 138.9, 128.5, 127.0, 126.3, 125.8, 125.7, 123.8, 123.7, 123.4, 121.7, 121.6, 120.7, 113.0, 111.4, 53.8, 30.4, 26.1, 25.4; HRMS (ESI, m/z) calcd. For $C_{26}H_{24}N_3O$ $[M+H]^+$: 410.1869; found: 410.1870.

3-cyclohexyl-5-(1H-pyrazol-1-yl)-1-(pyridin-2-yl)-1H-naphtho[1,2-d]imidazol-2(3H)-one (3u)

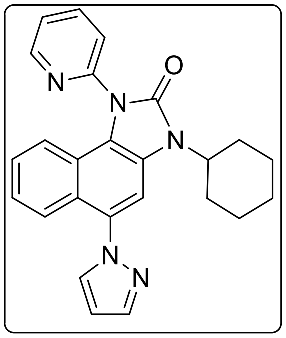

The general procedure 1 for imidazolone was followed. Column chromatography (SiO_2, eluting with 70:30 hexane/ethyl acetate) afforded the desired product as a creamy white solid (79.3 mg, 97% yield), mp 192-194 °C.

^{1}H NMR (400 MHz, $CDCl_3$): δ 8.68-8.66 (m, 1H), 8.01 (td, J_1 = 7.6 Hz, J_2 = 2.0 Hz, 1H), 7.85-7.84 (m, 1H), 7.76 (dd, J_1 = 2.4 Hz, J_2 = 0.8 Hz, 1H), 7.73 (dt, J_1 = 8.0 Hz, J_2 = 0.8 Hz, 1H), 7.62 (s, 1H), 7.54-7.51 (m, 1H), 7.48-7.45 (m, 1H), 7.30-7.25 (m, 1H), 7.23-7.19 (m, 1H), 7.05-7.02 (m, 1H), 6.56 (t, J = 2.0 Hz, 1H), 4.37-4.29 (m, 1H), 2.31-2.21 (m, 2H), 1.97-1.89 (m, 4H), 1.73 (d, J = 12.8 Hz, 1H), 1.49-1.39 (m, 2H), 1.33-1.25 (m, 1H); ^{13}C NMR (100 MHz, $CDCl_3$): δ 153.8, 149.9, 149.7, 140.9, 139.0, 133.0, 132.6, 126.4, 126.1, 125.2, 124.9, 124.0, 123.96, 123.5, 122.5, 121.6, 120.5, 108.95, 106.7, 54.1, 30.3, 26.1, 25.3; HRMS (EI, m/z) calcd. For $C_{25}H_{24}N_5O$ $[M+H]^+$: 410.1981; found: 410.1985.

3-cyclohexyl-5-fluoro-1-(pyridin-2-yl)-1H-naphtho[1,2-d]imidazol-2(3H)-one (3v)

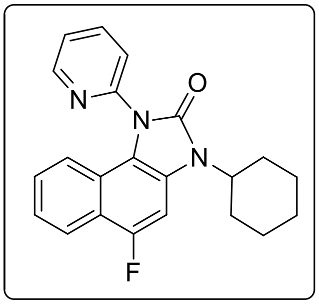

The general procedure 1 for imidazolone was followed. Column chromatography (SiO_2, eluting with 75:25 hexane/ethyl acetate) afforded the desired product as a light brown crystalline solid (47 mg, 65% yield), mp 142-144 °C.

^{1}H NMR (400 MHz, $CDCl_3$): 8.65-8.64 (m, 1H), 8.08 (d, J = 8.0 Hz, 1H), 7.98 (td, J_1 = 7.6 Hz, J_2 = 1.6 Hz, 1H), 7.71 (dt, J_1 = 8.0 Hz, J_2 = 0.8 Hz, 1H), 7.45-7.42 (m, 1H), 7.35- 7.21 (m, 3H), 7.00-6.98 (m, 1H), 4.38-4.29 (m, 1H), 2.27-2.17 (m, 2H), 1.97-1.93 (m, 4H), 1.78 (d, J = 12.8 Hz, 1H), 1.53-1.41 (m, 2H), 1.36-1.29 (m, 1H); ^{13}C NMR (100 MHz, $CDCl_3$): δ 155.5 (d, J = 244.7 Hz), 153.9, 150.0, 149.5, 138.9, 126.8, 125.2 (d, J = 12.5 Hz), 123.7, 123.69, 123.3, 121.7 (d, J = 6.3 Hz), 121.2 (d, J = 2.7 Hz), 120.8 (d, J = 4.8 Hz), 119.5 (d, J = 16.8 Hz), 117.9, 96.1 (d, J = 27.7 Hz), 53.9, 30.3, 26.1, 25.4; ^{19}F NMR (376 MHz, $CDCl_3$): δ -127.04 (s, 1F); HRMS (ESI, m/z) calcd. For $C_{22}H_{21}FN_3O_3$ $[M+H]^+$: 362.1669; found: 362.1667.

3-cyclohexyl-5-methyl-1-(pyridin-2-yl)-1H-naphtho[1,2-d]imidazol-2(3H)-one (3w)

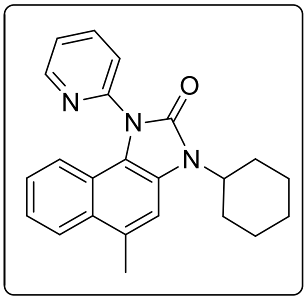

The general procedure 1 for imidazolone was followed. Column chromatography (SiO_2, eluting with 75:25 hexane/ethyl acetate) afforded the desired product as a light brown solid (53.5 mg, 75% yield), mp 134-136 °C.

^{1}H NMR (400 MHz, $CDCl_3$): δ 8.66-8.64 (m, 1H), 7.99-7.94 (m, 2H), 7.69 (dt, J_1 = 8.0 Hz, J_2 = 0.4 Hz, 1H), 7.43-7.40 (m, 1H), 7.37 (s, 1H), 7.34-7.30 (m, 1H), 7.21-7.16 (m, 1H), 7.02 (d, J = 8.4 Hz, 1H), 4.40-4.32 (m, 1H), 2.76 (s, 3H), 2.34-2.24 (m, 2H), 1.98-1.92 (m, 4H), 1.78 (d, J = 12.4 Hz, 1H), 1.53-1.43 (m, 2H), 1.39-1.31 (m, 1H), 1.25 (grease); ^{13}C NMR (100 MHz, $CDCl_3$): δ 153.8, 150.4, 149.5, 138.8, 129.6, 128.8, 125.7, 125.5, 125.3, 123.5, 123.39, 123.36, 121.8, 120.7, 120.5, 111.3, 53.7, 30.4, 26.2, 25.5, 20.3; HRMS (ESI, m/z) calcd. For $C_{23}H_{24}N_3O$ $[M+H]^+$: 358.1919; found: 358.1920.

3-cyclohexyl-9-methyl-1-(pyridin-2-yl)-1H-naphtho[1,2-d]imidazol-2(3H)-one (3x)

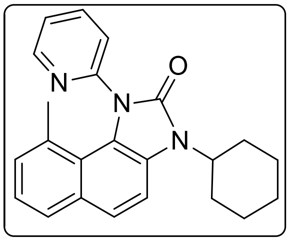

The general procedure 1 for imidazolone was followed. Column chromatography (SiO_2, eluting with 75:25 hexane/ethyl acetate) afforded the desired product as a light brown solid (67.8 mg, 95 % yield), mp 164-166 °C.

^{1}H NMR (400 MHz, $CDCl_3$): δ 8.33-8.31 (m, 1H), 7.87 (td, J_1 = 8.0 Hz, J_2 = 2.0 Hz, 1H), 7.78 (d, J = 8.0 Hz, 1H), 7.69-7.66 (m, 2H), 7.45 (d, J = 8.8 Hz, 1H), 7.25-7.20 (m, 2H), 7.08 (d, J = 7.2 Hz, 1H), 4.38-4.30 (m, 1H), 2.34-2.23 (m, 2H), 1.94 (d, J = 11.2 Hz, 4H), 1.77-1.74 (m, 4H), 1.51-1.41 (m, 2H), 1.36-1.28 (m, 1H); ^{13}C NMR (100 MHz, $CDCl_3$): δ 155.1, 153.3, 148.3, 138.2, 131.1, 131.0, 129.0, 128.5, 127.1, 125.3, 123.5, 123.2, 122.2, 122.1, 121.6, 109.7, 53.9, 30.2, 26.2, 25.5, 21.7; HRMS (ESI, m/z) calcd. For $C_{23}H_{24}N_3O$ $[M+H]^+$: 358.1919; found: 358.1925.

3-cyclohexyl-1-(pyridin-2-yl)-5-tosyl-1H-naphtho[1,2-d]imidazol-2(3H)-one (3y)

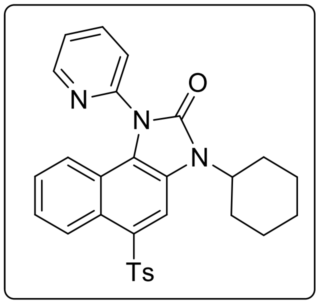

The general procedure 1 for imidazolone was followed. Column chromatography (SiO_2, eluting with 70:30 hexane/ethyl acetate) afforded the desired product as a light brown powder (63.6 mg, 64% yield), mp 172-174 °C.

^{1}H NMR (400 MHz, $CDCl_3$): 8.63-8.62 (m, 1H), 8.60 (s, 1H), 8.58 (dt, J_1 = 8.8 Hz, J_2 = 0.8 Hz, 1H), 8.01 (td, J_1 = 7.6 Hz, J_2 = 1.6 Hz, 1H), 7.81-7.78 (m, 2H), 7.69 (dt, J_1 = 8.0 Hz, J_2 = 0.8 Hz, 1H), 7.49-7.46 (m, 1H), 7.35- 7.31 (m, 1H), 7.25-7.23 (m, 2H), 7.19-7.14 (m, 1H), 7.00-6.97 (m, 1H), 4.45-4.37 (m, 1H), 2.39-2.28 (m, 5H), 2.00-1.96 (m, 4H), 1.79 (d, J = 12.4 Hz, 1H), 1.57-1.46 (m, 2H), 1.42-1.34 (m, 1H); ^{13}C NMR (100 MHz, $CDCl_3$): δ 153.6, 149.8, 149.4, 144.0, 139.2, 130.1, 129.8, 127.3, 127.1, 126.3, 125.8, 125.7, 125.4, 124.4, 123.7, 122.2, 120.6, 114.2, 54.4, 30.4, 25.2, 21.6; HRMS (ESI, m/z) calcd. For $C_{29}H_{28}N_3O_3S$ $[M+H]^+$: 498.1851; found: 498.1853.

3-cyclohexyl-1-(pyridin-2-yl)-1H-imidazo[4,5-f]quinolin-2(3H)-one (3z)

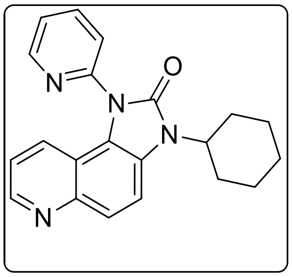

The general procedure 1 for imidazolone was followed. Column chromatography (SiO_2, eluting with 65:35 hexane/ethyl acetate) afforded the desired product as a creamy white powder (62 mg, 90% yield), mp 144-146 °C.

^{1}H NMR (400 MHz, $CDCl_3$): δ 8.73 (d, J = 3.6 Hz, 1H), 8.64-8.62 (m, 1H), 8.01-7.93 (m, 2H), 7.74-7.72 (m, 2H), 7.45-7.41 (m, 2H), 7.11-7.08 (m, 1H), 4.42-4.34 (m, 1H), 2.32-2.21 (m, 2H), 1.97-1.92 (m, 4H), 1.77 (d, J = 12.8 Hz, 1H), 1.52-1.41 (m, 2H), 1.36-1.28 (m, 1H); ^{13}C NMR (100 MHz, $CDCl_3$): δ 153.7, 149.7, 149.5, 148.8, 144.6, 139.0, 129.7, 126.2, 124.6, 123.9, 123.2, 121.1, 120.2, 115.9, 113.5, 53.9, 36.4, 26.1, 25.4; HRMS (ESI, m/z) calcd. For $C_{21}H_{20}N_4ONa$ $[M+Na]^+$: 367.1535; found: 367.1551.

3-cyclohexyl-1-(pyridin-2-yl)-1H-imidazo[4,5-f]isoquinolin-2(3H)-one (3aa)

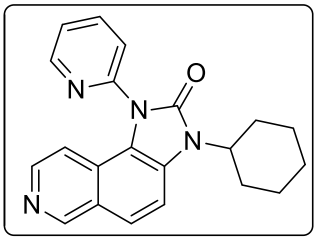

The general procedure 1 for imidazolone was followed. Column chromatography (SiO_2, eluting with 65:35 hexane/ethyl acetate) afforded the desired product as a creamy white powder (56.4 mg, 82% yield), mp 160-162 °C.

^{1}H NMR (400 MHz, $CDCl_3$): δ 9.20 (s, 1H), 8.68-8.66 (m, 1H), 8.20 (d, J = 5.6 Hz, 1H), 8.01 (td, J_1 = 8.0 Hz, J_2 = 2.0 Hz, 1H), 7.82 (d, J = 8.8 Hz, 1H), 7.74 (d, J = 7.6 Hz, 1H), 7.65 (d, J = 8.8 Hz, 1H), 7.49-7.46 (m, 1H), 6.84 (d, J = 6.0 Hz, 1H), 4.44-4.36 (m, 1H), 2.32-2.23 (m, 2H), 1.96 (d, J = 11.6 Hz, 4H), 1.79 (d, J = 12.8 Hz, 1H), 1.53-1.43 (m, 2H), 1.37-1.29 (m, 1H); ^{13}C NMR (100 MHz, $CDCl_3$): δ 153.5, 152.8, 149.5, 149.3, 141.6, 139.0, 129.8, 124.0, 123.8, 123.2, 122.8, 120.5, 114.8, 111.8, 54.1, 30.4, 26.1, 25.4; HRMS (ESI, m/z) calcd. For $C_{21}H_{21}N_4O$ $[M+H]^+$: 345.1715; found: 345.1721.

3-cyclopentyl-5-phenyl-1-(pyridin-2-yl)-1H-naphtho[1,2-d]imidazol-2(3H)-one (3ab)

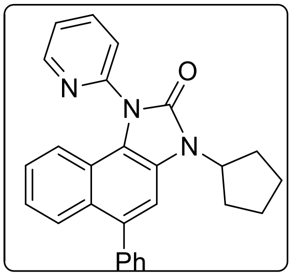

The general procedure 1 for imidazolone was followed. Column chromatography (SiO_2, eluting with 75:25 hexane/ethyl acetate) afforded the desired product as a creamy white powder (45.4 mg, 56% yield), mp 142-144 °C.

^{1}H NMR (400 MHz, $CDCl_3$): δ 8.68-8.67 (m, 1H), 8.01 (td, J_1 = 8.0 Hz, J_2 = 2.0 Hz, 1H), 7.85-7.83 (m, 1H), 7,75 (dt, J_1 = 8.0 Hz, J_2 = 0.8 Hz, 1H), 7.53-7.42 (m, 6H), 7.36 (s, 1H), 7.24-7.16 (m, 2H), 7.07-7.03 (m, 1H), 4.94 (quintet, J = 8.8 Hz, 1H), 2.31-2.22 (m, 2H), 2.13-2.06 (m, 2H), 2.01-1.92 (m, 2H), 1.76-1.70 (m, 2H); ^{13}C NMR: (100 MHz, $CDCl_3$):δ 154.0, 150.2, 149.6, 141.1, 138.9, 136.1, 130.4, 128.4, 128.3, 127.5, 127.4, 125.7, 125.5, 123.7, 123.6, 123.4, 121.5, 120.7, 111.1, 54.1, 29.3, 25.1; HRMS (ESI, m/z) calcd. For $C_{27}H_{24}N_3O$ $[M+H]^+$: 406.1919; found: 406.1917.

3-cyclohexyl-1-(3-methylpyridin-2-yl)-1H-naphtho[1,2-d]imidazol-2(3H)-one (3ac)

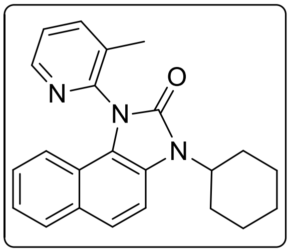

The general procedure 1 for imidazolone was followed. Column chromatography (SiO_2, eluting with 75:25 hexane/ethyl acetate) afforded the desired product as a light brown crystalline solid (54.3 mg, 76% yield), mp 192-194 °C.

^{1}H NMR (400 MHz, $CDCl_3$): δ 8.52 (s, 1H), 7.83-7.80 (m, 2H), 7.62 (d, *J* = 8.8 Hz, 1H), 7.54 (d, *J* = 8.4 Hz, 1H), 7.42 (dd, J_1 = 7.6 Hz, J_2 = 4.8 Hz, 1H), 7.26-7.22 (m, 1H), 7.13-7.09 (m, 1H), 6.63 (d, *J* = 8.8 Hz, 1H), 4.45-4.37 (m, 1H), 2.30-2.23 (m, 5H), 1.99-1.92 (m, 4H), 1.77 (d, *J* = 12.8 Hz, 1H), 1.53-1.44 (m, 2H), 1.37-1.29 (m, 1H); ^{13}C NMR (100 MHz, $CDCl_3$): δ 153.0, 149.3, 147.6, 140.4, 133.4, 129.7, 129.1, 126.1, 125.8, 124.8, 123.5, 122.6, 122.1, 120.3, 119.9, 110.6, 53.6, 30.6, 30.5, 26.1, 25.5, 17.5; HRMS (ESI, m/z) calcd. For $C_{23}H_{24}N_3O$ $[M+H]^+$: 358.1919; found: 358.1921.

3-cyclohexyl-1-(5-methylpyrazin-2-yl)-1H-naphtho[1,2-d]imidazol-2(3H)-one (3ad)

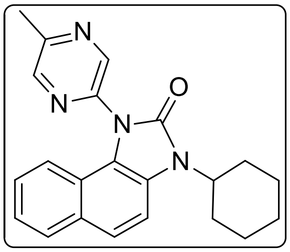

The general procedure 1 for imidazolone was followed. Column chromatography (SiO_2, eluting with 70:30 hexane/ethyl acetate) afforded the desired product as a light brown solid (40.8 mg, 57% yield), mp 134-136 °C.

^{1}H NMR (400 MHz, $CDCl_3$): 8.89 (d, *J* = 0.8 Hz, 1H), 8.50 (s, 1H), 7.84 (d, *J* = 8.0 Hz, 1H), 7.67 (d, *J* = 8.8 Hz, 1H), 7.52 (d, *J* = 8.8 Hz, 1H), 7.32-7.28 (m, 1H), 7.24-7.19 (m, 1H), 6.98 (dd, J_1 = 8.8 Hz, J_2 = 0.8 Hz, 1H), 4.43-4.34 (m, 1H), 2.72 (s, 3H), 2.32-2.21(m, 2H), 1.98-1.93 (m, 4H), 1.78 (d, *J* = 12.4 Hz, 1H), 1.54-1.42 (m, 2H), 1.36-1.29 (m, 1H); ^{13}C NMR (100 MHz, $CDCl_3$):δ 153.6, 153.5, 144.4, 143.4, 143.3, 129.9, 129.4, 126.4, 126.1, 123.72, 123.69, 121.5, 121.1, 120.3, 110.5, 53.9, 30.4, 26.1, 25.5, 21.4; HRMS (ESI, m/z) calcd. For $C_{22}H_{23}N_3O$ $[M+H]^+$: 359.1872; found: 359.1887.

3-cyclohexyl-1-(quinolin-2-yl)-1H-naphtho[1,2-d]imidazol-2(3H)-one (3ae)

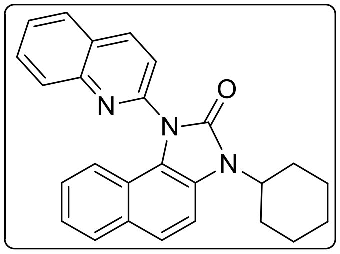

The general procedure 1 for imidazolone was followed. Column chromatography (SiO_2, eluting with 75:25 hexane/ethyl acetate) afforded the desired product as a creamy white powder (57.4 mg, 73% yield), mp 188-190 °C.

^{1}H NMR (400 MHz, $CDCl_3$): δ 8.42 (d, *J* = 8.4 Hz, 1H), 8.05-8.02 (m, 1H), 7.97 (dd, J_1 = 8.0 Hz, J_2 = 0.8 Hz, 1H), 7.84-7.82 (m, 2H), 7.77-7.73 (m, 1H), 7.64-7.62 (m, 2H), 7.54 (d, *J* = 8.8 Hz, 1H), 7.28-7.24 (m, 1H), 7.08-7.07 (m, 2H), 4.46-4.38 (m, 1H), 2.35-2.25 (m, 2H), 2.01-1.93 (m, 4H), 1.78 (d, *J* = 13.2 Hz, 1H), 1.54-1.44 (m, 2H), 1.38-1.30 (m, 1H); ^{13}C NMR (100 MHz, $CDCl_3$): δ 153.8, 149.6, 147.3, 138.9, 130.3, 130.0, 129.3, 129.1, 127.8, 127.6, 127.4, 126.3, 125.7, 123.5, 121.73, 121.68, 121.2, 120.7, 110.4, 53.7, 30.4, 26.2, 25.5; HRMS (ESI, m/z) calcd. For $C_{26}H_{24}N_3O$ $[M+H]^+$: 394.1919; found: 394.1920.

3-butyl-1-(pyrazin-2-yl)-1H-naphtho[1,2-d]imidazol-2(3H)-one (3af)

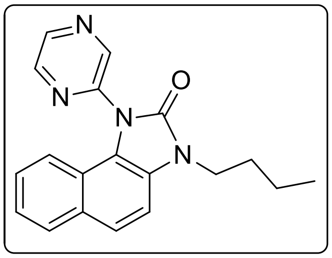

The general procedure 1 for imidazolone was followed. Column chromatography (SiO_2, eluting with 70:30 hexane/ethyl acetate) afforded the desired product as a light brown crystalline solid (41.3 mg, 65% yield), mp 140-142 °C.

^{1}H NMR (400 MHz, $CDCl_3$): δ 9.07 (s, 1H), 8.69-8.63 (m, 2H), 7.86 (d, *J* = 8.0 Hz, 1H), 7.73 (d, *J* = 8.8 Hz, 1H), 7.36-7.30 (m, 2H), 7.27-7.23 (m, 1H), 7.04 (d, *J* = 8.4 Hz, 1H), 4.03 (t, *J* = 7.2 Hz, 2H), 1.86-1.79 (m, 2H), 1.50-1.41 (m, 2H), 0.97 (t, *J* = 7.6 Hz, 3H);^{13}C NMR (100 MHz, $CDCl_3$): δ 153.9, 146.9, 144.4, 143.7, 143.4, 130.4, 129.6, 127.2, 126.2, 124.6, 123.8, 121.3, 121.0, 120.4, 109.2, 41.5, 30.7, 20.2, 13.8; HRMS (ESI, m/z) calcd. For $C_{19}H_{18}N_4O$ $[M+H]^+$: 319.1559; found: 319.1558.

3-(tert-butyl)-1-(pyrazin-2-yl)-1H-naphtho[1,2-d]imidazol-2(3H)-one (3ag)

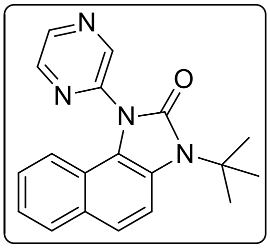

The general procedure 1 for imidazolone was followed. Column chromatography (SiO_2, eluting with 70:30 hexane/ethyl acetate) afforded the desired product as a creamy white powder (62.3 mg, 98% yield), mp 148-150 °C.

^{1}H NMR (400 MHz, $CDCl_3$): δ 9.00 (d, J = 1.6 Hz, 1H), 8.68 (d, J = 2.4 Hz, 1H), 8.63 (dd, J_1 = 2.4 Hz, J_2 = 1.2 Hz, 1H), 7.82 (d, J = 8.4 Hz, 1H), 7.76 (d, J = 9.2 Hz, 1H), 7.63 (d, J = 9.2 Hz, 1H), 7.32-7.28 (m, 1H), 7.21-7.16 (m, 1H), 6.85 (d, J = 8.4 Hz, 1H), 1.88 (s, 9H); ^{13}C NMR (100 MHz, $CDCl_3$): δ 153.8, 147.2, 145.1, 143.8, 143.6, 129.7, 129.1, 127.2, 126.0, 123.9, 123.2, 121.9, 121.3, 119.9, 113.1, 59.1, 29.8; HRMS (ESI, m/z) calcd. For $C_{19}H_{19}N_4O$ $[M+H]^+$: 319.1559; found: 319.1563.

3-cyclohexyl-1-(isoquinolin-1-yl)-1H-naphtho[1,2-d]imidazol-2(3H)-one (3ah)

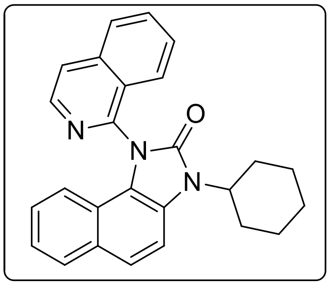

The general procedure 1 for imidazolone was followed. Column chromatography (SiO_2, eluting with 70:30 hexane/ethyl acetate) afforded the desired product as a white powder (23.6 mg, 30% yield), mp 184-186 °C.

^{1}H NMR (400 MHz, $CDCl_3$): δ 8.60 (d, J = 5.6 Hz, 1H), 8.00 (d, J = 8.4 Hz, 1H), 7.94 (d, J = 8.8 Hz, 1H), 7.91 (d, J = 6.0 Hz, 1H), 7.82-7.56 (m, 2H), 7.67 (d, J = 8.8 Hz, 1H), 7.61-7.57 (m, 2H), 7.22-7.18 (m, 1H), 6.99-6.95 (m, 1H), 6.44 (d, J = 8.4 Hz, 1H), 4.99-4.41 (m, 1H), 2.37-2.27 (m, 2H), 2.07-1.95 (m, 4H), 1.79 (d, J = 11.6 Hz, 1H), 1.56-1.44 (m, 2H), 1.39-1.31 (m, 1H); ^{13}C NMR (100 MHz, $CDCl_3$): δ 153.8, 141.7, 138.5, 131.5, 129.8, 129.1, 128.9, 127.1, 126.7, 126.1, 125.6, 123.5, 123.1, 122.8, 120.4, 120.1, 110.6, 53.7, 30.7, 26.2, 25.5; HRMS (ESI, m/z) calcd. For $C_{26}H_{24}N_3O$ $[M+H]^+$: 394.1919; found: 394.1920.

***N*-(2-(cyclohexylamino)naphthalen-1-yl)isoquinoline-1-carboxamide (3ah')**

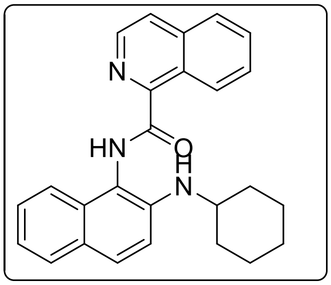

The general procedure 1 for imidazolone was followed. Column chromatography (SiO_2, eluting with 85:15 hexane/ethyl acetate) afforded the desired product as a yellow fluffy solid (32.4 mg, 41% yield), mp 134-136 °C.

^{1}H NMR (400 MHz, $CDCl_3$): δ 9.96 (S, 1H), 9.67-9.64 (m, 1H), 8.62 (d, J = 5.6 Hz, 1H), 7.92-7.90 (m, 2H), 7.81 (d, J = 8.4 Hz, 1H), 7.78- 7.69 (m, 4H), 7.43-7.39 (m, 1H), 7.28-7.21 (m, 2H), 3.52-3.46 (m, 1H), 2.12-2.09 (m, 2H), 1.79-1.74 (m, 2H), 1.64-1.60 (m, 1H), 1.42-1.19 (m, 5H); ^{13}C NMR (100 MHz, $CDCl_3$): δ 165.2, 160.5, 154.8, 147.9, 140.5, 131.6, 130.8, 129.1, 128.3, 128.0, 127.4, 127.1, 127.0, 125.0, 122.3, 120.8, 110.9, 33.6, 25.9, 25.0; HRMS (ESI, m/z) calcd. For $C_{26}H_{26}N_3O$ $[M+H]^+$: 396.2076; found: 396.2078.

_N_-(5-(cyclohexylamino)quinolin-6-yl)picolinamide (3ai)

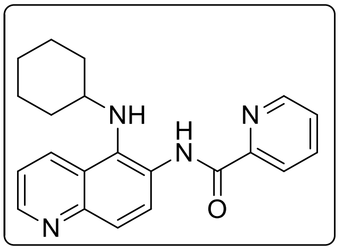

The general procedure 1 for imidazolone was followed. Column chromatography (SiO_2, eluting with 80:20 hexane/ethyl acetate) afforded the desired product as a pale yellow solid (42.2 mg, 61% yield), mp 146-148 °C.

^{1}H NMR (400 MHz, $CDCl_3$): δ 10.98 (s, 1H), 8.87 (d, J = 9.2 Hz, 1H), 8.81 (dd, J_1 = 4.0 Hz, J_2 = 1.6 Hz, 1H), 8.67-8.65 (m, 1H), 8.33 (dt, J_1 = 7.6 Hz, J_2 = 1.2 Hz, 1H), 8.29-8.27 (m, 1H), 7.94-7.89 (m, 2H), 7.49-7.46 (m, 1H), 7.37 (dd, J_1 = 8.4 Hz, J_2 = 4.0 Hz, 1H), 3.35 (s, 1H), 3.02-2.95 (m, 1H), 2.06-2.02 (m, 2H), 1.73-1.68 (m, 2H), 1.59-1.55 (m, 1H), 1.39-1.30 (m, 2H), 1.67-1.08 (m, 3H); ^{13}C NMR (100 MHz, $CDCl_3$): δ 162.3, 150.4, 148.9, 148.2, 146.2, 137.7, 131.6, 131.0, 130.6, 126.5, 125.9, 125.5, 123.6, 122.6, 120.8, 58.6, 34.8, 25.9, 25.4; HRMS (ESI, m/z) calcd. For $C_{21}H_{23}N_4O_3$ $[M+H]^+$: 347.1872; found: 347.1889.

3-methyl-2-(pyridin-2-yl)-3H-naphtho[1,2-d]imidazole (3aj)

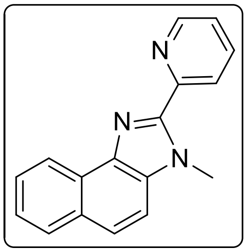

The general procedure 1 for imidazolone was followed. Column chromatography (SiO_2, eluting with 90:10 hexane/ethyl acetate) afforded the desired product as a colorless crystalline solid (26 mg, 50% yield), mp 108-110 °C.

^{1}H NMR (400 MHz, $CDCl_3$): δ 8.74 (d, J = 8.0 Hz, 1H), 8.70-8.68 (m, 1H), 8.51 (d, J = 8.0 Hz, 1H), 7.93 (d, J = 8.0 Hz, 1H), 7.85 (td, J_1 = 7.6 Hz, J_2 = 1.2 Hz, 1H), 7.74 (d, J = 8.8 Hz, 1H), 7.66-7.62 (m, 1H), 7.56-7.48 (m, 2H), 7.33-7.30 (m, 1H), 4.36 (s, 3H); ^{13}C NMR (100 MHz, $CDCl_3$): δ 150.9, 148.7, 148.3, 138.2, 136.9, 133.6, 130.6, 128.6, 127.2, 126.7, 124.8, 124.73, 124.67, 123.4, 122.1, 110.5, 33.1; HRMS (ESI, m/z) calcd. For $C_{17}H_{14}N_3$ $[M+H]^+$: 260.1188; found: 260.1196.

1-cyclohexyl-4-phenyl-3-(pyridin-2-yl)-1H-benzo[d]imidazol-2(3H)-one (3ak)

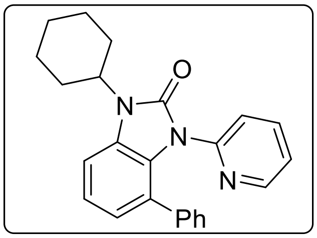

The general procedure 2 for imidazolone was followed. Column chromatography (SiO_2, eluting with 80:20 hexane/ethyl acetate) afforded the desired product as a white solid (29.5 mg, 40% yield), mp 196-198 °C.

^{1}H NMR (400 MHz, $CDCl_3$): δ 7.87-7.85 (m, 1H), 7.54 (td, J_1 = 8.0 Hz, J_2 = 2.0 Hz, 1H), 7.32 (d, J = 8.0 Hz, 1H), 7.25-7.17 (m, 2H), 7.06-6.89 (m, 7H), 4.38-4.29 (m, 1H), 2.32-2.22 (m, 2H), 1.94 (d, J = 10.8 Hz, 4H), 1.76 (d, J = 12.8 Hz, 1H), 1.51-1.42 (m, 2H), 1.35-1.25 (m, 1H); ^{13}C NMR (100 MHz, $CDCl_3$): δ 153.5, 148.5, 148.1, 138.7, 137.3, 130.2, 128.3, 127.6, 126.4, 126.1, 125.7, 123.8, 122.2, 122.0, 121.9, 108.3, 53.6, 29.9, 26.1, 25.5; HRMS (ESI, m/z) calcd. For $C_{24}H_{24}N_3O$ $[M+H]^+$: 370.1919; found: 370.1913.

1-(tert-butyl)-4-phenyl-3-(pyridin-2-yl)-1H-benzo[d]imidazol-2(3H)-one (3al)

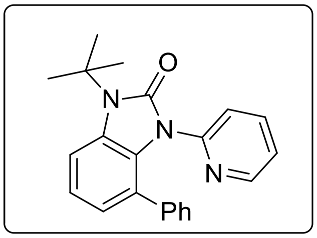

The general procedure 2 for imidazolone was followed was followed. Column chromatography (SiO_2, eluting with 80:20 hexane/ethyl acetate) afforded the desired product as a white solid (20.5 mg, 30% yield), mp 228-230 °C.

^{1}H NMR (400 MHz, $CDCl_3$): δ 7.93 (d, *J* = 3.6 Hz, 1H), 7.51-7.46 (m, 2H), 7.23 (d, *J* = 7.6 Hz, 1H), 7.14 (t, *J* = 8.0 Hz, 1H), 7.03-6.89 (m, 7H), 1.86 (s, 9H); ^{13}C NMR (100 MHz, $CDCl_3$): δ 153.8, 148.6, 148.1, 138.8, 137.2, 130.9, 128.4, 127.6, 126.3, 126.2, 125.6, 123.8, 122.8, 121.9, 121.5, 111.2, 58.7, 29.6; HRMS (ESI, m/z) calcd. For $C_{22}H_{22}N_3O$ $[M+H]^+$: 344.1763; found: 344.1752.

1-cyclohexyl-4-isopropyl-3-(pyridin-2-yl)-1H-benzo[d]imidazol-2(3H)-one (3am)

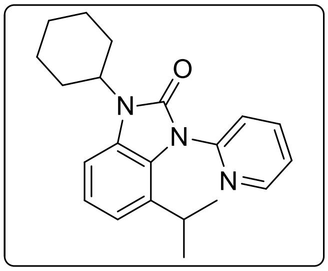

The general procedure 2 for imidazolone was followed was followed. Column chromatography (SiO_2, eluting with 80:20 hexane/ethyl acetate) afforded the desired product as a creamy white solid (43.5 mg, 65% yield), mp 164-166 °C.

^{1}H NMR (400 MHz, $CDCl_3$): δ 8.58 (dd, J_1 = 4.8 Hz, J_2 = 2.0 Hz, 1H), 7.89 (td, J_1 = 7.6 Hz, J_2 = 2.0 Hz, 1H), 7.57 (d, *J* = 8.0 Hz, 1H), 7.37-7.34 (m, 1H), 7.12-7.04 (m, 2H), 7.00-6.98 (m, 1H), 4.29-4.21 (m, 1H), 2.46-2.36 (m, 1H), 2.27-2.16 (m, 2H), 1.89 (d, *J* = 11.2 Hz, 4H), 1.73 (d, *J* = 12.8 Hz, 1H), 1.48-1.38 (m, 2H), 1.32-1.24 (m, 1H), 1.02 (d, *J* = 6.8 Hz, 6H); ^{13}C NMR (100 MHz, $CDCl_3$): δ 153.9, 150.6, 149.0, 138.4, 132.4, 129.8, 126.1, 123.5, 123.4, 122.3, 119.0, 106.9, 53.5, 29.9, 27.7, 26.1, 25.5, 23.2; HRMS (ESI, m/z) calcd. For $C_{21}H_{26}N_3O$ $[M+H]^+$: 336.2076; found: 336.2067.

4-(tert-butyl)-1-cyclohexyl-3-(pyridin-2-yl)-1H-benzo[d]imidazol-2(3H)-one (3an)

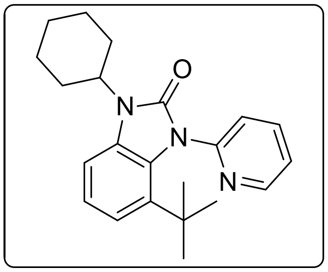

The general procedure 2 for imidazolone was followed was followed. Column chromatography (SiO_2, eluting with 80:20 hexane/ethyl acetate) afforded the desired product as a creamy white solid (40 mg, 57% yield), mp 160-162 °C.

^{1}H NMR (400 MHz, $CDCl_3$): δ 8.49 (dd, J_1 = 4.8 Hz, J_2 = 1.6 Hz, 1H), 7.86 (td, J_1 = 7.6 Hz, J_2 = 2.0 Hz, 1H), 7.60 (d, J = 8.0 Hz, 1H), 7.31-7.28 (m, 1H), 7.17 (dd, J_1 = 8.0 Hz, J_2 = 1.6 Hz, 1H), 7.12-7.04 (m, 2H), 4.22-4.14 (m, 1H), 2.29-2.18 (m, 2H), 1.91-1.85 (m, 4H), 1.72 (d, J = 12.8 Hz, 1H), 1.46-1.35 (m, 2H), 1.30-1.22 (m, 1H), 1.07 (s, 9H); ^{13}C NMR (100 MHz, $CDCl_3$): δ 155.6, 153.8, 148.7, 138.1, 136.5, 132.2, 127.3, 124.5, 123.2, 122.6, 121.7, 107.1, 53.8, 34.9, 31.4, 29.7, 26.2, 25.5; HRMS (ESI, m/z) calcd. For $C_{22}H_{28}N_3O$ $[M+H]^+$: 350.2232; found: 350.2225.

1-cyclohexyl-4-ethyl-3-(pyridin-2-yl)-1H-benzo[d]imidazol-2(3H)-one (3ao)

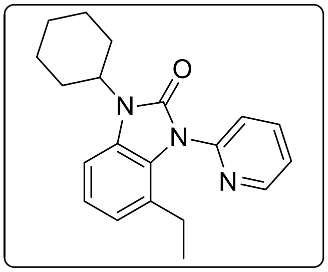

The general procedure 2 for imidazolone was followed was followed. Column chromatography (SiO_2, eluting with 90:10 hexane/ethyl acetate) afforded the desired product as a creamy white solid (16 mg, 25% yield).

^{1}H NMR (400 MHz, $CDCl_3$): δ 8.60-8.58 (m, 1H), 7.89 (td, J_1 = 7.6 Hz, J_2 = 1.6 Hz, 1H), 7.58 (dt, J_1 = 8.0 Hz, J_2 = 0.8 Hz, 1H), 7.37-7.34 (m, 1H), 7.09-7.04 (m, 2H), 6.89-6.88 (m, 1H), 4.30-4.22 (m, 1H), 2.26-2.19 (m, 4H), 1.89 (d, J = 9.6 Hz, 4H), 1.73 (d, J = 12.8 Hz, 1H), 1.48-1.38 (m, 2H), 1.32-1.24 (m, 1H), 0.89 (t, J = 7.2 Hz, 3H); ^{13}C NMR (100 MHz, $CDCl_3$): δ 153.8, 150.0, 148.9, 138.3, 129.7, 127.4, 126.6, 123.6, 123.4, 122.4, 122.1, 107.2, 53.5, 29.9, 26.1, 25.5, 25.1, 14.2; HRMS (ESI, m/z) calcd. For $C_{20}H_{24}N_3O$ $[M+H]^+$: 322.1919; found: 322.1913.

***N*-(2-(cyclohexylamino)-3,5-dimethoxyphenyl)picolinamide (3ap)**

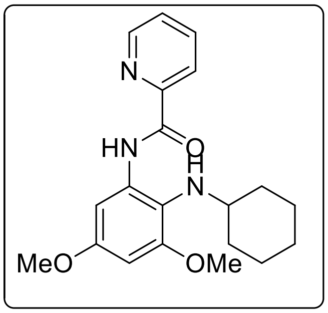

The general procedure for amination in aniline system was followed was followed. Column chromatography (SiO_2, eluting with 90:10 hexane/ethyl acetate) afforded the desired product as a gummy liquid (51 mg, 72% yield).

^{1}H NMR (400 MHz, $CDCl_3$): δ 11.04 (s, 1H), 8.62-8.60 (m, 1H), 8.25 (dt, J_1 = 8.0 Hz, J_2 = 0.8 Hz, 1H), 7.89 (d, J = 2.8 Hz, 1H), 7.85 (td, J_1 = 8.0 Hz, J_2 = 2.0 Hz, 1H), 7.43-7.39 (m, 1H), 6.25 (d, J = 2.8 Hz, 1H), 3.82 (s, 3H), 3.77 (s, 3H), 2.74-2.68 (m, 1H), 1.93 (d, J = 12.8 Hz, 2H), 1.69-1.65 (m, 2H), 1.50 (s, 1H), 1.27-1.19 (m, 3H), 1.15-1.09 (m, 2H); ^{13}C NMR (100 MHz, $CDCl_3$): δ 162.0, 156.8, 154.9, 150.6, 148.3, 137.5, 134.8, 126.2, 122.3, 119.5, 95.9, 94.9, 57.8, 55.7, 55.6, 34.2, 26.1, 25.3; HRMS (ESI, m/z) calcd. For $C_{20}H_{26}N_3O_3$ $[M+H]^+$: 356.1974; found: 356.1973.

N-(2-(cyclohexylamino)-3,5-dimethylphenyl)picolinamide (3aq)

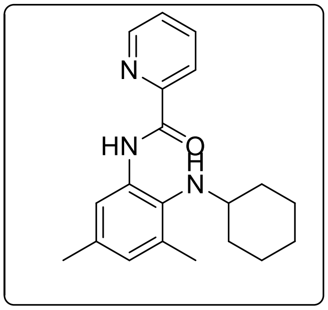

The general procedure for amination in aniline system was followed. Column chromatography (SiO_2, eluting with 95:5 hexane/ethyl acetate) afforded the desired product as a gummy liquid (33.5 mg, 52% yield).

^{1}H NMR (400 MHz, $CDCl_3$): δ 10.87 (s, 1H), 8.63-8.61 (m, 1H), 8.29 (dt, J_1 = 7.6 Hz, J_2 = 1.2 Hz, 1H), 8.23 (s, 1H), 7.88 (td, J_1 = 7.6 Hz, J_2 = 1.6 Hz, 1H), 7.45-7.42 (m, 1H), 6.75 (m, 1H), 2.84-2.77 (m, 1H), 2.31 (s, 3H), 2.26 (s, 3H), 2.06-2.03 (m, 2H), 1.71-1.68 (m, 2H), 1.55-1.54 (m, 1H), 1.32-1.25 (m, 2H), 1.13-1.08 (m, 3H); ^{13}C NMR (100 MHz, $CDCl_3$): δ 161.9, 150.8, 148.1, 137.5, 133.5, 133.2, 131.5, 126.6, 126.1, 122.4, 118.6, 58.0, 34.7, 25.9, 25.5, 21.3, 18.3; HRMS (ESI, m/z) calcd. For $C_{20}H_{26}N_3O$ $[M+H]^+$: 324.2076; found: 324.2066.

N-(2-(cyclopentylamino)-3,5-dimethoxyphenyl)picolinamide (3ar)

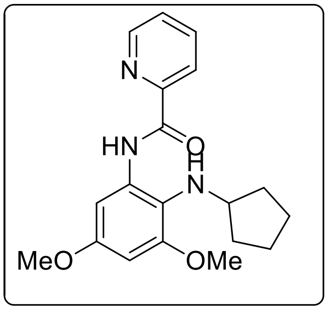

The general procedure for amination in aniline system was followed was followed. Column chromatography (SiO_2, eluting with 90:10 hexane/ethyl acetate) afforded the desired product as a gummy liquid (34 mg, 50% yield).

^{1}H NMR (400 MHz, $CDCl_3$): δ 11.13 (s, 1H), 8.64-8.62 (m, 1H), 8.26 (dt, J_1 = 8.0 Hz, J_2 = 0.8 Hz, 1H), 7.92 (d, J = 2.4 Hz, 1H), 7.86 (td, J_1 = 7.6 Hz, J_2 = 1.6 Hz, 1H), 7.45-7.41 (m, 1H), 6.26 (d, J = 2.8 Hz, 1H), 3.84 (s, 3H), 3.79 (s, 3H), 3.47 (quintet, J = 5.6 Hz, 1H), 1.80-1.70 (m, 4H), 1.56-1.46 (m, 4H); ^{13}C NMR (100 MHz, $CDCl_3$): δ 162.1, 157.1, 155.2, 150.6, 148.3, 137.5, 135.3, 126.2, 122.3, 119.9, 95.8, 95.0, 60.8, 55.6, 33.2, 23.6; HRMS (ESI, m/z) calcd. For $C_{19}H_{24}N_3O_3$ $[M+H]^+$: 342.1818; found: 342.1819.

_N_-(2-(butylamino)-3,5-dimethoxyphenyl)picolinamide (3as)

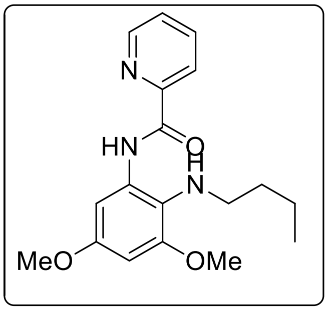

The general procedure for amination in aniline system was followed was followed. Column chromatography (SiO_2, eluting with 90:10 hexane/ethyl acetate) afforded the desired product as a gummy liquid (31 mg, 47% yield).

^{1}H NMR (400 MHz, $CDCl_3$): δ 11.03 (s, 1H), 8.65-8.63 (m, 1H), 8.28-8.25 (m, 1H), 7.90-7.86 (m, 2H), 7.46-7.43 (m, 1H), 6.28 (d, J = 2.4 Hz, 1H), 3.84 (s, 3H), 3.81 (s, 3H), 2.85 (t, J = 7.2 Hz, 2H), 1.61 (quintet, J = 7.2 Hz, 2H), 1.49-1.39 (m, 2H), 0.89 (t, J = 7.2 Hz, 3H); ^{13}C NMR (100 MHz, $CDCl_3$): δ 162.3, 157.1, 154.6, 150.5, 148.3, 137.5, 134.5, 126.3, 122.3, 96.2, 95.2, 55.8, 55.7, 49.8, 32.8, 20.3, 14.1; HRMS (ESI, m/z) calcd. For $C_{18}H_{24}N_3O_3$ $[M+H]^+$: 330.1818; found: 330.1822.

_N_-(2-(sec-butylamino)-3,5-dimethoxyphenyl)picolinamide (3at)

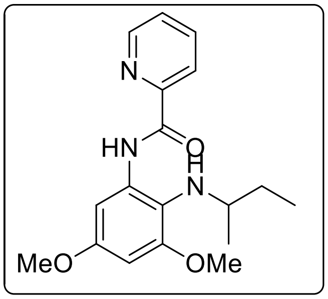

The general procedure for amination in aniline system was followed was followed. Column chromatography (SiO_2, eluting with 90:10 hexane/ethyl acetate) afforded the desired product as a gummy liquid (27.6 mg, 42% yield).

^{1}H NMR (400 MHz, $CDCl_3$): δ 11.03 (s, 1H), 8.63-8.62 (m, 1H), 8.26 (dt, J_1 = 7.6 Hz, J_2 = 1.2 Hz, 1H), 7.89-7.85 (m, 2H), 7.45-7.42 (m, 1H), 6.27 (d, J = 2.4 Hz, 1H), 3.84 (s, 3H), 3.79 (s, 3H), 2.98-2.90 (m, 1H), 1.68-1.57 (m, 1H), 1.47-1.39 (m, 1H), 1.04 (d, J = 6.4 Hz, 3H), 0.95 (t, J = 12.8 Hz, 3H); ^{13}C NMR (100 MHz, $CDCl_3$): δ 162.1, 156.9, 154.9, 150.6, 148.3, 137.5, 134.9, 126.2, 122.3, 96.1, 95.1, 55.7, 30.2, 19.9, 10.5; HRMS (ESI, m/z) calcd. For $C_{18}H_{24}N_3O_3$ $[M+H]^+$: 330.1818; found: 330.1826.

3-cyclohexyl-2-(pyridin-2-yl)-3H-naphtho[1,2-d]imidazole (5a)

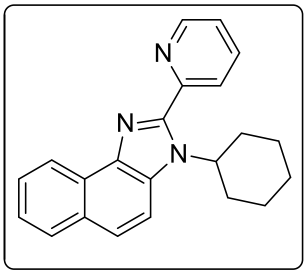

The general procedure for imidazole was followed. Column chromatography (SiO_2, eluting with 90:10 hexane/ethyl acetate) afforded the desired product as a white solid (58 mg, 88% yield), mp 88-90°C.

^{1}H NMR (400 MHz, $CDCl_3$): δ 8.77 (d, J = 8.0 Hz, 1H), 8.72-8.70 (m, 1H), 8.36 (d, J = 8.0 Hz, 1H), 7.92 (d, J = 8.0 Hz, 1H), 7.89-7.83 (m, 2H), 7.68 (d, J = 8.8 Hz, 1H), 7.64-7.60 (m, 1H), 7.51-7.47 (m, 1H), 7.36-7.33 (m, 1H), 5.59-5.52 (m, 1H), 2.39-2.29 (m, 2H), 2.16-2.12 (m, 2H), 1.99-1.94 (m, 2H), 1.81 (d, J = 12.4 Hz, 1H), 1.52-1.34 (m, 3H); ^{13}C NMR (100 MHz, $CDCl_3$): δ 148.9, 137.0, 131.3, 130.1, 128.6, 128.2, 126.6, 125.83, 125.8, 124.9, 123.81, 123.79, 123.55, 123.53, 122.2, 113.7, 57.2, 31.8, 26.3, 25.6; HRMS (ESI, m/z) calcd. For $C_{22}H_{22}N_3$ $[M+H]^+$: 328.1814; found: 328.1822.

3-cyclopentyl-2-(pyridin-2-yl)-3H-naphtho[1,2-d]imidazole (5b)

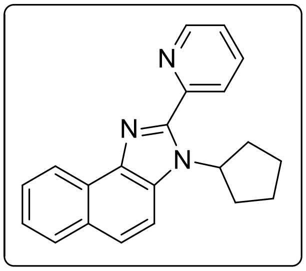

The general procedure for imidazole was followed. Column chromatography (SiO_2, eluting with 90:10 hexane/ethyl acetate) afforded the desired product as a light brown solid (50 mg, 80% yield), mp 146−148 °C.

^{1}H NMR (400 MHz, $CDCl_3$): δ 8.73-8.72 (m, 1H), 8.51-8.48 (m, 1H), 8.25 (dt, J_1 = 8.0 Hz, J_2 = 1.2 Hz, 1H), 8.02-7.98 (m, 2H), 7.81 (d, J = 9.2 Hz, 1H), 7.75 (d, J = 9.2 Hz, 1H), 7.62-7.58 (m, 1H), 7.50-7.46 (m, 2H), 6.09 (quintet, J = 9.2 Hz, 1H), 2.25-2.14 (m, 4H), 2.05-1.98 (m, 2H), 1.75-1.68 (m, 2H); ^{13}C NMR (100 MHz, $CDCl_3$): δ 151.1, 149.4, 149.3, 138.9, 137.9, 130.9, 130.2, 128.9, 127.4, 126.9, 125.7, 125.3, 124.5, 124.1, 121.8, 113.7, 57.7, 31.0, 25.3; HRMS (ESI, m/z) calcd. For $C_{21}H_{20}N_3$ $[M+H]^+$: 314.1657; found: 314.1653.

3-propyl-2-(pyridin-2-yl)-3H-naphtho[1,2-d]imidazole (5c)

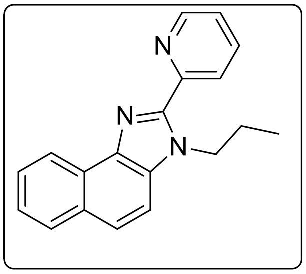

The general procedure for imidazole was followed. Column chromatography (SiO_2, eluting with 90:10 hexane/ethyl acetate) afforded the desired product as a light brown solid (40 mg, 70% yield), mp 58-60 °C.

^{1}H NMR (400 MHz, $CDCl_3$): δ 8.75 (d, J = 8.0 Hz, 1H), 8.68-8.66 (m, 1H), 8.52 (dt, J_1 = 8.0 Hz, J_2 = 0.8 Hz, 1H), 7.93 (d, J = 8.4 Hz, 1H), 7.84 (td, J_1 = 8.0 Hz, J_2 = 1.6 Hz, 1H), 7.72 (d, J = 8.8 Hz, 1H), 7.66-7.62 (m, 1H), 7.55 (d, J = 8.8 Hz, 1H), 7.52-7.48 (m, 1H), 7.31-7.28 (m, 1H), 4.89 (t, J = 7.2 Hz, 2H), 1.98-1.89 (m, 2H), 0.93 (t, J = 7.2 Hz, 3H), ^{13}C NMR (100 MHz, $CDCl_3$): δ 151.0, 148.7, 148.0, 138.2, 136.8, 133.1, 130.6, 128.5, 127.3, 126.6, 124.74, 124.66, 124.5, 123.3, 122.0, 110.9, 47.2, 24.0, 11.4; HRMS (ESI, m/z) calcd. For $C_{19}H_{18}N_3$ $[M+H]^+$: 288.1501; found: 288.1497.

3-butyl-2-(pyridin-2-yl)-3H-naphtho[1,2-d]imidazole (5d)

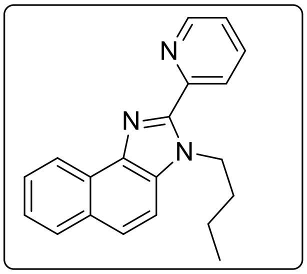

The general procedure for imidazole was followed. Column chromatography (SiO_2, eluting with 90:10 hexane/ethyl acetate) afforded the desired product as a light brown solid (50 mg, 83% yield), mp 158-160 °C.

^{1}H NMR (400 MHz, $CDCl_3$): δ 9.69 (d, J = 8.4 Hz, 1H), 9.41 (d, J = 8.0 Hz, 1H), 8.78 (d, J = 4.4 Hz, 1H), 8.12 (td, J_1 = 7.6 Hz, J_2 = 1.2 Hz, 1H), 7.97 (d, J = 2.0 Hz, 1H), 7.95 (d, J = 3.2 Hz, 1H), 7.86-7.82 (m, 1H), 7.68-7.61 (m, 2H), 7.54 (dd, J_1 = 7.6 Hz, J_2 = 4.8 Hz, 1H), 5.04 (t, J = 7.6 Hz, 2H), 1.98-1.94 (m, 2H), 1.44 (sextet, J = 7.6 Hz, 2H), 0.97 (t, J = 7.2 Hz, 3H); ^{13}C NMR (100 MHz, $CDCl_3$): δ 149.6, 143.7, 143.2, 138.4, 131.7, 130.1, 129.2, 129.0, 128.6, 128.2, 127.8, 126.5, 125.3, 109.6, 46.9, 32.0, 20.0, 13.6; HRMS (ESI, m/z) calcd. For $C_{20}H_{20}N$ $[M+H]^+$: 302.1657; found: 302.1663.

3-isobutyl-2-(pyridin-2-yl)-3H-naphtho[1,2-d]imidazole (5e)

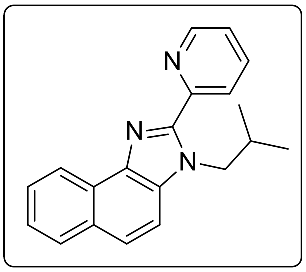

The general procedure for imidazole was followed. Column chromatography (SiO_2, eluting with 90:10 hexane/ethyl acetate) afforded the desired product as a brown solid (52 mg, 86% yield), mp 74-76 °C.

^{1}H NMR (400 MHz, $CDCl_3$): δ 8.76-8.73 (m, 1H), 8.67-8.66 (m, 1H), 8.52 (dt, J_1 = 8.0 Hz, J_2 = 1.2 Hz, 1H), 7.93 (d, J = 7.6 Hz, 1H), 7.84 (td, J_1 = 8.0 Hz, J_2 = 2.0 Hz, 1H), 7.72 (d, J = 8.8 Hz, 1H), 7.66-7.62 (m, 1H), 7.55 (d, J = 9.2 Hz, 1H), 7.52-7.47 (m, 1H), 7.32-7.28 (m, 1H), 4.79 (d, J = 7.2 Hz, 2H), 2.31-2.20 (m, 1H), 0.88 (d, J = 6.4 Hz, 6H); ^{13}C NMR (100 MHz, $CDCl_3$): δ 151.2, 148.5, 148.2, 138.2, 136.8, 133.5, 130.5, 128.5, 127.3, 126.6, 124.8, 124.7, 124.4, 123.3, 122.0, 111.3, 52.6, 30.1, 20.2; HRMS (ESI, m/z) calcd. For $C_{20}H_{20}N_3$ $[M+H]^+$: 302.1579; found: 302.1658.

3-(sec-butyl)-2-(pyridin-2-yl)-3H-naphtho[1,2-d]imidazole (5f)

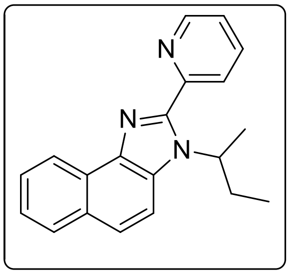

The general procedure for imidazole was followed. Column chromatography (SiO_2, eluting with 90:10 hexane/ethyl acetate) afforded the desired product as a light brown solid (48 mg, 80% yield), mp 88-90 °C.

^{1}H NMR (400 MHz, $CDCl_3$): δ 8.76 (d, J = 8.0 Hz, 1H), 8.70-8.68 (m, 1H), 8.37 (dt, J_1 = 8.0 Hz, J_2 = 0.8 Hz, 1H), 7.92 (d, J = 8.0 Hz, 1H), 7.85 (td, J_1 = 7.6 Hz, J_2 = 2.0 Hz, 1H), 7.77 (d, J = 8.8 Hz, 1H), 7.67 (d, J = 8.8 Hz, 1H), 7.65-7.61 (m, 1H), 7.52-7.47 (m, 1H), 7.33-7.30 (m, 1H), 5.89-5.81 (m, 1H), 2.33-2.21 (m, 1H), 2.03-1.92 (m, 1H), 1.76 (d, J = 7.2 Hz, 3H), 0.74 (d, J = 7.2 Hz, 3H); ^{13}C NMR (100 MHz, $CDCl_3$): δ 151.3, 149.2, 148.7, 139.1, 136.9, 131.1, 130.1, 128.3, 127.4, 126.5, 125.7, 124.8, 123.8, 123.4, 122.1, 113.3, 54.9, 28.8, 20.2, 11.2; HRMS (ESI, m/z) calcd. For $C_{20}H_{20}N_3$ $[M+H]^+$: 302.1579; found: 302.1660.

3-(tert-butyl)-2-(pyridin-2-yl)-3H-naphtho[1,2-d]imidazole (5g)

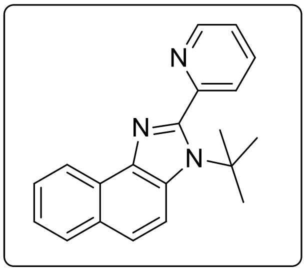

The general procedure for imidazole was followed. Column chromatography (SiO_2, eluting with 80:20 hexane/ethyl acetate) afforded the desired product as light brown solid (43 mg, 72 % yield), mp 146-148 °C.

^{1}H NMR (400 MHz, $CDCl_3$): δ 8.75 (d, J = 8.0 Hz, 1H), 8.71-8.69 (m, 1H), 7.92-7.88 (m, 2H), 7.84 (td, J_1 = 7.6 Hz, J_2 = 2.0 Hz, 1H), 7.77 (d, J = 7.6 Hz, 1H), 7.70 (d, J = 9.2 Hz, 1H), 7.61-7.57 (m, 1H), 7.51-7.47 (m, 1H), 7.40-7.37 (m, 1H), 1.70 (m, 9H); ^{13}C NMR (100 MHz, $CDCl_3$): δ 148.9, 136.6, 130.0, 128.0, 126.6, 125.9, 125.1, 124.0, 123.6, 122.5, 122.4, 114.9, 31.5; HRMS (ESI, m/z) calcd. For $C_{20}H_{20}N_3$ $[M+H]^+$:302.1579; found:302.1660.

3-benzyl-2-(pyridin-2-yl)-3H-naphtho[1,2-d]imidazole (5h)

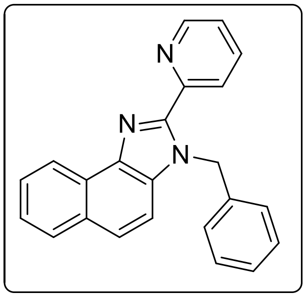

The general procedure 1 for imidazolone was followed. Column chromatography (SiO_2, eluting with 90:10 hexane/ethyl acetate) afforded the desired product as a pale yellow solid (26.8 mg, 40% yield), mp 144-142 °C.

^{1}H NMR (400 MHz, $CDCl_3$): δ 8.78 (d, *J* = 8.4 Hz, 1H), 8.62-8.60 (m, 1H), 8.57 (d, *J* = 8.0 Hz, 1H), 7.91 (d, *J* = 8.0 Hz, 1H), 7.84 (td, J_1 = 8.0 Hz, J_2 = 2.0 Hz, 1H), 7.67-7.63 (m, 2H), 7.52- 7.48 (m, 1H), 7.44 (d, *J* = 8.8 Hz, 1H), 7.30-7.27 (m, 1H), 7.22-7.13 (m, 5H), 6.30 (s, 2H); ^{13}C NMR (100 MHz, $CDCl_3$): δ 150.6, 148.7, 147.9, 137.6, 136.9, 133.2, 130.7, 128.7, 128.5, 127.5, 127.1, 126.8, 124.9, 124.7, 123.6, 122.1, 111.1, 49.2; HRMS (ESI, m/z) calcd. For $C_{24}H_{18}N_3$ $[M+H]^+$: 336.1501; found: 336.1502.

3-(furan-2-ylmethyl)-2-(pyridin-2-yl)-3H-naphtho[1,2-d]imidazole (5i)

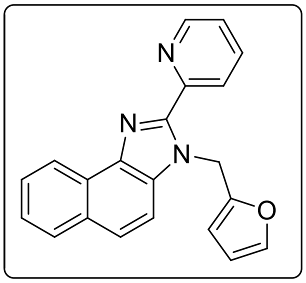

The general procedure 1 for imidazolone was followed. Column chromatography (SiO_2, eluting with 90:10 hexane/ethyl acetate) afforded the desired product as a yellow solid (24.7 mg, 38% yield), mp 122-124 °C.

^{1}H NMR (400 MHz, $CDCl_3$): δ 8.75 (d, *J* = 8.4 Hz, 1H), 8.71-8.69 (m, 1H), 8.56 (d, *J* = 8.0 Hz, 1H), 7.93 (d, *J* = 8.4 Hz, 1H), 7.86 (td, J_1 = 8.0 Hz, J_2 = 2.0 Hz, 1H), 7.76-7.70 (m, 2H), 7.66-7.62 (m, 1H), 7.52-7.48 (m, 1H), 7.35-7.31 (m, 1H), 7.26 (dd, J_1 = 1.6 Hz, J_2 = 0.8 Hz, 1H), 6.29 (s, 2H), 6.22-6.19 (m, 2H); ^{13}C NMR (100 MHz, $CDCl_3$): δ 150.8, 148.5, 147.4, 142.3, 137.0, 133.1, 130.7, 128.5, 127.0, 126.7, 124.9, 124,8, 123.6, 122.1, 111.1, 110.5, 108.3, 42.3; HRMS (ESI, m/z) calcd. For $C_{21}H_{16}N_3O$ $[M+H]^+$: 326.1293; found: 326.1294.

3-((3r,5r,7r)-adamantan-1-ylmethyl)-2-(pyridin-2-yl)-3H-naphtho[1,2-d]imidazole (5j)

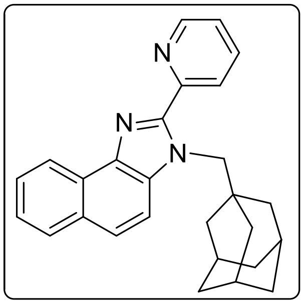

The general procedure for imidazole was followed. Column chromatography (SiO_2, eluting with 90:10 hexane/ethyl acetate) afforded the desired product as a creamy white solid (61 mg, 78% yield), mp 176−178 °C.

^{1}H NMR (400 MHz, $CDCl_3$): δ 8.75 (d, *J* = 8.0 Hz, 1H), 8.67-8.65 (m, 1H), 8.41 (dt, J_1 = 8.0 Hz, J_2 = 0.8 Hz, 1H), 7.93 (d, *J* = 8.0 Hz, 1H), 7.86 (td, J_1 = 7.6 Hz, J_2 = 2.0 Hz, 1H), 7.70 (d, *J* = 8.8 Hz, 1H), 7.66-7.62 (m, 1H), 7.59 (d, *J* = 8.8 Hz, 1H), 7.51-7.47 (m, 1H), 7.33-7.30 (m, 1H), 1.79 (s, 3H), 1.57-1.36 (m, 14H); ^{13}C NMR (100 MHz, $CDCl_3$): δ 152.0, 149.1, 148.4, 137.1, 134.2, 130.4, 128.7, 128.4, 127.1, 126.7, 125.2, 124.8, 124.1, 123.5, 122.0, 112.4, 55.8, 41.0, 36.8, 36.6, 28.3; HRMS (ESI, m/z) calcd. For $C_{27}H_{28}N_3$ $[M+H]^+$: 394.2283; found: 394.2304.

***tert*-butyl 4-(2-(pyridin-2-yl)-3H-naphtho[1,2-d]imidazol-3-yl)piperidine-1-carboxylate (5k)**

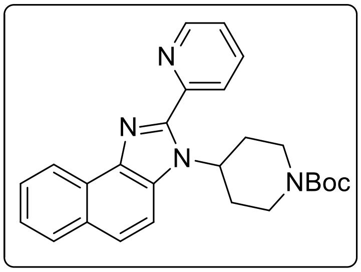

The general procedure for imidazole was followed. Column chromatography (SiO_2, eluting with 85:15 hexane/ethyl acetate) afforded the desired product as a creamy white solid (59 mg, 69 % yield), mp 112-114 °C.

^{1}H NMR (400 MHz, $CDCl_3$): δ 8.76 (d, *J* = 8.0 Hz, 1H), 8.69-8.67 (m, 1H), 8.44 (d, *J* = 8.0 Hz, 1H), 7.93-7.87 (m, 2H), 7.74 (d, *J* = 9.2 Hz, 1H), 7.68 (d, *J* = 9.2 Hz, 1H), 7.65-7.61 (m, 1H), 7.52-7.48 (m, 1H), 7.37-7.34 (m, 1H), 5.94-5.85 (m, 1H), 4.35 (s, 2H), 2.88 (t, *J* = 9.6 Hz, 2H), 2.59-2.49 (m, 2H), 2.10 (dd, J_1 = 12.0 Hz, J_2 = 2.0 Hz, 2H), 1.53 (s, 9H); ^{13}C NMR (100 MHz, $CDCl_3$): δ 154.9, 148.8, 148.3, 137.2, 131.1, 130.1, 128.3, 126.8, 125.8, 125.1, 124.3, 123.7, 122.1, 113.2, 80.0, 55.4, 30.8, 28.6; HRMS (ESI, m/z) calcd. For $C_{26}H_{29}N_4O_2$ $[M+H]^+$: 429.2291; found: 429.2285.

3-cyclohexyl-2-(5-methylpyrazin-2-yl)-3H-naphtho[1,2-d]imidazole (5l)

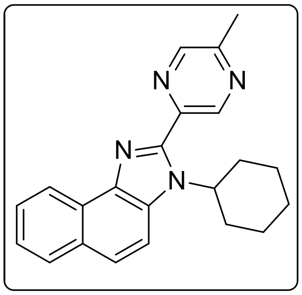

The general procedure for imidazole was followed. Column chromatography (SiO_2, eluting with 85:15 hexane/ethyl acetate) afforded the desired product as a pale yellow powder (40.3 mg, 59% yield), mp 188-190 °C.

^{1}H NMR (400 MHz, $CDCl_3$): δ 9.46 (s, 1H), 8.74 (d, *J* = 8.0 Hz, 1H), 8.51 (s, 1H), 7.91 (d, *J* = 8.0 Hz, 1H), 7.82 (d, *J* = 9.2 Hz, 1H), 7.68 (d, *J* = 9.2 Hz, 1H), 7.65-7.61 (m, 1H), 7.52-7.48 (m, 1H), 5.45-5.37 (m, 1H), 2.65 (s, 3H), 2.38-2.29 (m, 2H), 2.11 (d, *J* = 11.2 Hz, 2H), 1.96 (d, *J* = 12.8 Hz, 2H), 1.81 (d, *J* = 11.6 Hz, 1H), 1.50-1.34 (m, 3H); ^{13}C NMR (100 MHz, $CDCl_3$): δ 153.1, 146.17, 146.16, 145.7, 142.7, 131.5, 130.1, 128.3, 127.3, 126.7, 125.0, 124.1, 122.1, 113.5, 57.3, 31.9, 26.2, 25.6, 21.7; HRMS (ESI, m/z) calcd. For $C_{22}H_{23}N_4$ $[M+H]^+$: 343.1923; found: 343.1926.

3-cyclohexyl-2-(quinolin-2-yl)-3H-naphtho[1,2-d]imidazole (5m)

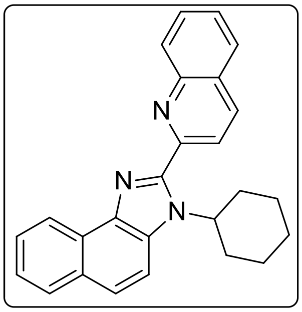

The general procedure for imidazole was followed. Column chromatography (SiO_2, eluting with 90:10 hexane/ethyl acetate) afforded the desired product as a creamy white solid (38 mg, 50% yield), mp 180-182 °C.

^{1}H NMR (400 MHz, $CDCl_3$): δ 8.80 (d, *J* = 8.0 Hz, 1H), 8.61 (d, *J* = 8.8 Hz, 1H), 8.31 (d, *J* = 8.4 Hz, 1H), 8.11 (d, *J* = 8.8 Hz, 1H), 7.93 (d, *J* = 8.0 Hz, 1H), 7.89-7.86 (m, 2H), 7.78-7.73 (m, 1H), 7.70 (d, *J* = 9.2 Hz, 1H), 7.67-7.63 (m, 1H), 7.60-7.56 (m, 1H), 7.53-7.49 (m, 1H), 6.14-6.06 (m, 1H), 2.47-2.37 (m, 2H), 2.27-2.24 (m, 2H), 2.02 (d, *J* = 13.6 Hz, 2H), 1.85 (d, *J* = 12.8 Hz, 1H), 1.61-1.50 (m, 2H), 1.47-1.39 (m, 1H); ^{13}C NMR (100 MHz, $CDCl_3$): δ 151.0, 148.3, 147.4, 136.6, 132.0, 130.1, 129.83, 129.79, 128.3, 127.8, 127.6, 127.5, 127.2, 126.6, 124.9, 124.1, 122.9, 122.2, 113.8, 57.5, 32.0, 26.5, 25.8; HRMS (ESI, m/z) calcd. For $C_{26}H_{24}N_3$ $[M+H]^+$: 378.1970; found: 378.1974.

5-bromo-3-cyclohexyl-2-(pyridin-2-yl)-3H-naphtho[1,2-d]imidazole (5n)

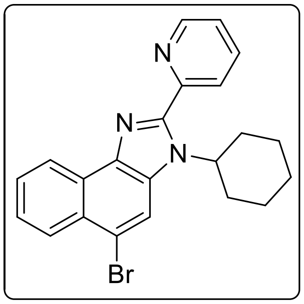

The general procedure for imidazole was followed. Column chromatography (SiO_2, eluting with 90:10 hexane/ethyl acetate) afforded the desired product as a creamy white powder (67 mg, 83% yield), mp 154-156 °C.

^{1}H NMR (400 MHz, $CDCl_3$): δ 8.79 (d, J = 8.0 Hz, 1H), 8.72-8.70 (m, 1H), 8.35 (dt, J_1 = 8.0 Hz, J_2 = 0.8 Hz, 1H), 8.31-8.29 (m, 1H), 8.19 (s, 1H), 7.88 (td, J_1 = 7.6 Hz, J_2 = 2.0 Hz, 1H), 7.69-7.65 (m, 1H), 7.62-7.57 (m, 1H), 7.38-7.34 (m, 1H), 5.59-5.52 (m, 1H), 2.33-2.23 (m, 2H), 2.16-2.12 (m, 2H), 2.00-1.96 (m, 2H), 1.82-1.79 (m, 1H), 1.52-1.37 (m, 3H); ^{13}C NMR (100 MHz, $CDCl_3$): δ 148.9, 137.0, 131.2, 128.6, 128.2, 127.8, 127.3, 126.8, 126.1, 125.8, 123.7, 122.5, 117.6, 57.4, 31.9, 26.2, 25.5; HRMS (ESI, m/z) calcd. For $C_{22}H_{21}BrN_3$ $[M+H]^+$: 406.0919; found: 406.0926.

3-cyclohexyl-5-fluoro-2-(pyridin-2-yl)-3H-naphtho[1,2-d]imidazole (5o)

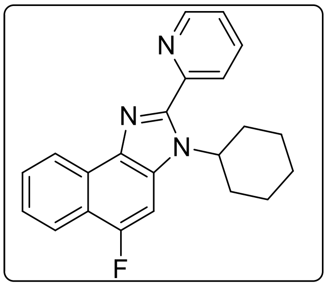

The general procedure for imidazole was followed. Column chromatography (SiO_2, eluting with 90:10 hexane/ethyl acetate) afforded the desired product as a white solid (63.5 mg, 92% yield), mp 128-130 °C.

^{1}H NMR (400 MHz, $CDCl_3$): δ 8.77 (d, J = 8.0 Hz, 1H), 8.71-8.69 (m, 1H), 8.34 (d, J = 8.0 Hz, 1H), 8.14 (d, J = 8.0 Hz, 1H), 7.87 (td, J_1 = 7.6 Hz, J_2 = 1.6 Hz, 1H), 7.69-7.66 (m, 1H), 7.57-7.53 (m, 2H), 7.36-7.33 (m, 1H), 5.61-5.23 (m, 1H), 2.31-2.21 (m, 2H), 2.15-2.11 (m, 2H), 1.99-1.95 (m, 2H), 1.82-1.78 (m, 1H), 1.52-1.33 (m, 3H); ^{13}C NMR (100 MHz, $CDCl_3$): δ 155.7 (d, J = 245.4 Hz), 151.0, 148.9, 136.9, 129.9 (d, J = 13.4 Hz), 128.6, 127.6, 127.4 (d, J = 5.3 Hz), 125.5, 125.0, 123.5, 122.1, 121.3 (d, J = 5.7 Hz), 120.8 (d, J = 18.2 Hz), 98.2 (d, J = 27.3 Hz), 57.2, 31.7, 26.2, 25.6; ^{19}F NMR (376 MHz, $CDCl_3$): δ -126.54 (s, 1F); HRMS (ESI, m/z) calcd. For $C_{22}H_{21}FN_3$ $[M+H]^+$: 346.1720; found: 346.1719.

3-cyclohexyl-5-methyl-2-(pyridin-2-yl)-3H-naphtho[1,2-d]imidazole (5p)

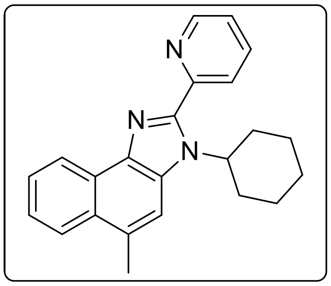

The general procedure for imidazole was followed. Column chromatography (SiO_2, eluting with 90:10 hexane/ethyl acetate) afforded the desired product as creamy white solid (58 mg, 85% yield), mp 132-134 °C.

^{1}H NMR (400 MHz, $CDCl_3$): δ 8.95 (s, 1H), 8.73-8.71 (m, 1H), 8.48 (s, 1H), 8.04 (d, J = 8.4 Hz, 1H), 7.93-7.89 (m, 1H), 7.69-7.65 (m, 2H), 7.58-7.55 (m, 1H), 7.38-7.35 (m, 1H), 5.59-5.50 (m, 1H), 2.81 (s, 3H), 2.40-2.31 (m, 2H), 2.16-2.12 (m, 2H), 1.99-1.96 (m, 2H), 1.83-1.79 (m, 1H), 1.52-1.37 (m, 3H); ^{13}C NMR (100 MHz, $CDCl_3$): δ 151.2, 148.8, 148.0, 137.9, 136.9, 131.1, 130.1, 129.4, 127.3, 126.3, 125.6, 124.7, 123.3, 122.6, 113.9, 57.1, 31.9, 26.3, 25.6, 20.7; HRMS (ESI, m/z) calcd. For $C_{23}H_{24}N_3$ $[M+H]^+$: 342.1970; found: 342.1971.

3-cyclohexyl-9-methyl-2-(pyridin-2-yl)-3H-naphtho[1,2-d]imidazole (5q)

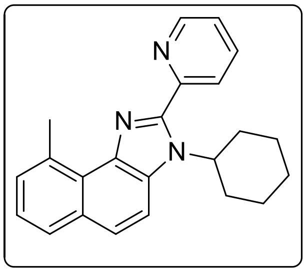

The general procedure for imidazole was followed. Column chromatography (SiO_2, eluting with 90:10 hexane/ethyl acetate) afforded the desired product as a creamy white solid (54.5 mg, 80% yield), mp 140-142 °C.

^{1}H NMR (400 MHz, $CDCl_3$): δ 8.70-8.68 (m, 1H), 8.42 (dt, J_1 = 8.0 Hz, J_2 = 0.8 Hz, 1H), 7.87-7.82 (m, 2H), 7.79-7.76 (m, 1H), 7.67 (d, J = 8.8 Hz, 1H), 7.43-7.36 (m, 2H), 7.32- 7.29 (m, 1H), 5.86-5.78 (m, 1H), 3.29 (s, 3H), 2.43-2.33 (m, 2H), 2.16-2.11 (m, 2H), 1.99- 1.95 (m, 2H), 1.81 (d, J = 11.2 Hz, 1H), 1.55-1.35 (m, 3H); ^{13}C NMR (100 MHz, $CDCl_3$): δ 148.6, 147.3, 136.8, 135.0, 132.53, 132.48, 131.0, 128.5, 126.5, 126.4, 125.7, 124.6, 124.2, 123.2, 113.5, 56.9, 31.7, 26.3, 25.7, 23.8; HRMS (ESI, m/z) calcd. For $C_{23}H_{24}N_3$ $[M+H]^+$: 342.1970; found: 342.1973.

3-cyclohexyl-5-(1H-pyrazol-1-yl)-2-(pyridin-2-yl)-3H-naphtho[1,2-d]imidazole (5r)

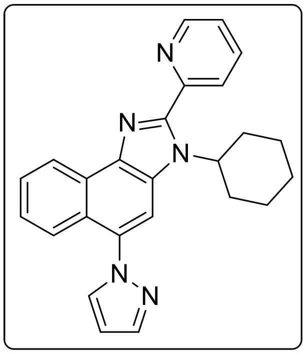

The general procedure for imidazole was followed. Column chromatography (SiO_2, eluting with 80:20 hexane/ethyl acetate) afforded the desired product as a creamy white solid (43 mg, 55% yield), mp 174-176 °C.

^{1}H NMR (400 MHz, $CDCl_3$): δ 8.85 (d, J = 8.0 Hz, 1H), 8.73-8.71 (m, 1H), 8.38 (d, J = 8.0 Hz, 1H), 7.95 (s, 1H), 7.92-7.84 (m, 3H), 7.68-7.64 (m, 1H), 7.60 (d, J = 8.0 Hz, 1H), 7.50-7.46 (m, 1H), 7.39-7.35 (m, 1H), 6.58 (t, J = 2.0 Hz, 1H), 5.62-5.54 (m, 1H), 2.34-2.23 (m, 2H), 2.17-2.13 (m, 2H), 1.96-1.91 (m, 2H), 1.76 (d, J = 12.4 Hz, 1H), 1.50-1.40 (m, 2H), 1.37-1.29 (m, 1H); ^{13}C NMR (100 MHz, $CDCl_3$): δ 149.9, 149.0, 140.8, 137.1, 133.5, 132.5, 129.9, 127.3, 127.2, 126.6, 126.0, 125.9, 123.8, 123.6, 122.5, 112.1, 106.5, 57.5, 32.0, 26.2, 25.4; HRMS (ESI, m/z) calcd. For $C_{25}H_{24}N_5$ $[M+H]^+$: 394.2032; found: 394.2036.

5-(1H-benzo[d][1,2,3]triazol-1-yl)-3-cyclohexyl-2-(pyridin-2-yl)-3H-naphtho[1,2-d]imidazole (5s)

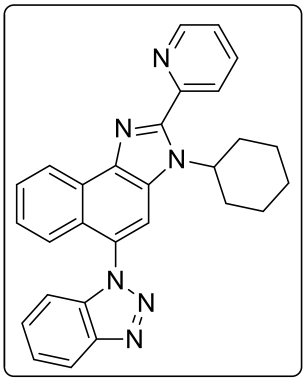

The general procedure for imidazole was followed. Column chromatography (SiO_2, eluting with 80:20 hexane/ethyl acetate) afforded the desired product as a creamy white solid (43.5 mg, 49% yield), mp 214-216 °C.

^{1}H NMR (400 MHz, $CDCl_3$): δ 8.90 (d, J = 8.0 Hz, 1H), 8.74-8.73 (m, 1H), 8.40 (d, J = 7.6 Hz, 1H), 8.26-8.21 (m, 1H), 8.07 (s, 1H), 7.91 (td, J_1 = 8.0 Hz, J_2 = 2.0 Hz, 1H), 7.71-7.67 (m, 1H), 7.51-7.46 (m, 2H), 7.45-7.37 (m, 2H), 7.30-7.27 (m, 2H), 5.67-5.59 (m, 1H), 2.29-2.16 (m, 4H),

1.91 (d, J = 13.2 Hz, 2H), 1.72 (d, J = 12.8 Hz, 1H), 1.49-1.38 (m, 2H), 1.31-1.20 (m, 1H); ^{13}C NMR (100 MHz, $CDCl_3$): δ 150.6, 150.5, 149.0, 145.8, 140.3, 137.1, 135.4, 130.0, 128.3, 128.1, 127.6, 127.5, 126.2, 125.96, 125.92, 124.4, 123.9, 123.2, 122.8, 120.3, 113.6, 110.7, 57.6, 32.1, 26.1, 25.3; HRMS (ESI, m/z) calcd. For $C_{28}H_{25}N_6$ $[M+H]^+$: 445.2141; found: 445.2140.

3-cyclohexyl-1H-naphtho[1,2-d]imidazol-2(3H)-one (8a)

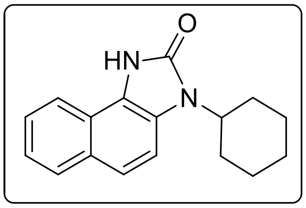

Column chromatography (SiO_2, eluting with 75:25 hexane/ethyl acetate) afforded the desired product as a light pink solid.

^{1}H NMR (400 MHz, $CDCl_3$): δ 11.40 (s, 1H), 8.04 (d, J = 8.0 Hz, 1H) 7.85 (d, J = 8.4 Hz, 1H), 7.58-7.53 (m, 2H), 7.50 (d, J = 8.8 Hz, 1H), 7.41-7.37 (m, 1H), 4.51-4.24 (m, 1H), 2.28-2.18 (m, 2H), 1.99-1.94 (m, 4H), 1.81 (d, J = 12.8 Hz, 1H), 1.59-1.48 (m, 2H), 1.39-1.32 (m, 1H); ^{13}C NMR (100 MHz, $CDCl_3$): δ 155.4, 129.1, 128.6, 126.5, 124.9, 124.2, 122.5, 121.4, 120.6, 119.9, 110.9, 53.1, 30.8, 26.1, 25.5; HRMS (ESI, m/z) calcd. For $C_{17}H_{19}N_2O$ $[M+H]^+$: 267.1497; found: 267.1506.

3-(*tert*-butyl)-1H-naphtho[1,2-d]imidazol-2(3H)-one (8b)

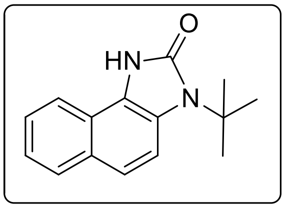

Column chromatography (SiO_2, eluting with 75:25 hexane/ethyl acetate) afforded the desired product as a white solid.

^{1}H NMR (400 MHz, $CDCl_3$): δ 11.71 (s, 1H), 8.05 (d, J = 8.4 Hz, 1H) 7.83 (d, J = 8.4 Hz, 1H), 7.69 (d, J = 8.8 Hz, 1H), 7.54-7-49 (m, 2H), 7.41-7.39 (m, 1H), 1.91 (s, 9H); ^{13}C NMR (100 MHz, $CDCl_3$): δ 156.3, 128.8, 128.3, 126.2, 125.7, 124.3, 123.2, 120.7, 120.6, 119.7, 113.3, 58.6, 29.8; HRMS (ESI, m/z) calcd. For $C_{15}H_{17}N_2O$ $[M+H]^+$: 241.1341; found: 241.1344.

References.

1) Nemchik, A.; Badescu, V., *Tetrahedron*, 2003, **59**, 4315-4325.
2) Phanstiel, O.; Wang, Q. X.; Powell, D. H.; Ospina, M. P.; Leeson, B. A., *J. Org. Chem.*, 1999, **64**, 803-806.
3) Xue, Y.; Fan, Z.; Jiang, X.; Wu, K.; Wang, M.; Ding, C.; Yao, Q.; Zhang, A., *Eur. J. Org. Chem.*, 2014, **2014**, 7481-7488.
4) Selvam, J. J. P.; Suresh, V.; Rajesh, K.; Reddy, S. R.; Venkateswarlu, Y., *Tet. Lett.*, 2006, **47**, 2507-2509.
5) Beesu, M.; Malladi, S. S.; Fox, L. M.; Jones, C. D.; Dixit, A.; David, S. A., *J. Med. Chem.*, 2014, **57**, 7325-7341.
6) Smout, V.; Peschiulli, A.; Verbeeck, S.; Mitchell, E. A.; Herrebout, W.; Bultinck, P.; Vande Velde, C. M. L.; Berthelot, D.; Meerpoel, L.; Maes, B. U. W., *J. Org. Chem.*, 2013, **78**, 9803-9814.

^{1}H NMR and ^{13}C NMR Spectra:

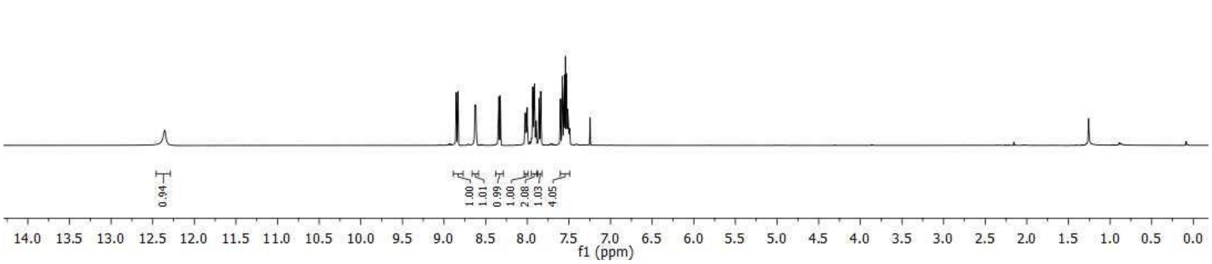

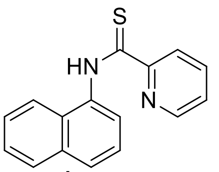

4a

400 MHz, $CDCl_3$

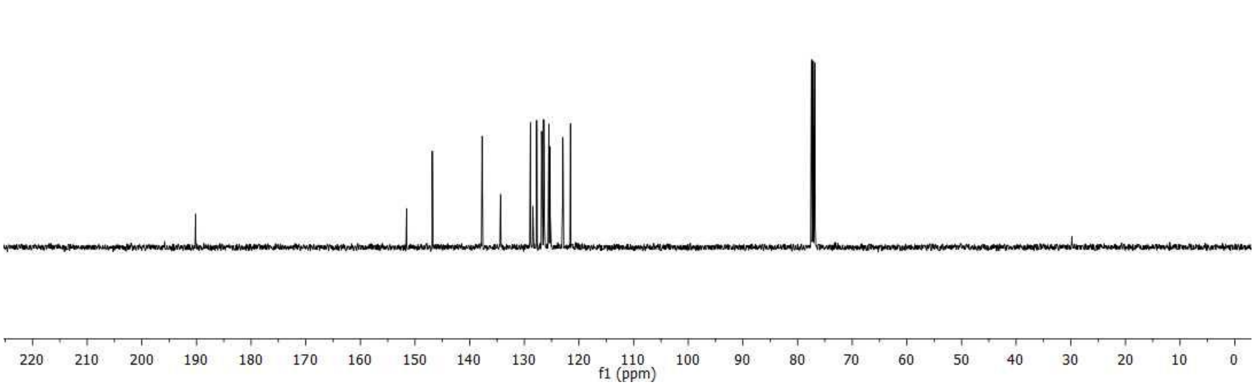

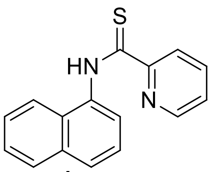

4a

100 MHz, $CDCl_3$

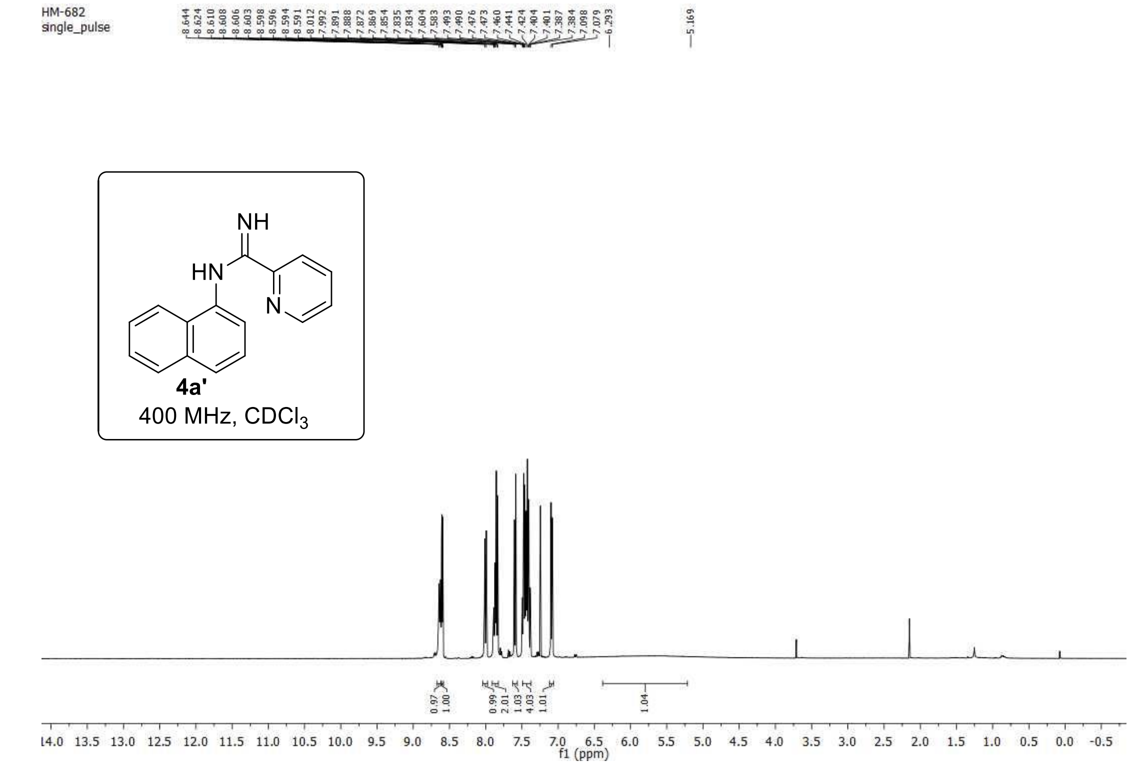
HM-682
single_pulse
8.644
8.624
8.610
8.608
8.606
8.603
8.598
8.596
8.594
8.591
8.012
7.992
7.891
7.888
7.872
7.869
7.854
7.835
7.834
7.604
7.583
7.493
7.490
7.476
7.473
7.460
7.441
7.424
7.404
7.401
7.387
7.384
7.098
7.079
6.293
5.169
NH
HN
N
4a'
400 MHz, CDCl3
0.97
1.00
0.99
2.01
1.03
4.03
1.01
1.04
f1 (ppm)

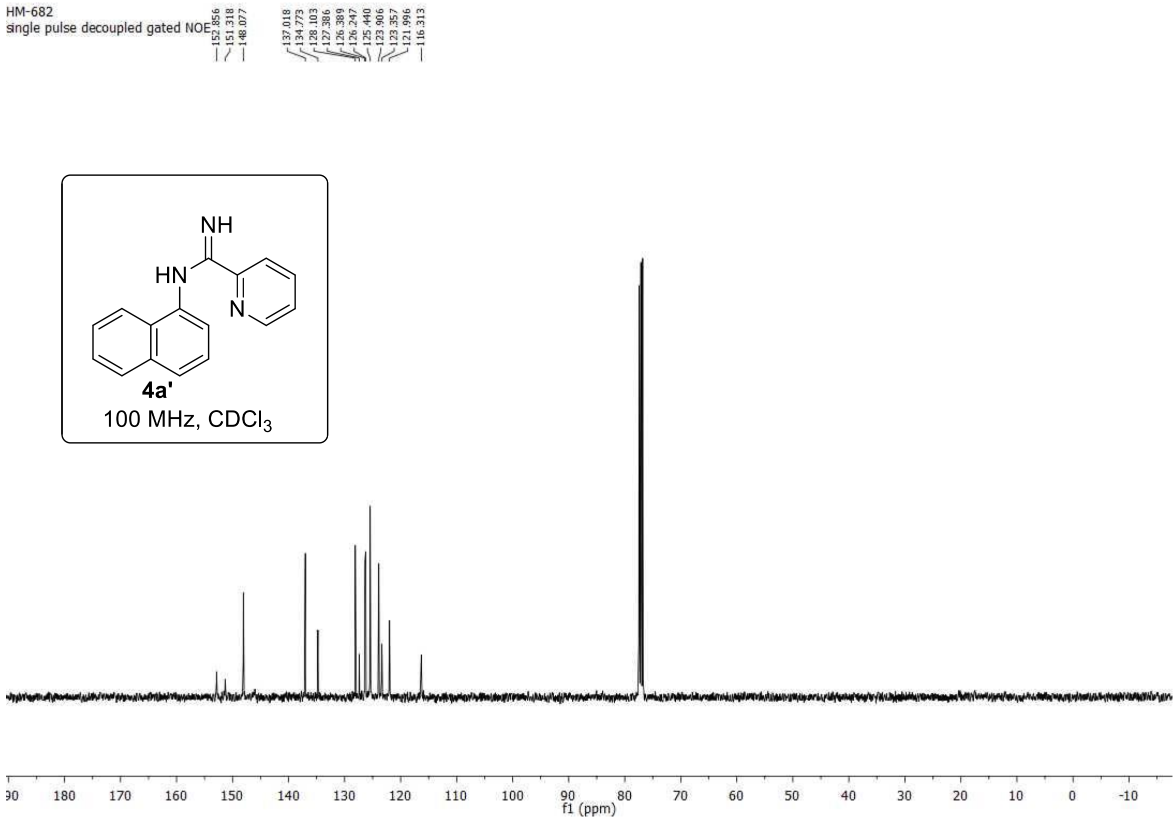
HM-682
single pulse decoupled gated NOE
152.856
151.318
148.077
137.018
134.773
128.103
127.386
126.389
126.247
125.440
123.906
123.357
121.996
116.313
NH
HN
N
4a'
100 MHz, CDCl3
f1 (ppm)

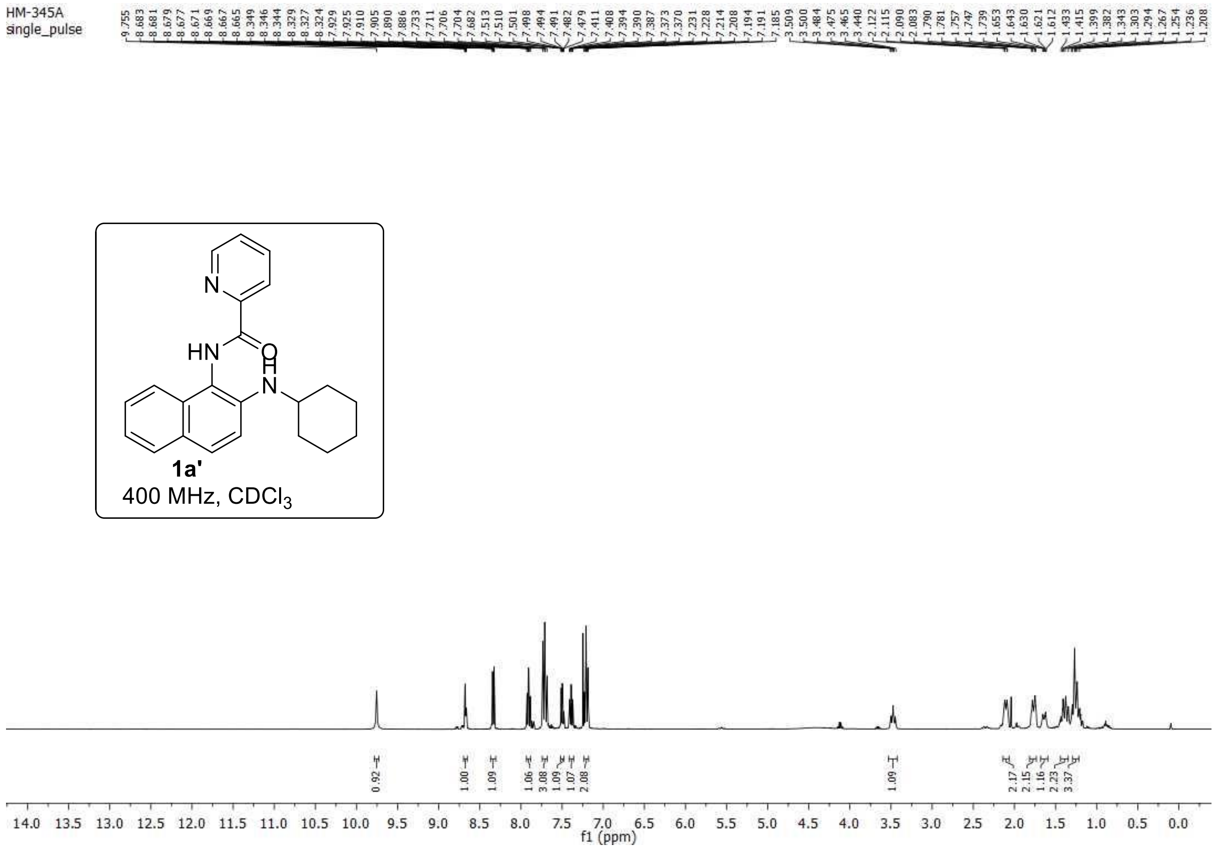
HM-345A
single_pulse
9.755 8.683 8.681 8.679 8.677 8.671 8.669 8.667 8.665 8.349 8.346 8.344 8.329 8.327 8.324 7.929 7.925 7.910 7.905 7.890 7.886 7.733 7.711 7.706 7.704 7.682 7.513 7.510 7.501 7.498 7.494 7.491 7.482 7.479 7.411 7.408 7.394 7.390 7.387 7.373 7.370 7.231 7.228 7.214 7.208 7.194 7.191 7.185 3.509 3.500 3.484 3.475 3.465 3.440 2.122 2.115 2.090 2.083 1.790 1.781 1.757 1.747 1.739 1.653 1.643 1.630 1.621 1.612 1.433 1.415 1.399 1.382 1.343 1.303 1.294 1.267 1.254 1.236 1.208
1a'
400 MHz, CDCl3
0.92 1.00 1.09 1.06 3.08 1.09 1.07 2.08 1.09 2.17 2.15 1.16 2.23 3.37
f1 (ppm)

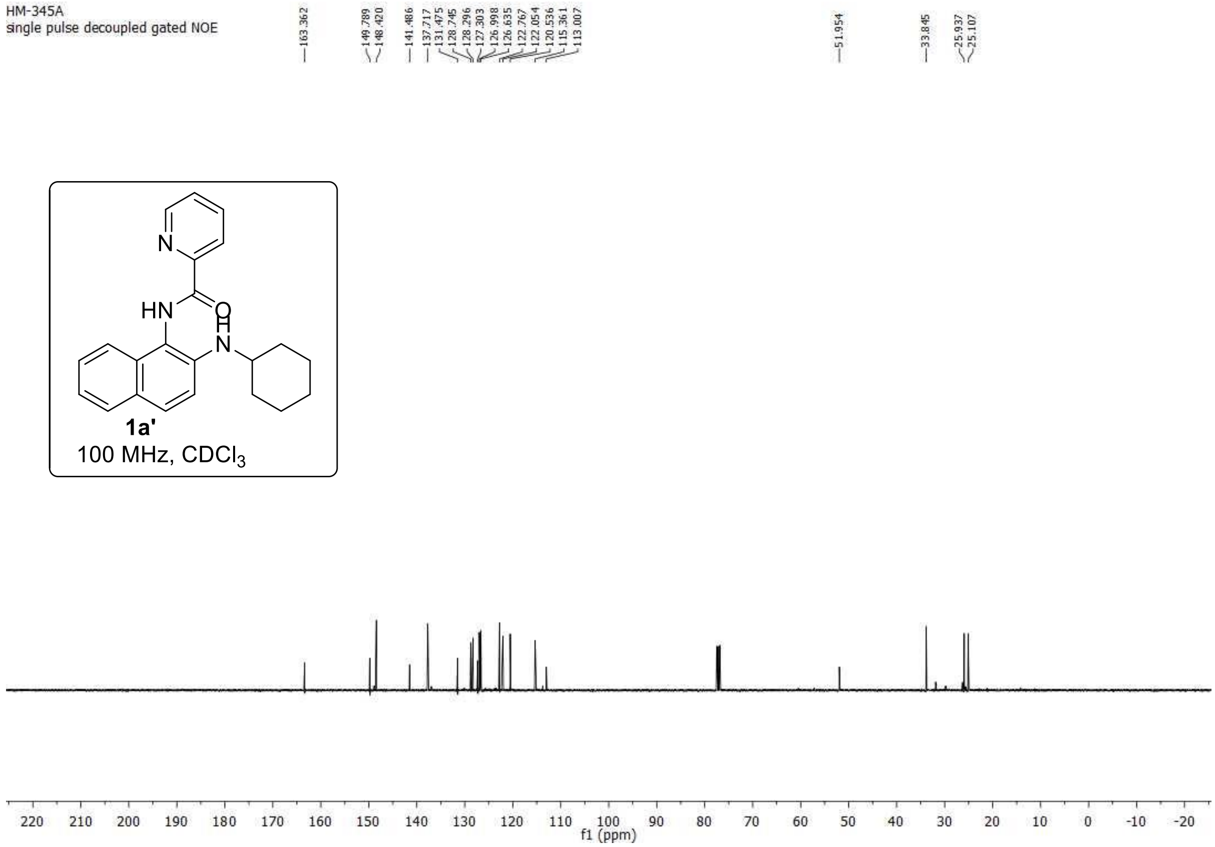
HM-345A
single pulse decoupled gated NOE
163.362 149.789 148.420 141.486 137.717 131.475 128.745 128.296 127.303 126.998 126.635 122.767 122.054 120.536 115.361 113.007 51.954 33.845 25.937 25.107
1a'
100 MHz, CDCl3
f1 (ppm)

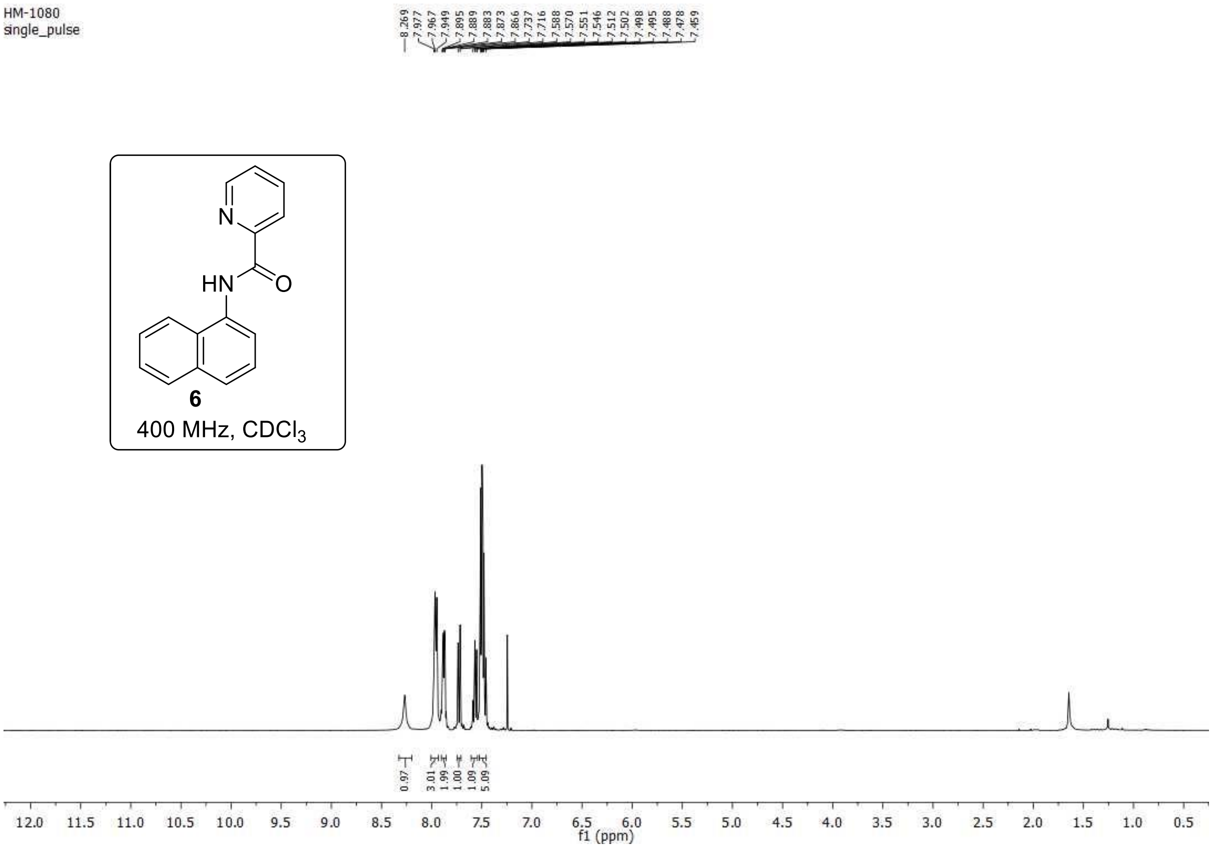
HM-1080
single_pulse
8.269 7.977 7.967 7.949 7.895 7.889 7.883 7.873 7.866 7.737 7.716 7.588 7.570 7.551 7.546 7.512 7.502 7.498 7.495 7.488 7.478 7.459
N
HN
O
6
400 MHz, CDCl3
0.97 3.01 1.99 1.00 1.09 5.09
12.0 11.5 11.0 10.5 10.0 9.5 9.0 8.5 8.0 7.5 7.0 6.5 6.0 5.5 5.0 4.5 4.0 3.5 3.0 2.5 2.0 1.5 1.0 0.5
f1 (ppm)

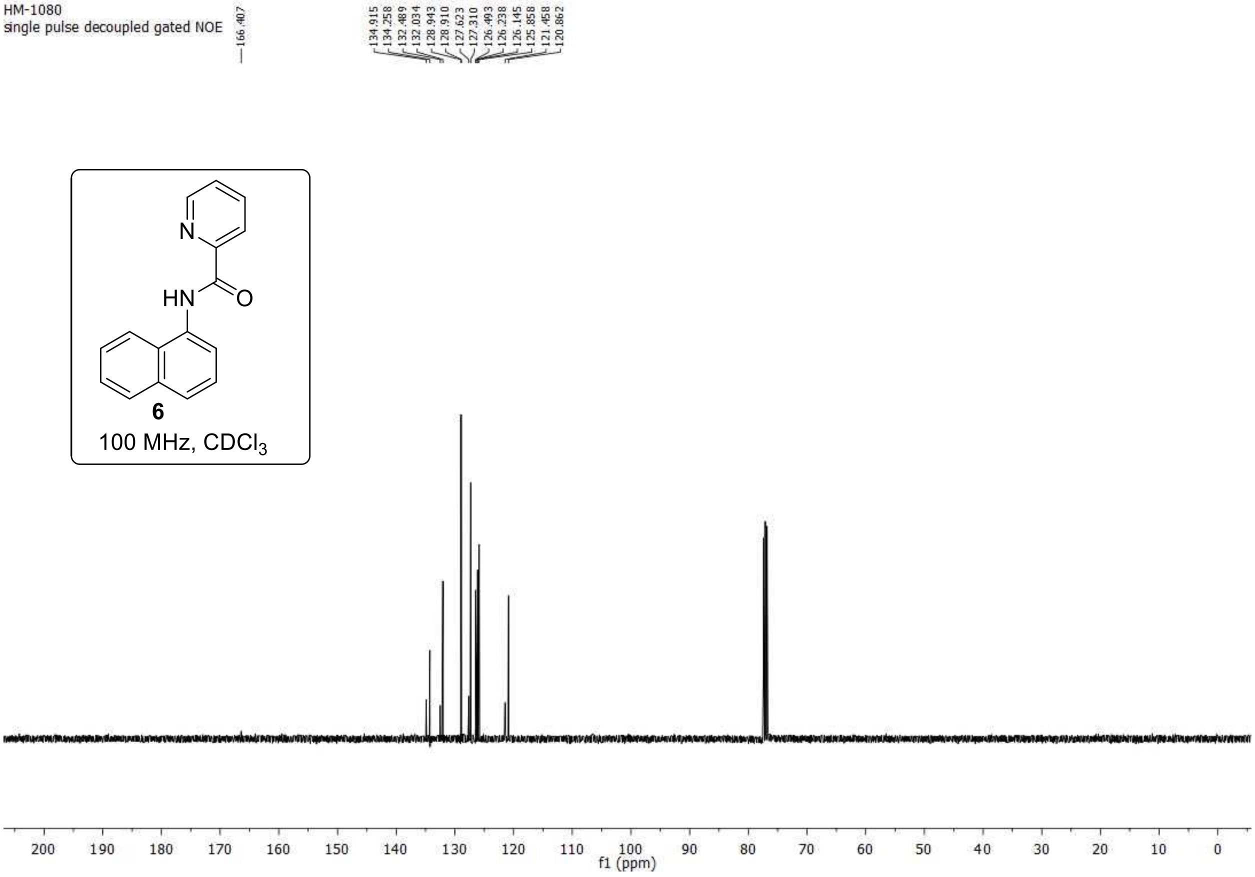
HM-1080
single pulse decoupled gated NOE
166.407
134.915 134.258 132.489 132.034 128.943 128.910 127.623 127.310 126.493 126.238 126.145 125.858 121.458 120.862
N
HN
O
6
100 MHz, CDCl3
200 190 180 170 160 150 140 130 120 110 100 90 80 70 60 50 40 30 20 10 0
f1 (ppm)

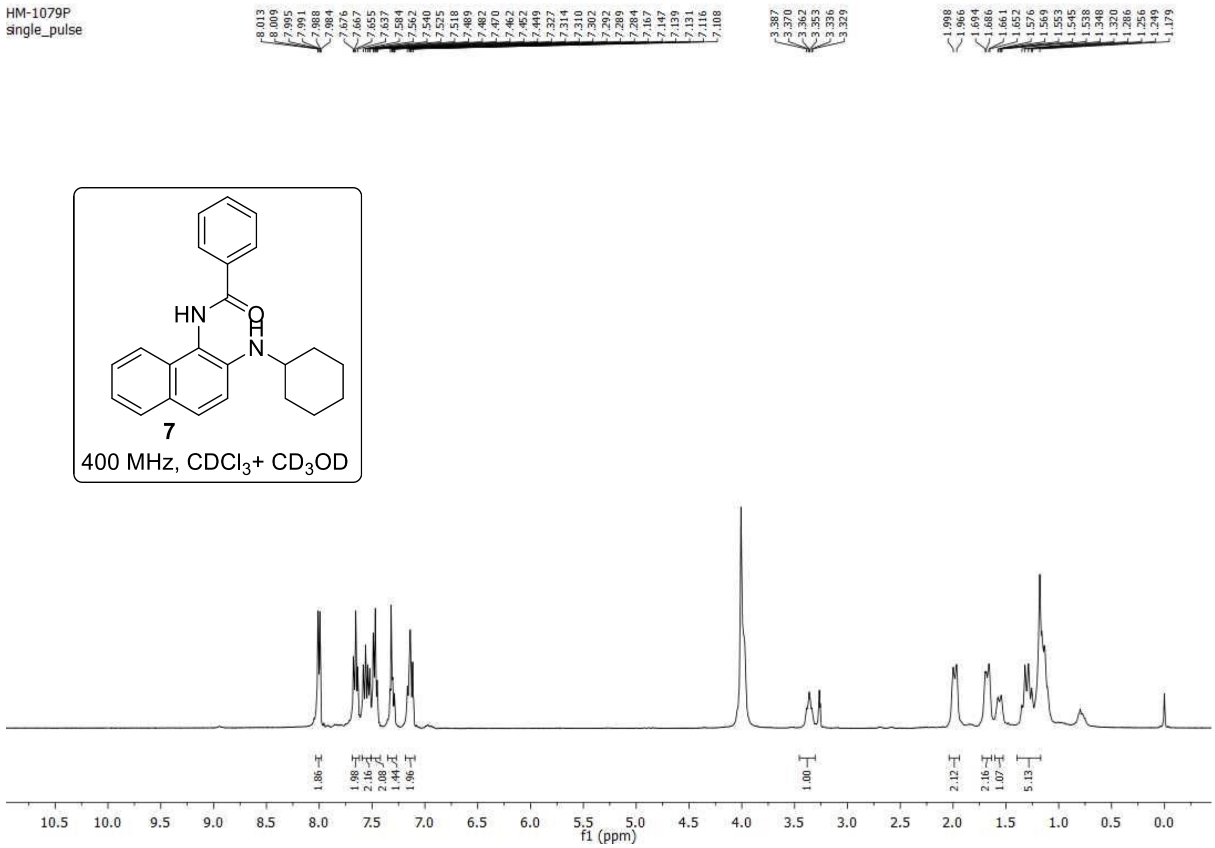

HM-1079P
single_pulse
8.013
8.009
7.995
7.991
7.988
7.984
7.676
7.667
7.655
7.637
7.584
7.562
7.540
7.525
7.518
7.489
7.482
7.470
7.462
7.452
7.449
7.327
7.314
7.310
7.302
7.292
7.289
7.284
7.167
7.147
7.139
7.131
7.116
7.108
3.387
3.370
3.362
3.353
3.336
3.329
1.998
1.966
1.694
1.686
1.661
1.652
1.576
1.569
1.553
1.545
1.538
1.348
1.320
1.286
1.256
1.249
1.179
HN
O
H
N
7
400 MHz, $CDCl_3$+ CD_3OD
1.86
1.98
2.16
2.08
1.44
1.96
1.00
2.12
2.16
1.07
5.13
10.5
10.0
9.5
9.0
8.5
8.0
7.5
7.0
6.5
6.0
5.5
5.0
4.5
4.0
3.5
3.0
2.5
2.0
1.5
1.0
0.5
0.0
f1 (ppm)

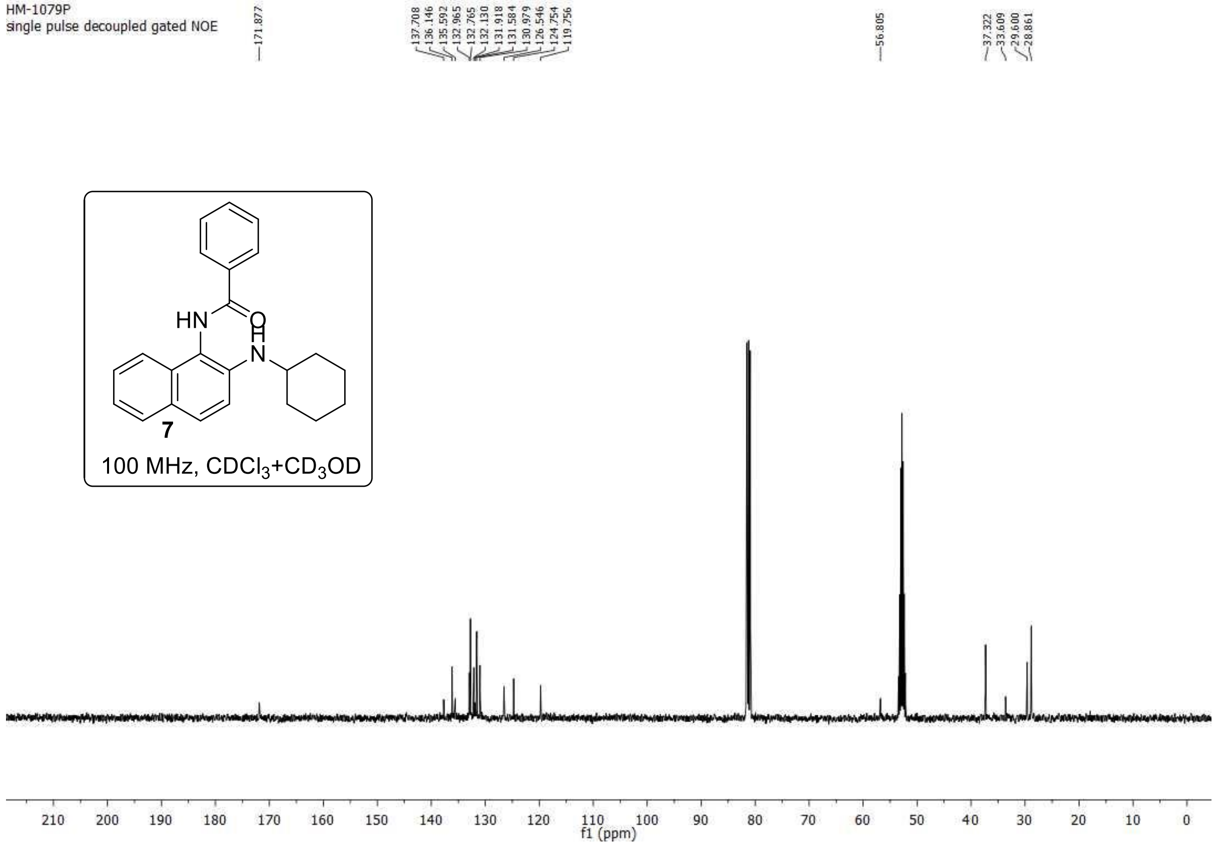

HM-1079P
single pulse decoupled gated NOE
171.877
137.708
136.146
135.592
132.965
132.765
132.130
131.918
131.584
130.979
126.546
124.754
119.756
56.805
37.322
33.609
29.600
28.861
HN
O
H
N
7
100 MHz, $CDCl_3$+CD_3OD
210
200
190
180
170
160
150
140
130
120
110
100
90
80
70
60
50
40
30
20
10
0
f1 (ppm)

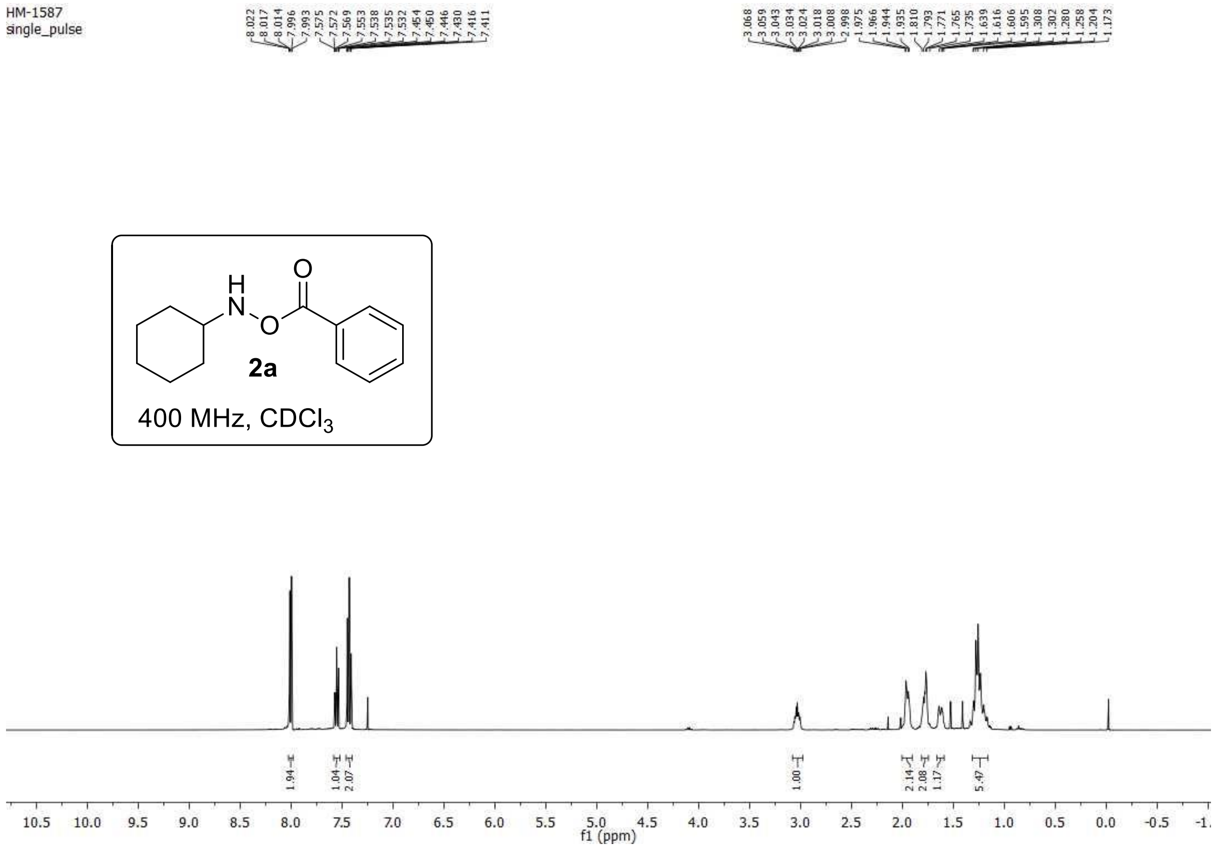
HM-1587
single_pulse
8.022
8.017
8.014
7.996
7.993
7.575
7.572
7.569
7.553
7.538
7.535
7.532
7.454
7.450
7.446
7.430
7.416
7.411
3.068
3.059
3.043
3.034
3.024
3.018
3.008
2.998
1.975
1.966
1.944
1.935
1.810
1.793
1.771
1.765
1.735
1.639
1.616
1.606
1.595
1.308
1.302
1.280
1.258
1.204
1.173
2a
400 MHz, CDCl3
1.94
1.04
2.07
1.00
2.14
2.08
1.17
5.47
f1 (ppm)

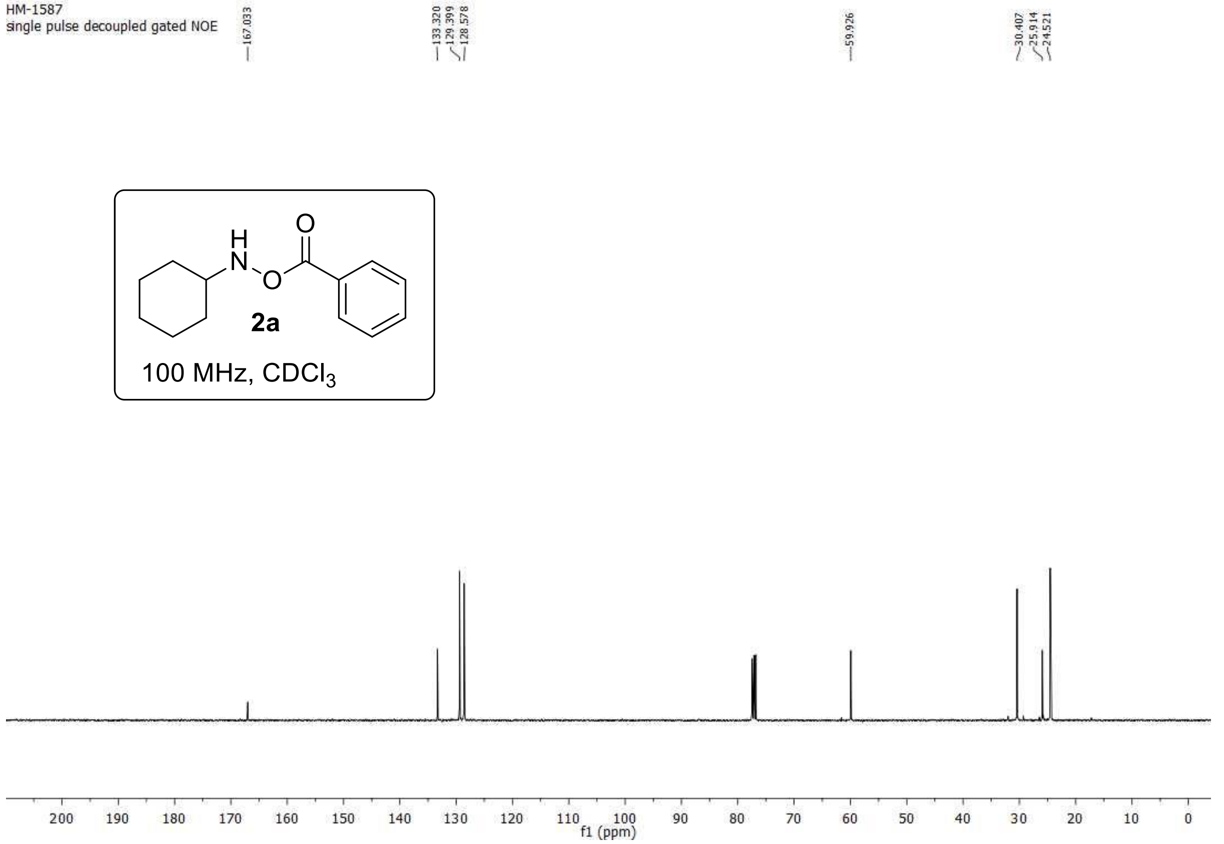
HM-1587
single pulse decoupled gated NOE
167.033
133.320
129.399
128.578
59.926
30.407
25.914
24.521
2a
100 MHz, CDCl3
f1 (ppm)

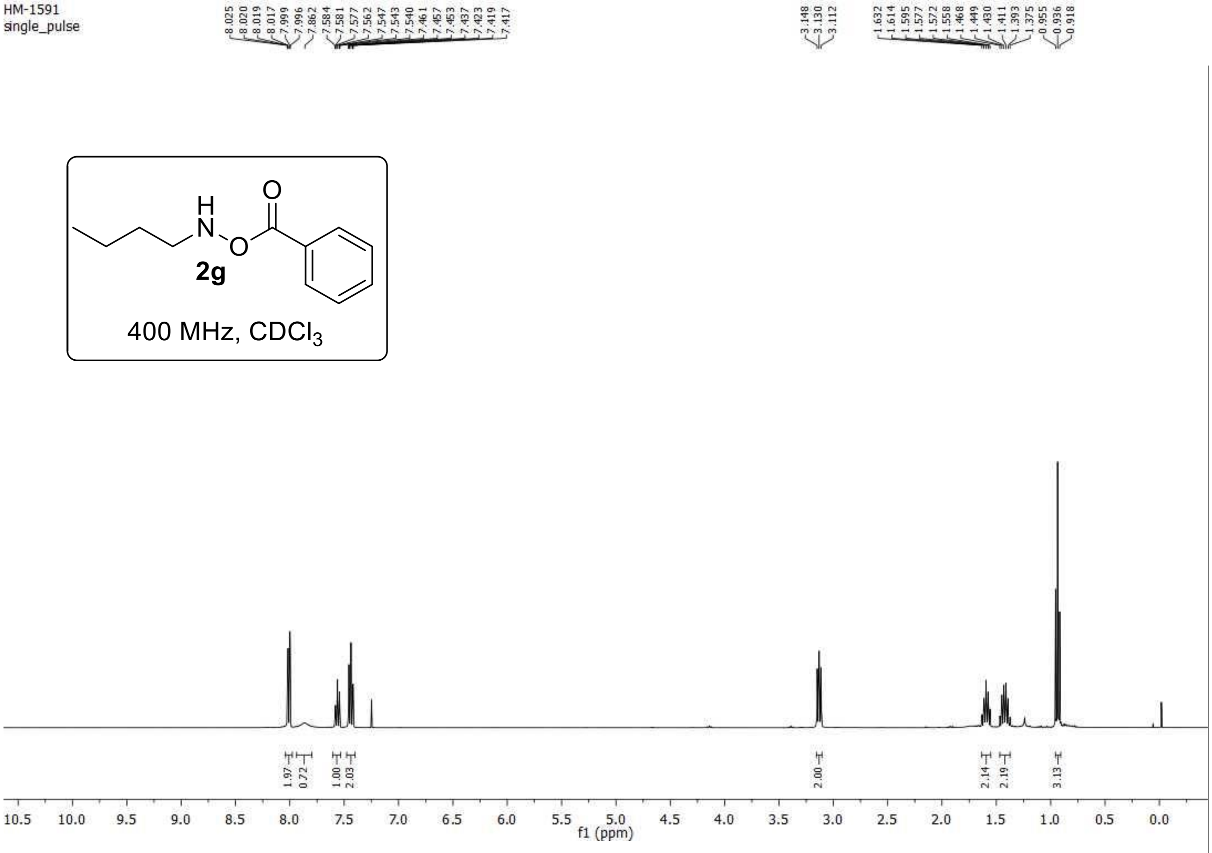
HM-1591
single_pulse
8.025 8.020 8.019 8.017 7.999 7.996 7.862 7.584 7.581 7.577 7.562 7.547 7.543 7.540 7.461 7.457 7.453 7.437 7.423 7.419 7.417
3.148 3.130 3.112
1.632 1.614 1.595 1.577 1.572 1.558 1.468 1.449 1.430 1.411 1.393 1.375 0.955 0.936 0.918
2g
400 MHz, CDCl3
1.97 0.72 1.00 2.03 2.00 2.14 2.19 3.13
f1 (ppm)

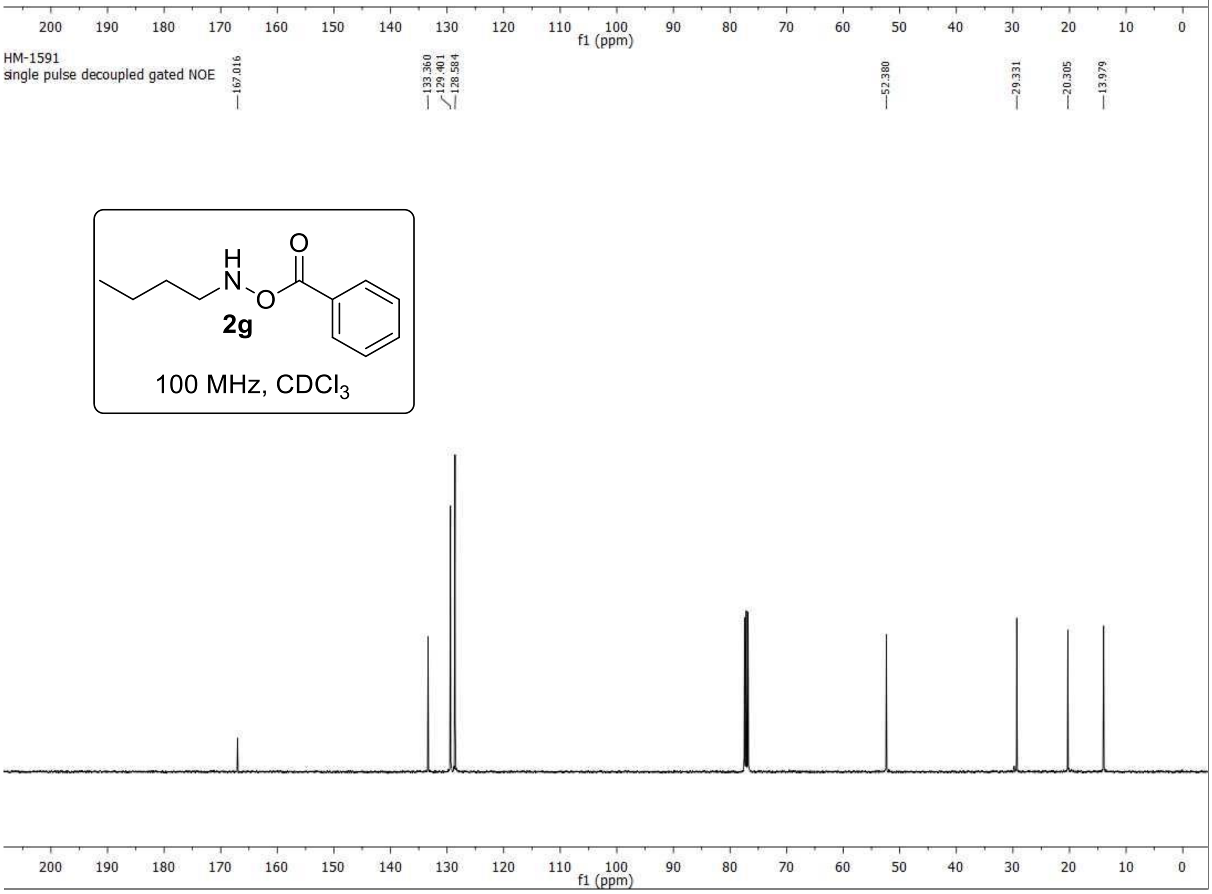
f1 (ppm)
HM-1591
single pulse decoupled gated NOE
167.016
133.360 129.401 128.584
52.380
29.331
20.305
13.979
2g
100 MHz, CDCl3
f1 (ppm)

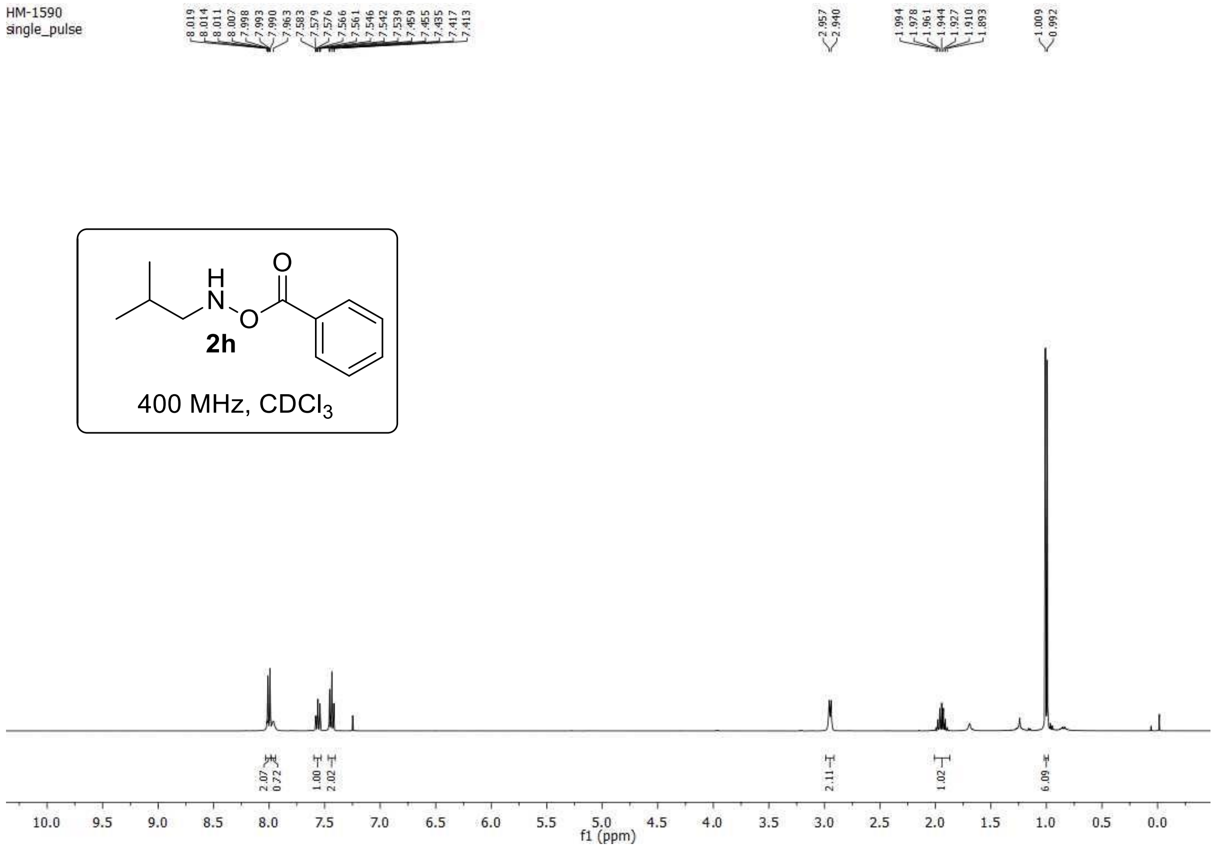
HM-1590
single_pulse
8.019
8.014
8.011
8.007
7.998
7.993
7.990
7.963
7.583
7.579
7.576
7.566
7.561
7.546
7.542
7.539
7.459
7.455
7.435
7.417
7.413
2.957
2.940
1.994
1.978
1.961
1.944
1.927
1.910
1.893
1.009
0.992
H
N
O
O
2h
400 MHz, $CDCl_3$
2.07
0.72
1.00
2.02
2.11
1.02
6.09
10.0
9.5
9.0
8.5
8.0
7.5
7.0
6.5
6.0
5.5
5.0
4.5
4.0
3.5
3.0
2.5
2.0
1.5
1.0
0.5
0.0
f1 (ppm)

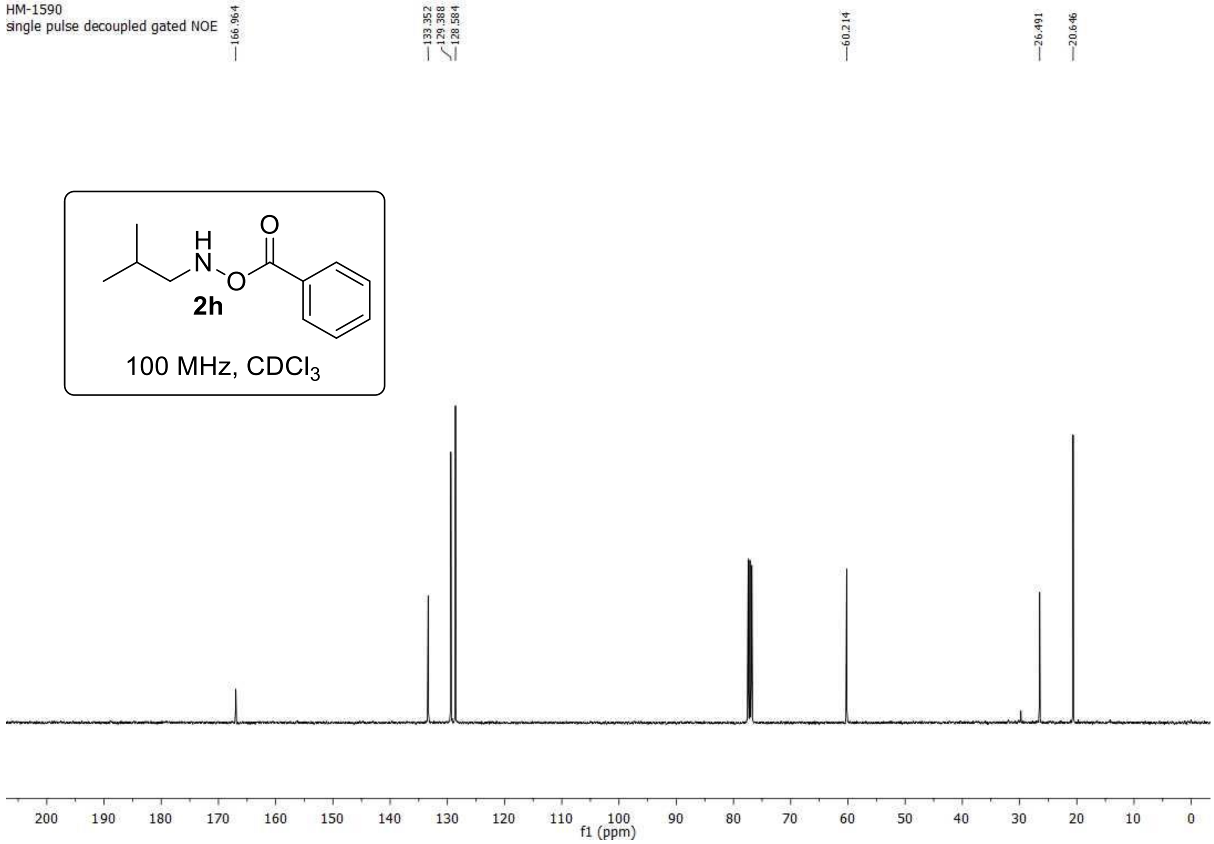
HM-1590
single pulse decoupled gated NOE
166.964
133.352
129.388
128.584
60.214
26.491
20.646
H
N
O
O
2h
100 MHz, $CDCl_3$
200
190
180
170
160
150
140
130
120
110
100
90
80
70
60
50
40
30
20
10
0
f1 (ppm)

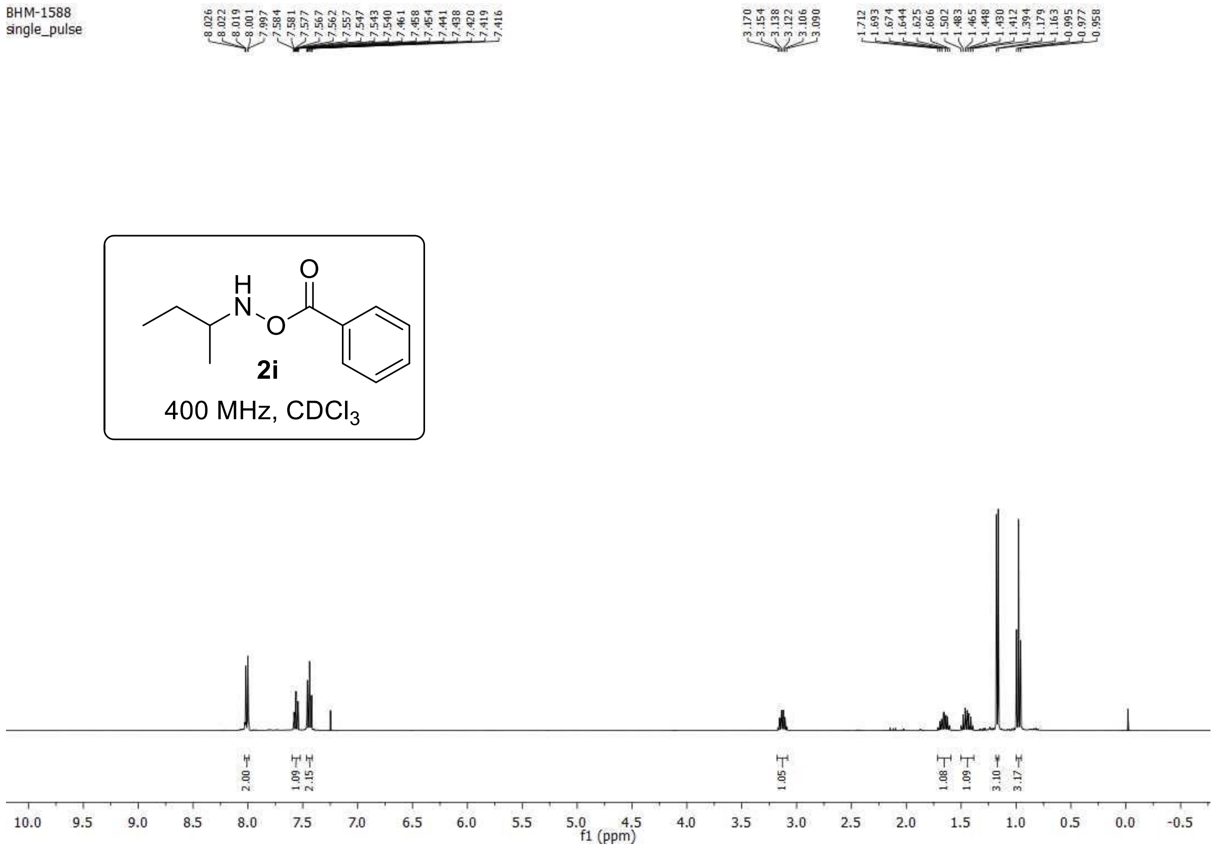

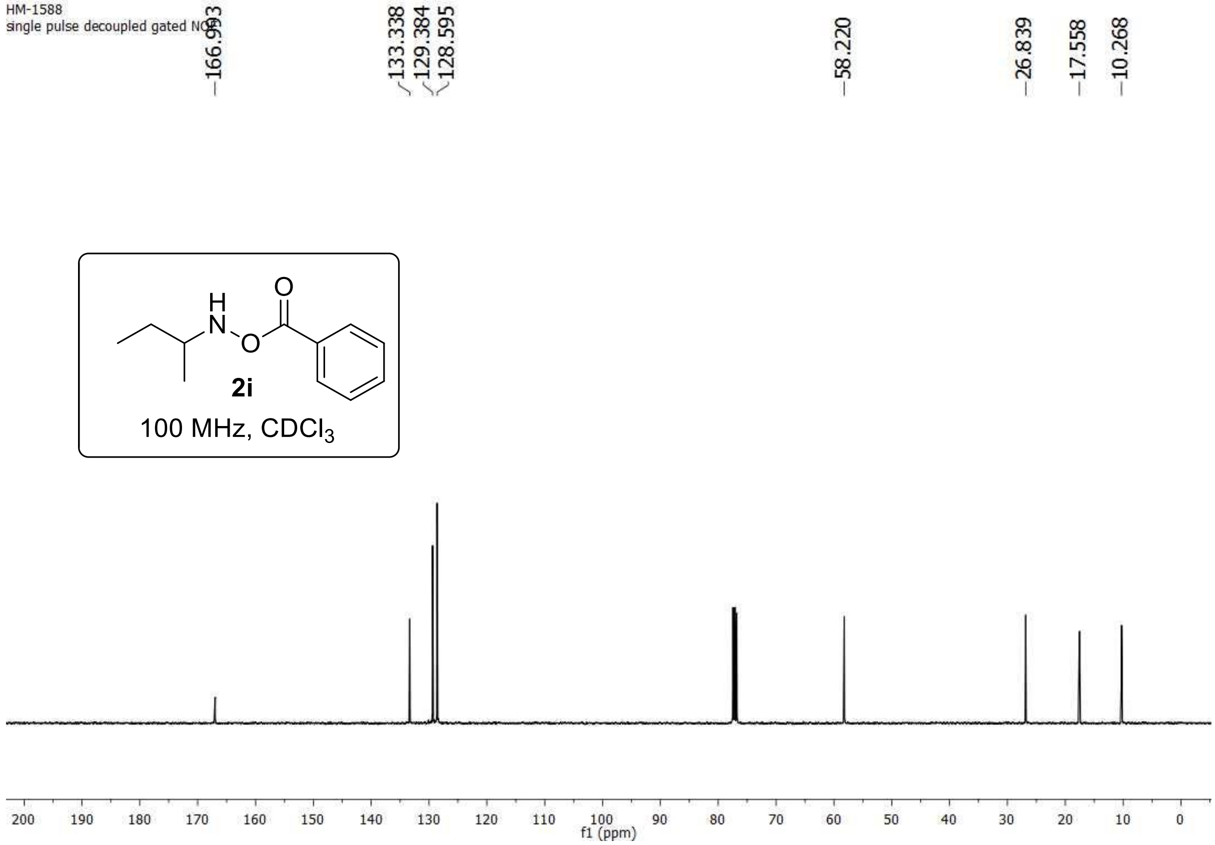

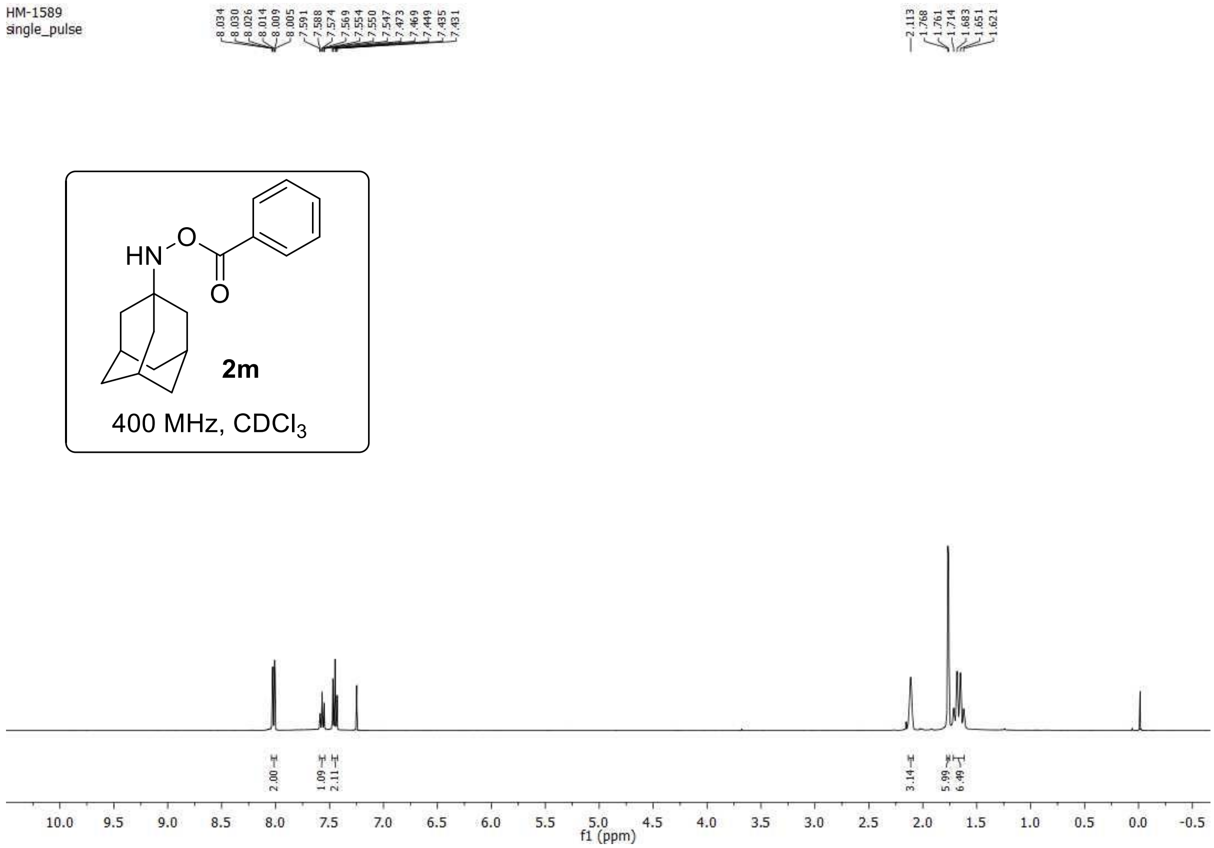
HM-1589
single_pulse
8.034
8.030
8.026
8.014
8.009
8.005
7.591
7.588
7.574
7.569
7.554
7.550
7.547
7.473
7.469
7.449
7.435
7.431
2.113
1.768
1.761
1.714
1.683
1.651
1.621
HN
O
O
2m
400 MHz, $CDCl_3$
2.00
1.09
2.11
3.14
5.99
6.49
10.0
9.5
9.0
8.5
8.0
7.5
7.0
6.5
6.0
5.5
5.0
4.5
4.0
3.5
3.0
2.5
2.0
1.5
1.0
0.5
0.0
-0.5
f1 (ppm)

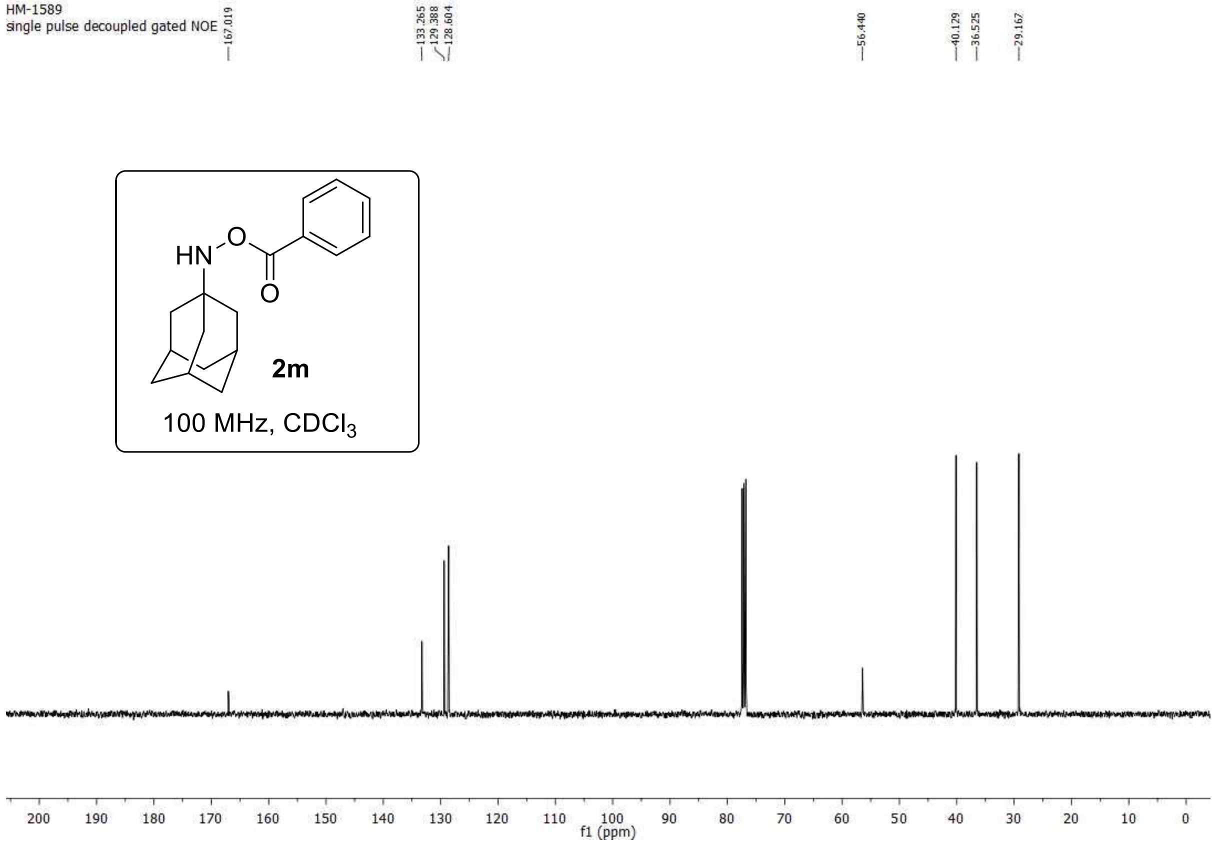
HM-1589
single pulse decoupled gated NOE
167.019
133.265
129.388
128.604
56.440
40.129
36.525
29.167
HN
O
O
2m
100 MHz, $CDCl_3$
200
190
180
170
160
150
140
130
120
110
100
90
80
70
60
50
40
30
20
10
0
f1 (ppm)

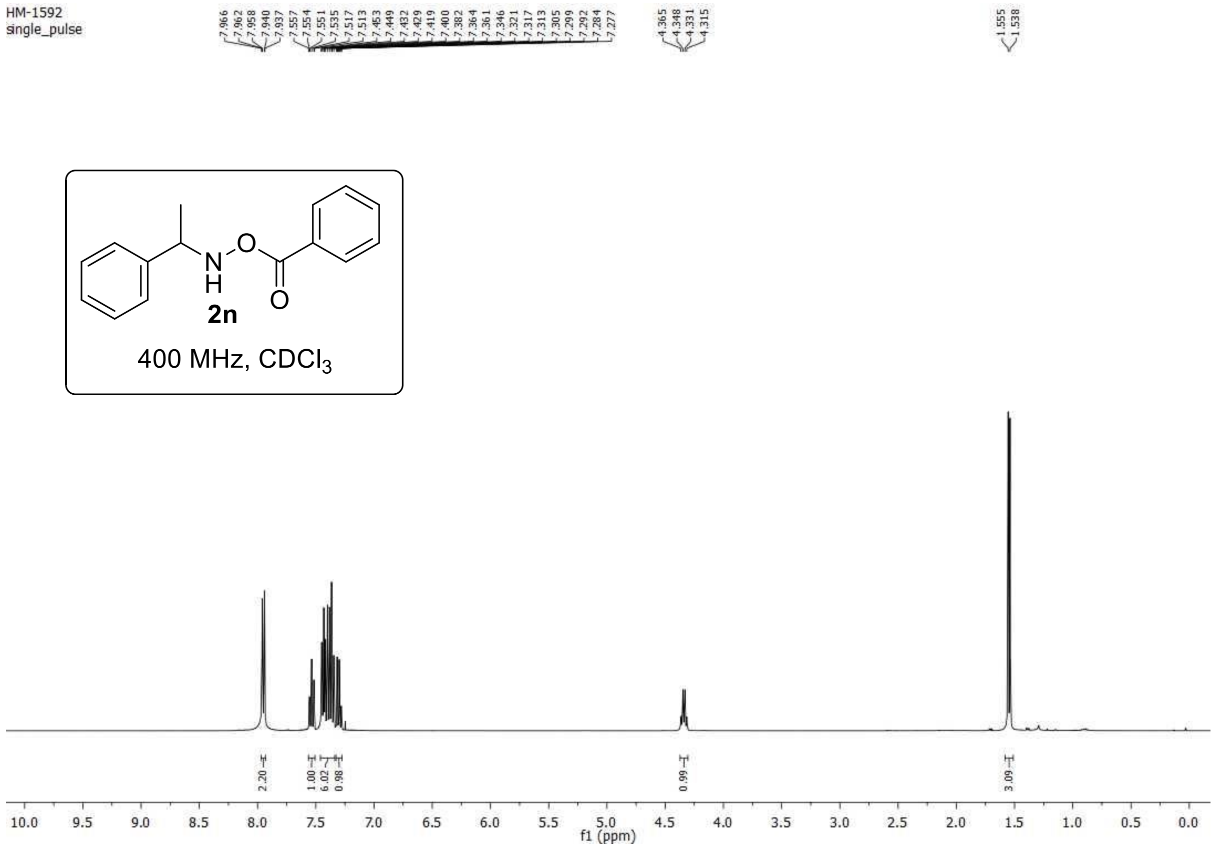
HM-1592
single_pulse
7.966 7.962 7.958 7.940 7.937 7.557 7.554 7.551 7.535 7.517 7.513 7.453 7.449 7.432 7.429 7.419 7.400 7.382 7.364 7.361 7.346 7.321 7.317 7.313 7.305 7.299 7.292 7.284 7.277
4.365 4.348 4.331 4.315
1.555 1.538
2n
400 MHz, $CDCl_3$
2.20 1.00 6.02 0.98 0.99 3.09
10.0 9.5 9.0 8.5 8.0 7.5 7.0 6.5 6.0 5.5 5.0 4.5 4.0 3.5 3.0 2.5 2.0 1.5 1.0 0.5 0.0
f1 (ppm)

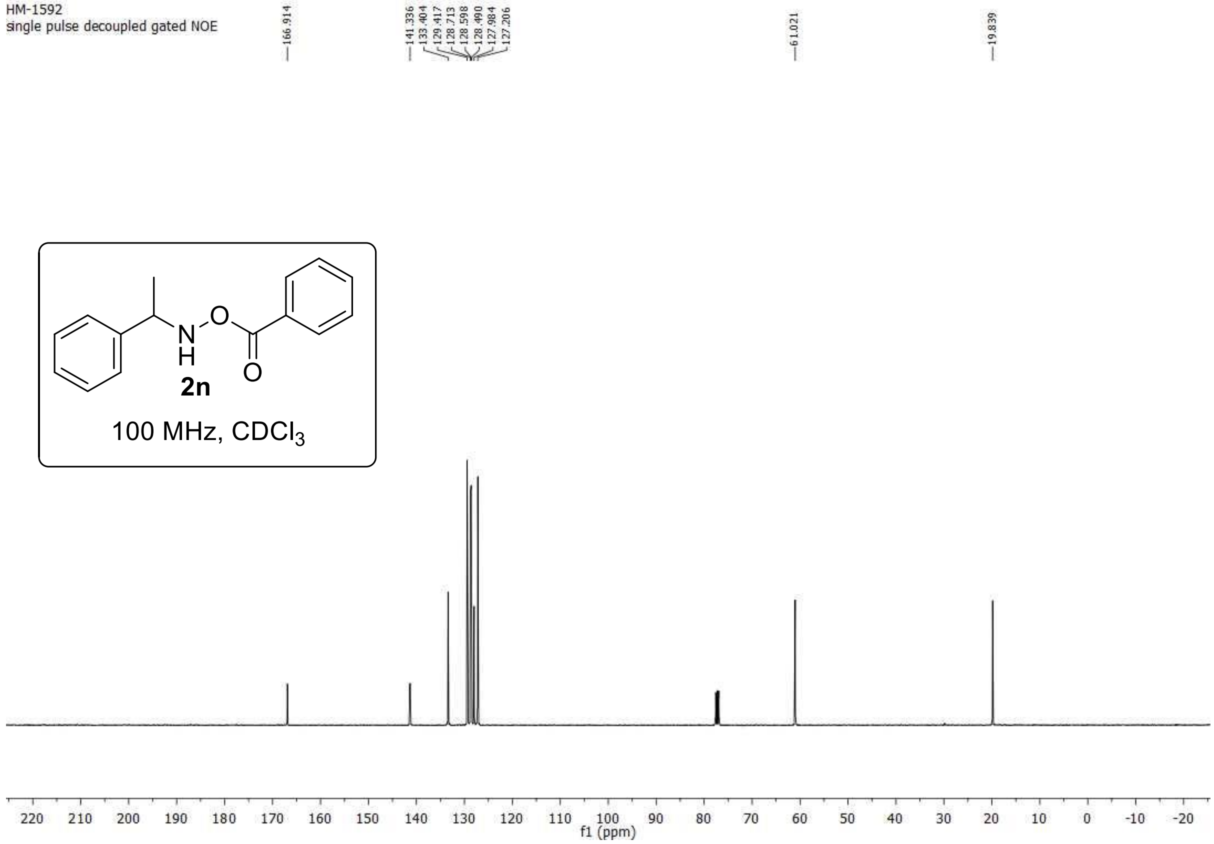
HM-1592
single pulse decoupled gated NOE
166.914
141.336 133.404 129.417 128.713 128.598 128.490 127.984 127.206
61.021
19.839
2n
100 MHz, $CDCl_3$
220 210 200 190 180 170 160 150 140 130 120 110 100 90 80 70 60 50 40 30 20 10 0 -10 -20
f1 (ppm)

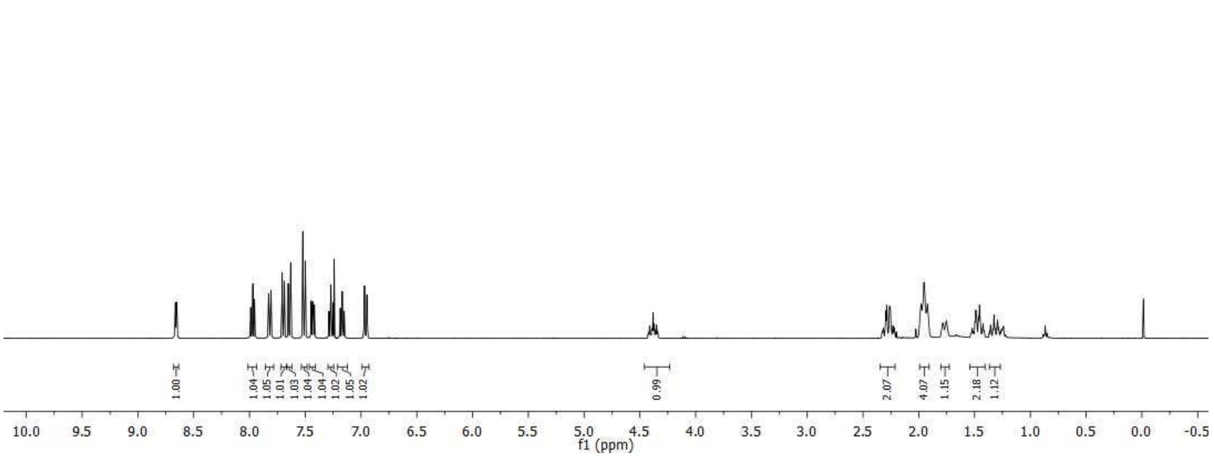

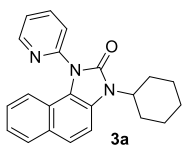

3a

400 MHz, $CDCl_3$

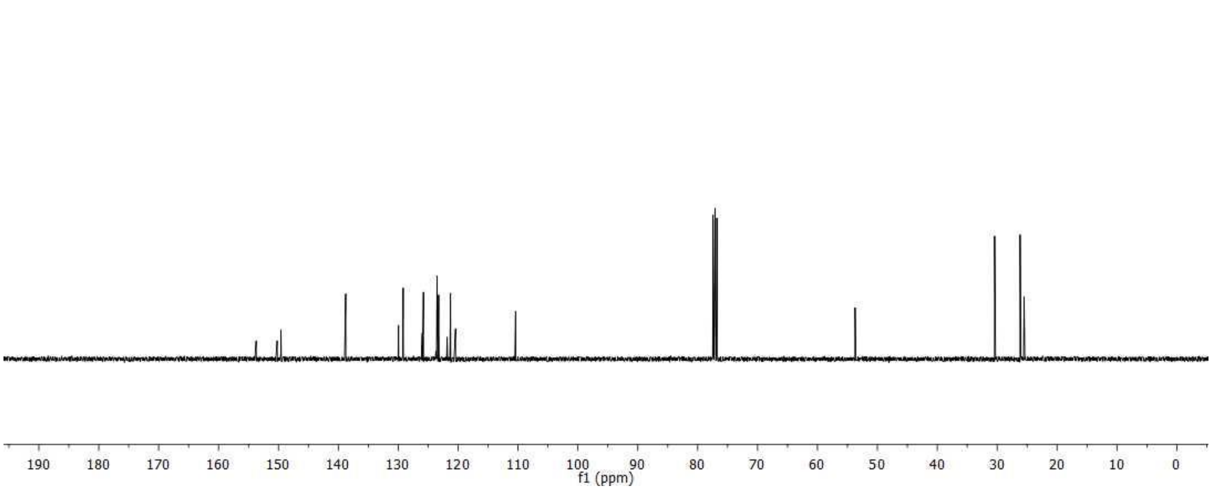

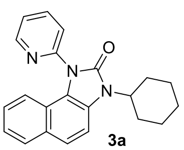

3a

100 MHz, $CDCl_3$

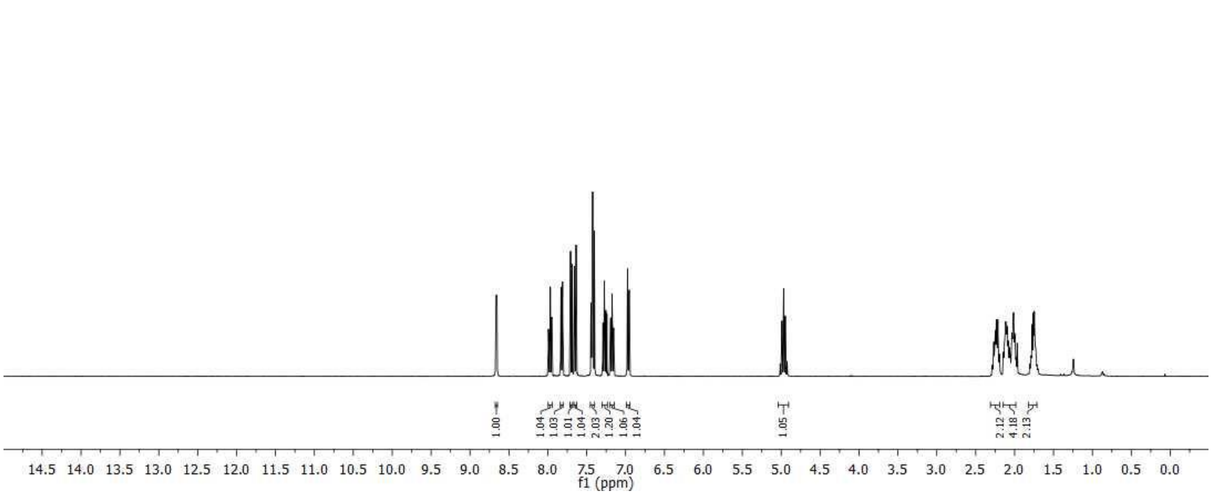

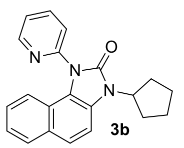

3b

400 MHz, $CDCl_3$

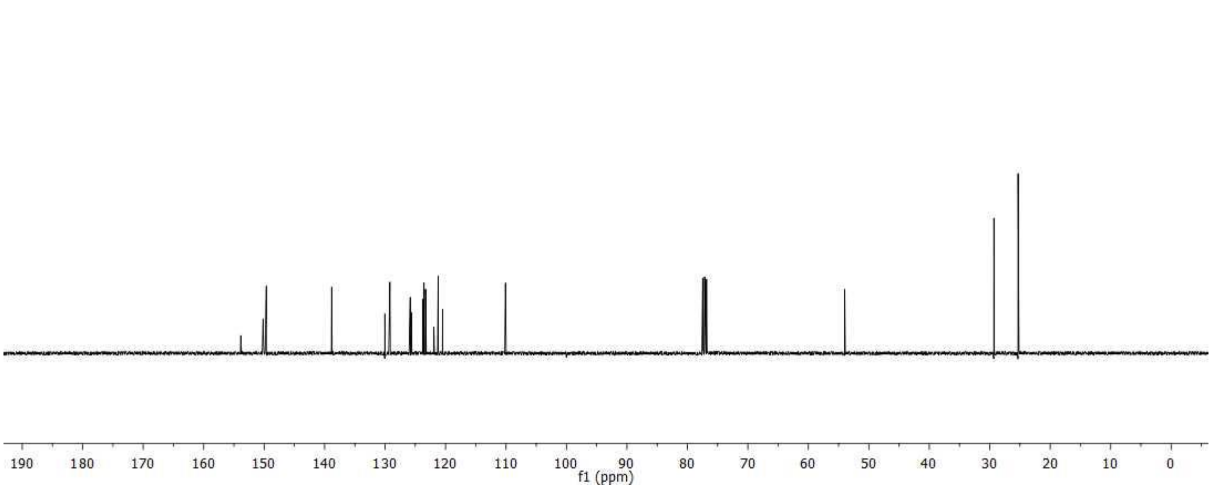

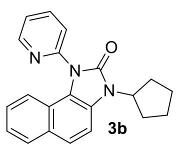

3b

100 MHz, $CDCl_3$

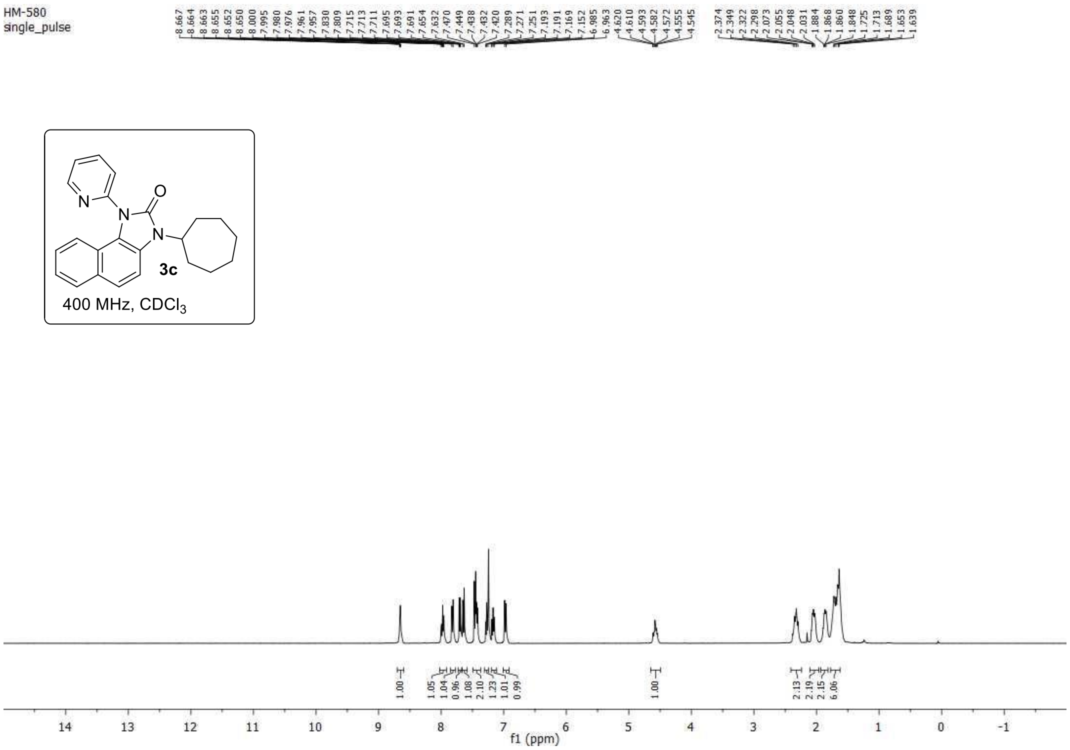
HM-580
single_pulse
8.667 8.664 8.663 8.655 8.652 8.650 8.000 7.995 7.980 7.976 7.961 7.957 7.830 7.809 7.715 7.713 7.711 7.695 7.693 7.691 7.654 7.632 7.470 7.449 7.438 7.432 7.420 7.289 7.271 7.251 7.193 7.191 7.169 7.152 6.985 6.963 4.620 4.610 4.593 4.582 4.572 4.555 4.545 2.374 2.349 2.322 2.298 2.073 2.055 2.048 2.031 1.884 1.868 1.860 1.848 1.725 1.713 1.689 1.653 1.639
N
O
N
N
3c
400 MHz, CDCl3
1.00 1.05 1.04 0.96 1.08 2.10 1.23 1.01 0.99 1.00 2.13 2.19 2.15 6.06
14 13 12 11 10 9 8 7 6 5 4 3 2 1 0 -1
f1 (ppm)

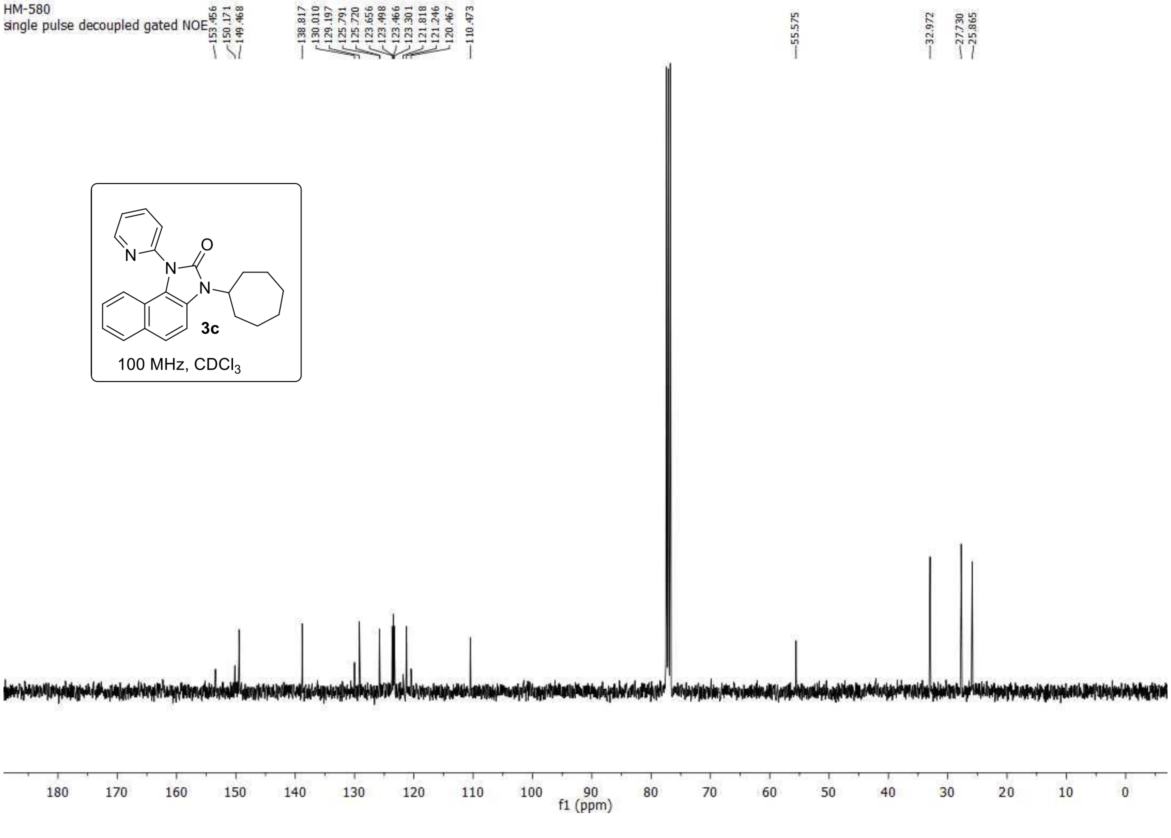
HM-580
single pulse decoupled gated NOE
153.456 150.171 149.468 138.817 130.010 129.197 125.791 125.720 123.656 123.498 123.466 123.301 121.818 121.246 120.467 110.473 55.575 32.972 27.730 25.865
N
O
N
N
3c
100 MHz, CDCl3
180 170 160 150 140 130 120 110 100 90 80 70 60 50 40 30 20 10 0
f1 (ppm)

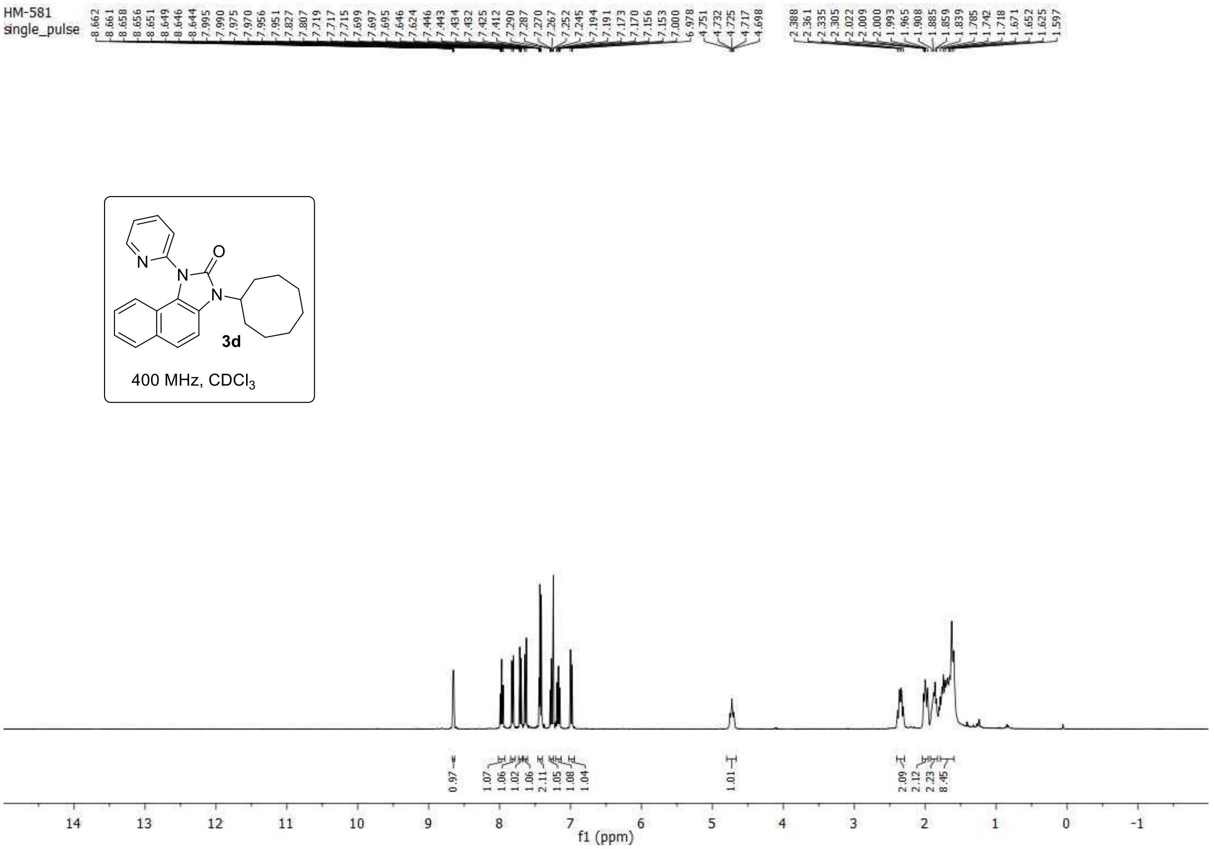
HM-581
single_pulse
8.662 8.661 8.658 8.656 8.651 8.649 8.646 8.644 7.995 7.990 7.975 7.970 7.956 7.951 7.827 7.807 7.719 7.717 7.715 7.699 7.697 7.695 7.646 7.624 7.446 7.443 7.434 7.432 7.425 7.412 7.290 7.287 7.270 7.267 7.252 7.245 7.194 7.191 7.173 7.170 7.156 7.153 7.000 6.978 4.751 4.732 4.725 4.717 4.698 2.388 2.361 2.335 2.305 2.022 2.009 2.000 1.993 1.965 1.908 1.885 1.859 1.839 1.785 1.742 1.718 1.671 1.652 1.625 1.597
3d
400 MHz, CDCl3
0.97 1.07 1.06 1.02 1.06 2.11 1.05 1.08 1.04 1.01 2.09 2.12 2.23 8.45
f1 (ppm)

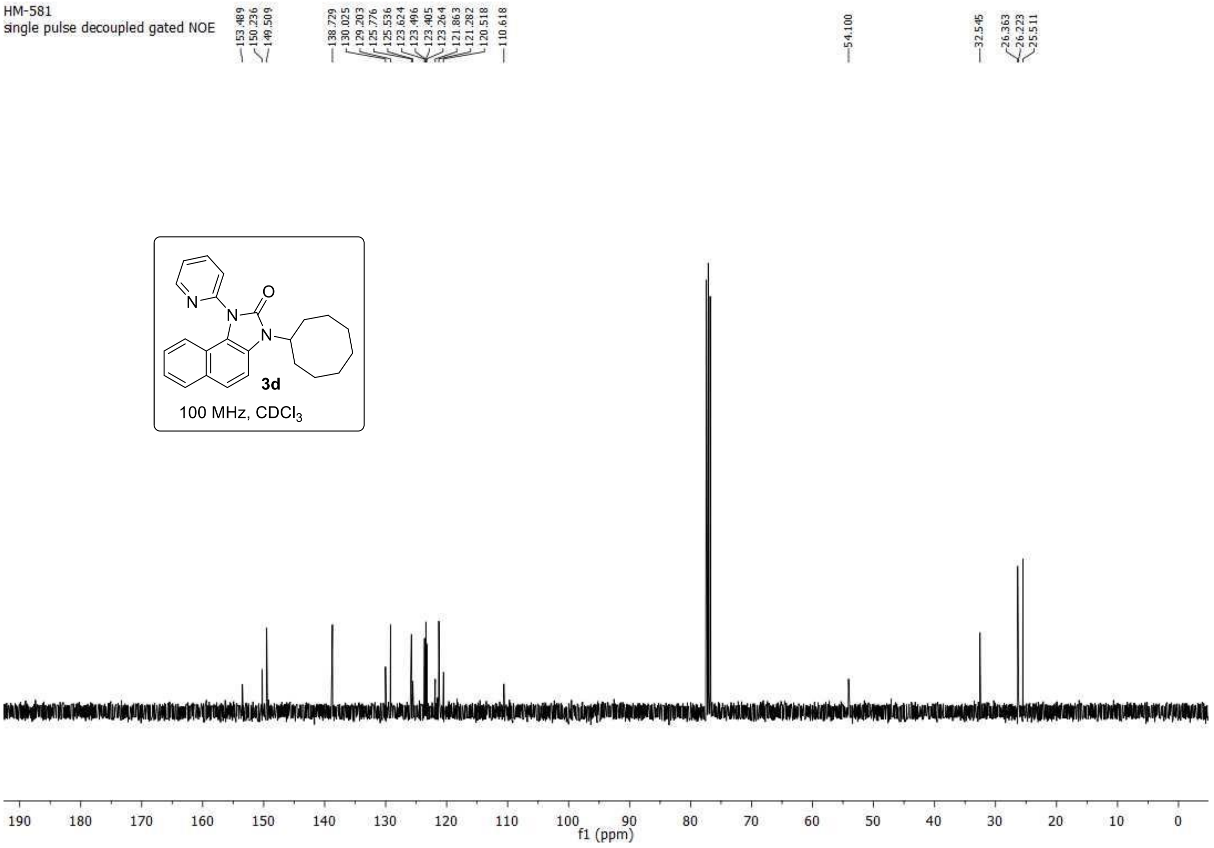
HM-581
single pulse decoupled gated NOE
153.489 150.236 149.509 138.729 130.025 129.203 125.776 125.536 123.624 123.496 123.405 123.264 121.863 121.282 120.518 110.618 54.100 32.545 26.363 26.223 25.511
3d
100 MHz, CDCl3
f1 (ppm)

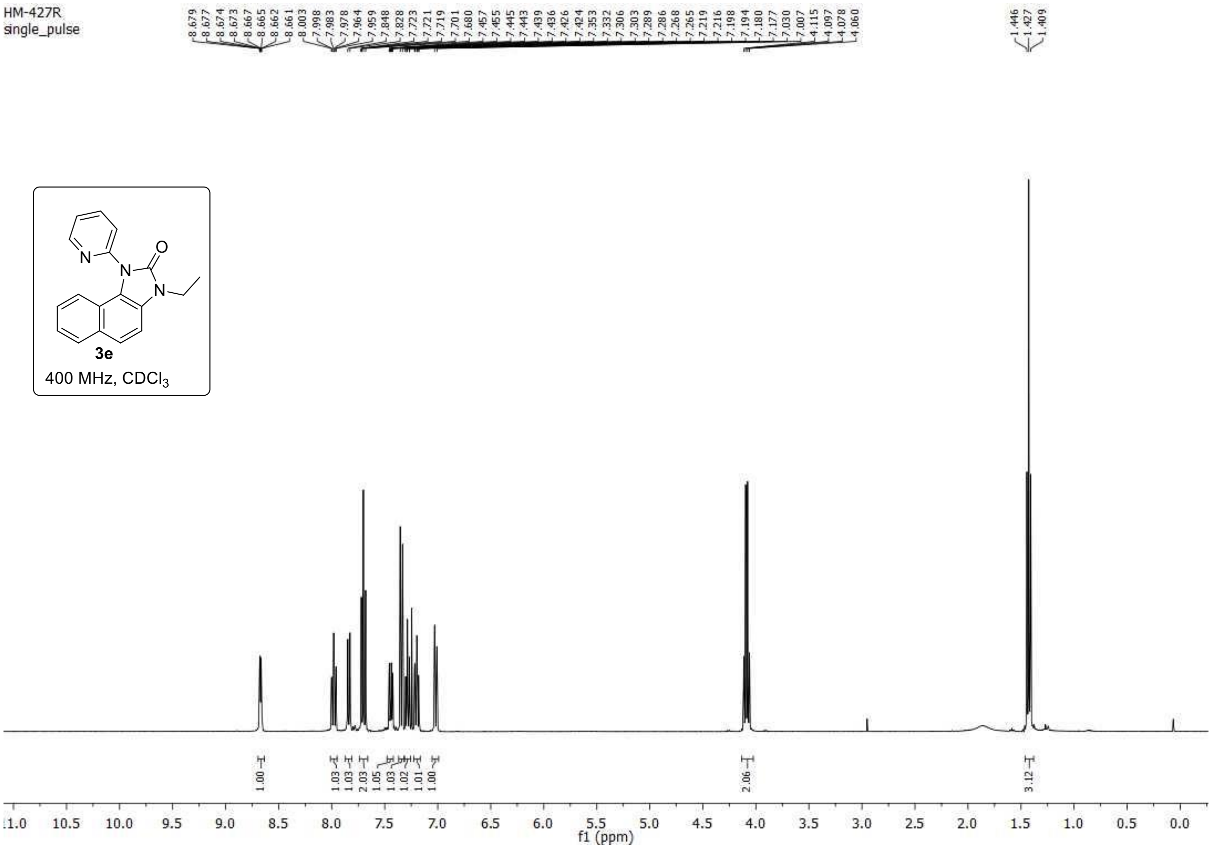
HM-427R
single_pulse
8.679 8.677 8.674 8.673 8.667 8.665 8.662 8.661 8.003 7.998 7.983 7.978 7.964 7.959 7.848 7.828 7.723 7.721 7.719 7.701 7.680 7.457 7.455 7.445 7.443 7.439 7.436 7.426 7.424 7.353 7.332 7.306 7.303 7.289 7.286 7.268 7.265 7.219 7.216 7.198 7.194 7.180 7.177 7.030 7.007 4.115 4.097 4.078 4.060
1.446 1.427 1.409
N
N
O
N
3e
400 MHz, CDCl3
1.00
1.03 1.03 2.03 1.05 1.03 1.02 1.01 1.00
2.06
3.12
f1 (ppm)
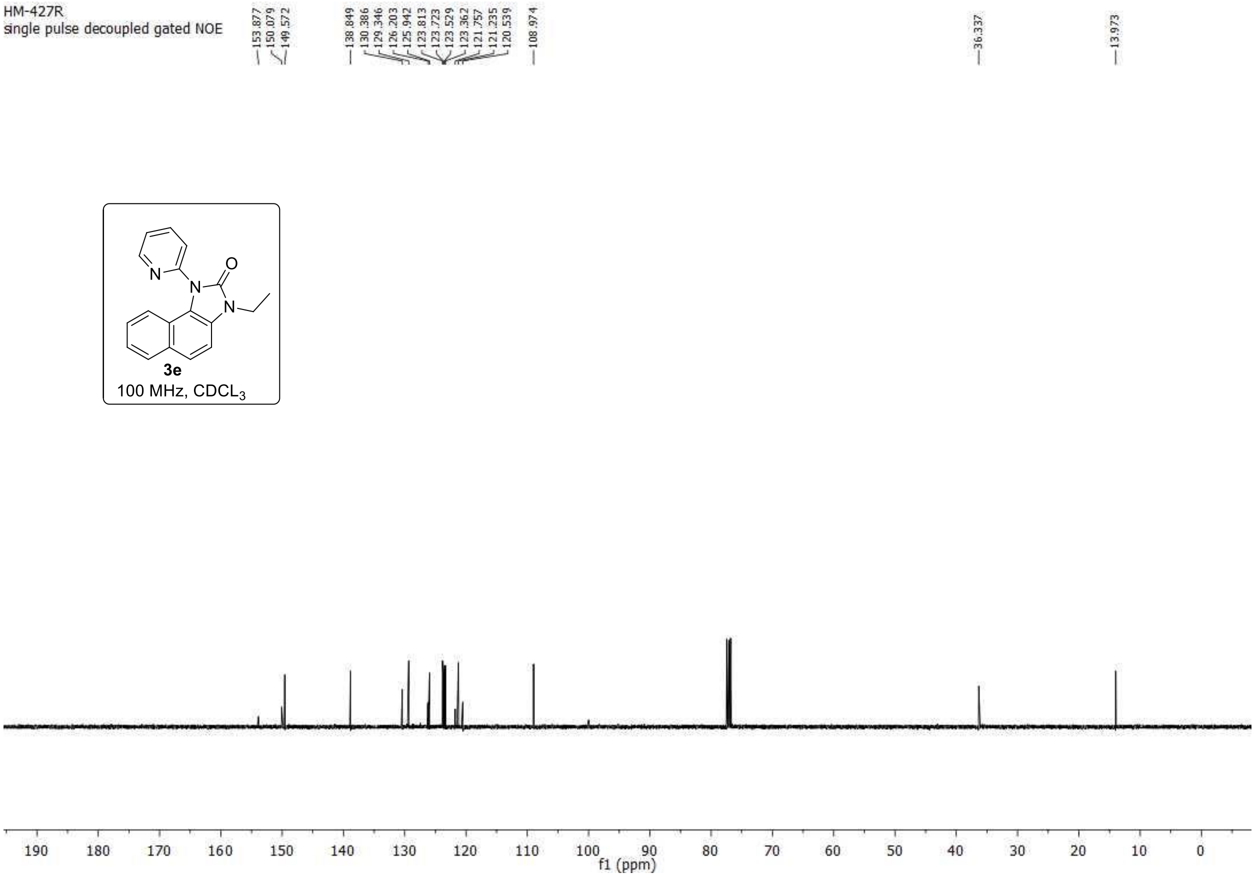
HM-427R
single pulse decoupled gated NOE
153.877 150.079 149.572
138.849 130.386 129.346 126.203 125.942 123.813 123.723 123.529 123.362 121.757 121.235 120.539
108.974
36.337
13.973
N
N
O
N
3e
100 MHz, CDCL3
f1 (ppm)

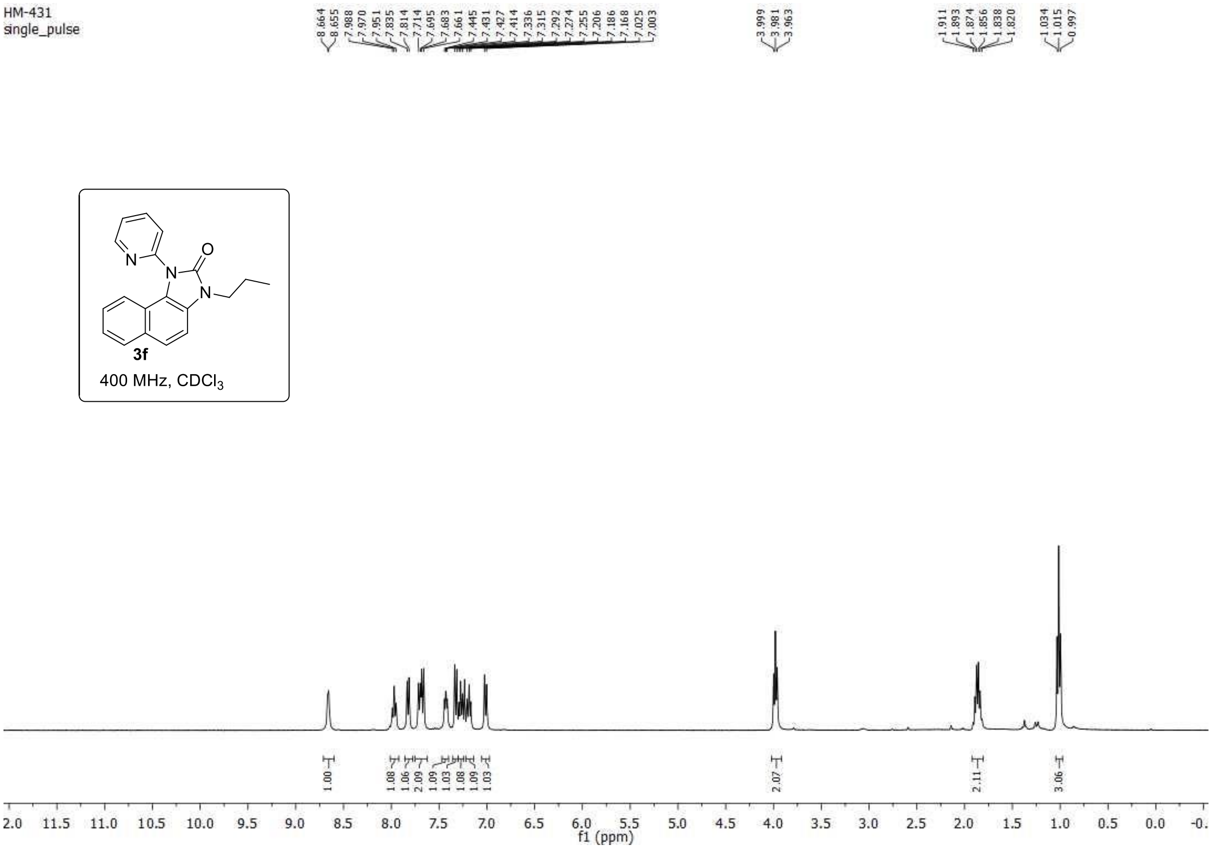
HM-431
single_pulse
8.664
8.655
7.988
7.970
7.951
7.835
7.814
7.714
7.695
7.683
7.661
7.445
7.431
7.427
7.414
7.336
7.315
7.292
7.274
7.255
7.206
7.186
7.168
7.025
7.003
3.999
3.981
3.963
1.911
1.893
1.874
1.856
1.838
1.820
1.034
1.015
0.997
3f
400 MHz, CDCl3
1.00
1.08
1.06
2.09
1.09
1.03
1.08
1.09
1.03
2.07
2.11
3.06
f1 (ppm)

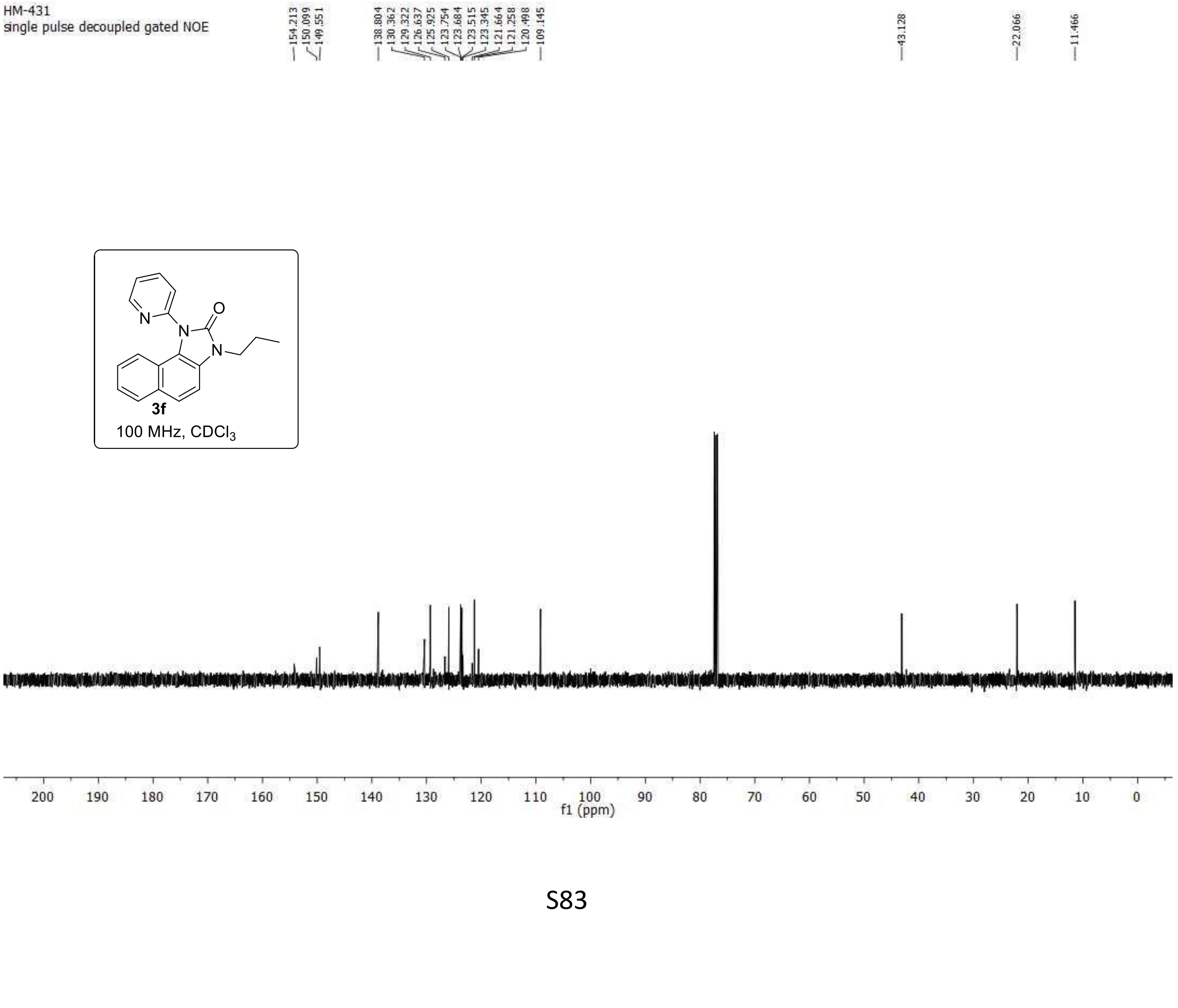
HM-431
single pulse decoupled gated NOE
154.213
150.099
149.551
138.804
130.362
129.322
126.637
125.925
123.754
123.684
123.515
123.345
121.664
121.258
120.498
109.145
43.128
22.066
11.466
3f
100 MHz, CDCl3
f1 (ppm)

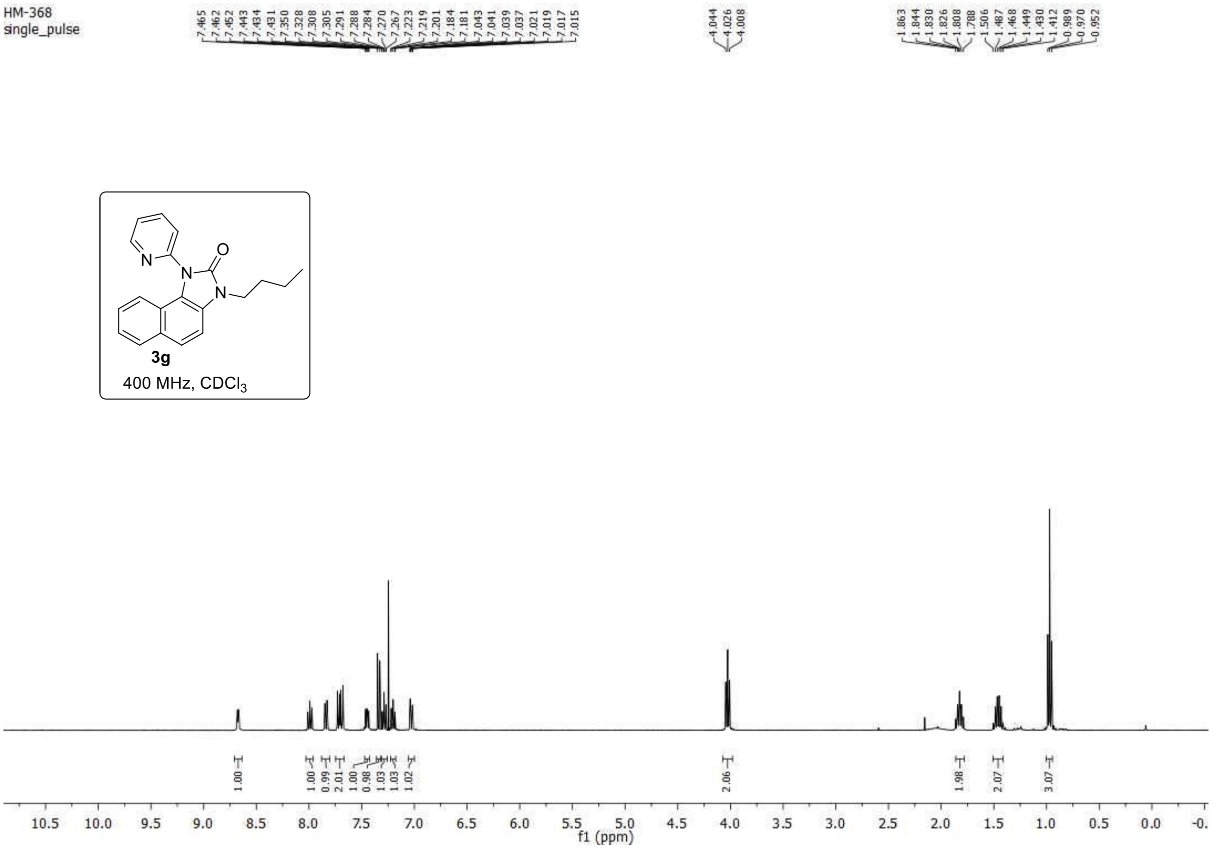

HM-368
single_pulse
7.465 7.462 7.452 7.443 7.434 7.431 7.350 7.328 7.308 7.305 7.291 7.288 7.284 7.270 7.267 7.223 7.219 7.201 7.184 7.181 7.043 7.041 7.039 7.037 7.021 7.019 7.017 7.015
4.044 4.026 4.008
1.863 1.844 1.830 1.826 1.808 1.788 1.506 1.487 1.468 1.449 1.430 1.412 0.989 0.970 0.952
N
O
N
N
3g
400 MHz, $CDCl_3$
1.00 1.00 0.99 2.01 1.00 0.98 1.03 1.03 1.02 2.06 1.98 2.07 3.07
f1 (ppm)

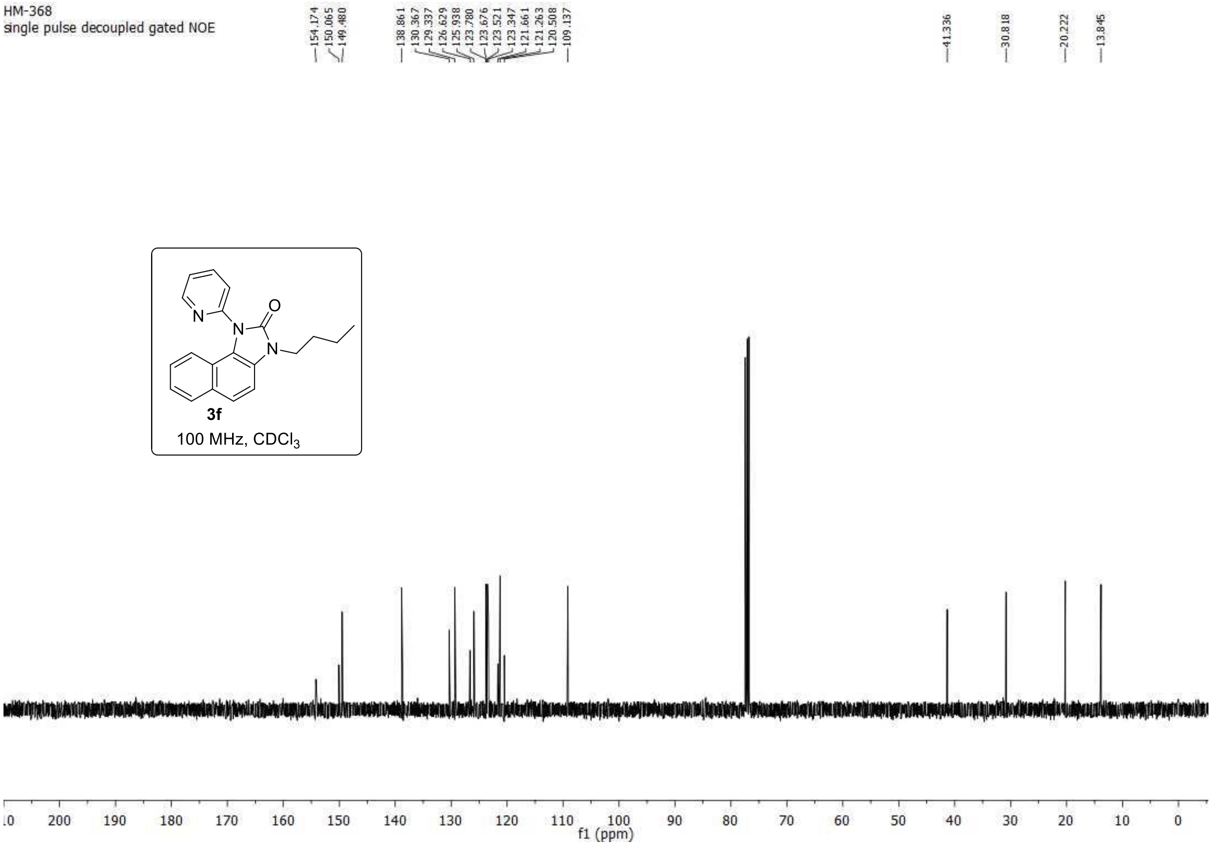

HM-368
single pulse decoupled gated NOE
154.174 150.065 149.480 138.861 130.367 129.337 126.629 125.938 123.780 123.676 123.521 123.347 121.661 121.263 120.508 109.137
41.336 30.818 20.222 13.845
N
O
N
N
3f
100 MHz, $CDCl_3$
f1 (ppm)

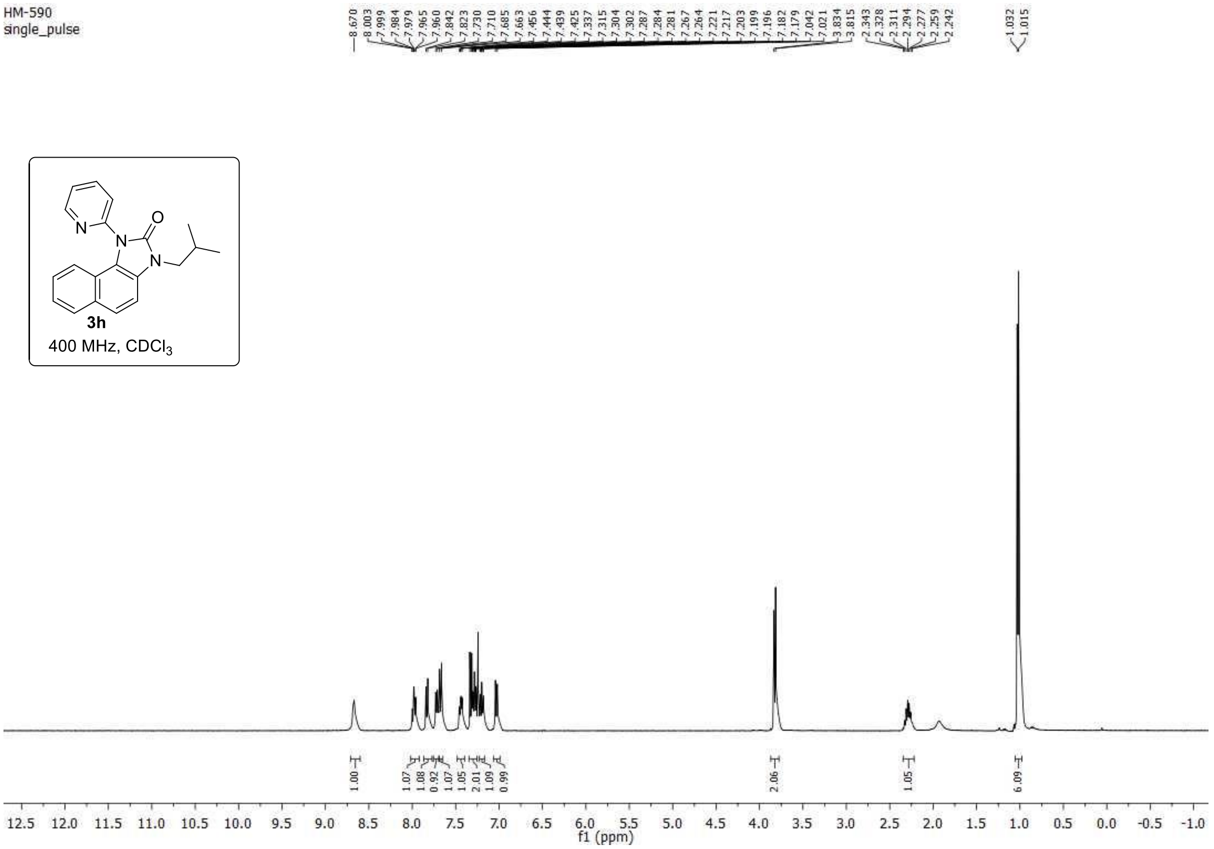
HM-590
single_pulse
8.670 8.003 7.999 7.984 7.979 7.965 7.960 7.842 7.823 7.730 7.710 7.685 7.663 7.456 7.444 7.439 7.425 7.337 7.315 7.304 7.302 7.287 7.284 7.281 7.267 7.264 7.221 7.217 7.203 7.199 7.196 7.182 7.179 7.042 7.021 3.834 3.815 2.343 2.328 2.311 2.294 2.277 2.259 2.242 1.032 1.015
3h
400 MHz, CDCl3
1.00 1.07 1.08 0.92 1.07 1.05 2.01 1.09 0.99 2.06 1.05 6.09
f1 (ppm)

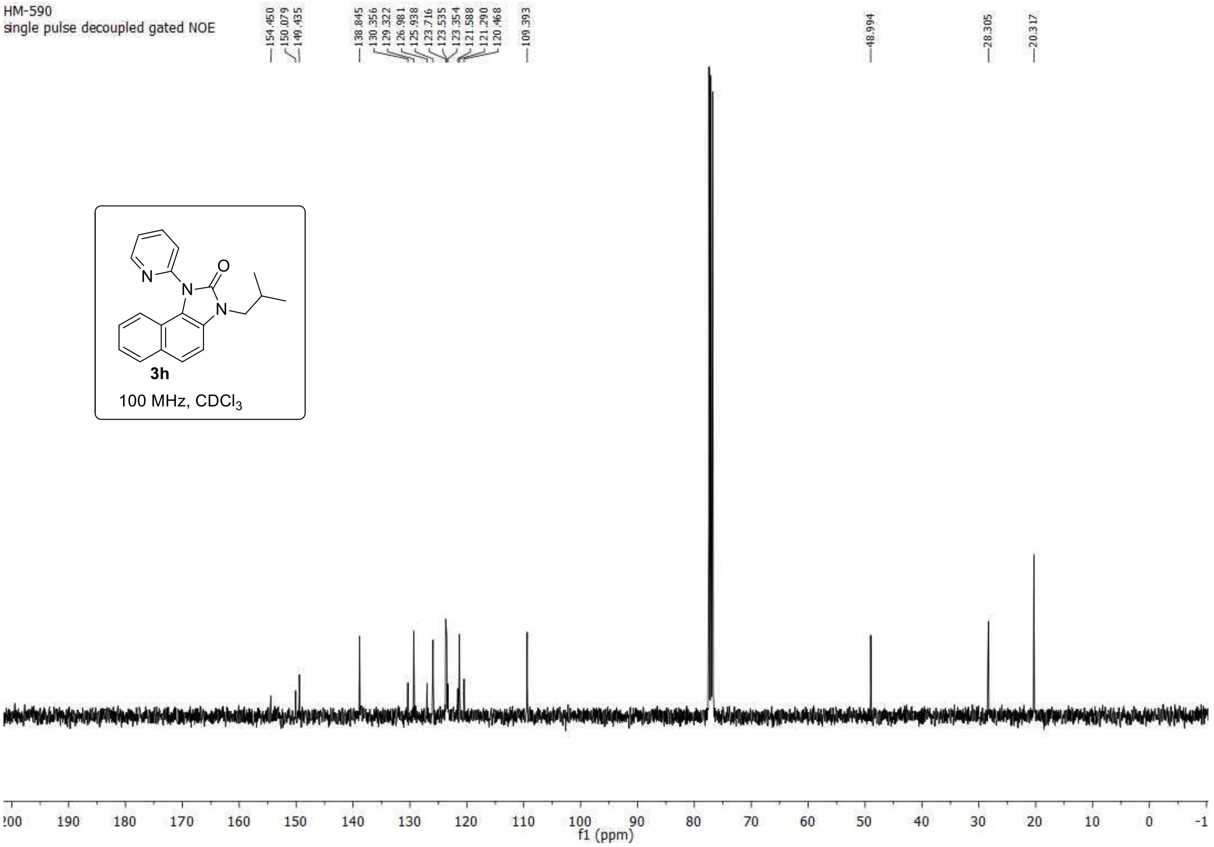
HM-590
single pulse decoupled gated NOE
154.450 150.079 149.435 138.845 130.356 129.322 126.981 125.938 123.716 123.535 123.354 121.588 121.290 120.468 109.393 48.994 28.305 20.317
3h
100 MHz, CDCl3
f1 (ppm)

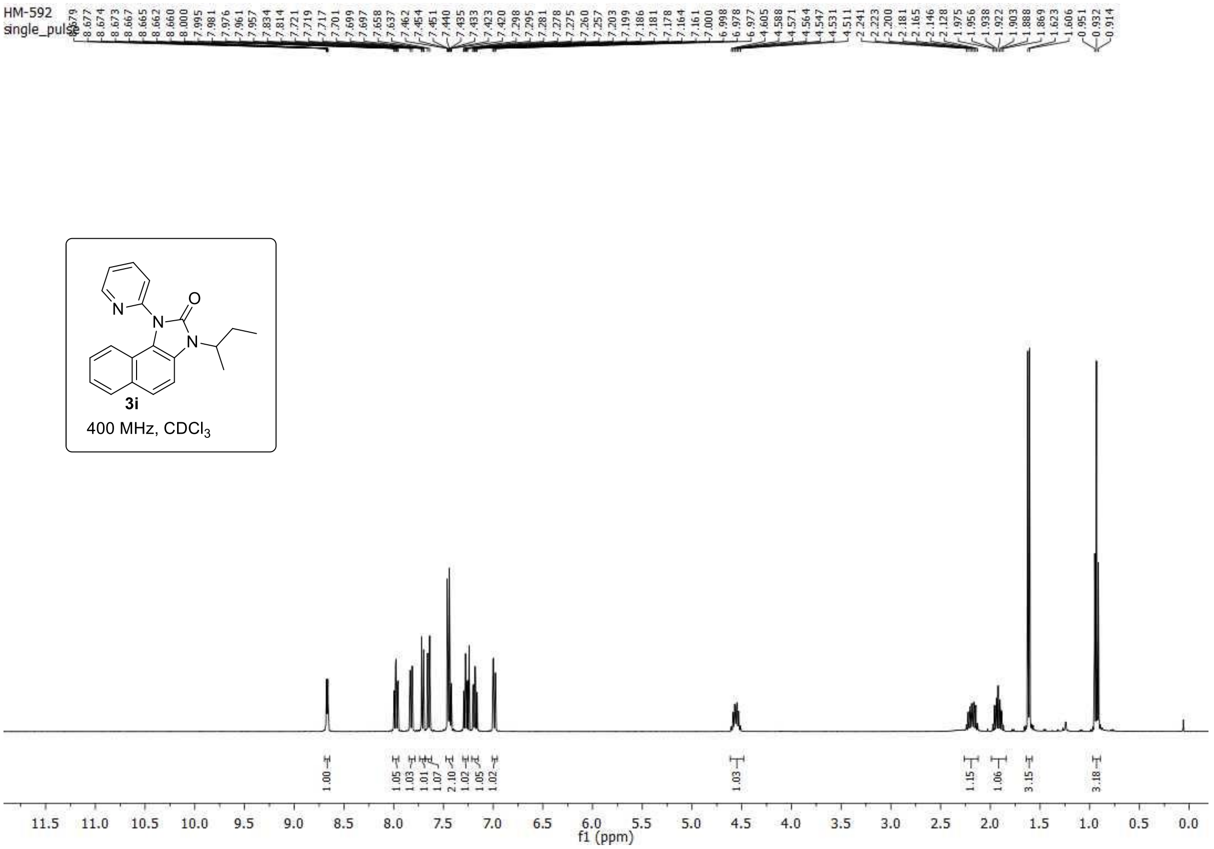
HM-592
single_pulse
8.679 8.677 8.674 8.673 8.667 8.665 8.662 8.660 8.000 7.995 7.981 7.976 7.961 7.957 7.834 7.814 7.721 7.719 7.717 7.701 7.699 7.697 7.658 7.637 7.462 7.454 7.451 7.440 7.435 7.433 7.423 7.420 7.298 7.295 7.281 7.278 7.275 7.260 7.257 7.203 7.199 7.186 7.181 7.178 7.164 7.161 7.000 6.998 6.978 6.977 4.605 4.588 4.571 4.564 4.547 4.531 4.511 2.241 2.223 2.200 2.181 2.165 2.146 2.128 1.975 1.956 1.938 1.922 1.903 1.888 1.869 1.623 1.606 0.951 0.932 0.914
N
O
N
N
3i
400 MHz, $CDCl_3$
1.00 1.05 1.03 1.01 1.07 2.10 1.02 1.05 1.02 1.03 1.15 1.06 3.15 3.18
11.5 11.0 10.5 10.0 9.5 9.0 8.5 8.0 7.5 7.0 6.5 6.0 5.5 5.0 4.5 4.0 3.5 3.0 2.5 2.0 1.5 1.0 0.5 0.0
f1 (ppm)

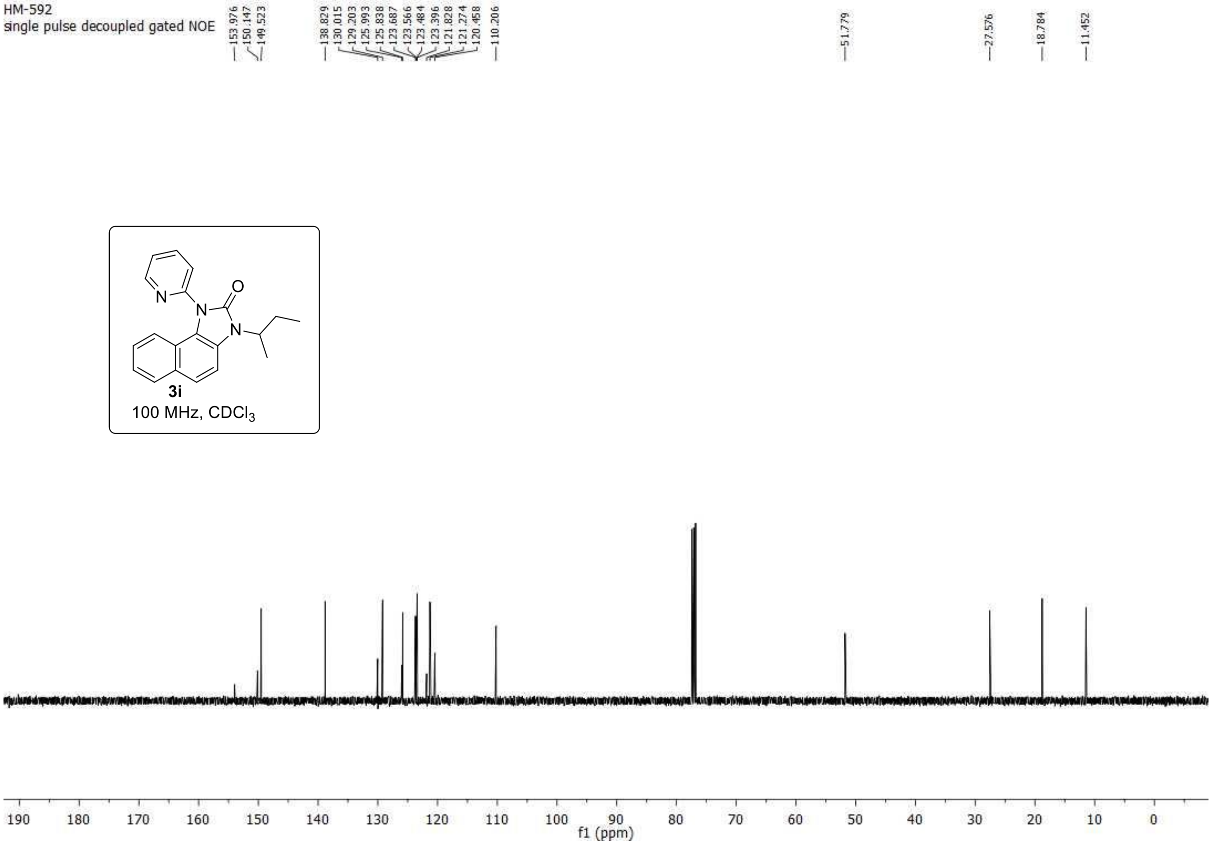
HM-592
single pulse decoupled gated NOE
153.976 150.147 149.523 138.829 130.015 129.203 125.993 125.838 123.687 123.566 123.484 123.396 121.828 121.274 120.458 110.206 51.779 27.576 18.784 11.452
N
O
N
N
3i
100 MHz, $CDCl_3$
190 180 170 160 150 140 130 120 110 100 90 80 70 60 50 40 30 20 10 0
f1 (ppm)

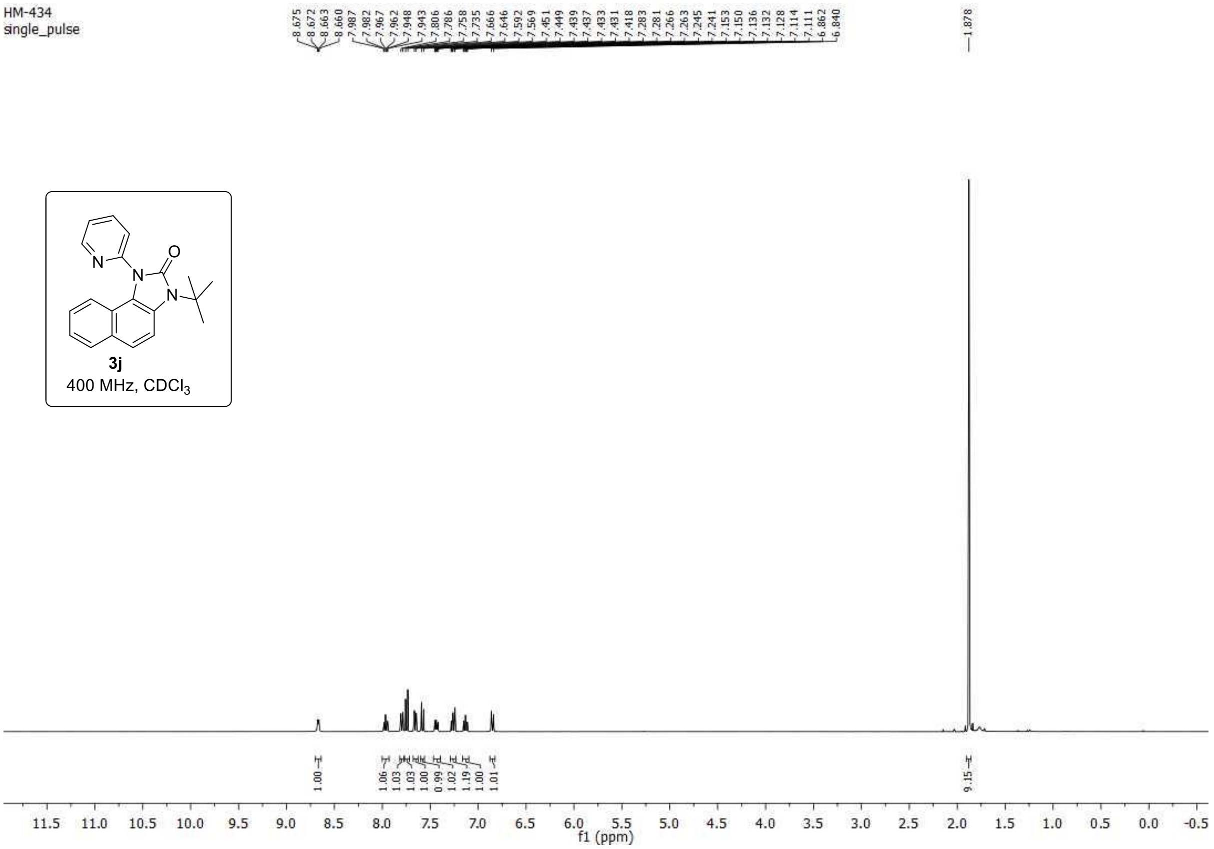
HM-434
single_pulse
8.675 8.672 8.663 8.660 7.987 7.982 7.967 7.962 7.948 7.943 7.806 7.786 7.758 7.735 7.666 7.646 7.592 7.569 7.451 7.449 7.439 7.437 7.433 7.431 7.418 7.283 7.281 7.266 7.263 7.245 7.241 7.153 7.150 7.136 7.132 7.128 7.114 7.111 6.862 6.840
1.878
3j
400 MHz, CDCl3
1.00
1.06 1.03 1.03 1.00 0.99 1.02 1.19 1.00 1.01
9.15
11.5 11.0 10.5 10.0 9.5 9.0 8.5 8.0 7.5 7.0 6.5 6.0 5.5 5.0 4.5 4.0 3.5 3.0 2.5 2.0 1.5 1.0 0.5 0.0 -0.5
f1 (ppm)

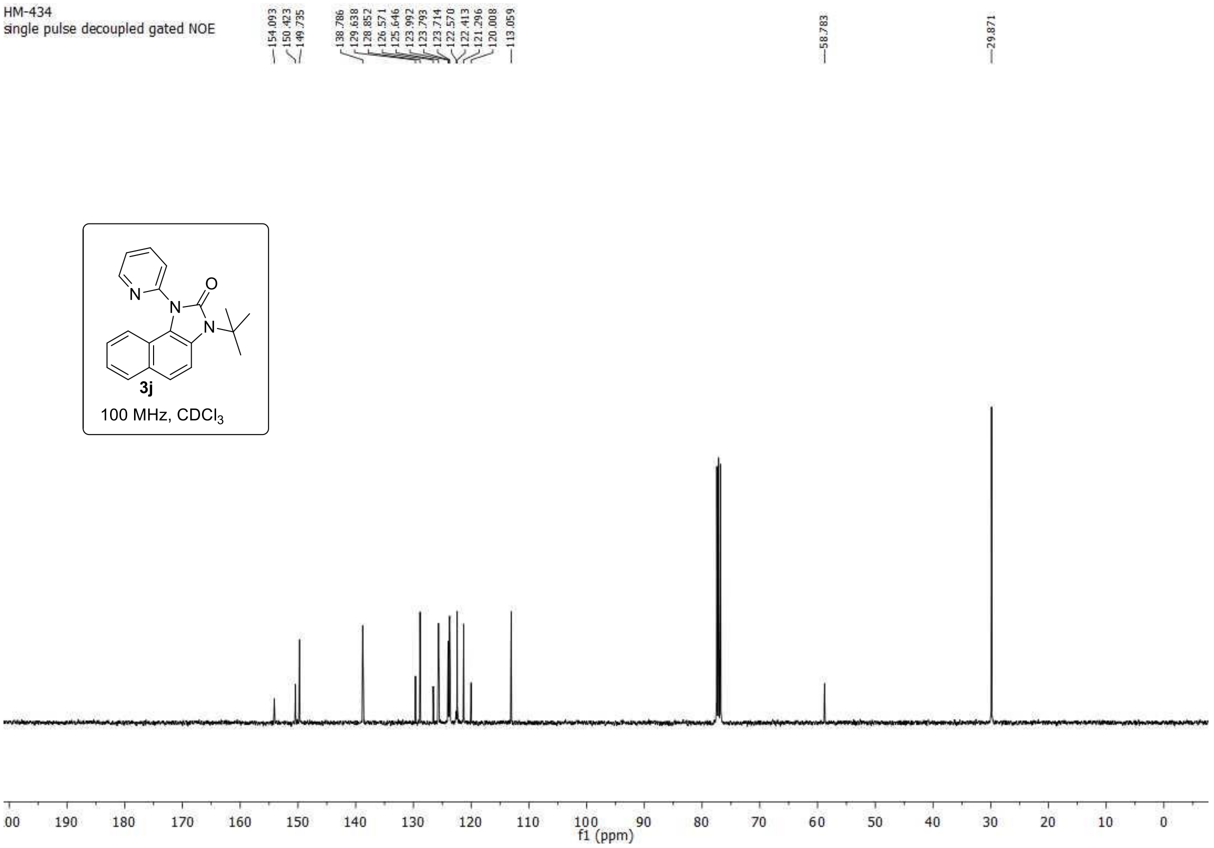
HM-434
single pulse decoupled gated NOE
154.093 150.423 149.735
138.786 129.638 128.852 126.571 125.646 123.992 123.793 123.714 122.570 122.413 121.296 120.008
113.059
58.783
29.871
3j
100 MHz, CDCl3
00 190 180 170 160 150 140 130 120 110 100 90 80 70 60 50 40 30 20 10 0
f1 (ppm)

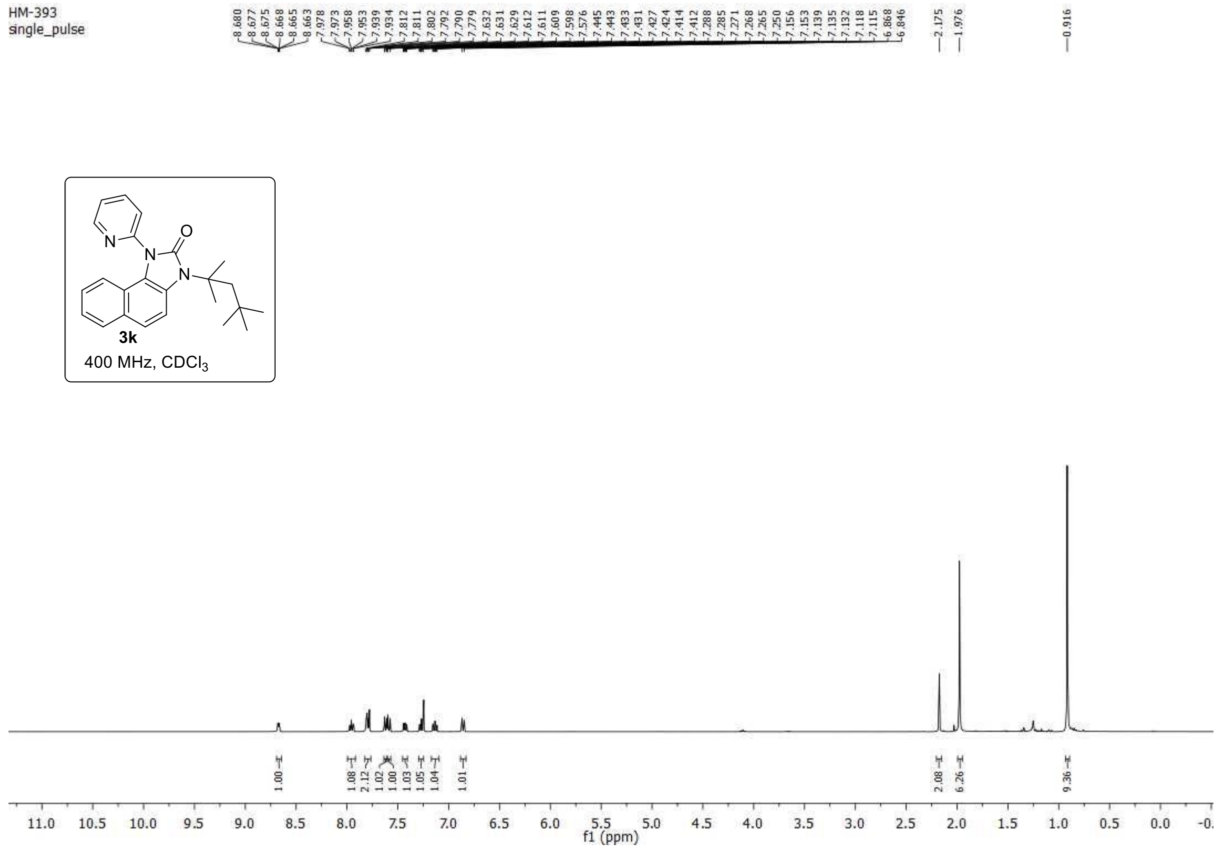
HM-393
single_pulse
8.680 8.677 8.675 8.668 8.665 8.663 7.978 7.973 7.958 7.953 7.939 7.934 7.812 7.811 7.802 7.792 7.790 7.779 7.632 7.631 7.629 7.612 7.611 7.609 7.598 7.576 7.445 7.443 7.433 7.431 7.427 7.424 7.414 7.412 7.288 7.285 7.271 7.268 7.265 7.250 7.156 7.153 7.139 7.135 7.132 7.118 7.115 6.868 6.846
2.175
1.976
0.916
N
O
N
N
3k
400 MHz, CDCl3
1.00
1.08
2.12
1.02
1.00
1.03
1.05
1.04
1.01
2.08
6.26
9.36
f1 (ppm)

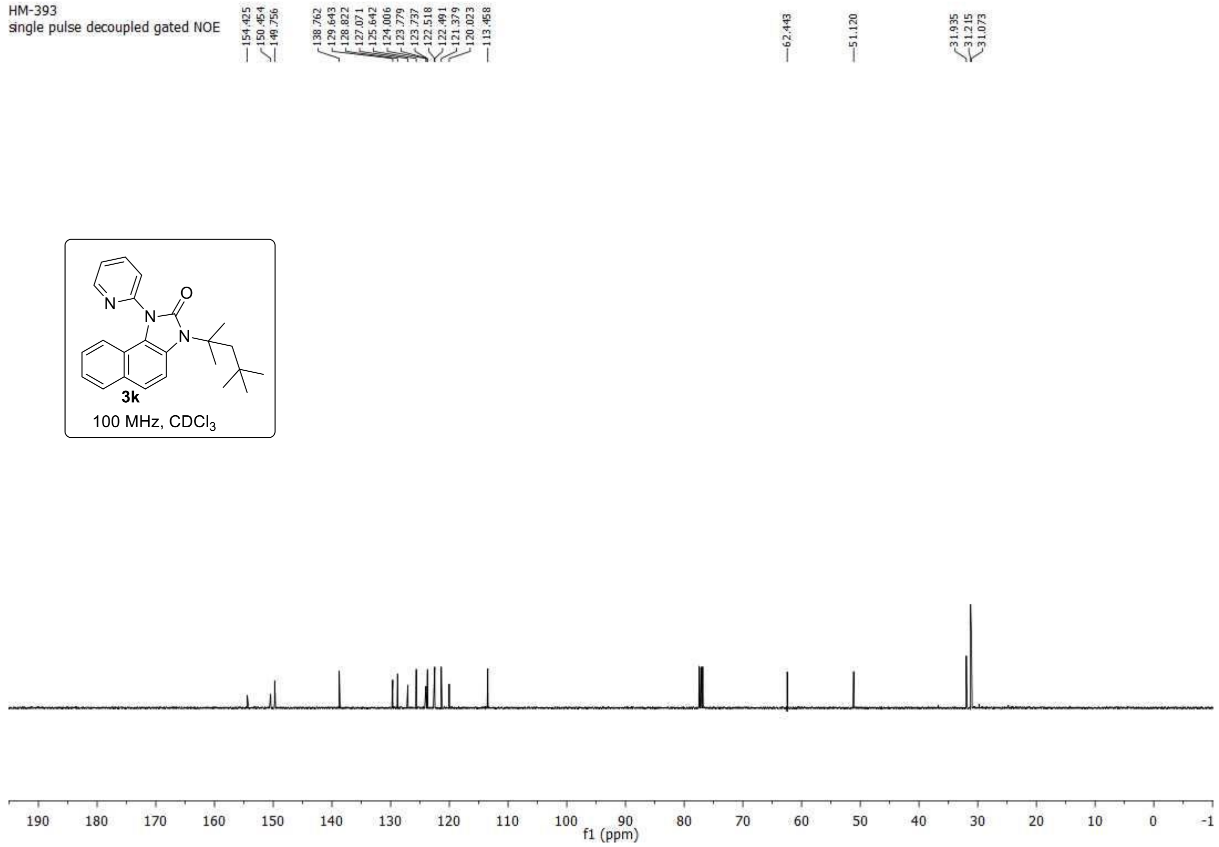
HM-393
single pulse decoupled gated NOE
154.425 150.454 149.756
138.762 129.643 128.822 127.071 125.642 124.006 123.779 123.737 122.518 122.491 121.379 120.023
113.458
62.443
51.120
31.935 31.215 31.073
N
O
N
N
3k
100 MHz, CDCl3
f1 (ppm)

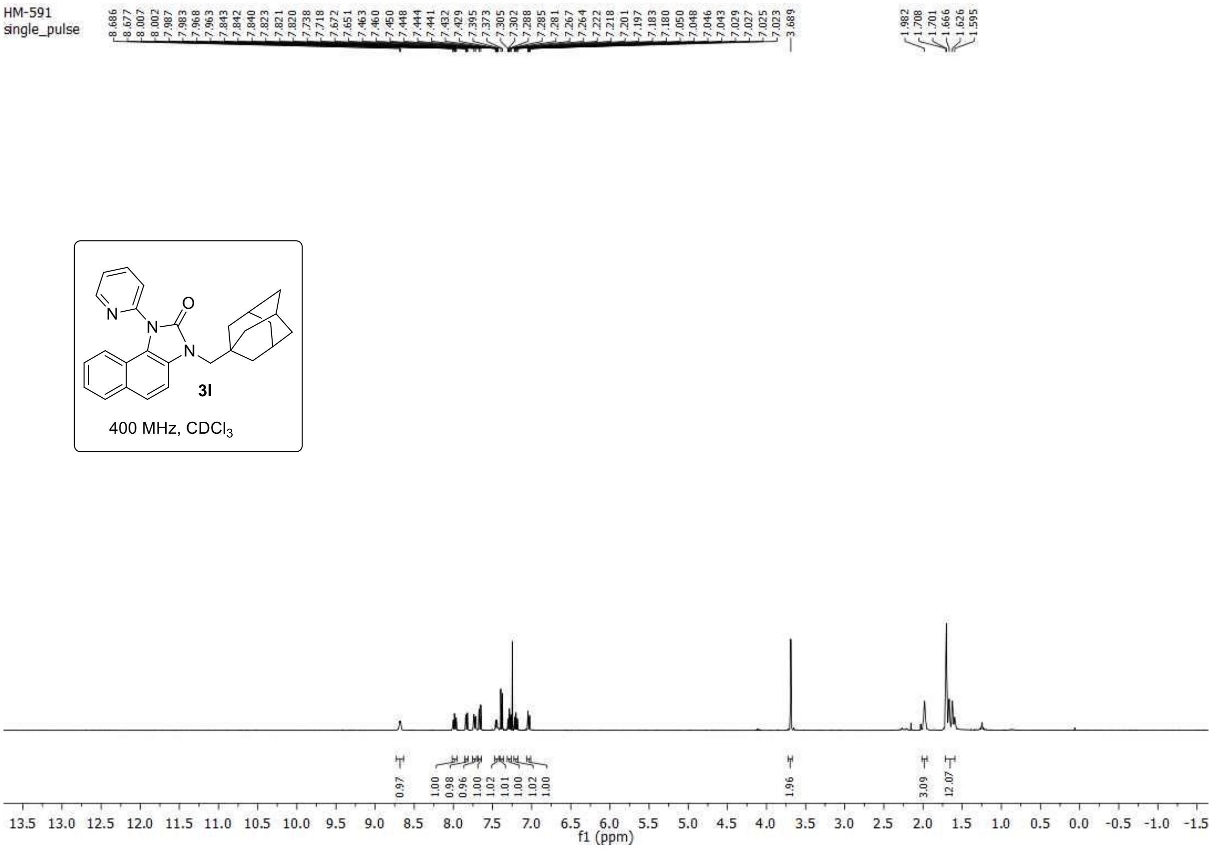

HM-591
single_pulse
8.686, 8.677, 8.007, 8.002, 7.987, 7.983, 7.968, 7.963, 7.843, 7.842, 7.840, 7.823, 7.821, 7.820, 7.738, 7.718, 7.672, 7.651, 7.463, 7.460, 7.450, 7.448, 7.444, 7.441, 7.432, 7.429, 7.395, 7.373, 7.305, 7.302, 7.288, 7.285, 7.281, 7.267, 7.264, 7.222, 7.218, 7.201, 7.197, 7.183, 7.180, 7.050, 7.048, 7.046, 7.043, 7.029, 7.027, 7.025, 7.023, 3.689
1.982, 1.708, 1.701, 1.666, 1.626, 1.595
N
N
O
N
3l
400 MHz, CDCl3
0.97, 1.00, 0.98, 0.96, 1.00, 1.02, 1.01, 1.00, 1.02, 1.00, 1.96, 3.09, 12.07
f1 (ppm)

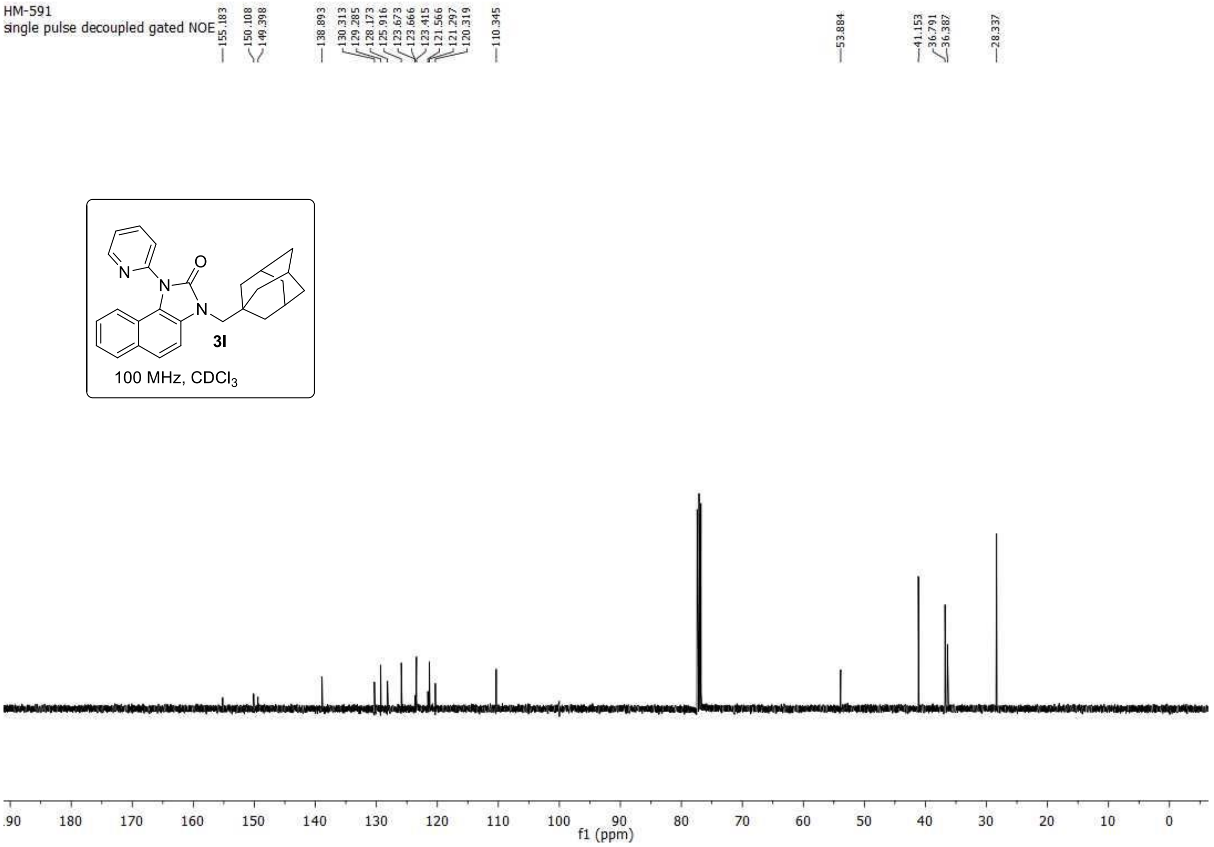

HM-591
single pulse decoupled gated NOE
155.183, 150.108, 149.398, 138.893, 130.313, 129.285, 128.173, 125.916, 123.673, 123.666, 123.415, 121.566, 121.297, 120.319, 110.345, 53.884, 41.153, 36.791, 36.387, 28.337
N
N
O
N
3l
100 MHz, CDCl3
f1 (ppm)

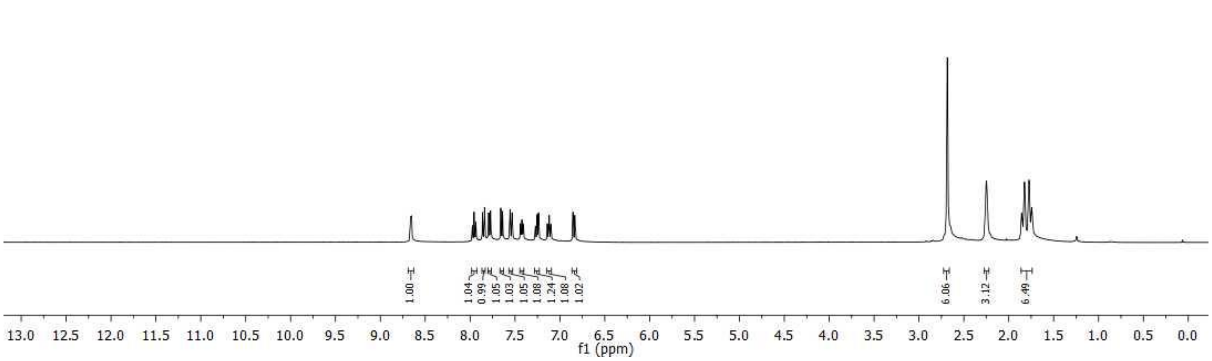

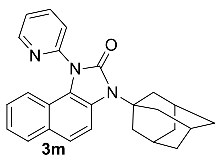

3m

400 MHz, $CDCl_3$

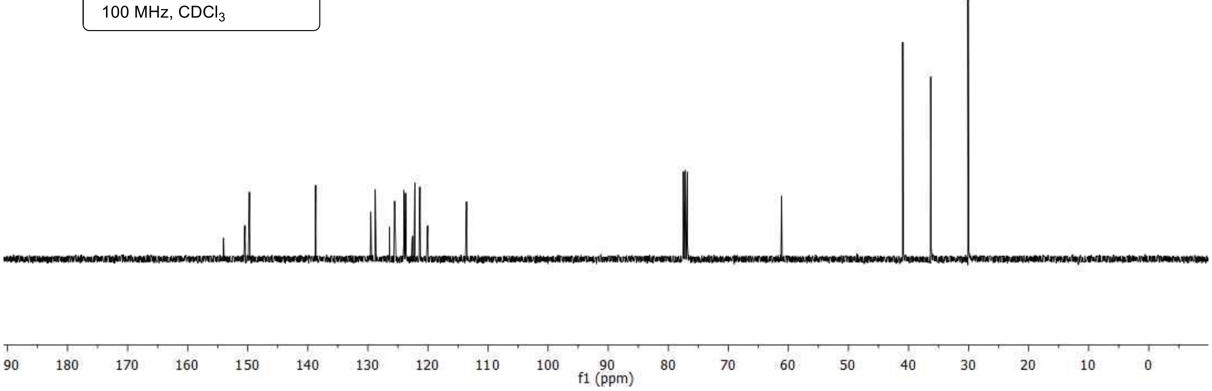

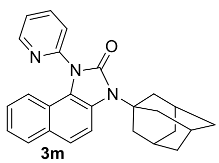

3m

100 MHz, $CDCl_3$

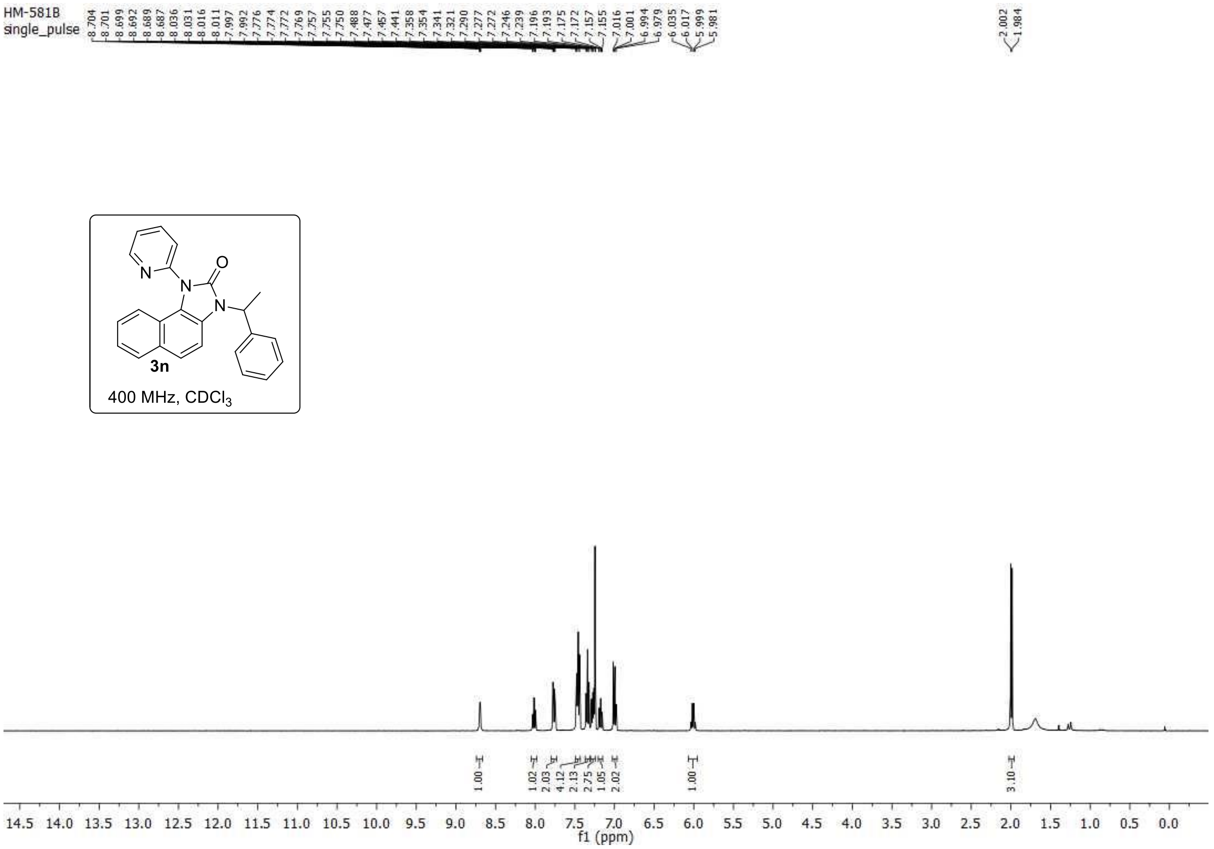

HM-581B
single_pulse
8.704 8.701 8.699 8.692 8.689 8.687 8.036 8.031 8.016 8.011 7.997 7.992 7.776 7.774 7.772 7.769 7.757 7.755 7.750 7.488 7.477 7.457 7.441 7.358 7.354 7.341 7.321 7.290 7.277 7.272 7.246 7.239 7.196 7.193 7.175 7.172 7.157 7.155 7.016 7.001 6.994 6.979 6.035 6.017 5.999 5.981
2.002 1.984
3n
400 MHz, CDCl3
1.00 1.02 2.03 4.12 2.13 2.75 1.05 2.02 1.00 3.10
14.5 14.0 13.5 13.0 12.5 12.0 11.5 11.0 10.5 10.0 9.5 9.0 8.5 8.0 7.5 7.0 6.5 6.0 5.5 5.0 4.5 4.0 3.5 3.0 2.5 2.0 1.5 1.0 0.5 0.0
f1 (ppm)

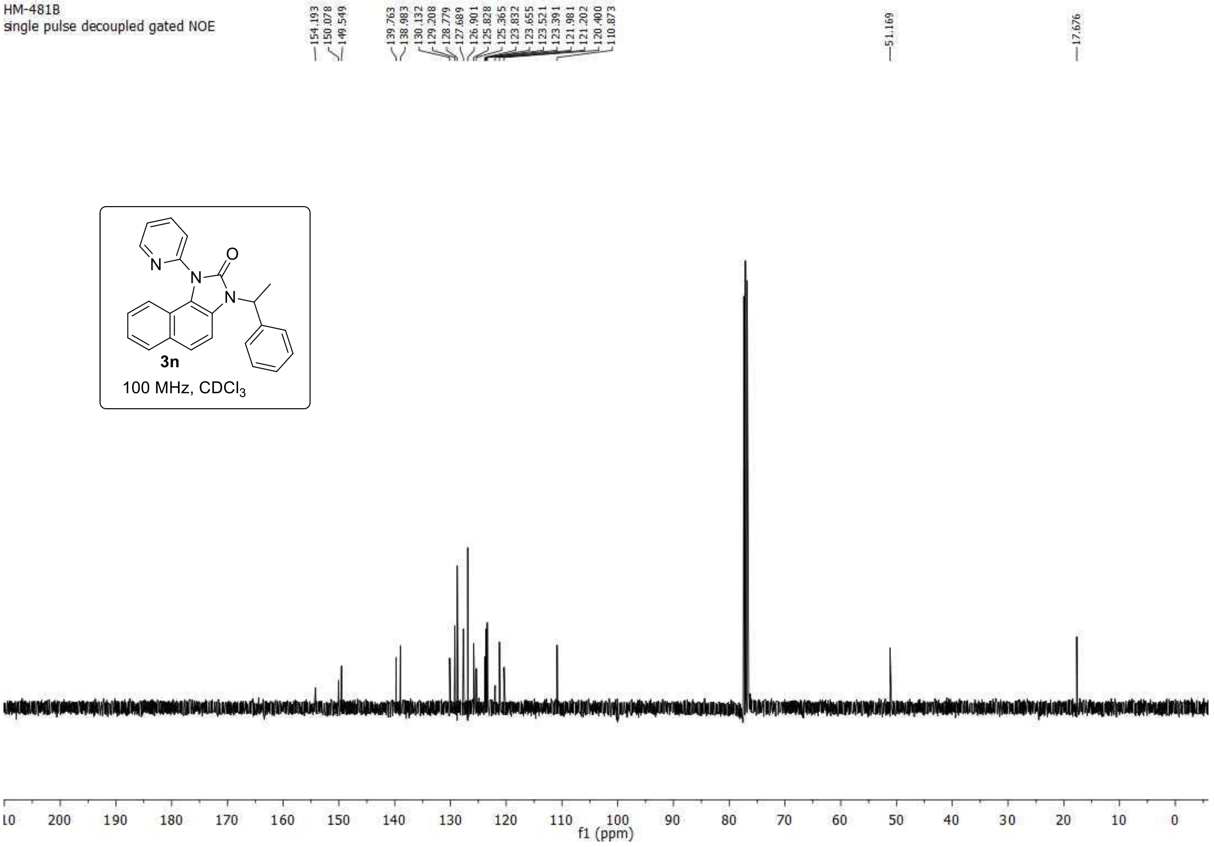

HM-481B
single pulse decoupled gated NOE
154.193 150.078 149.549 139.763 138.983 130.132 129.208 128.779 127.689 126.901 125.828 125.365 123.832 123.655 123.521 123.391 121.981 121.202 120.400 110.873
51.169
17.676
3n
100 MHz, CDCl3
10 200 190 180 170 160 150 140 130 120 110 100 90 80 70 60 50 40 30 20 10 0
f1 (ppm)

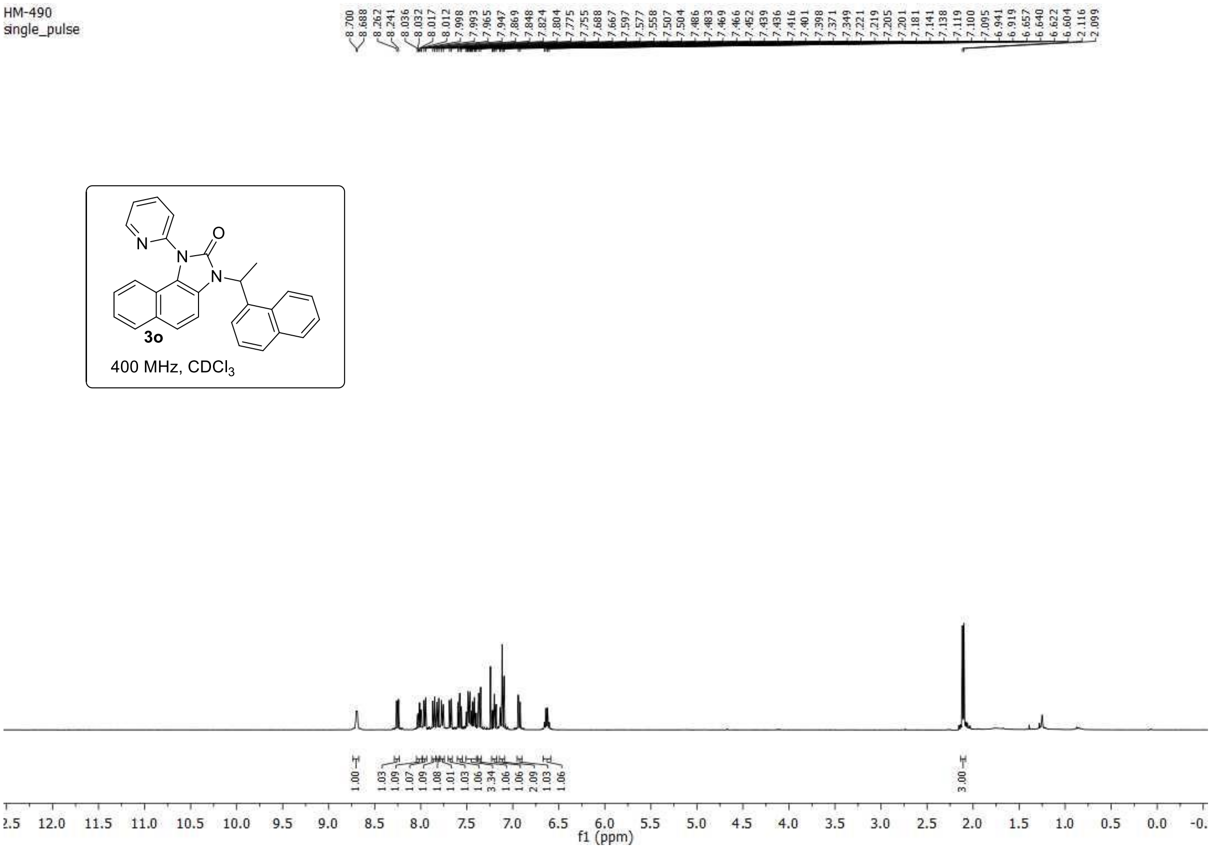
HM-490
single_pulse
8.700
8.688
8.262
8.241
8.036
8.032
8.017
8.012
7.998
7.993
7.965
7.947
7.869
7.848
7.824
7.804
7.775
7.755
7.688
7.667
7.597
7.577
7.558
7.507
7.504
7.486
7.483
7.469
7.466
7.452
7.439
7.436
7.416
7.401
7.398
7.371
7.349
7.221
7.219
7.205
7.201
7.181
7.141
7.138
7.119
7.100
7.095
6.941
6.919
6.657
6.640
6.622
6.604
2.116
2.099
3o
400 MHz, CDCl3
1.00
1.03
1.09
1.07
1.09
1.08
1.01
1.03
1.06
3.34
1.06
1.06
2.09
1.03
1.06
3.00
f1 (ppm)

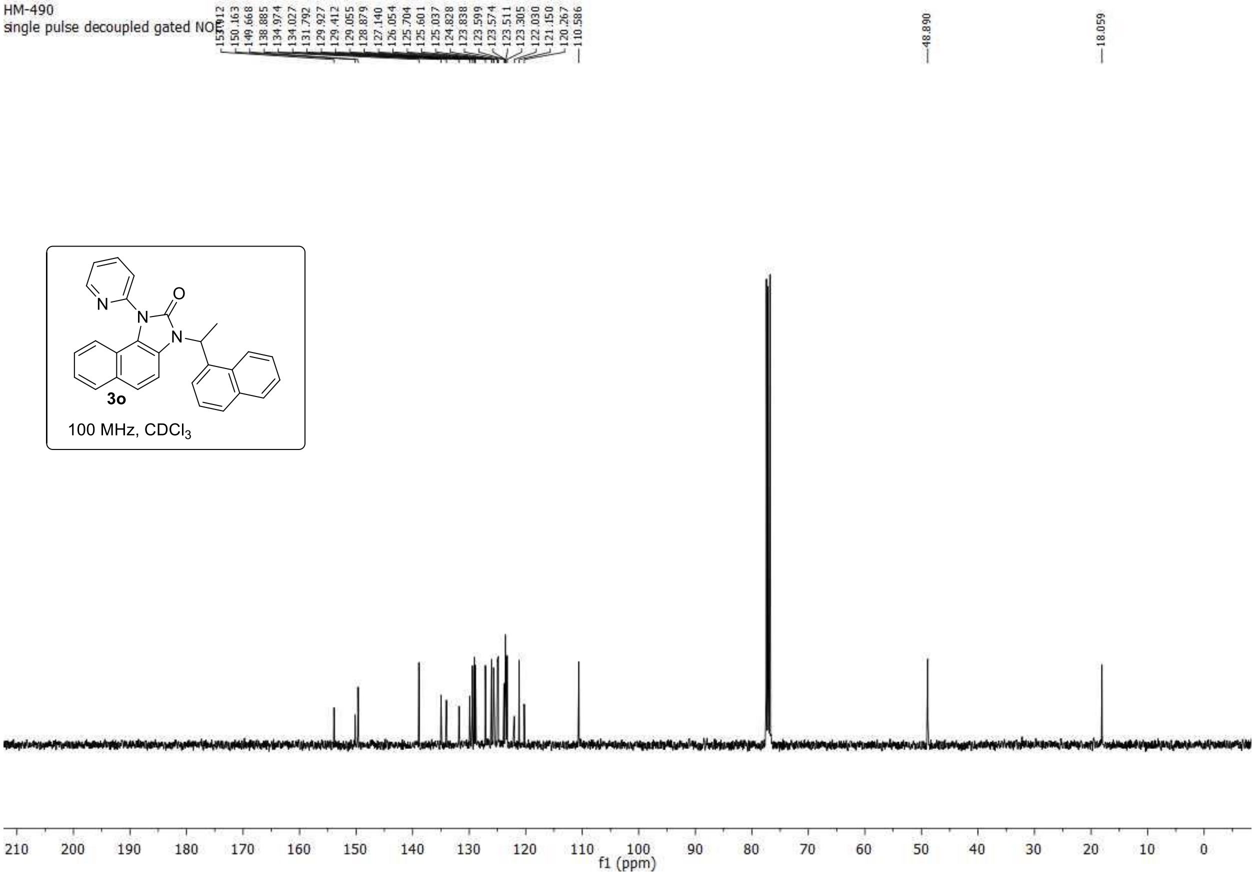
HM-490
single pulse decoupled gated NOE
153.912
150.163
149.668
138.885
134.974
134.027
131.792
129.927
129.412
129.055
128.879
127.140
126.054
125.704
125.601
125.037
124.828
123.838
123.599
123.574
123.511
123.305
122.030
121.150
120.267
110.586
48.890
18.059
3o
100 MHz, CDCl3
f1 (ppm)

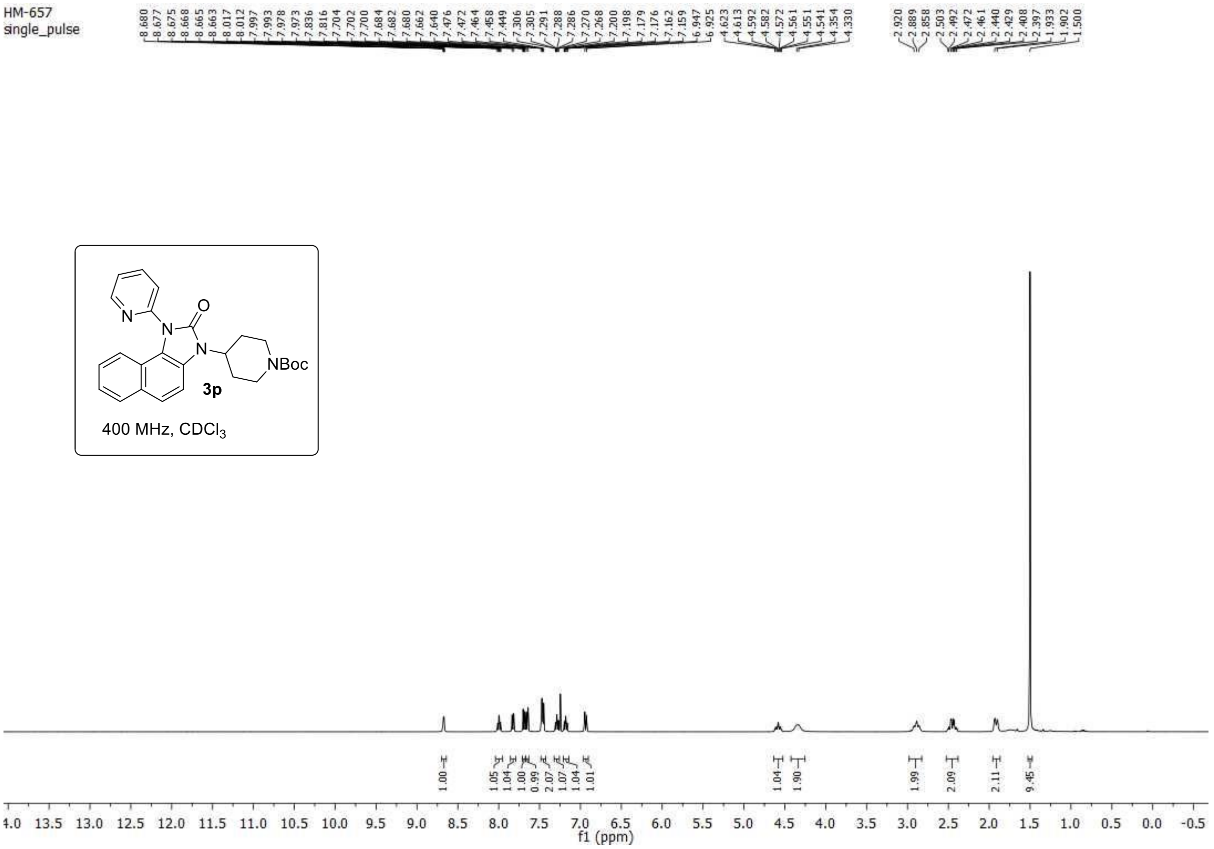
HM-657
single_pulse
8.680 8.677 8.675 8.668 8.665 8.663 8.017 8.012 7.997 7.993 7.978 7.973 7.836 7.816 7.704 7.702 7.700 7.684 7.682 7.680 7.662 7.640 7.476 7.472 7.464 7.458 7.449 7.306 7.305 7.291 7.288 7.286 7.270 7.268 7.200 7.198 7.179 7.176 7.162 7.159 6.947 6.925 4.623 4.613 4.592 4.582 4.572 4.561 4.551 4.541 4.354 4.330 2.920 2.889 2.858 2.503 2.492 2.472 2.461 2.440 2.429 2.408 2.397 1.933 1.902 1.500
N
O
N
N
NBoc
3p
400 MHz, CDCl3
1.00 1.05 1.04 1.00 0.99 2.07 1.07 1.04 1.01 1.04 1.90 1.99 2.09 2.11 9.45
f1 (ppm)

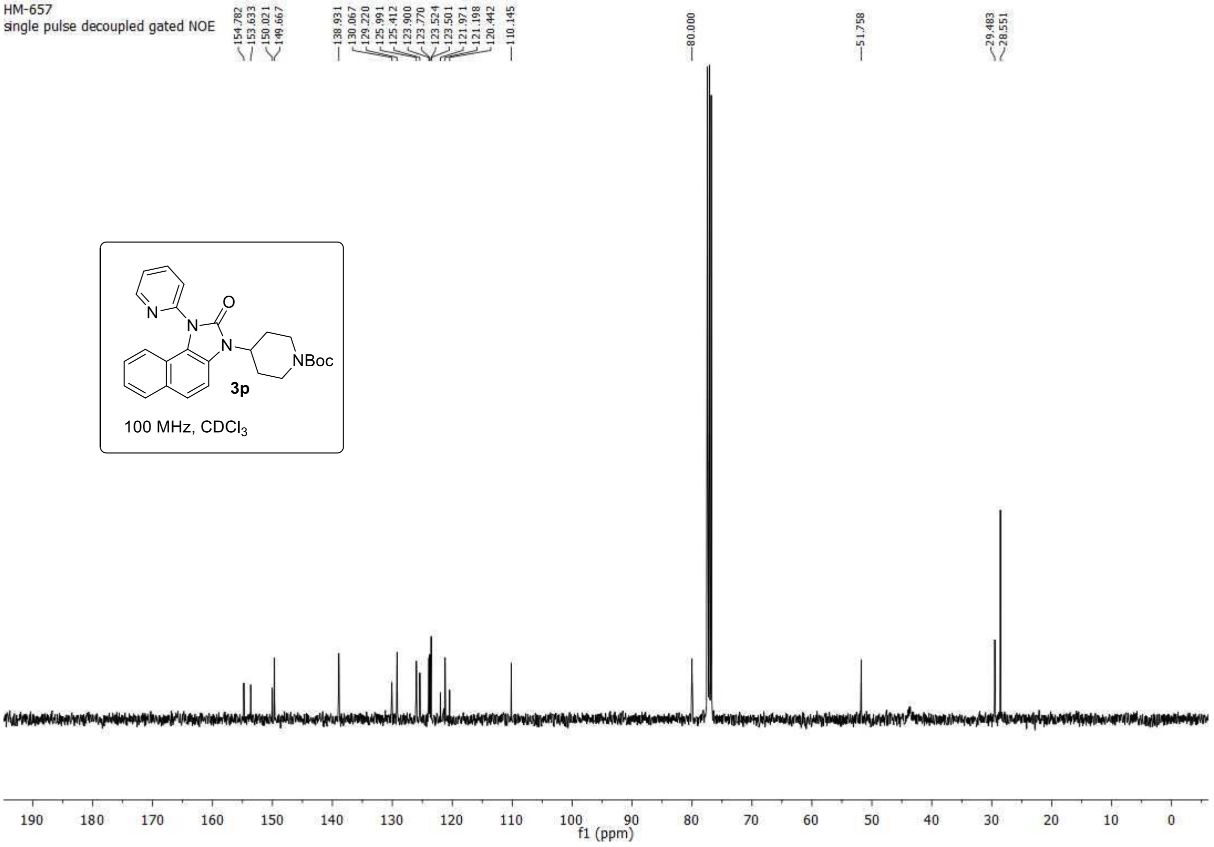
HM-657
single pulse decoupled gated NOE
154.782 153.633 150.021 149.667 138.931 130.067 129.220 125.991 125.412 123.900 123.770 123.524 123.501 121.971 121.198 120.442 110.145 80.000 51.758 29.483 28.551
N
O
N
N
NBoc
3p
100 MHz, CDCl3
f1 (ppm)

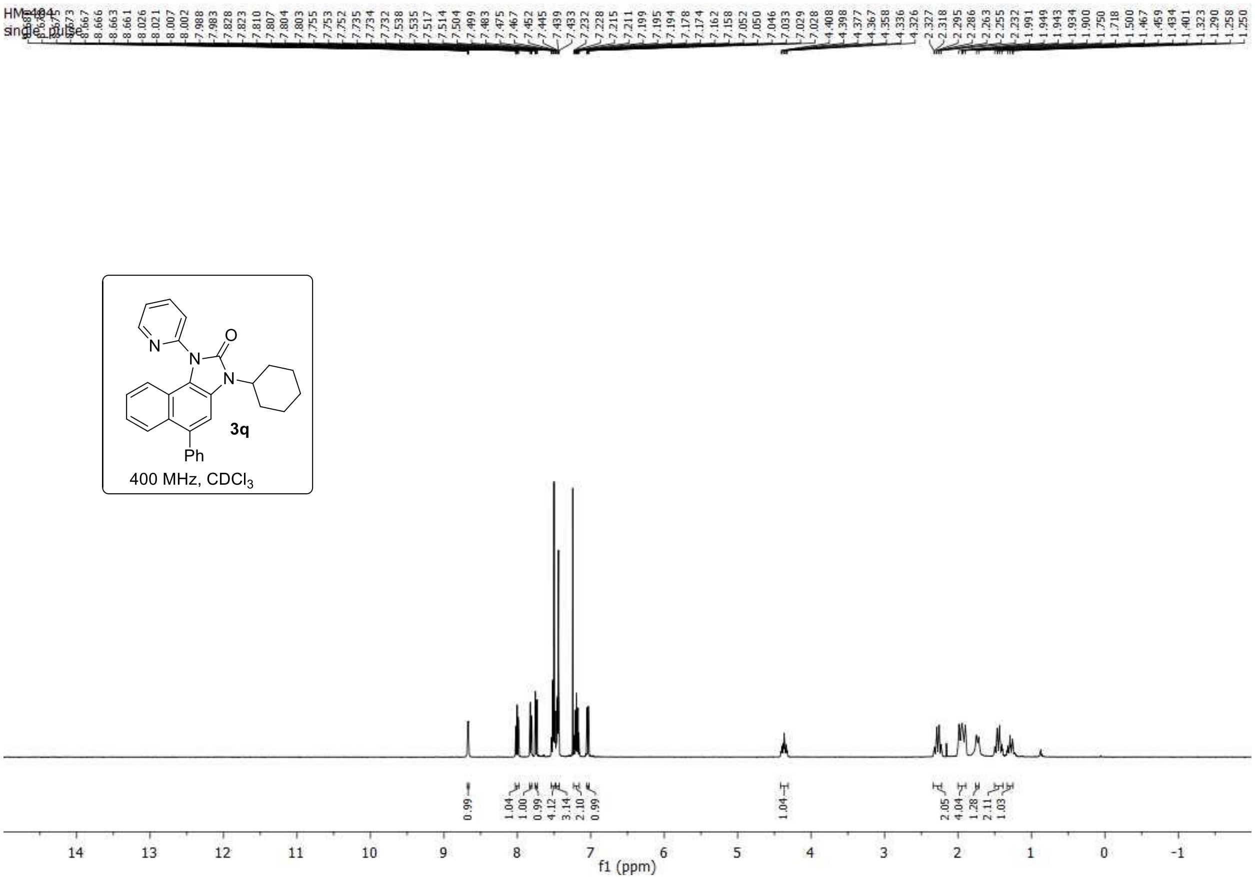
HM-404
single pulse
8.666 8.663 8.661 8.026 8.021 8.007 8.002 7.988 7.983 7.828 7.823 7.810 7.807 7.804 7.803 7.755 7.753 7.752 7.735 7.734 7.732 7.538 7.535 7.517 7.514 7.504 7.499 7.483 7.475 7.467 7.452 7.445 7.439 7.433 7.232 7.228 7.215 7.211 7.199 7.195 7.194 7.178 7.174 7.162 7.158 7.052 7.050 7.046 7.033 7.029 7.028 4.408 4.398 4.377 4.367 4.358 4.336 4.326 2.327 2.318 2.295 2.286 2.263 2.255 2.232 1.991 1.949 1.943 1.934 1.900 1.750 1.718 1.500 1.467 1.459 1.434 1.401 1.323 1.290 1.258 1.250
3q
400 MHz, $CDCl_3$
0.99 1.04 1.00 0.99 4.12 3.14 2.10 0.99 1.04 2.05 4.04 1.28 2.11 1.03
f1 (ppm)

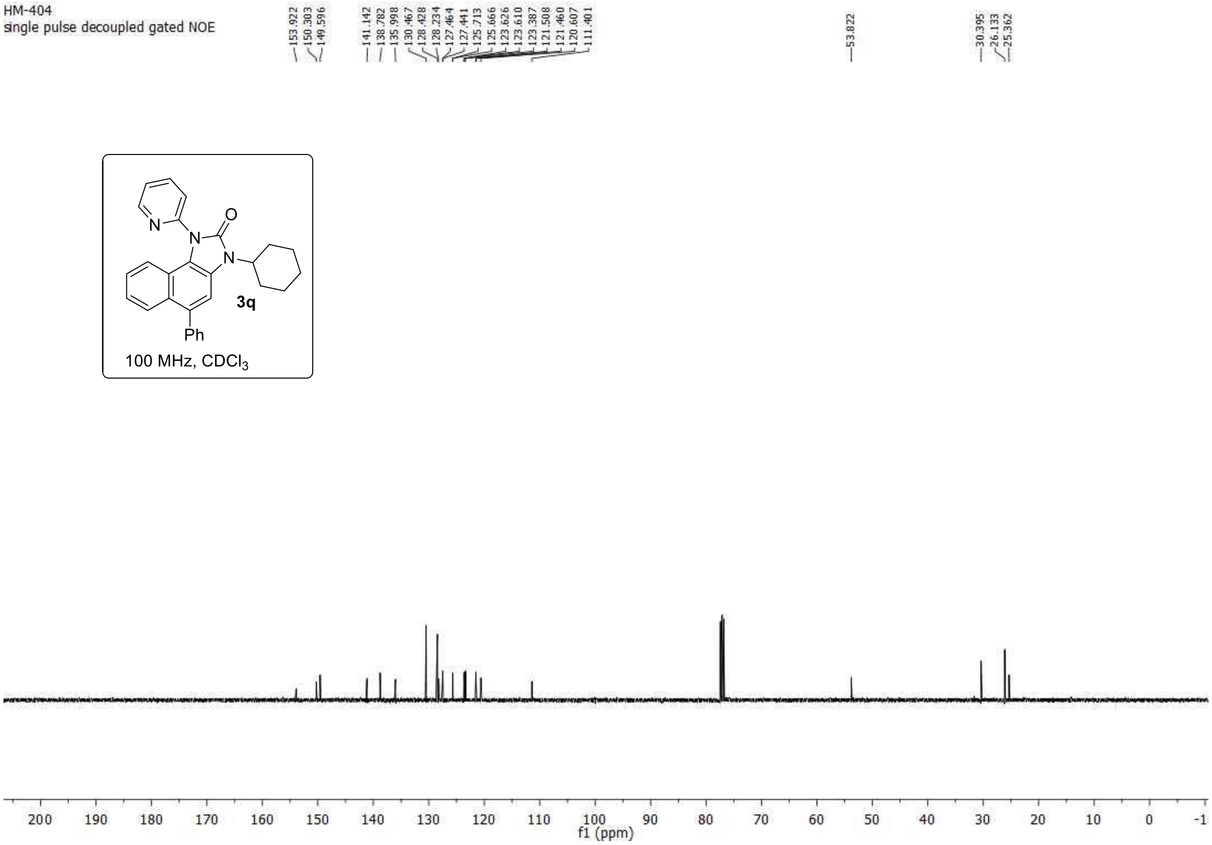
HM-404
single pulse decoupled gated NOE
153.922 150.303 149.596 141.142 138.782 135.998 130.467 128.428 128.234 127.464 127.441 125.713 125.666 123.626 123.610 123.387 121.508 121.460 120.607 111.401 53.822 30.395 26.133 25.362
3q
100 MHz, $CDCl_3$
f1 (ppm)

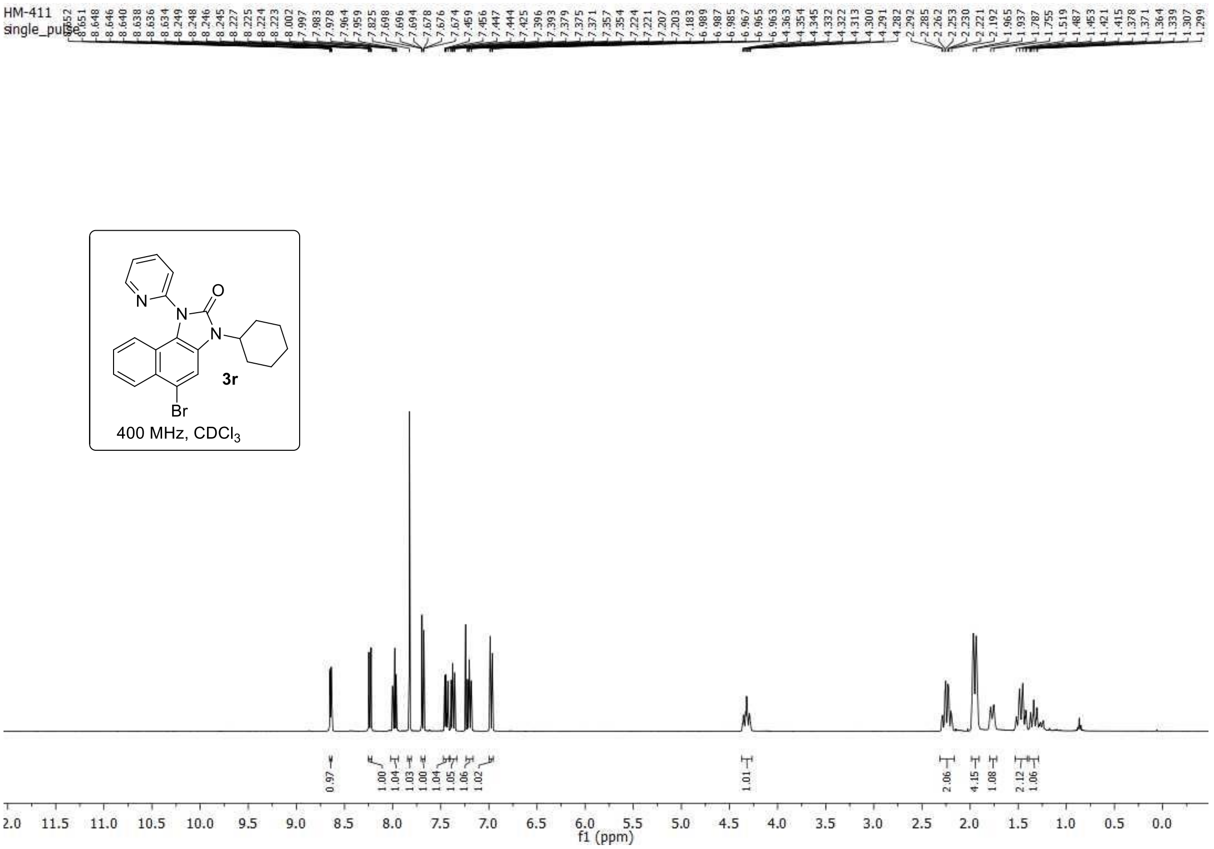
HM-411
single_pulse
8.652 8.651 8.648 8.646 8.640 8.638 8.636 8.634 8.249 8.248 8.246 8.245 8.227 8.225 8.224 8.223 8.002 7.997 7.983 7.978 7.964 7.959 7.825 7.698 7.696 7.694 7.678 7.676 7.674 7.459 7.456 7.447 7.444 7.425 7.396 7.393 7.379 7.375 7.371 7.357 7.354 7.224 7.221 7.207 7.203 7.183 6.989 6.987 6.985 6.967 6.965 6.963 4.363 4.354 4.345 4.332 4.322 4.313 4.300 4.291 4.282 2.292 2.285 2.262 2.253 2.230 2.221 2.192 1.965 1.937 1.787 1.755 1.519 1.487 1.453 1.421 1.415 1.378 1.371 1.364 1.339 1.307 1.299
N
O
N
N
3r
Br
400 MHz, $CDCl_3$
0.97 1.00 1.04 1.03 1.00 1.04 1.05 1.06 1.02 1.01 2.06 4.15 1.08 2.12 1.06
2.0 11.5 11.0 10.5 10.0 9.5 9.0 8.5 8.0 7.5 7.0 6.5 6.0 5.5 5.0 4.5 4.0 3.5 3.0 2.5 2.0 1.5 1.0 0.5 0.0
f1 (ppm)

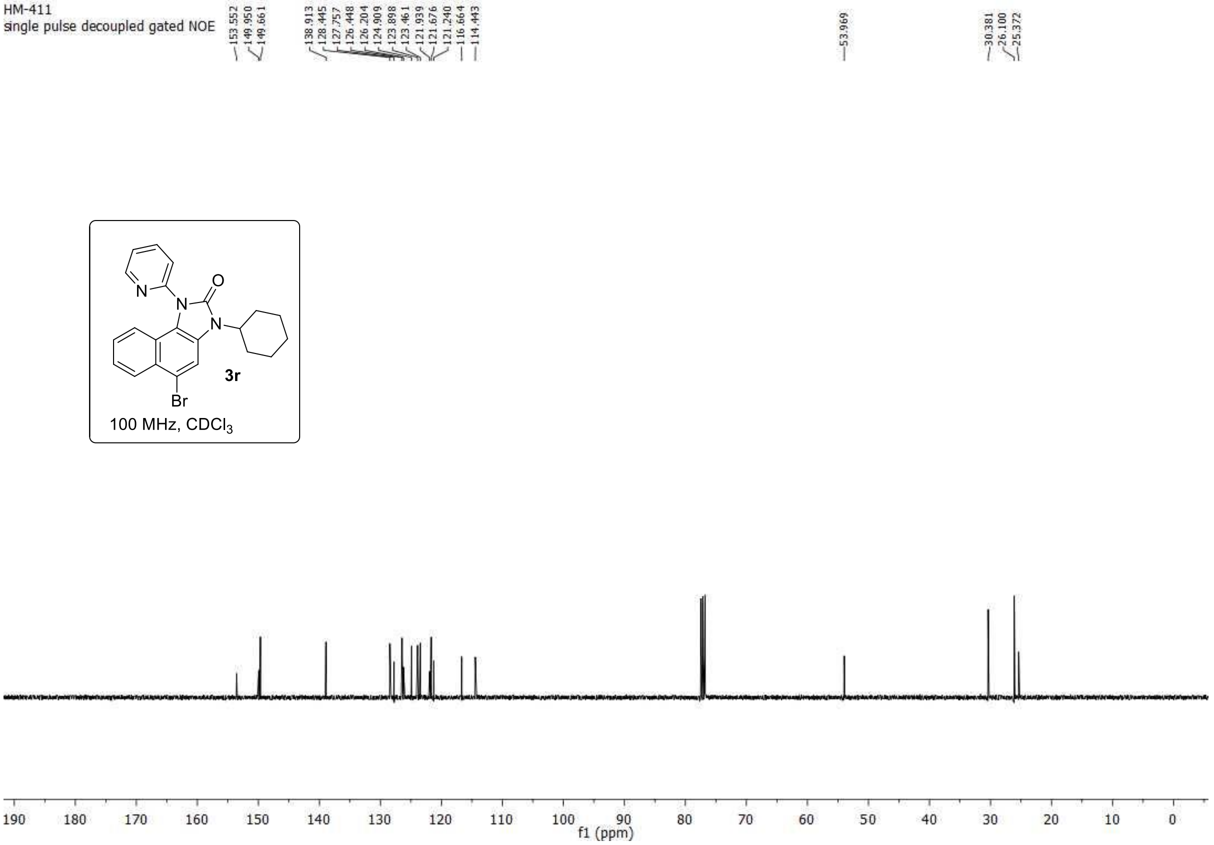
HM-411
single pulse decoupled gated NOE
153.552 149.950 149.661 138.913 128.445 127.757 126.448 126.204 124.909 123.898 123.461 121.939 121.676 121.240 116.664 114.443 53.969 30.381 26.100 25.372
N
O
N
N
3r
Br
100 MHz, $CDCl_3$
190 180 170 160 150 140 130 120 110 100 90 80 70 60 50 40 30 20 10 0
f1 (ppm)

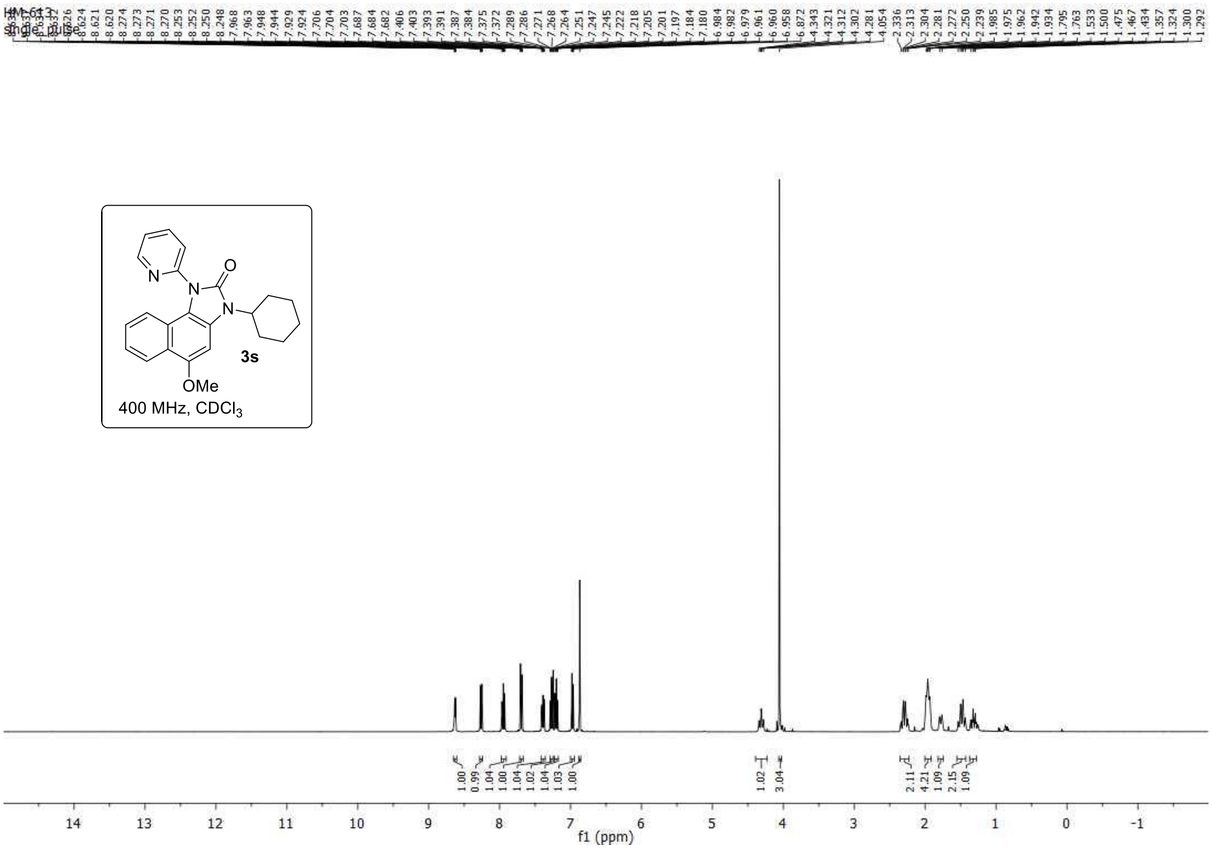
HM-613
single pulse
3s
OMe
400 MHz, CDCl3
1.00
0.99
1.04
1.00
1.04
1.02
1.04
1.03
1.00
1.02
3.04
2.11
4.21
1.09
2.15
1.09
f1 (ppm)

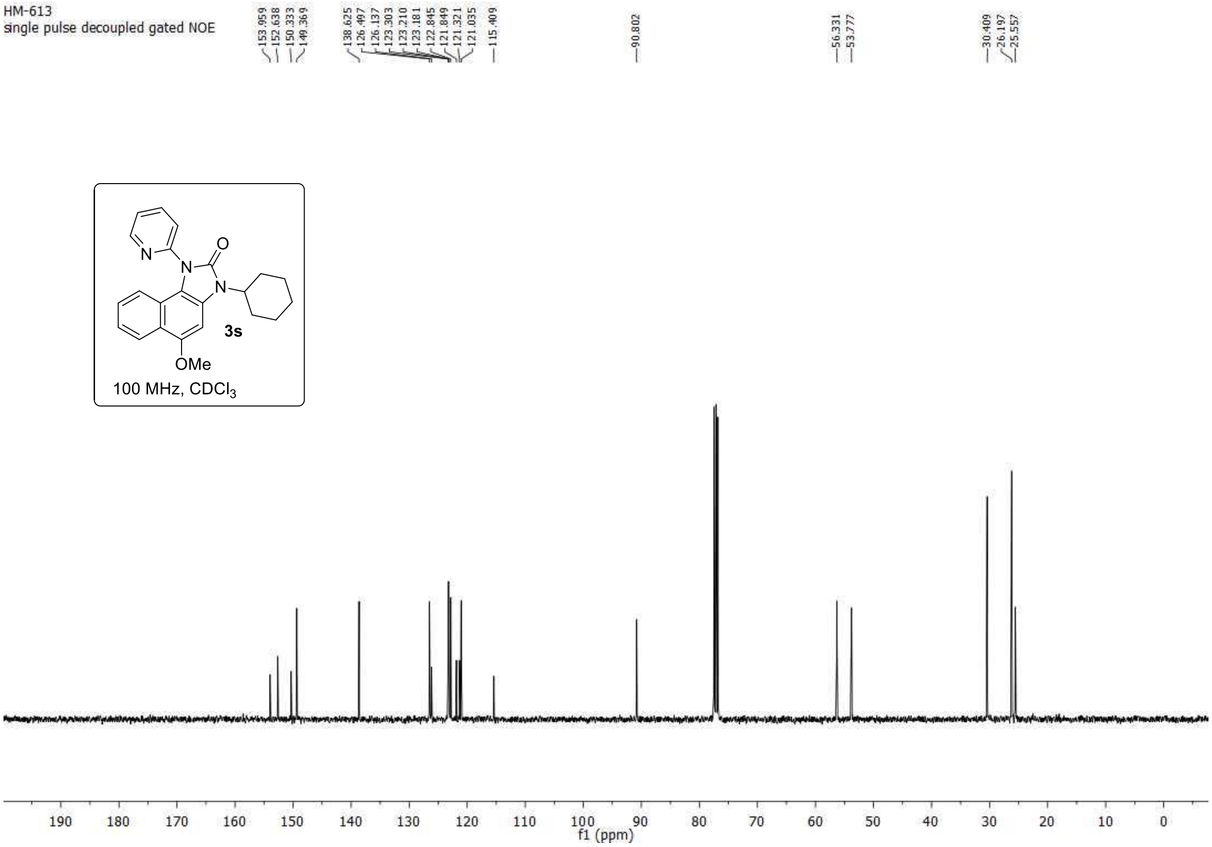
HM-613
single pulse decoupled gated NOE
153.959
152.638
150.333
149.369
138.625
126.497
126.137
123.303
123.210
123.181
122.845
121.849
121.321
121.035
115.409
90.802
56.331
53.777
30.409
26.197
25.557
3s
OMe
100 MHz, CDCl3
f1 (ppm)

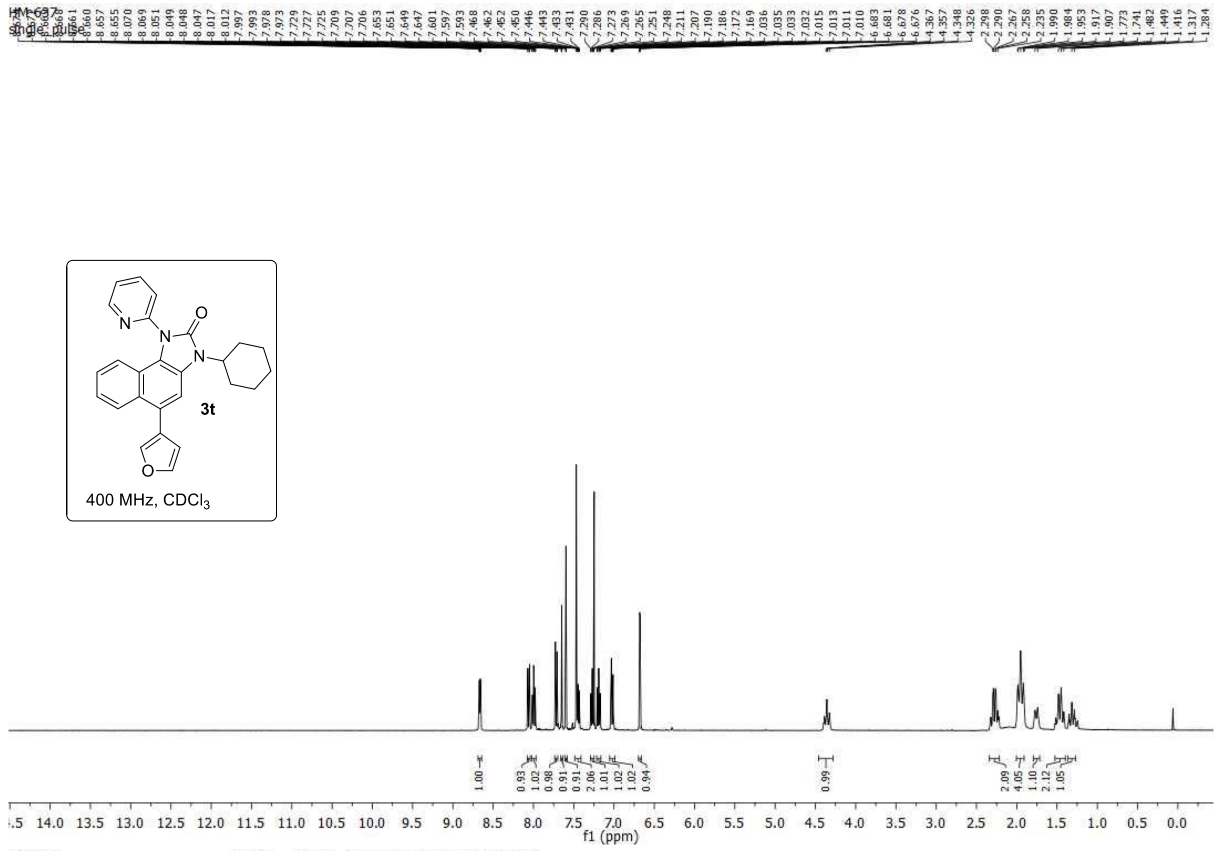

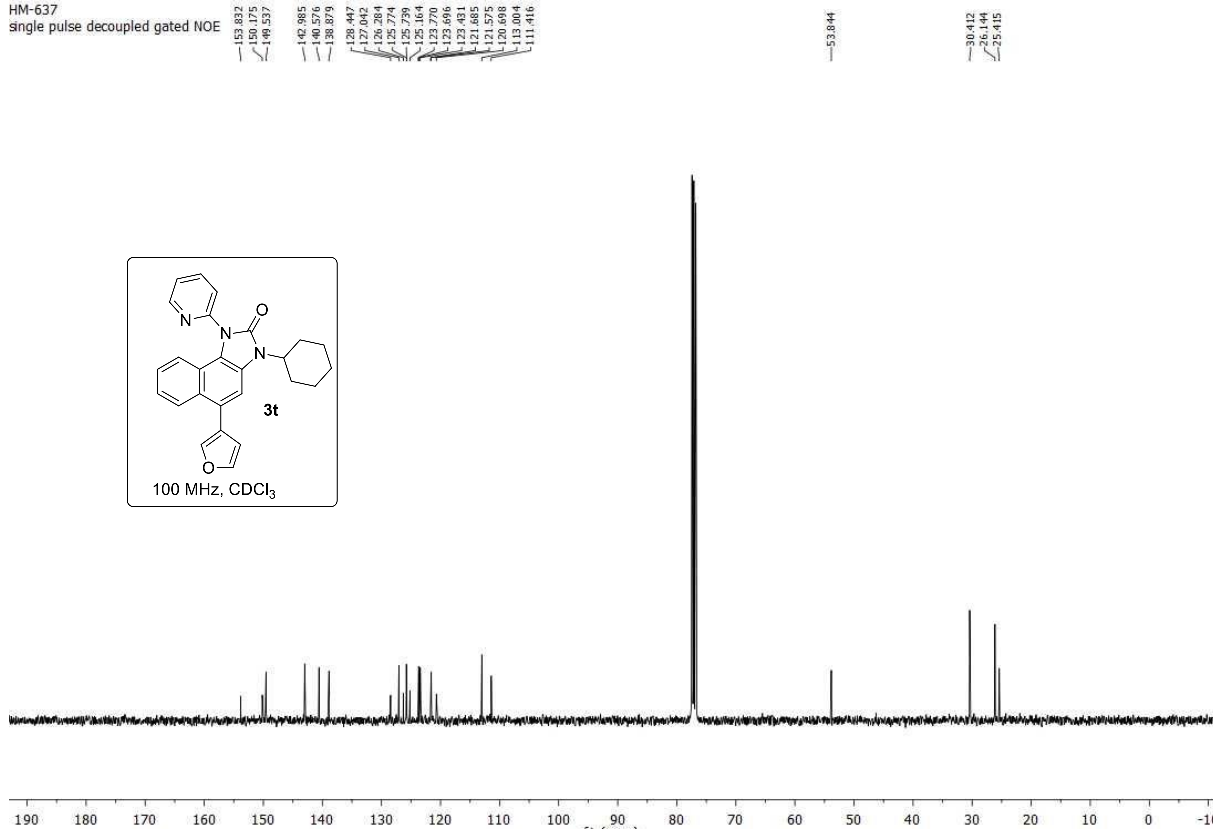

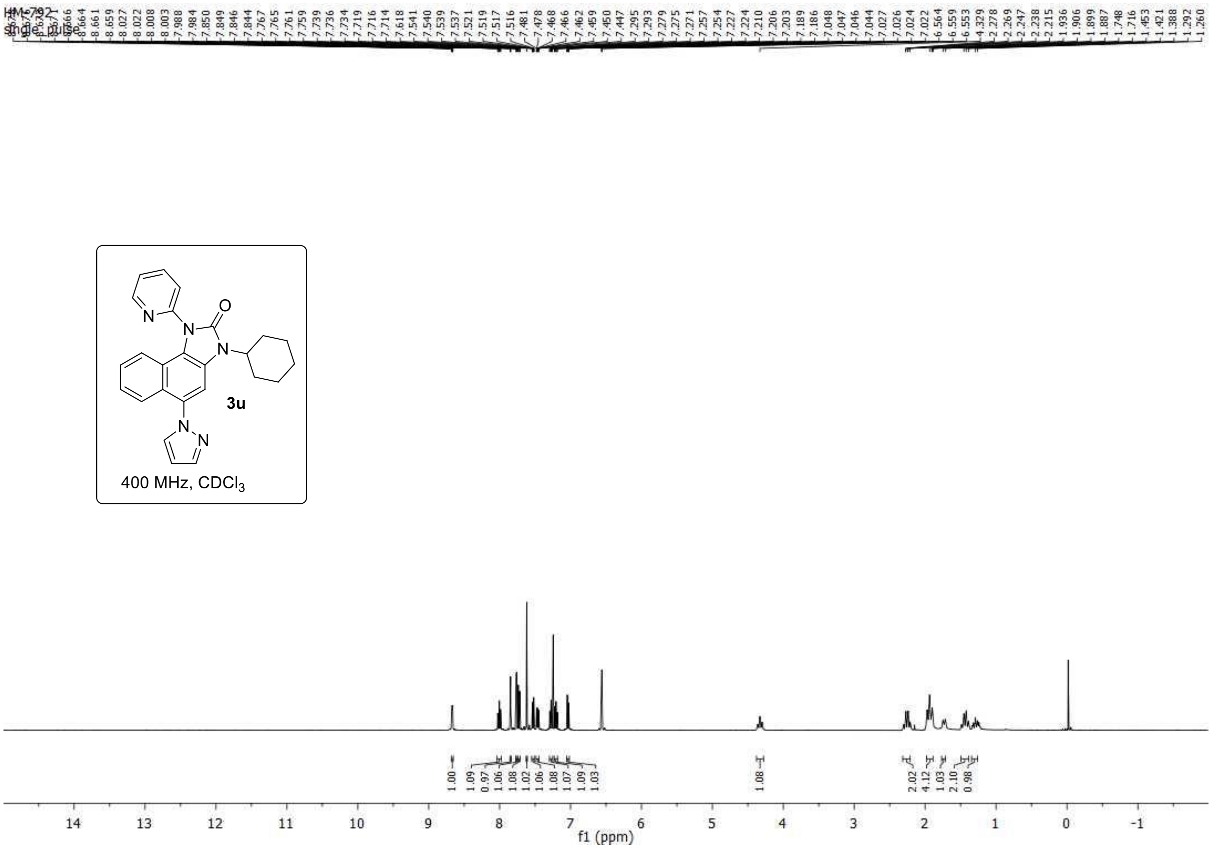
HM-792
single pulse
8.666
8.664
8.661
8.659
8.027
8.022
8.008
8.003
7.988
7.984
7.850
7.849
7.846
7.844
7.767
7.765
7.761
7.759
7.739
7.736
7.734
7.719
7.716
7.714
7.618
7.541
7.540
7.539
7.537
7.521
7.519
7.517
7.516
7.481
7.478
7.468
7.466
7.462
7.459
7.450
7.447
7.295
7.293
7.279
7.275
7.271
7.257
7.254
7.227
7.224
7.210
7.206
7.203
7.189
7.186
7.048
7.047
7.046
7.044
7.027
7.026
7.024
7.022
6.564
6.559
6.553
4.329
2.278
2.269
2.247
2.238
2.215
1.936
1.906
1.899
1.887
1.748
1.716
1.453
1.421
1.388
1.292
1.260
3u
400 MHz, CDCl3
1.00
1.09
0.97
1.06
1.08
1.02
1.06
1.08
1.07
1.09
1.03
1.08
2.02
4.12
1.03
2.10
0.98
f1 (ppm)

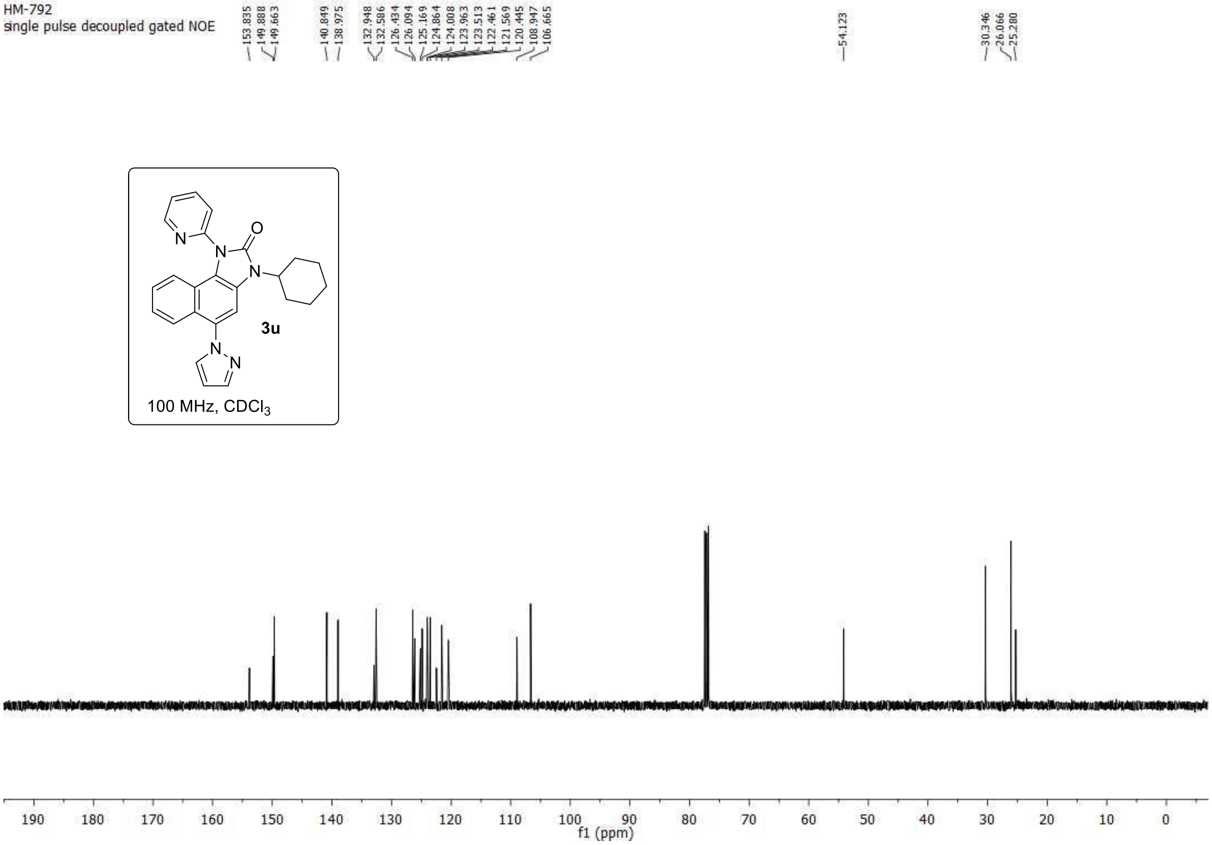
HM-792
single pulse decoupled gated NOE
153.835
149.888
149.663
140.849
138.975
132.948
132.586
126.434
126.094
125.169
124.864
124.008
123.963
123.513
122.461
121.569
120.445
108.947
106.665
54.123
30.346
26.066
25.280
3u
100 MHz, CDCl3
f1 (ppm)

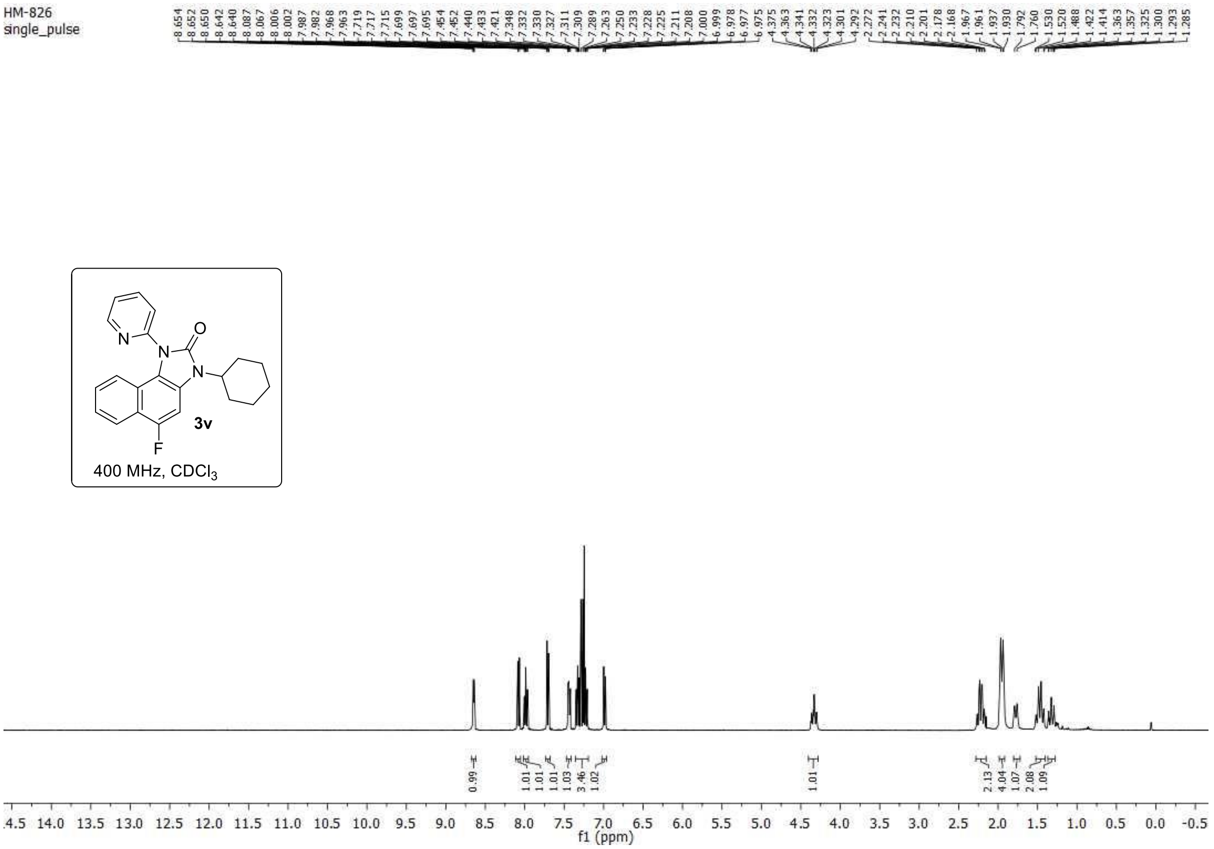
HM-826
single_pulse
8.654 8.652 8.650 8.642 8.640 8.087 8.067 8.006 8.002 7.987 7.982 7.968 7.963 7.719 7.717 7.715 7.699 7.697 7.695 7.454 7.452 7.440 7.433 7.421 7.348 7.332 7.330 7.327 7.311 7.309 7.289 7.263 7.250 7.233 7.228 7.225 7.211 7.208 7.000 6.999 6.978 6.977 6.975 4.375 4.363 4.341 4.332 4.323 4.301 4.292 2.272 2.241 2.232 2.210 2.201 2.178 2.168 1.967 1.961 1.937 1.930 1.792 1.760 1.530 1.520 1.488 1.422 1.414 1.363 1.357 1.325 1.300 1.293 1.285
N
O
N
N
F
3v
400 MHz, $CDCl_3$
0.99 1.01 1.01 1.01 1.03 3.46 1.02 1.01 2.13 4.04 1.07 2.08 1.09
14.5 14.0 13.5 13.0 12.5 12.0 11.5 11.0 10.5 10.0 9.5 9.0 8.5 8.0 7.5 7.0 6.5 6.0 5.5 5.0 4.5 4.0 3.5 3.0 2.5 2.0 1.5 1.0 0.5 0.0 -0.5
f1 (ppm)

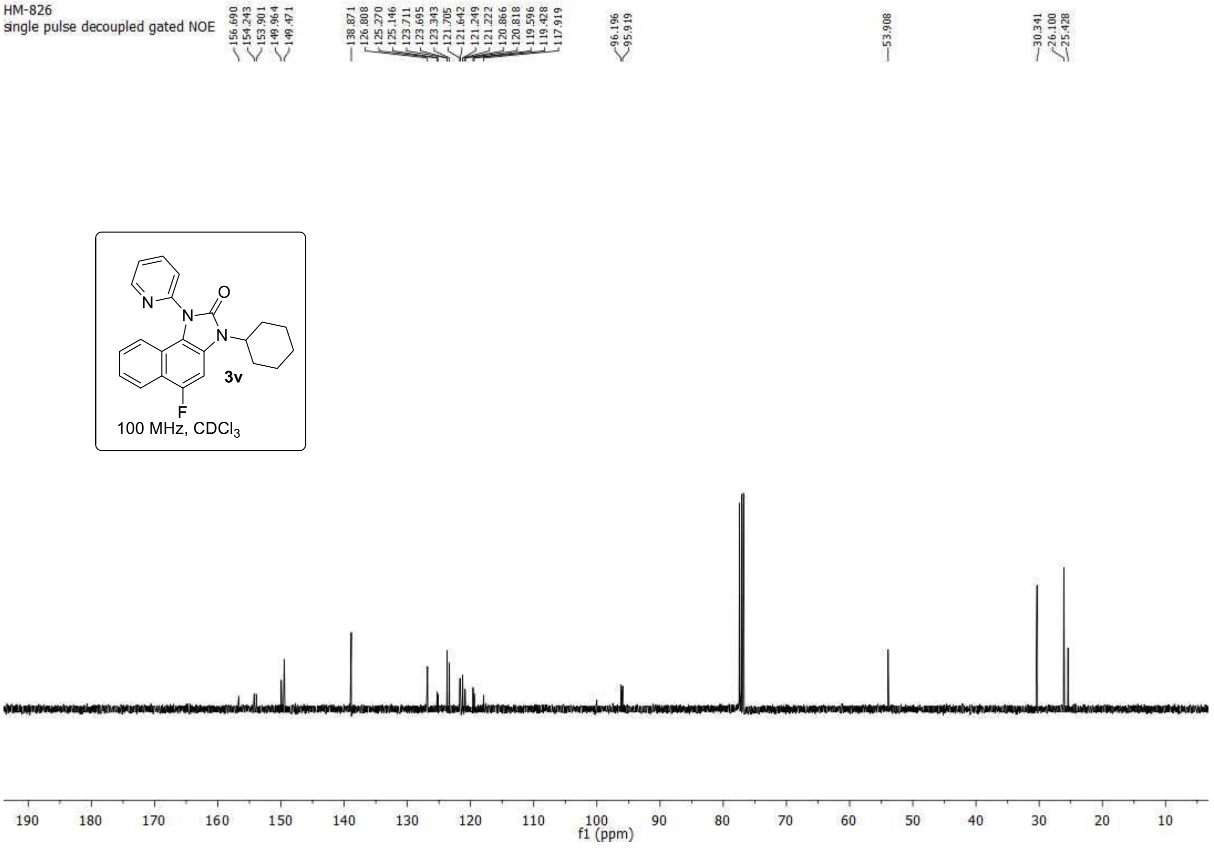
HM-826
single pulse decoupled gated NOE
156.690 154.243 153.901 149.964 149.471 138.871 126.808 125.270 125.146 123.711 123.695 123.343 121.705 121.642 121.249 121.222 120.866 120.818 119.596 119.428 117.919 96.196 95.919 53.908 30.341 26.100 25.428
N
O
N
N
F
3v
100 MHz, $CDCl_3$
190 180 170 160 150 140 130 120 110 100 90 80 70 60 50 40 30 20 10
f1 (ppm)

HM-826
single pulse decoupled gated NOE

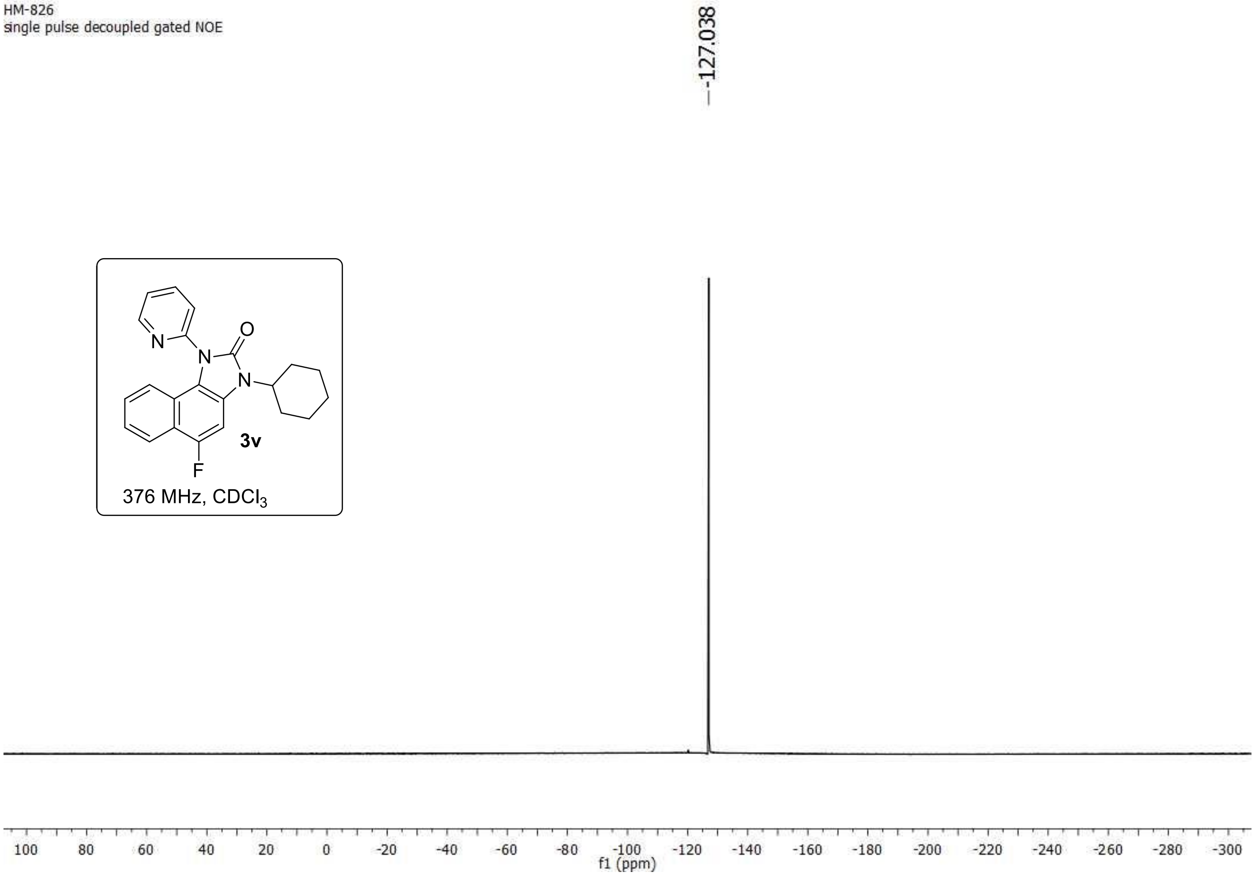

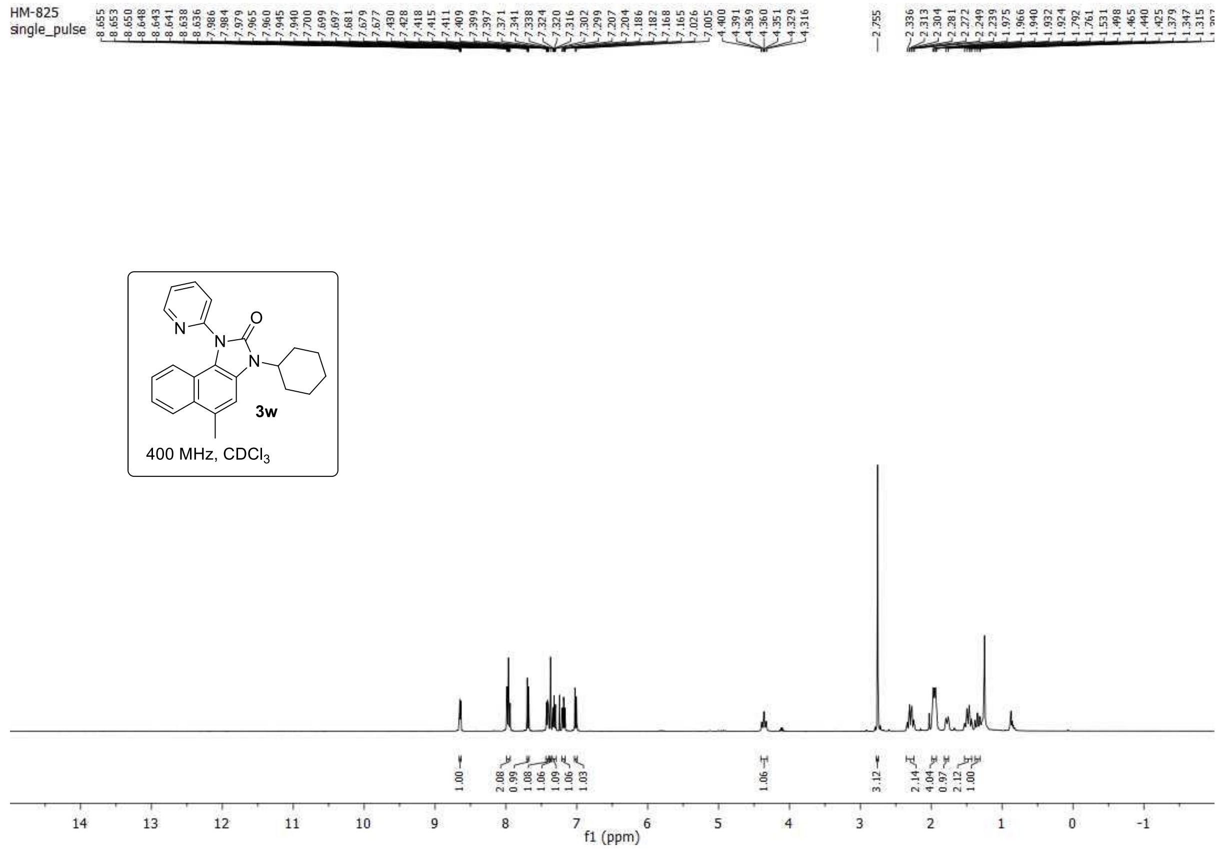
HM-825
single_pulse
8.655 8.653 8.650 8.648 8.643 8.641 8.638 8.636 7.986 7.984 7.979 7.965 7.960 7.945 7.940 7.700 7.699 7.697 7.681 7.679 7.677 7.430 7.428 7.418 7.415 7.411 7.409 7.399 7.397 7.371 7.341 7.338 7.324 7.320 7.316 7.302 7.299 7.207 7.204 7.186 7.182 7.168 7.165 7.026 7.005 4.400 4.391 4.369 4.360 4.351 4.329 4.316
2.755
2.336 2.313 2.304 2.281 2.272 2.249 2.239 1.975 1.966 1.940 1.932 1.924 1.792 1.761 1.531 1.498 1.465 1.440 1.425 1.379 1.347 1.315
N
O
N
N
3w
400 MHz, CDCl$_3$
1.00
2.08 0.99 1.08 1.06 1.09 1.06 1.03
1.06
3.12
2.14 4.04 0.97 2.12 1.00
14 13 12 11 10 9 8 7 6 5 4 3 2 1 0 -1
f1 (ppm)

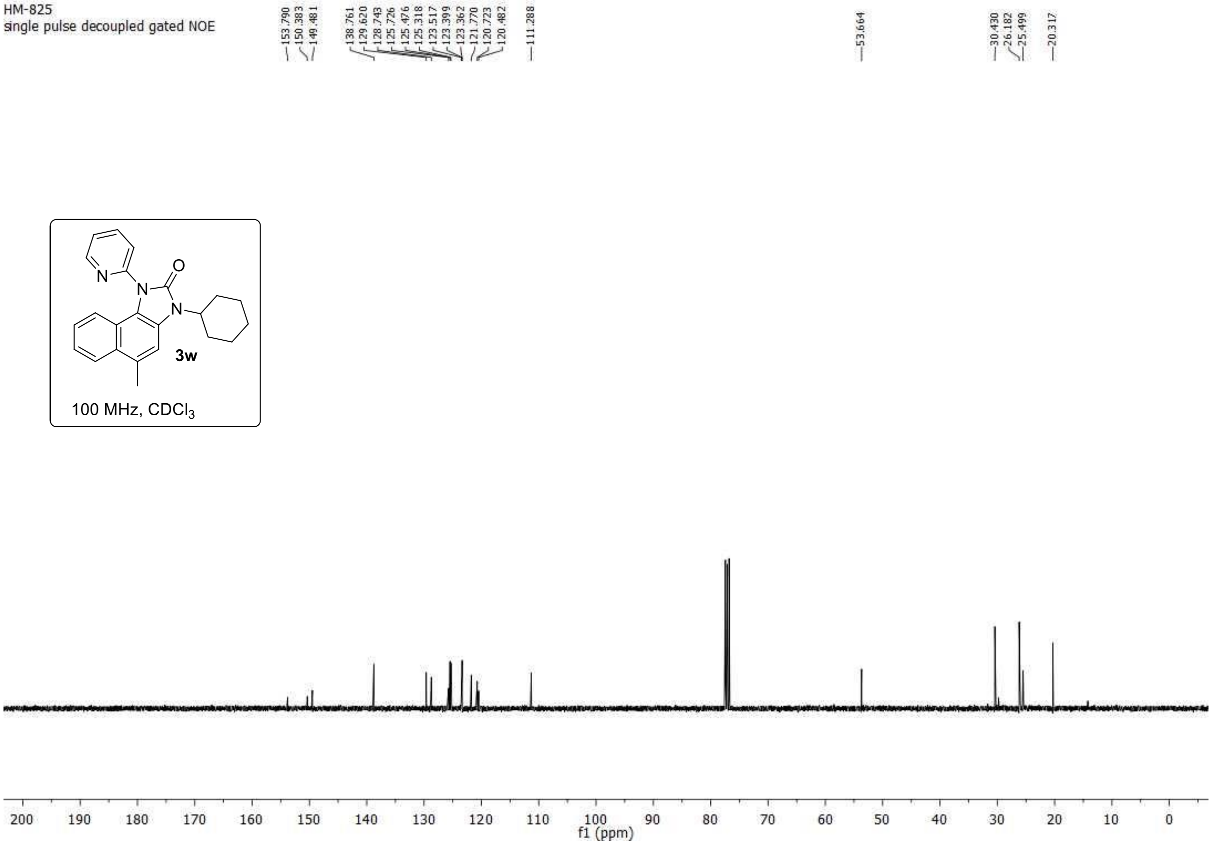
HM-825
single pulse decoupled gated NOE
153.790 150.383 149.481
138.761 129.620 128.743 125.726 125.476 125.318 123.517 123.399 123.362 121.770 120.723 120.482
111.288
53.664
30.430 26.182 25.499
20.317
N
O
N
N
3w
100 MHz, CDCl$_3$
200 190 180 170 160 150 140 130 120 110 100 90 80 70 60 50 40 30 20 10 0
f1 (ppm)

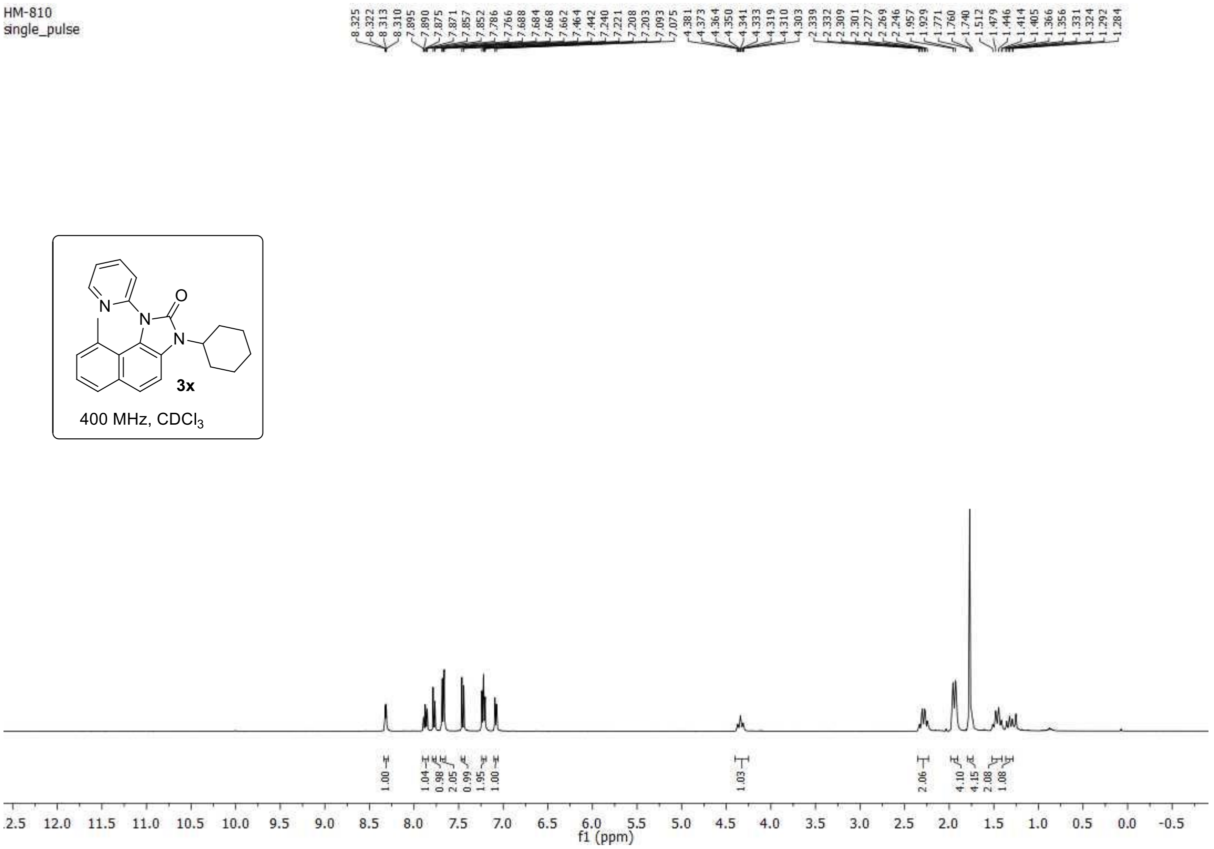
HM-810
single_pulse
8.325 8.322 8.313 8.310 7.895 7.890 7.875 7.871 7.857 7.852 7.786 7.766 7.688 7.684 7.668 7.662 7.464 7.442 7.240 7.221 7.208 7.203 7.093 7.075 4.381 4.373 4.364 4.350 4.341 4.333 4.319 4.310 4.303 2.339 2.332 2.309 2.301 2.277 2.269 2.246 1.957 1.929 1.771 1.760 1.740 1.512 1.479 1.446 1.414 1.405 1.366 1.356 1.331 1.324 1.292 1.284
3x
400 MHz, CDCl3
1.00 1.04 0.98 2.05 0.99 1.95 1.00 1.03 2.06 4.10 4.15 2.08 1.08
f1 (ppm)

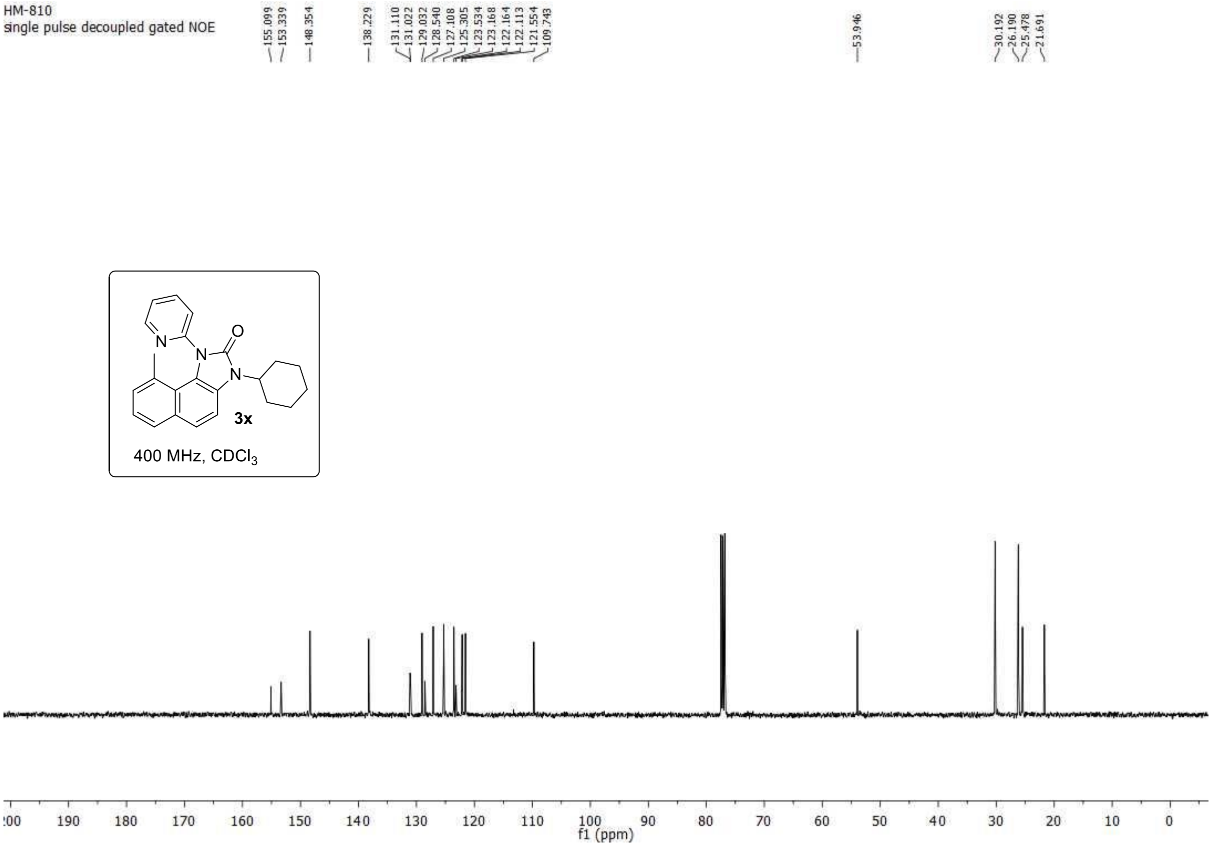
HM-810
single pulse decoupled gated NOE
155.099 153.339 148.354 138.229 131.110 131.022 129.032 128.540 127.108 125.305 123.534 123.168 122.164 122.113 121.554 109.743 53.946 30.192 26.190 25.478 21.691
3x
400 MHz, CDCl3
f1 (ppm)

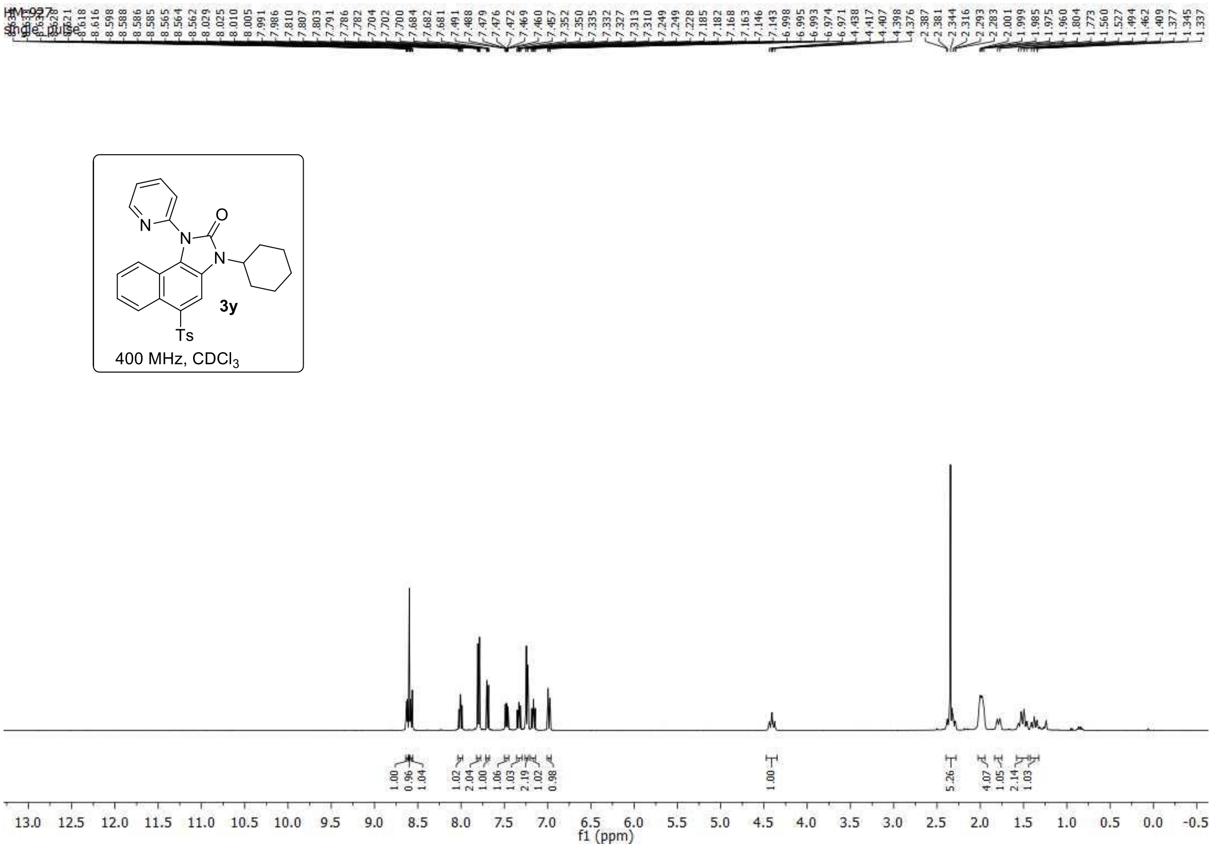
HM-927
single pulse
8.618 8.616 8.598 8.588 8.586 8.585 8.565 8.564 8.562 8.029 8.025 8.010 8.005 7.991 7.986 7.810 7.807 7.803 7.791 7.786 7.782 7.704 7.702 7.700 7.684 7.682 7.681 7.491 7.488 7.479 7.476 7.472 7.469 7.460 7.457 7.352 7.350 7.335 7.332 7.327 7.313 7.310 7.249 7.249 7.228 7.185 7.182 7.168 7.163 7.146 7.143 6.998 6.995 6.993 6.974 6.971 4.438 4.417 4.407 4.398 4.376 2.387 2.381 2.344 2.316 2.293 2.283 2.001 1.999 1.985 1.975 1.960 1.804 1.773 1.560 1.527 1.494 1.462 1.409 1.377 1.345 1.337
N
O
N
N
3y
Ts
400 MHz, CDCl3
1.00 0.96 1.04 1.02 2.04 1.00 1.06 1.03 2.19 1.02 0.98 1.00 5.26 4.07 1.05 2.14 1.03
13.0 12.5 12.0 11.5 11.0 10.5 10.0 9.5 9.0 8.5 8.0 7.5 7.0 6.5 6.0 5.5 5.0 4.5 4.0 3.5 3.0 2.5 2.0 1.5 1.0 0.5 0.0 -0.5
f1 (ppm)

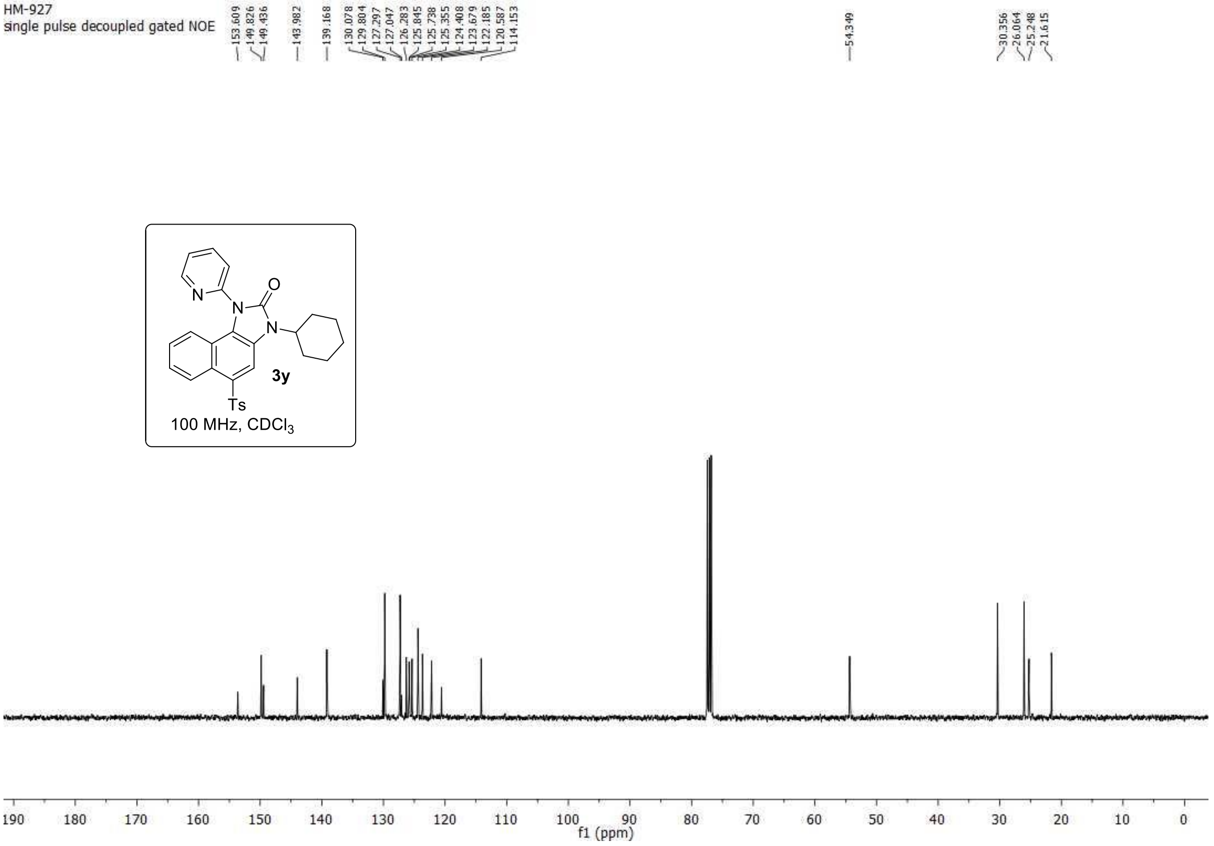
HM-927
single pulse decoupled gated NOE
153.609 149.826 149.436 143.982 139.168 130.078 129.804 127.297 127.047 126.283 125.845 125.738 125.355 124.408 123.679 122.185 120.587 114.153 54.349 30.356 26.064 25.248 21.615
N
O
N
N
3y
Ts
100 MHz, CDCl3
190 180 170 160 150 140 130 120 110 100 90 80 70 60 50 40 30 20 10 0
f1 (ppm)

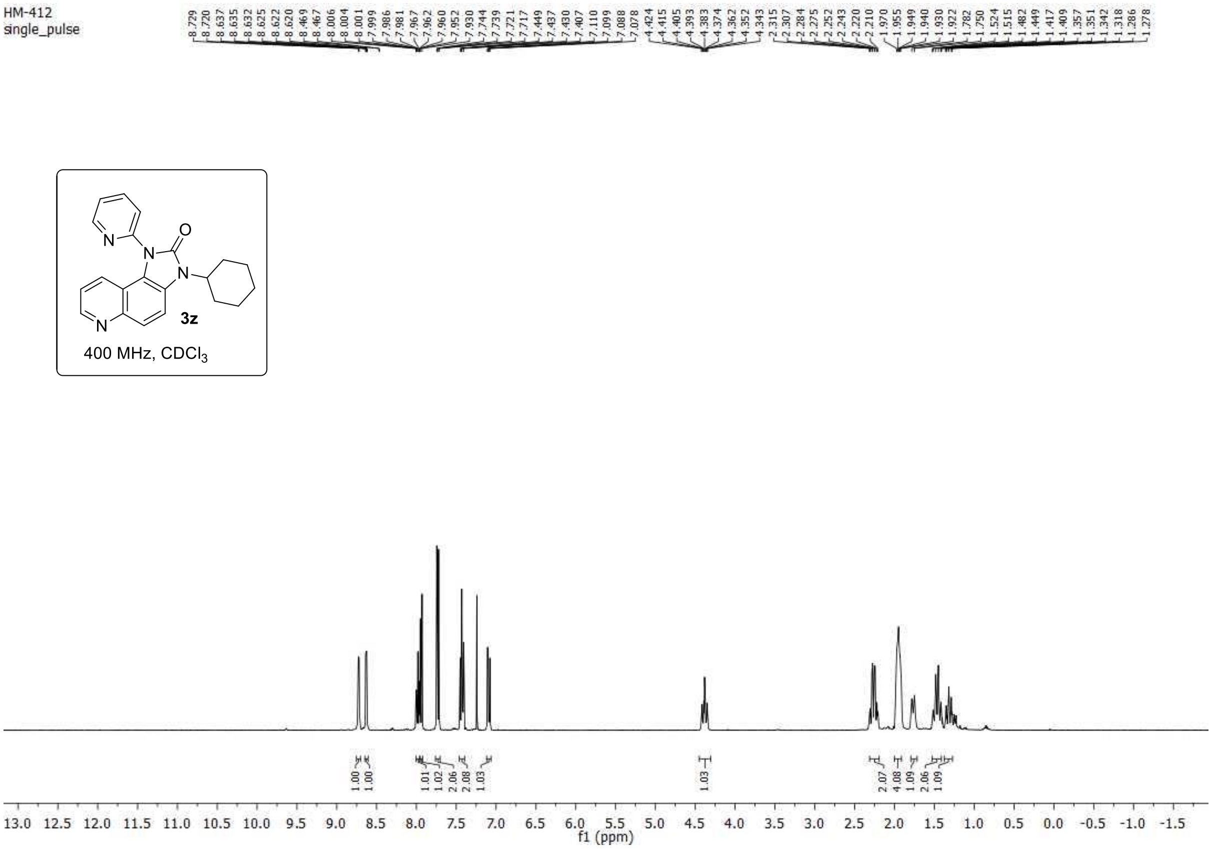
HM-412
single_pulse
8.729
8.720
8.637
8.635
8.632
8.625
8.622
8.620
8.469
8.467
8.006
8.004
8.001
7.999
7.986
7.981
7.967
7.962
7.960
7.952
7.930
7.744
7.739
7.721
7.717
7.449
7.437
7.430
7.407
7.110
7.099
7.088
7.078
4.424
4.415
4.405
4.393
4.383
4.374
4.362
4.352
4.343
2.315
2.307
2.284
2.275
2.252
2.243
2.220
2.210
1.970
1.955
1.949
1.940
1.930
1.922
1.782
1.750
1.524
1.515
1.482
1.449
1.417
1.409
1.357
1.351
1.342
1.318
1.286
1.278
3z
400 MHz, CDCl3
1.00
1.00
1.01
1.02
2.06
2.08
1.03
1.03
2.07
4.08
1.09
2.06
1.09
f1 (ppm)

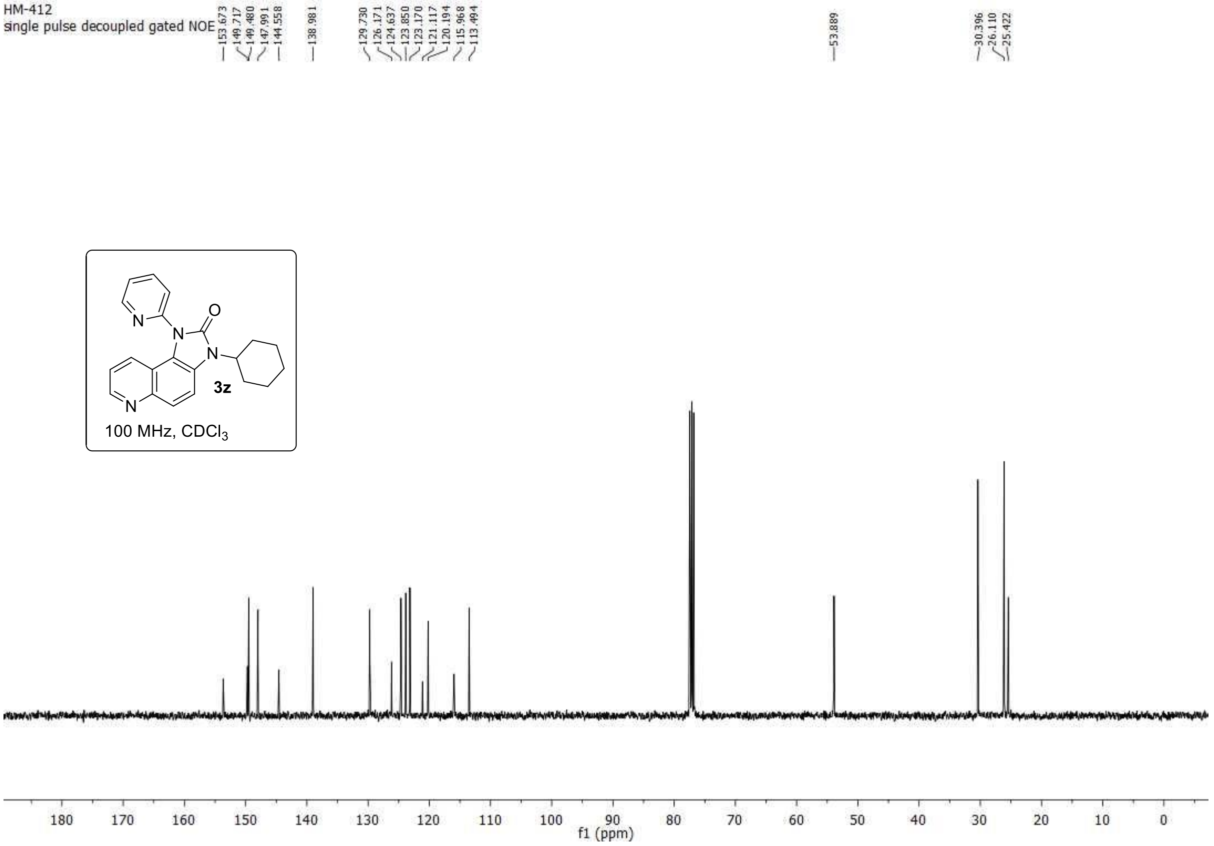
HM-412
single pulse decoupled gated NOE
153.673
149.717
149.480
147.991
144.558
138.981
129.730
126.171
124.637
123.850
123.170
121.117
120.194
115.968
113.494
53.889
30.396
26.110
25.422
3z
100 MHz, CDCl3
f1 (ppm)

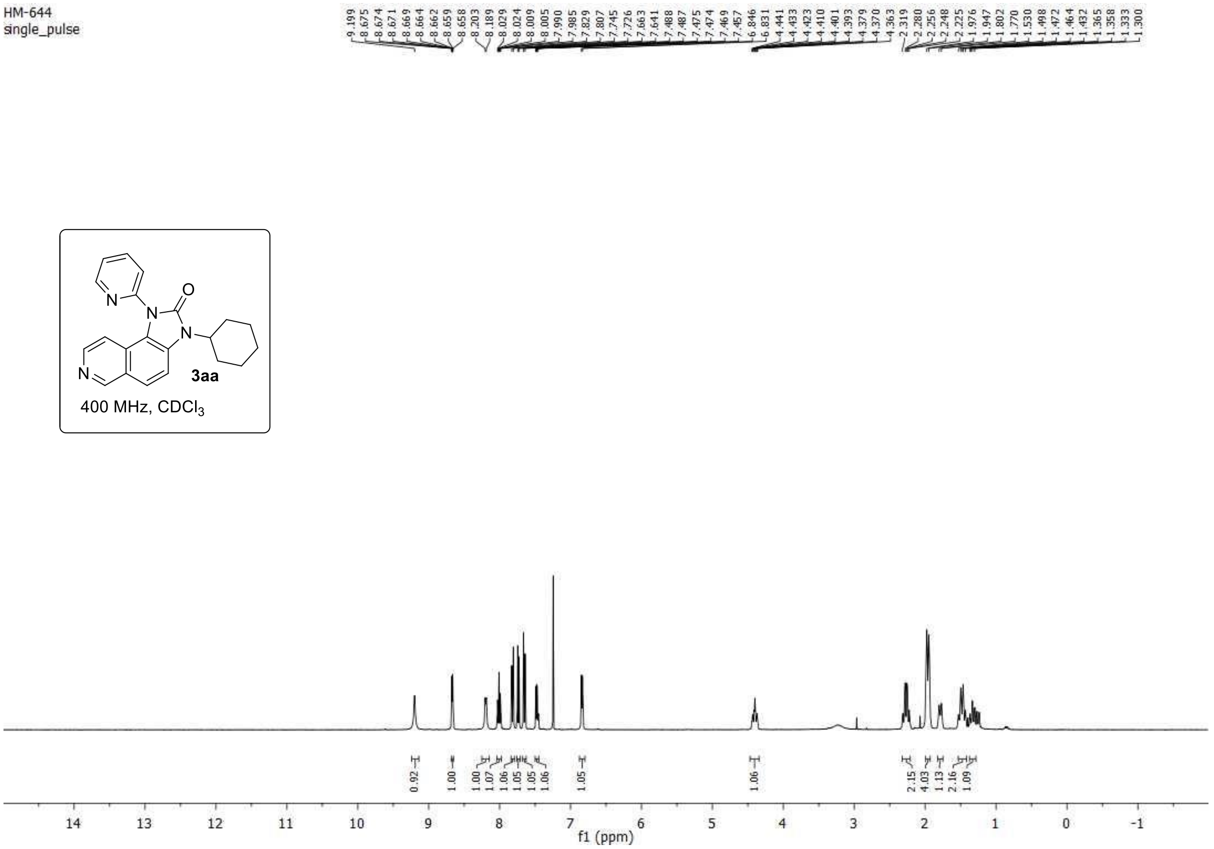
HM-644
single_pulse
9.199 8.675 8.674 8.671 8.669 8.664 8.662 8.659 8.658 8.203 8.189 8.029 8.024 8.009 8.005 7.990 7.985 7.829 7.807 7.745 7.726 7.663 7.641 7.488 7.487 7.475 7.474 7.469 7.457 6.846 6.831 4.441 4.433 4.423 4.410 4.401 4.393 4.379 4.370 4.363 2.319 2.280 2.256 2.248 2.225 1.976 1.947 1.802 1.770 1.530 1.498 1.472 1.464 1.432 1.365 1.358 1.333 1.300
3aa
400 MHz, CDCl3
0.92 1.00 1.00 1.07 1.06 1.05 1.05 1.06 1.05 1.06 2.15 4.03 1.13 2.16 1.09
f1 (ppm)

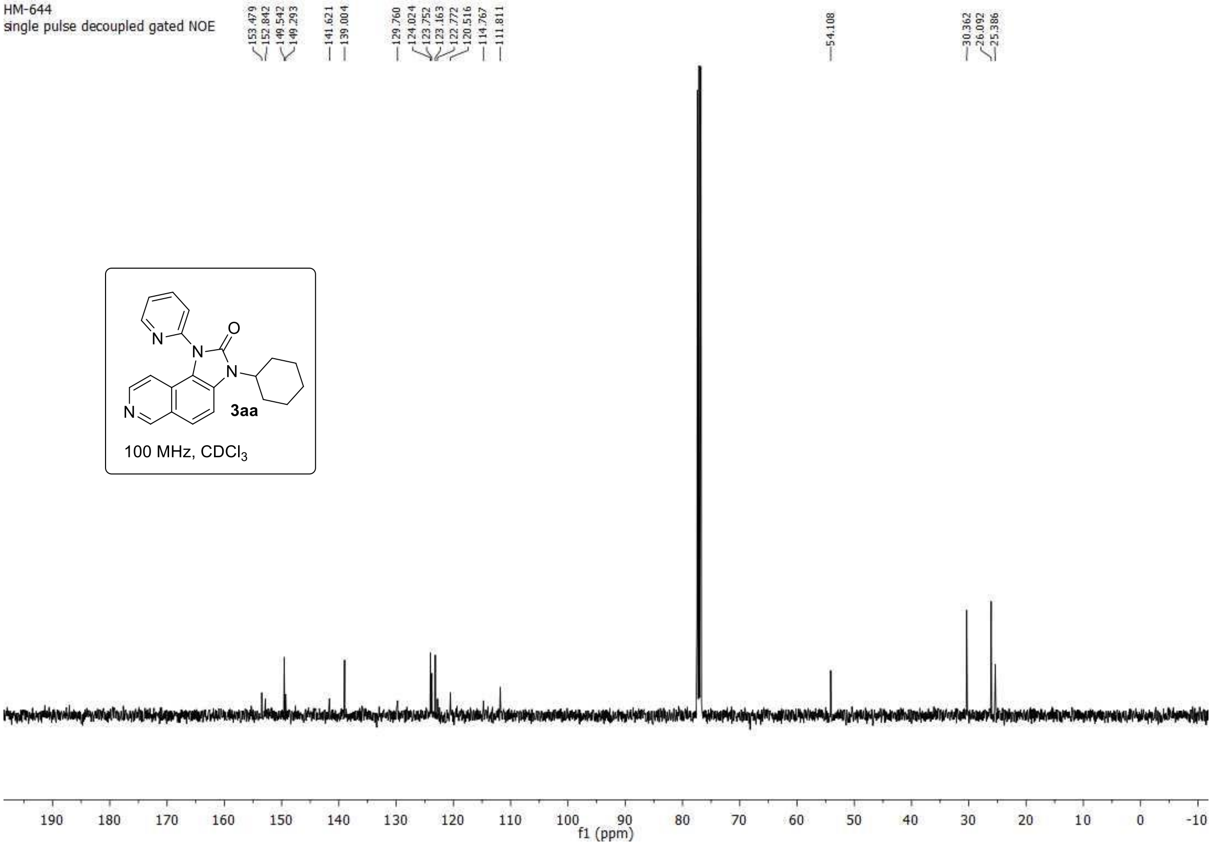
HM-644
single pulse decoupled gated NOE
153.479 152.842 149.542 149.293 141.621 139.004 129.760 124.024 123.752 123.163 122.772 120.516 114.767 111.811 54.108 30.362 26.092 25.386
3aa
100 MHz, CDCl3
f1 (ppm)

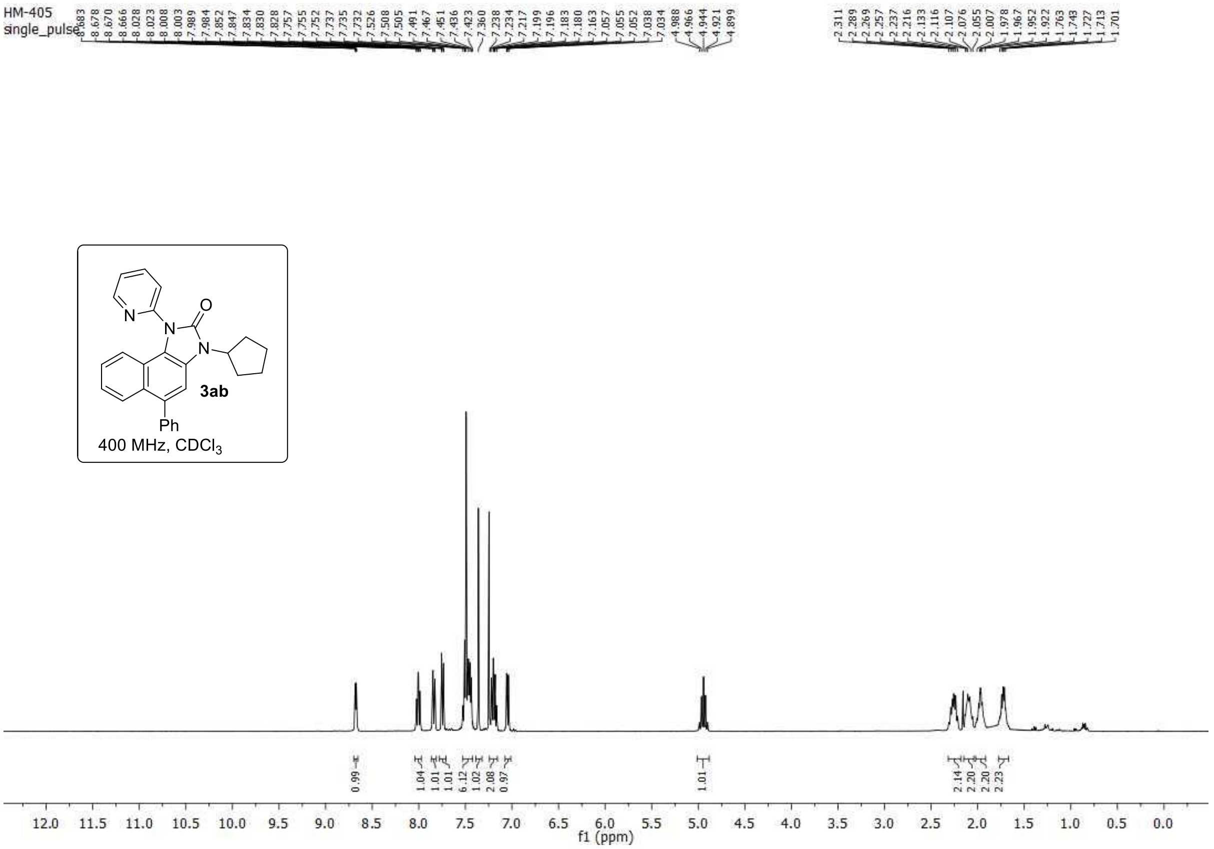
HM-405
single_pulse
8.683 8.678 8.670 8.666 8.028 8.023 8.008 8.003 7.989 7.984 7.852 7.847 7.834 7.830 7.828 7.757 7.755 7.752 7.737 7.735 7.732 7.526 7.508 7.505 7.491 7.467 7.451 7.436 7.423 7.360 7.238 7.234 7.217 7.199 7.196 7.183 7.180 7.163 7.057 7.055 7.052 7.038 7.034 4.988 4.966 4.944 4.921 4.899
2.311 2.289 2.269 2.257 2.237 2.216 2.133 2.116 2.107 2.076 2.055 2.007 1.978 1.967 1.952 1.922 1.763 1.743 1.727 1.713 1.701
0.99 1.04 1.01 1.01 6.12 1.02 2.08 0.97 1.01 2.14 2.20 2.20 2.23
3ab
Ph
400 MHz, CDCl3
f1 (ppm)

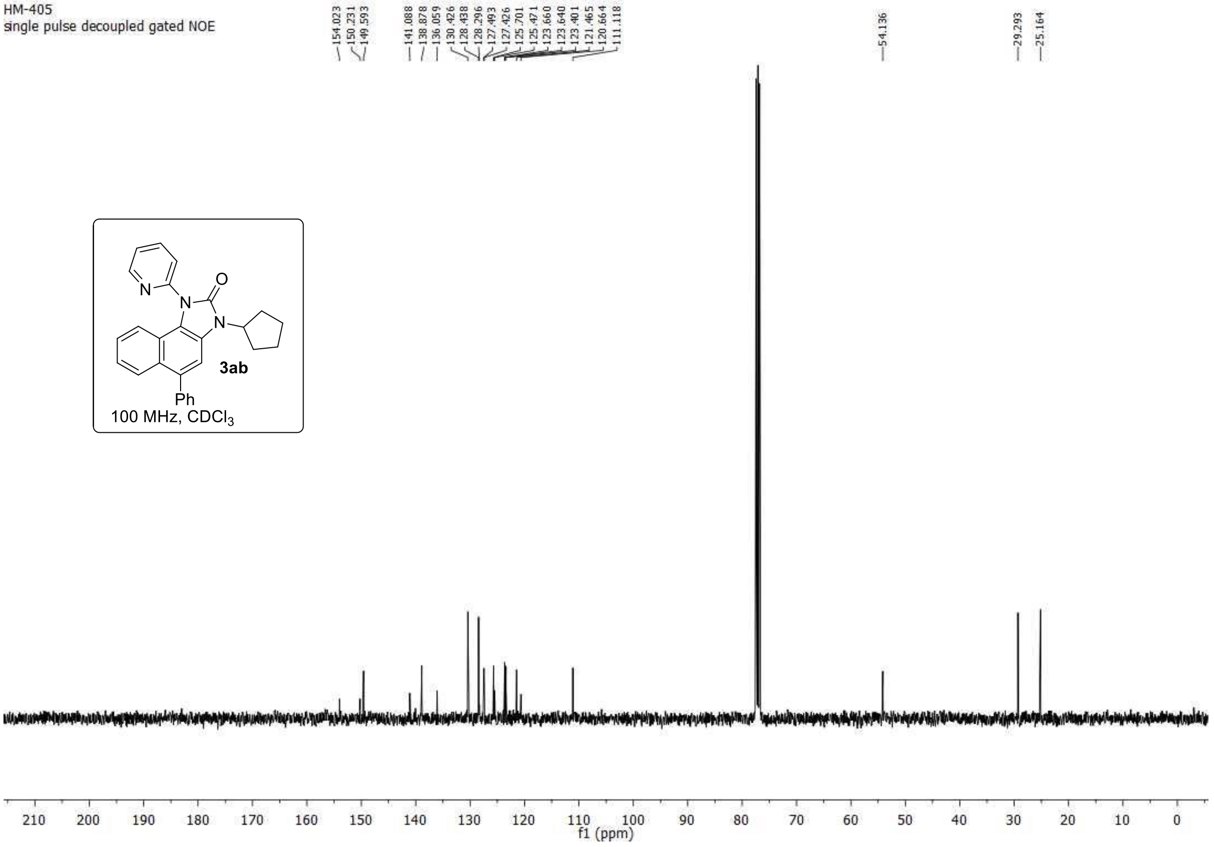
HM-405
single pulse decoupled gated NOE
154.023 150.231 149.593 141.088 138.878 136.059 130.426 128.438 128.296 127.493 127.426 125.701 125.471 123.660 123.640 123.401 121.465 120.664 111.118
54.136
29.293 25.164
3ab
Ph
100 MHz, CDCl3
f1 (ppm)

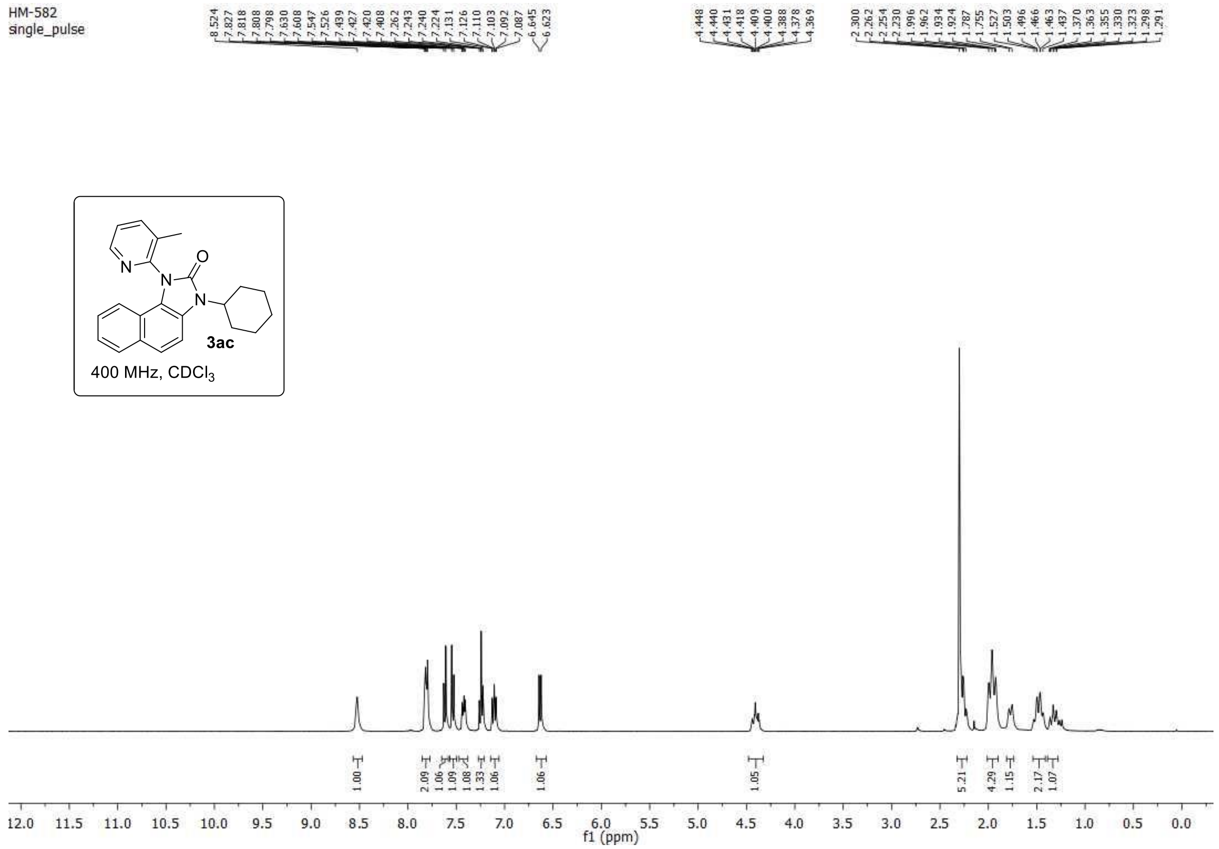
HM-582
single_pulse
8.524
7.827
7.818
7.808
7.798
7.630
7.608
7.547
7.526
7.439
7.427
7.420
7.408
7.262
7.243
7.240
7.224
7.131
7.126
7.110
7.103
7.092
7.087
6.645
6.623
4.448
4.440
4.431
4.418
4.409
4.400
4.388
4.378
4.369
2.300
2.262
2.254
2.230
1.996
1.962
1.934
1.924
1.787
1.755
1.527
1.503
1.496
1.466
1.463
1.437
1.370
1.363
1.355
1.330
1.323
1.298
1.291
N
O
N
N
3ac
400 MHz, CDCl3
1.00
2.09
1.06
1.09
1.08
1.33
1.06
1.06
1.05
5.21
4.29
1.15
2.17
1.07
12.0
11.5
11.0
10.5
10.0
9.5
9.0
8.5
8.0
7.5
7.0
6.5
6.0
5.5
5.0
4.5
4.0
3.5
3.0
2.5
2.0
1.5
1.0
0.5
0.0
f1 (ppm)

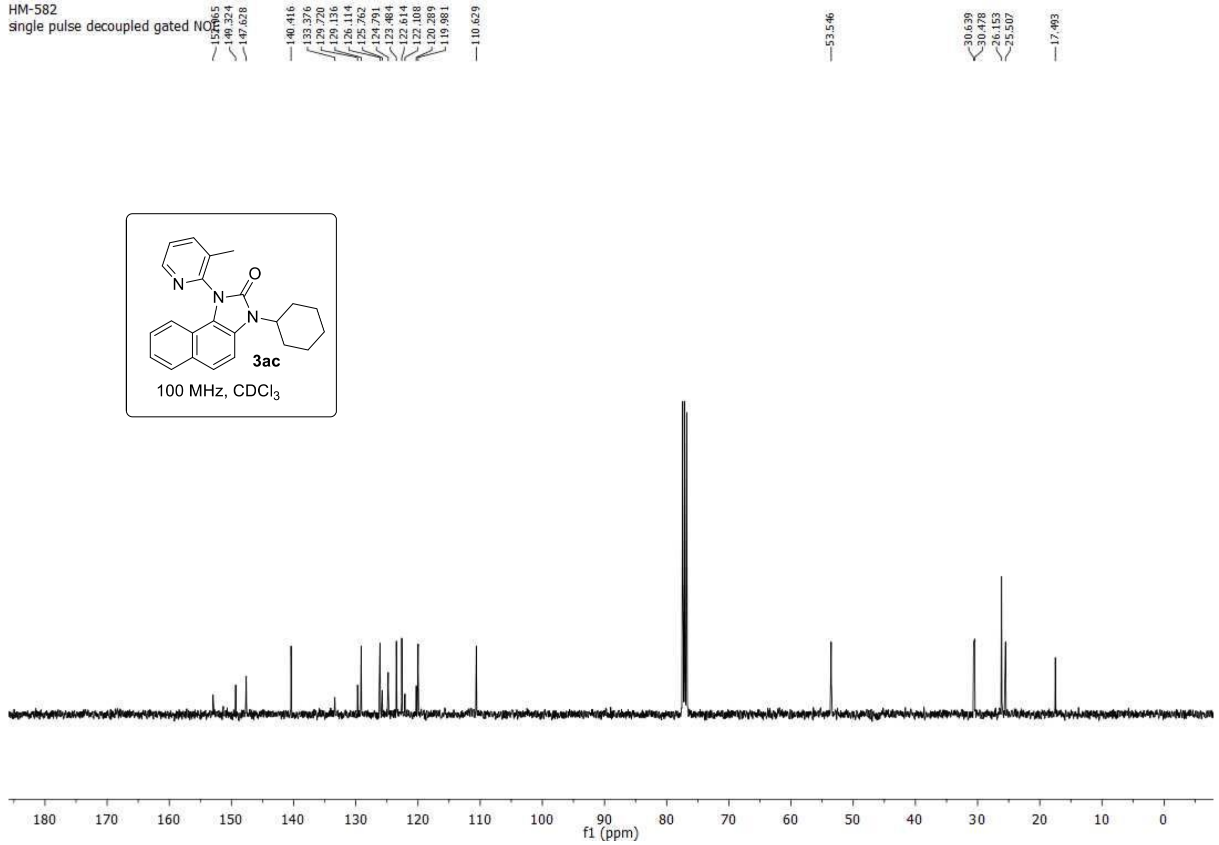
HM-582
single pulse decoupled gated NOE
149.324
147.628
140.416
133.376
129.720
129.136
126.114
125.762
124.791
123.484
122.614
122.108
120.289
119.981
110.629
53.546
30.639
30.478
26.153
25.507
17.493
N
O
N
N
3ac
100 MHz, CDCl3
180
170
160
150
140
130
120
110
100
90
80
70
60
50
40
30
20
10
0
f1 (ppm)

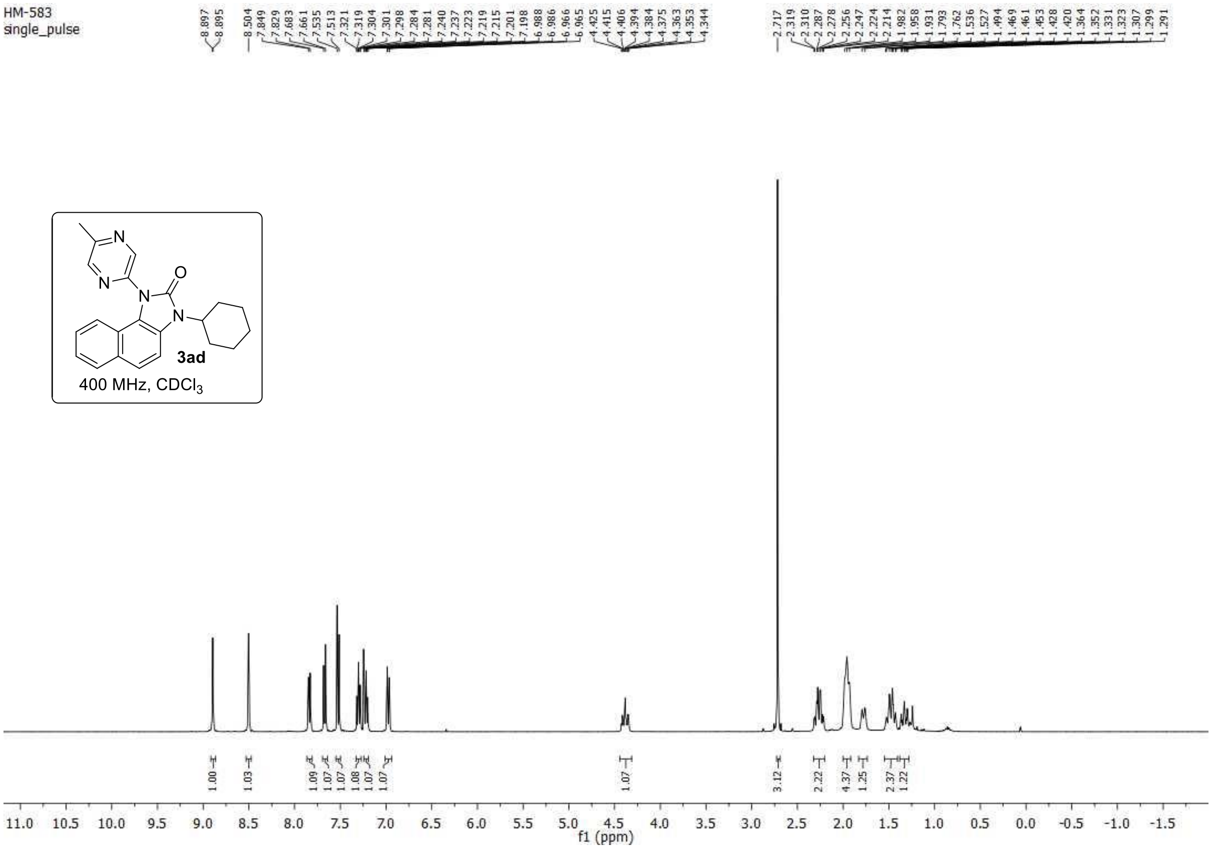
HM-583
single_pulse
8.897
8.895
8.504
7.849
7.829
7.683
7.661
7.535
7.513
7.321
7.319
7.304
7.301
7.298
7.284
7.281
7.240
7.237
7.223
7.219
7.215
7.201
7.198
6.988
6.986
6.966
6.965
4.425
4.415
4.406
4.394
4.384
4.375
4.363
4.353
4.344
2.717
2.319
2.310
2.287
2.278
2.256
2.247
2.224
2.214
1.982
1.958
1.931
1.793
1.762
1.536
1.527
1.494
1.469
1.461
1.453
1.428
1.420
1.364
1.352
1.331
1.323
1.307
1.299
1.291
3ad
400 MHz, CDCl3
1.00
1.03
1.09
1.07
1.07
1.08
1.07
1.07
1.07
3.12
2.22
4.37
1.25
2.37
1.22
f1 (ppm)

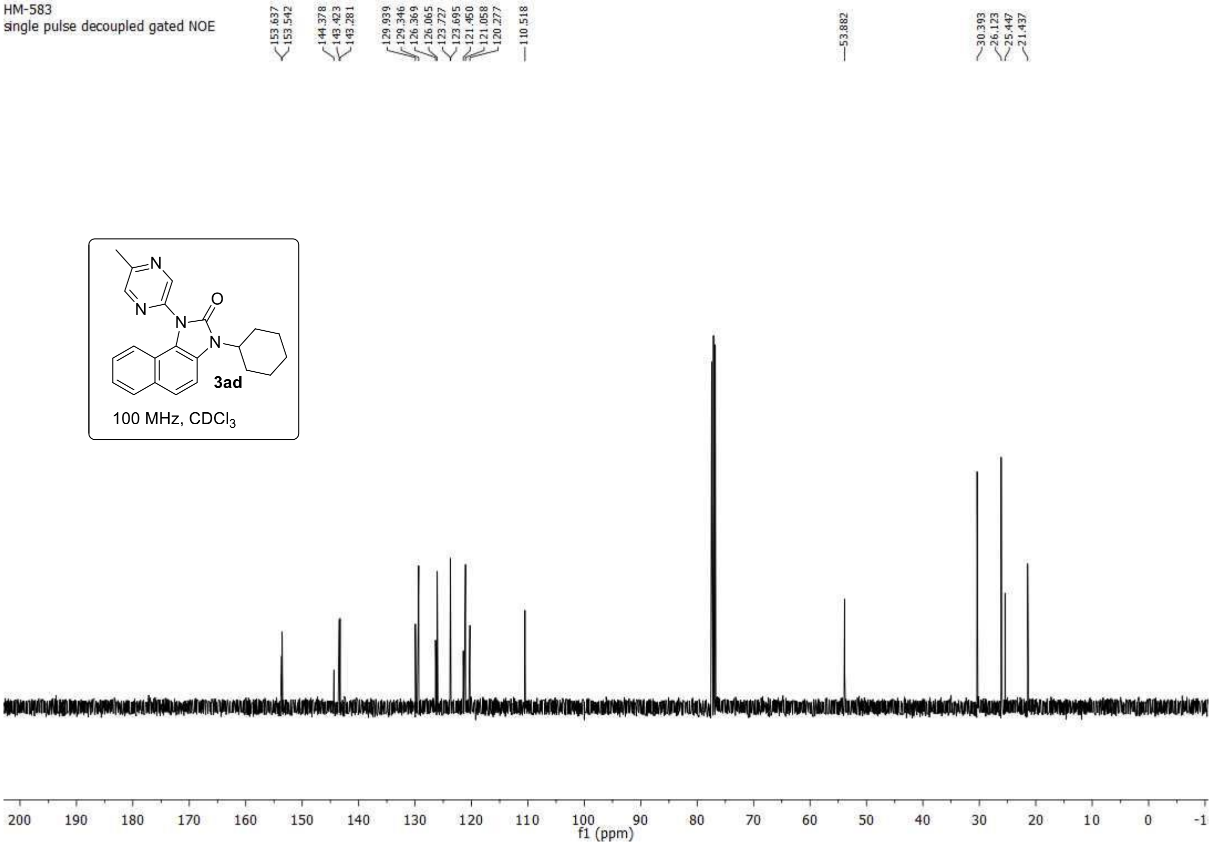
HM-583
single pulse decoupled gated NOE
153.637
153.542
144.378
143.423
143.281
129.939
129.346
126.369
126.065
123.727
123.695
121.450
121.058
120.277
110.518
53.882
30.393
26.123
25.447
21.437
3ad
100 MHz, CDCl3
f1 (ppm)

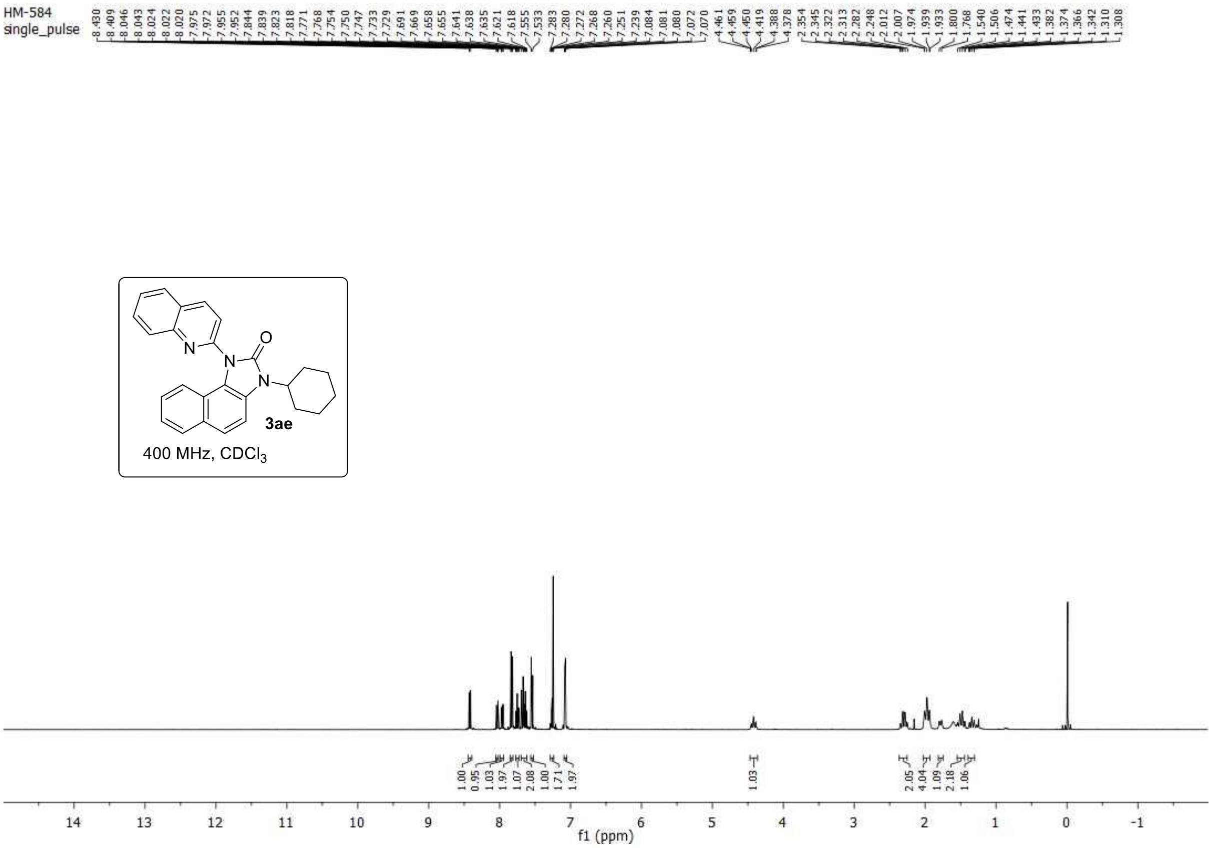
HM-584
single_pulse
8.430 8.409 8.046 8.043 8.024 8.022 8.020 7.975 7.972 7.955 7.952 7.844 7.839 7.823 7.818 7.771 7.768 7.754 7.750 7.747 7.733 7.729 7.691 7.669 7.658 7.655 7.641 7.638 7.635 7.621 7.618 7.555 7.533 7.283 7.280 7.272 7.268 7.260 7.251 7.239 7.084 7.081 7.080 7.072 7.070 4.461 4.459 4.450 4.419 4.388 4.378 2.354 2.345 2.322 2.313 2.282 2.248 2.012 2.007 1.974 1.939 1.933 1.800 1.768 1.540 1.506 1.474 1.441 1.433 1.382 1.374 1.366 1.342 1.310 1.308
O
N
N
N
3ae
400 MHz, CDCl$_3$
1.00 0.95 1.03 1.97 1.07 2.08 1.00 1.71 1.97
1.03
2.05 4.04 1.09 2.18 1.06
14 13 12 11 10 9 8 7 6 5 4 3 2 1 0 -1
f1 (ppm)

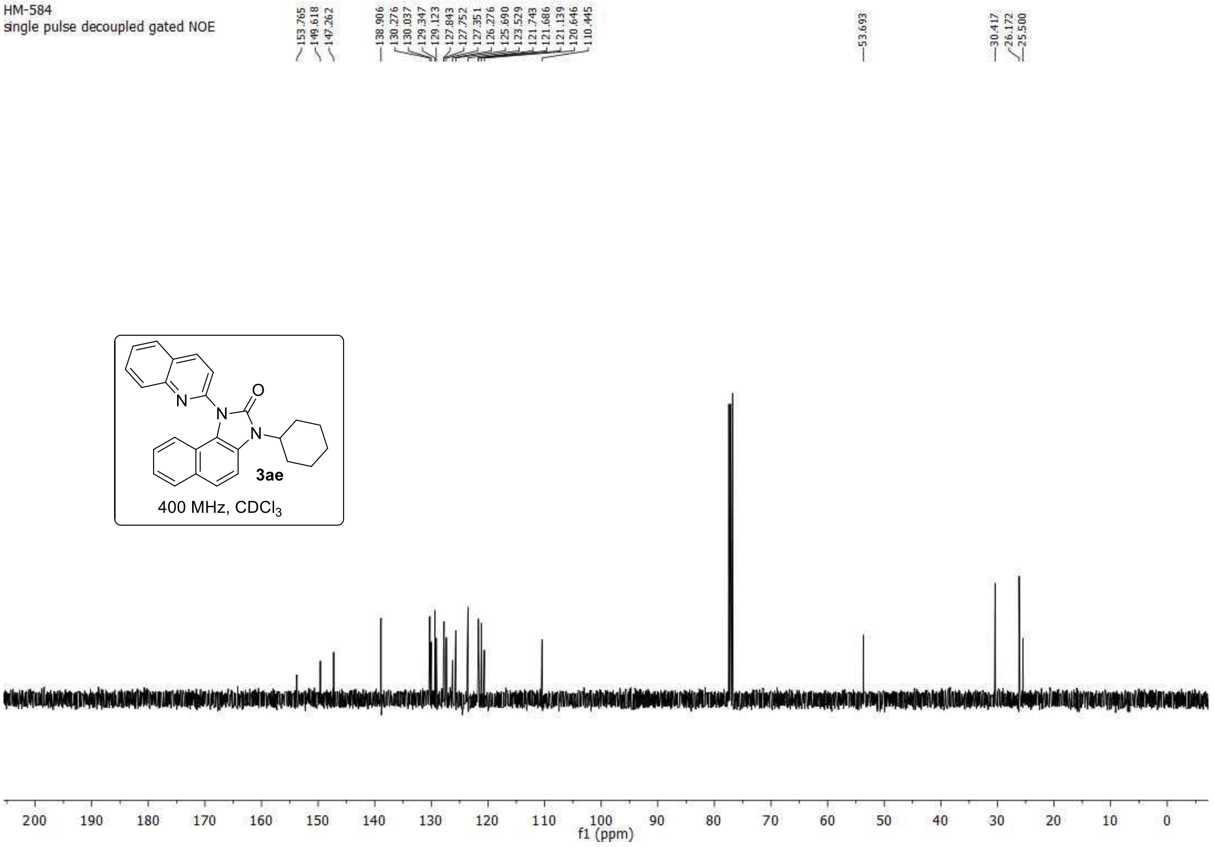
HM-584
single pulse decoupled gated NOE
153.765 149.618 147.262
138.906 130.276 130.037 129.347 129.123 127.843 127.752 127.351 126.276 125.690 123.529 121.743 121.686 121.139 120.646 110.445
53.693
30.417 26.172 25.500
O
N
N
N
3ae
400 MHz, CDCl$_3$
200 190 180 170 160 150 140 130 120 110 100 90 80 70 60 50 40 30 20 10 0
f1 (ppm)

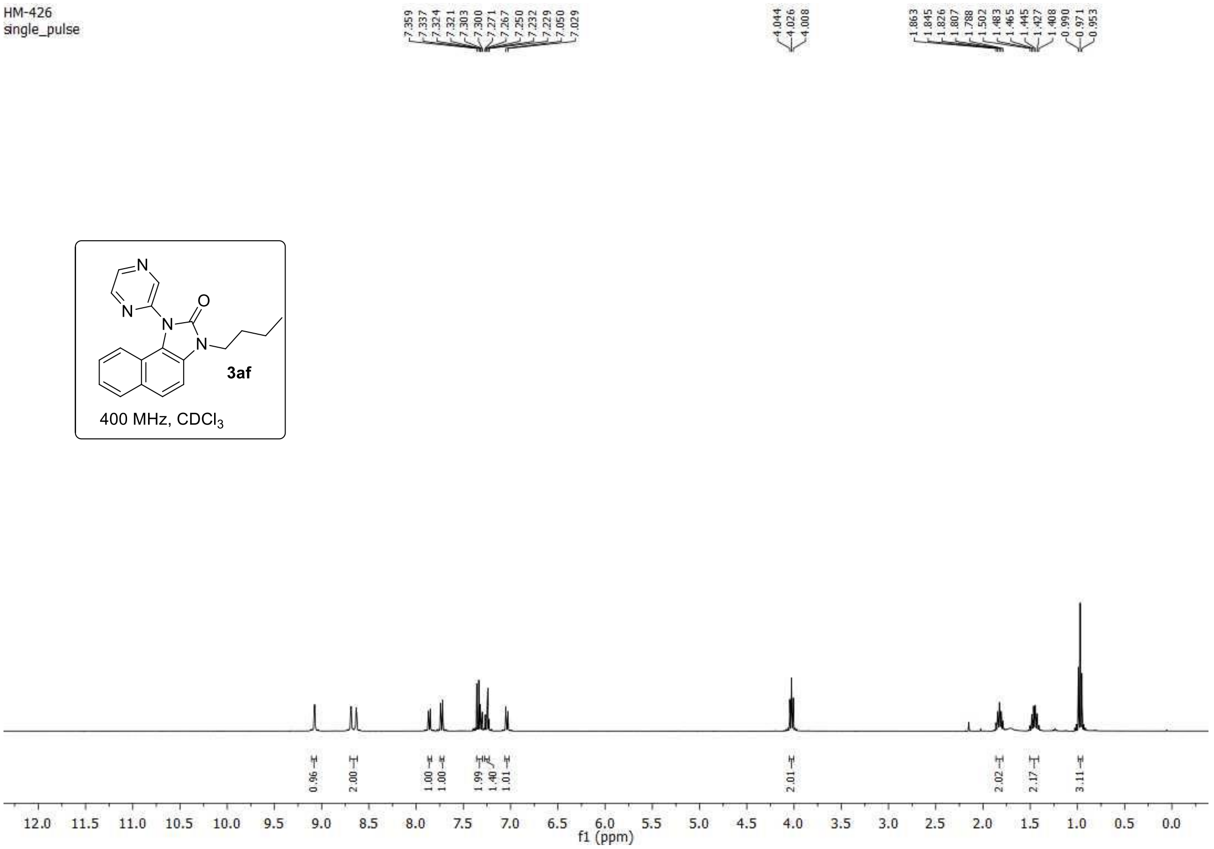
HM-426
single_pulse
7.359 7.337 7.324 7.321 7.303 7.300 7.271 7.267 7.250 7.232 7.229 7.050 7.029
4.044 4.026 4.008
1.863 1.845 1.826 1.807 1.788 1.502 1.483 1.465 1.445 1.427 1.408 0.990 0.971 0.953
3af
400 MHz, $CDCl_3$
0.96 2.00 1.00 1.00 1.99 1.40 1.01 2.01 2.02 2.17 3.11
f1 (ppm)

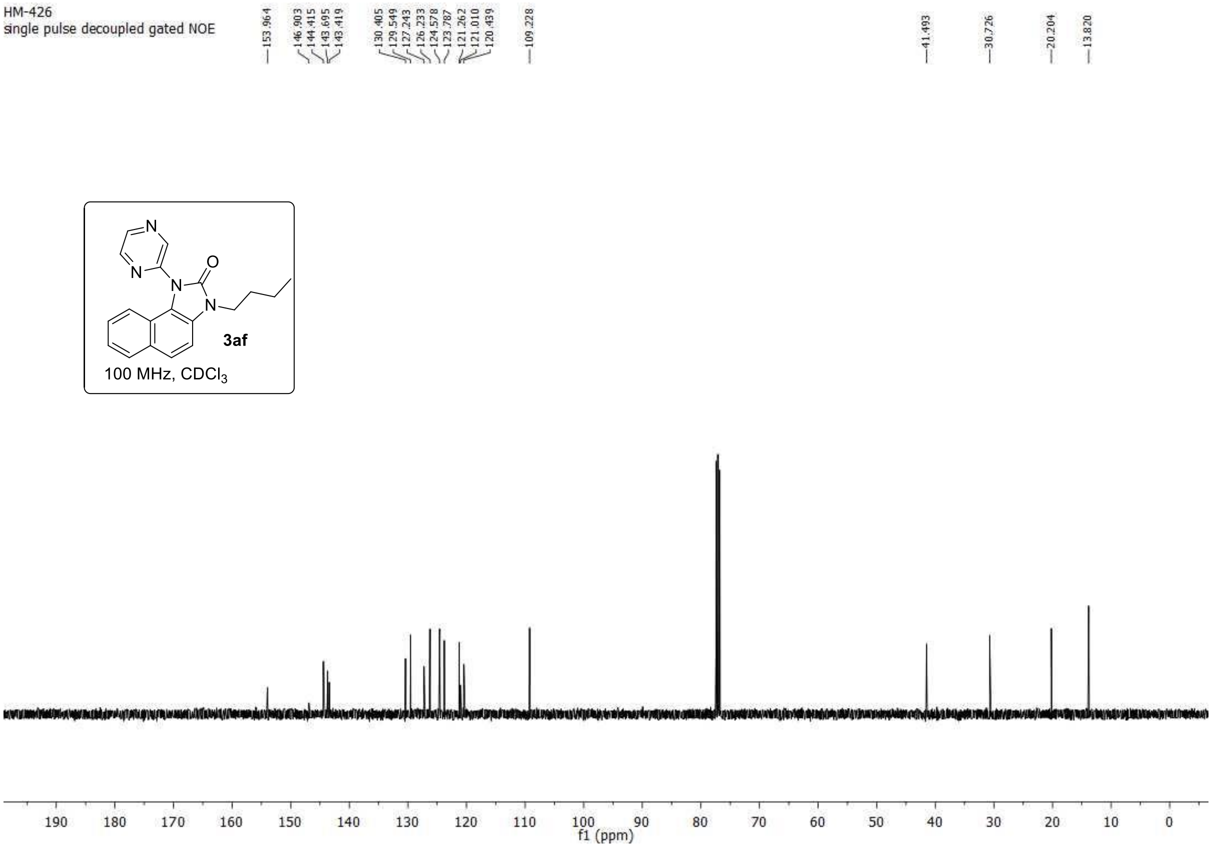
HM-426
single pulse decoupled gated NOE
153.964
146.903 144.415 143.695 143.419
130.405 129.549 127.243 126.233 124.578 123.787 121.262 121.010 120.439
109.228
41.493
30.726
20.204
13.820
3af
100 MHz, $CDCl_3$
f1 (ppm)

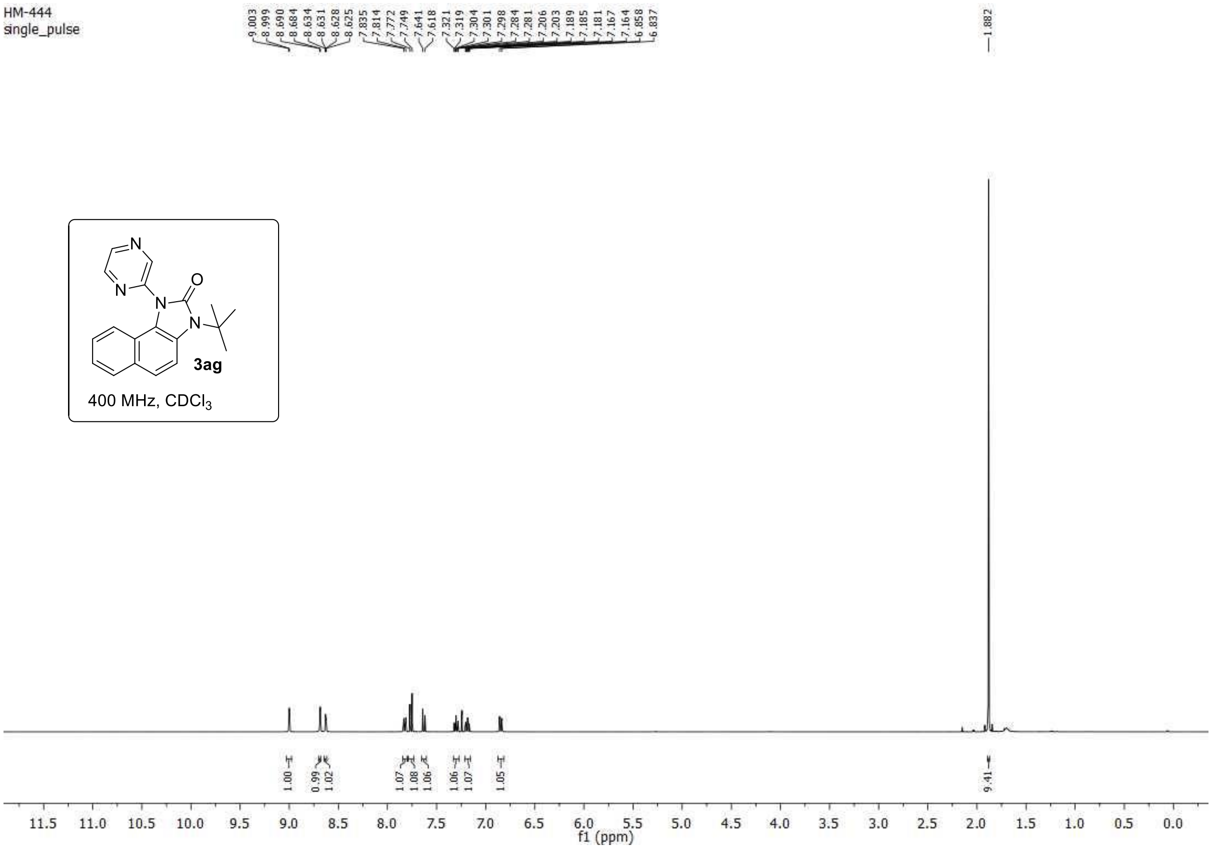
HM-444
single_pulse
9.003 8.999 8.690 8.684 8.634 8.631 8.628 8.625 7.835 7.814 7.772 7.749 7.641 7.618 7.321 7.319 7.304 7.301 7.298 7.284 7.281 7.206 7.203 7.189 7.185 7.181 7.167 7.164 6.858 6.837
1.882
N
N
N
O
N
3ag
400 MHz, CDCl$_3$
1.00 0.99 1.02 1.07 1.08 1.06 1.06 1.07 1.05 9.41
11.5 11.0 10.5 10.0 9.5 9.0 8.5 8.0 7.5 7.0 6.5 6.0 5.5 5.0 4.5 4.0 3.5 3.0 2.5 2.0 1.5 1.0 0.5 0.0
f1 (ppm)

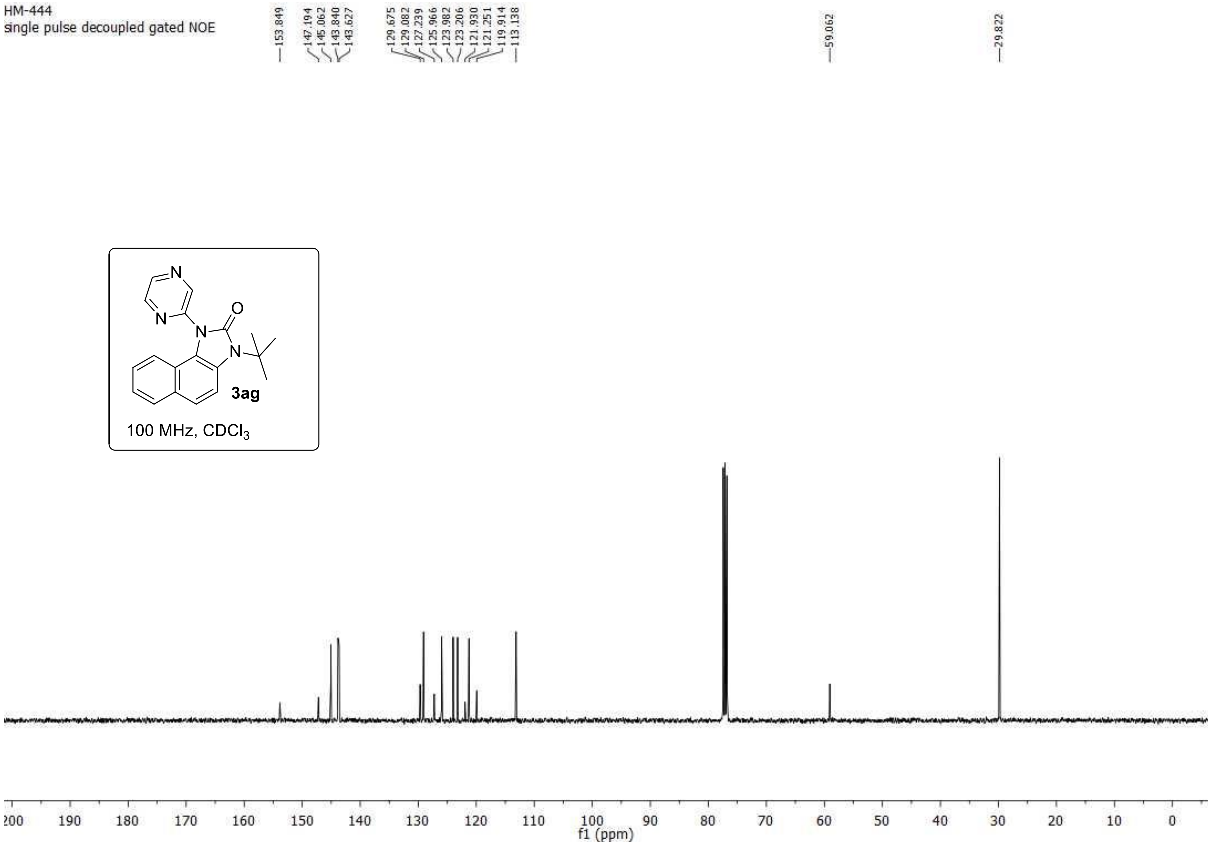
HM-444
single pulse decoupled gated NOE
153.849 147.194 145.062 143.840 143.627 129.675 129.082 127.239 125.966 123.982 123.206 121.930 121.251 119.914 113.138
59.062
29.822
N
N
N
O
N
3ag
100 MHz, CDCl$_3$
200 190 180 170 160 150 140 130 120 110 100 90 80 70 60 50 40 30 20 10 0
f1 (ppm)

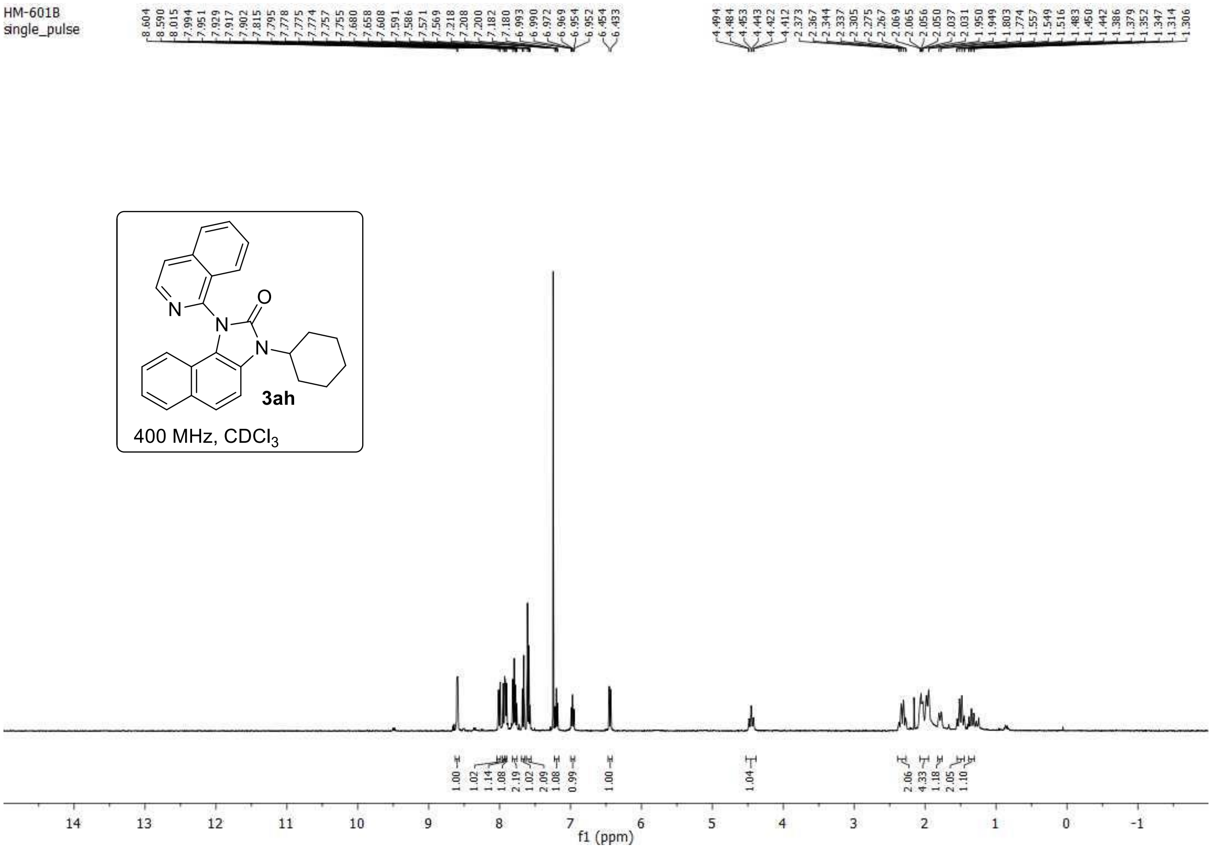
HM-601B
single_pulse
8.604 8.590 8.015 7.994 7.951 7.929 7.917 7.902 7.815 7.795 7.778 7.775 7.774 7.757 7.755 7.680 7.658 7.608 7.591 7.586 7.571 7.569 7.218 7.208 7.200 7.182 7.180 6.993 6.990 6.972 6.969 6.954 6.952 6.454 6.433
4.494 4.484 4.453 4.443 4.422 4.412 2.373 2.367 2.344 2.337 2.305 2.275 2.267 2.069 2.065 2.056 2.050 2.037 2.031 1.950 1.949 1.803 1.774 1.557 1.549 1.516 1.483 1.450 1.442 1.386 1.379 1.352 1.347 1.314 1.306
N
N
N
O
3ah
400 MHz, CDCl3
1.00 1.02 1.14 1.08 2.19 1.02 2.09 1.08 0.99 1.00 1.04 2.06 4.33 1.18 2.05 1.10
14 13 12 11 10 9 8 7 6 5 4 3 2 1 0 -1
f1 (ppm)

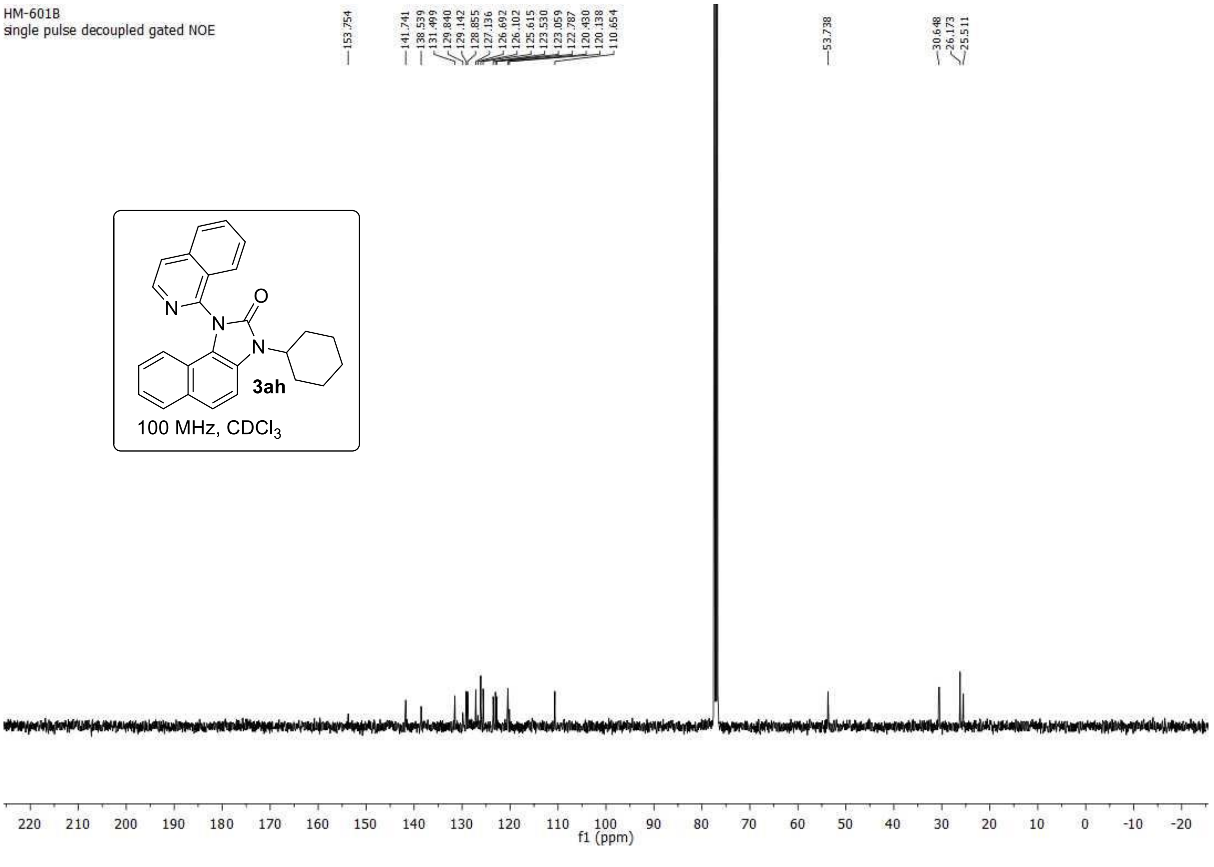
HM-601B
single pulse decoupled gated NOE
153.754
141.741 138.539 131.499 129.840 129.142 128.855 127.136 126.692 126.102 125.615 123.530 123.059 122.787 120.430 120.138 110.654
53.738
30.648 26.173 25.511
N
N
N
O
3ah
100 MHz, CDCl3
220 210 200 190 180 170 160 150 140 130 120 110 100 90 80 70 60 50 40 30 20 10 0 -10 -20
f1 (ppm)

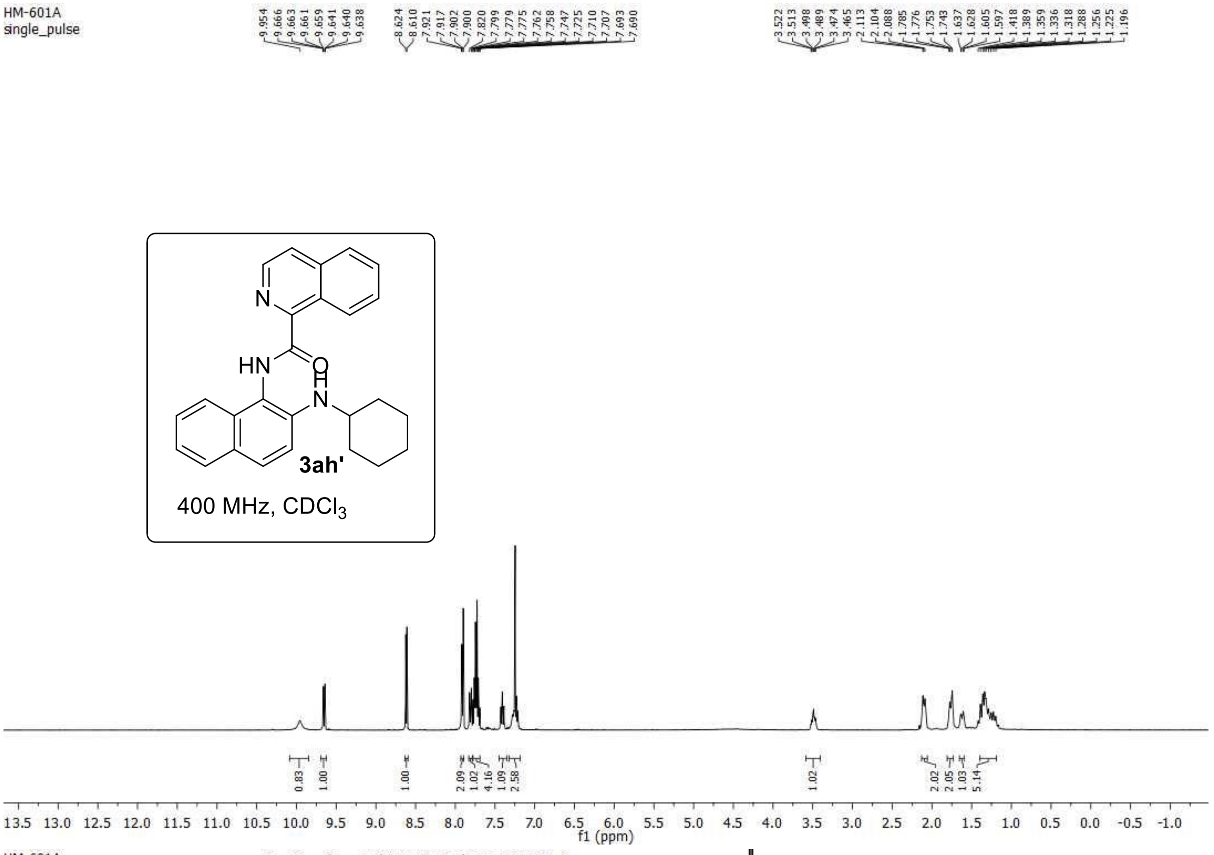
HM-601A
single_pulse
9.954 9.666 9.663 9.661 9.659 9.641 9.640 9.638
8.624 8.610 7.921 7.917 7.902 7.900 7.820 7.799 7.779 7.775 7.762 7.758 7.747 7.725 7.710 7.707 7.693 7.690
3.522 3.513 3.498 3.489 3.474 3.465 2.113 2.104 2.088 1.785 1.776 1.753 1.743 1.637 1.628 1.605 1.597 1.418 1.389 1.359 1.336 1.318 1.288 1.256 1.225 1.196
N
HN
O
H
N
3ah'
400 MHz, CDCl3
0.83 1.00 1.00 2.09 1.02 4.16 1.09 2.58 1.02 2.02 2.05 1.03 5.14
13.5 13.0 12.5 12.0 11.5 11.0 10.5 10.0 9.5 9.0 8.5 8.0 7.5 7.0 6.5 6.0 5.5 5.0 4.5 4.0 3.5 3.0 2.5 2.0 1.5 1.0 0.5 0.0 -0.5 -1.0
f1 (ppm)

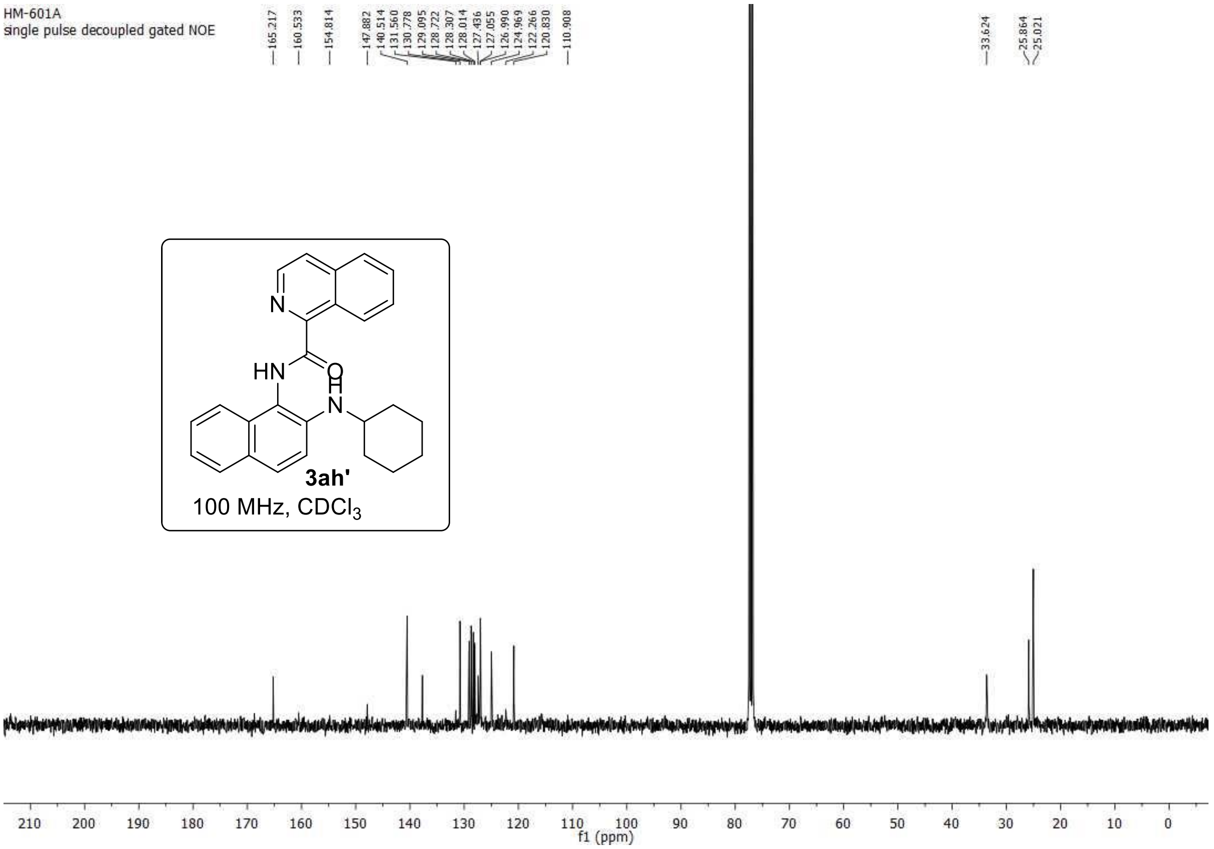
HM-601A
single pulse decoupled gated NOE
165.217 160.533 154.814 147.882 140.514 131.560 130.778 129.095 128.722 128.307 128.014 127.436 127.055 126.990 124.969 122.266 120.830 110.908
33.624 25.864 25.021
N
HN
O
H
N
3ah'
100 MHz, CDCl3
210 200 190 180 170 160 150 140 130 120 110 100 90 80 70 60 50 40 30 20 10 0
f1 (ppm)

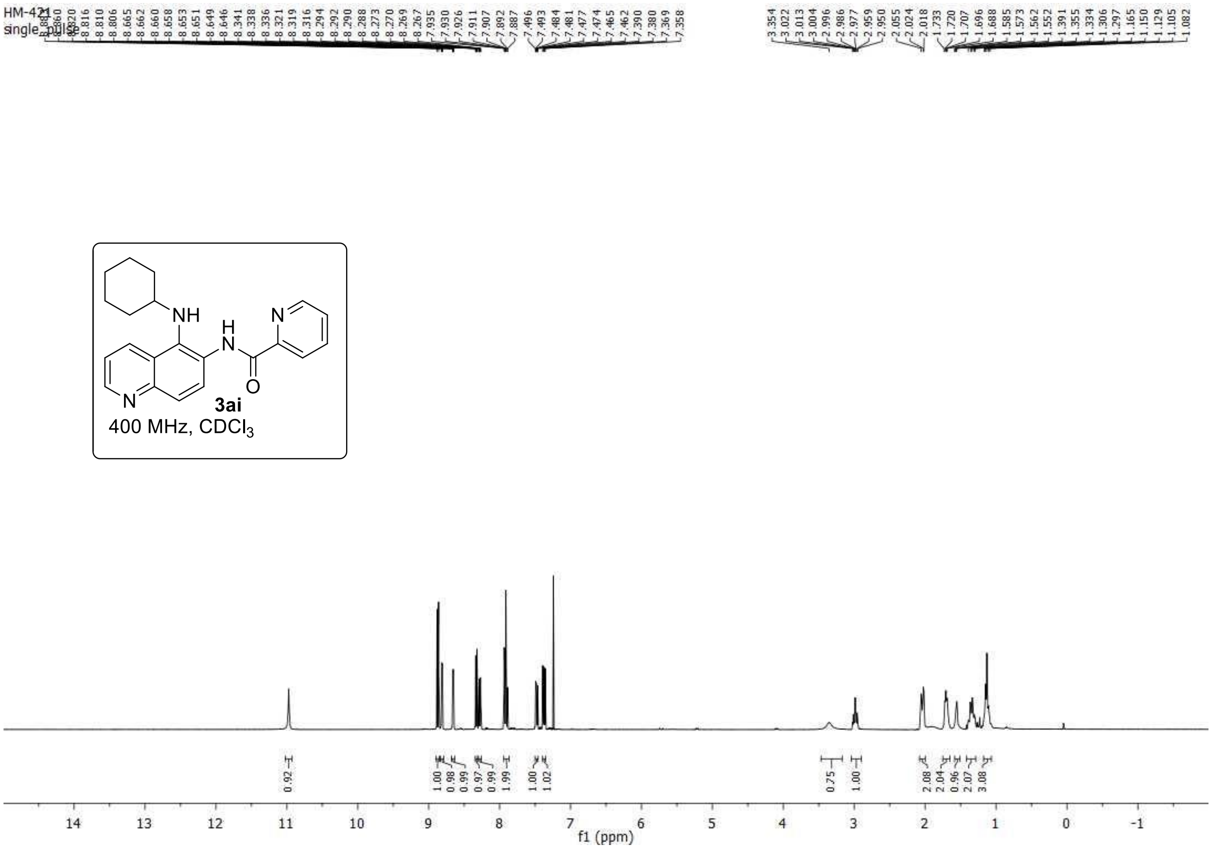
HM-421
single_pulse
8.864
8.860
8.820
8.816
8.810
8.806
8.665
8.662
8.660
8.658
8.653
8.651
8.649
8.646
8.341
8.338
8.336
8.321
8.319
8.316
8.294
8.292
8.290
8.288
8.273
8.270
8.269
8.267
7.935
7.930
7.926
7.911
7.907
7.892
7.887
7.496
7.493
7.484
7.481
7.477
7.474
7.465
7.462
7.390
7.380
7.369
7.358
3.354
3.022
3.013
3.004
2.996
2.986
2.977
2.959
2.950
2.055
2.024
2.018
1.733
1.720
1.707
1.696
1.688
1.585
1.573
1.562
1.552
1.391
1.355
1.334
1.306
1.297
1.165
1.150
1.129
1.105
1.082
NH
H
N
N
O
N
3ai
400 MHz, CDCl3
0.92
1.00
0.98
0.99
0.97
0.99
1.99
1.00
1.02
0.75
1.00
2.08
2.04
0.96
2.07
3.08
14
13
12
11
10
9
8
7
6
5
4
3
2
1
0
-1
f1 (ppm)

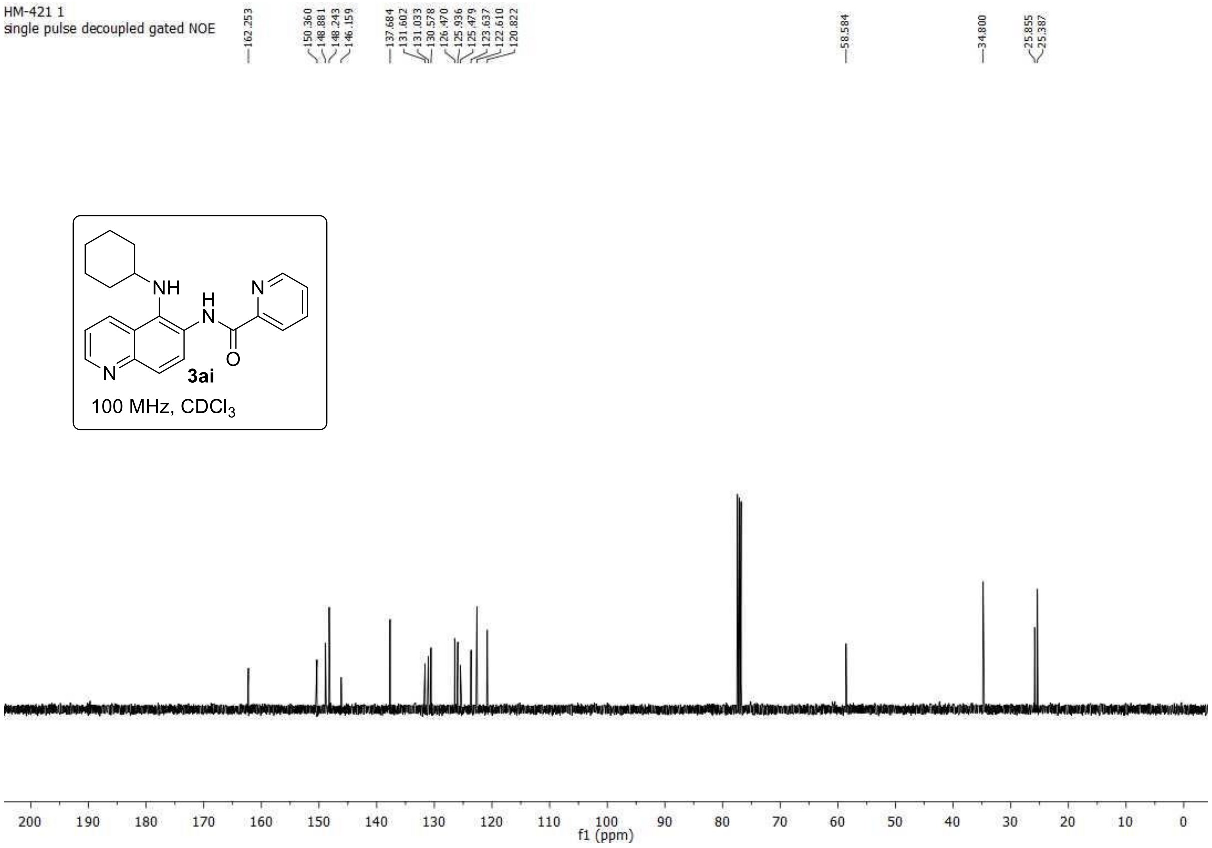
HM-421 1
single pulse decoupled gated NOE
162.253
150.360
148.881
148.243
146.159
137.684
131.602
131.033
130.578
126.470
125.936
125.479
123.637
122.610
120.822
58.584
34.800
25.855
25.387
NH
H
N
N
O
N
3ai
100 MHz, CDCl3
200
190
180
170
160
150
140
130
120
110
100
90
80
70
60
50
40
30
20
10
0
f1 (ppm)

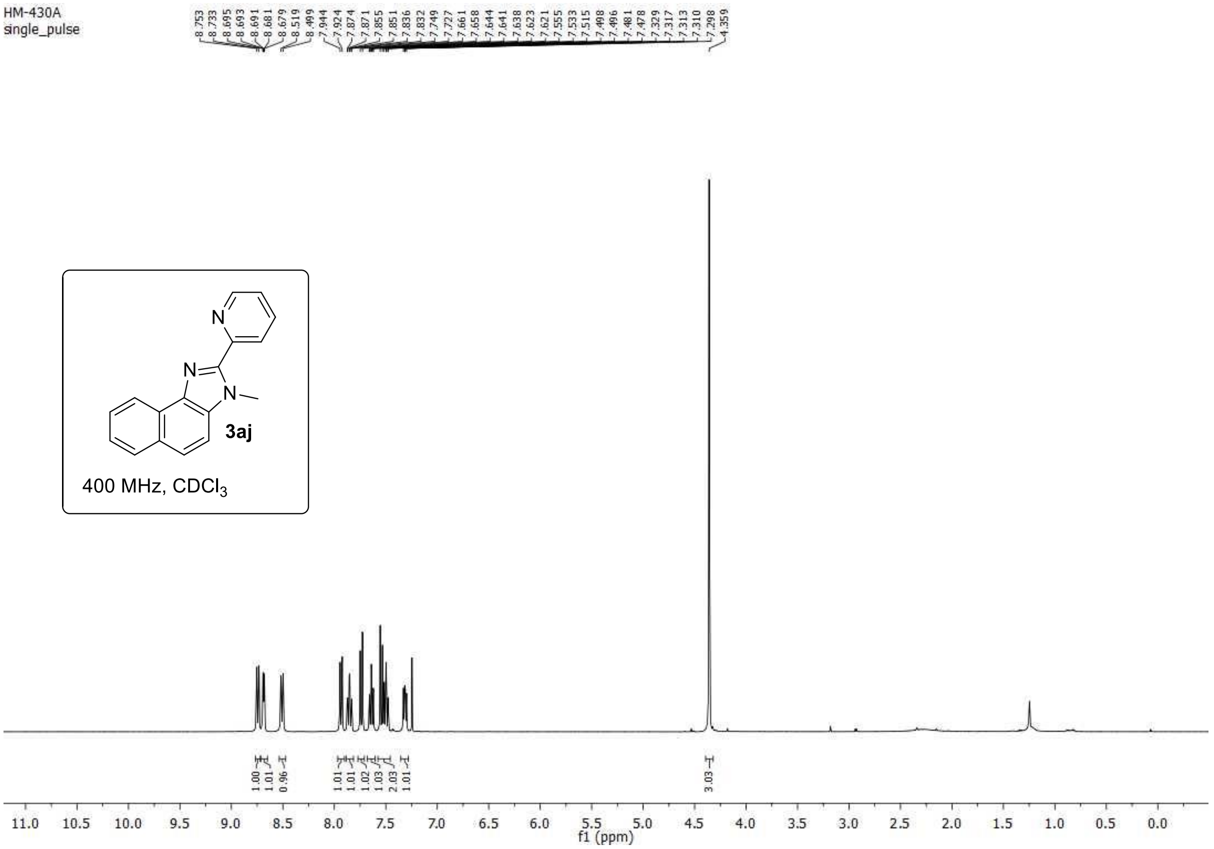
HM-430A
single_pulse
3aj
400 MHz, CDCl3
f1 (ppm)

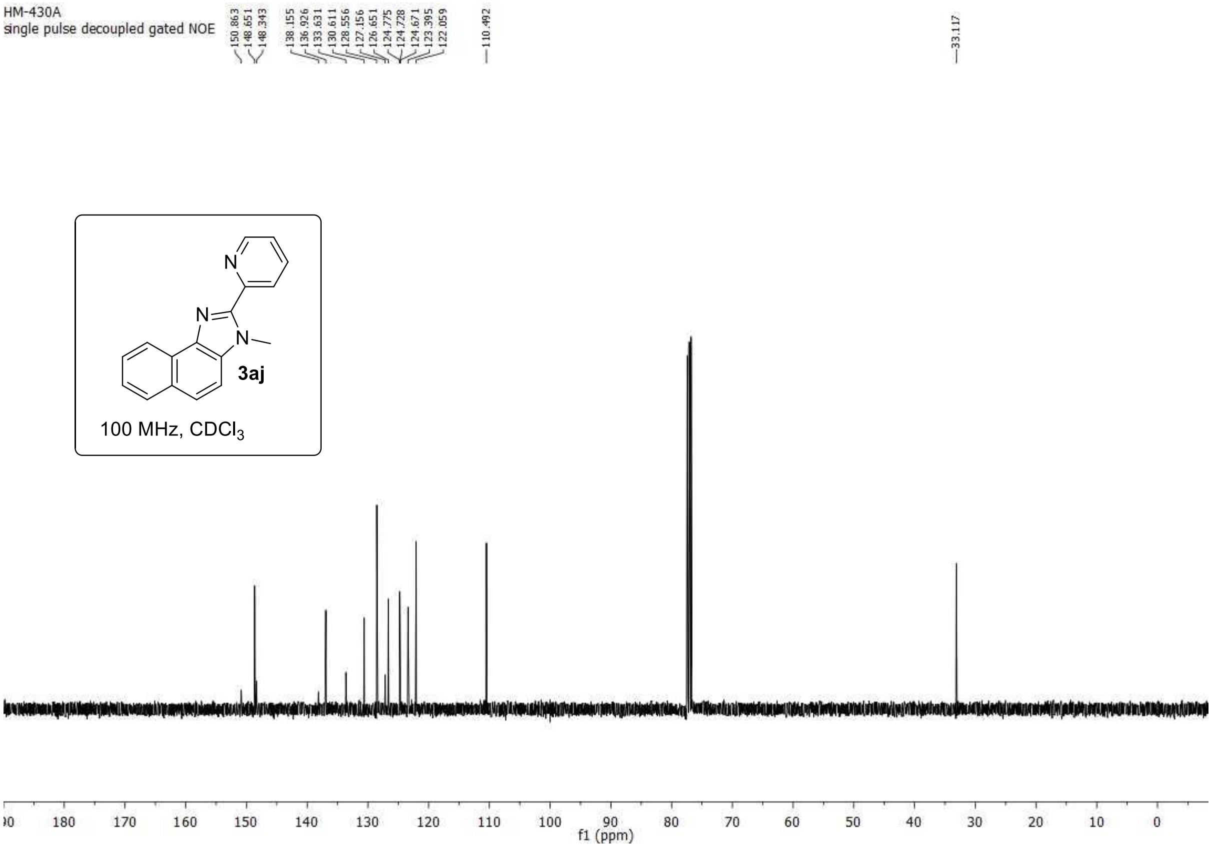
HM-430A
single pulse decoupled gated NOE
3aj
100 MHz, CDCl3
f1 (ppm)

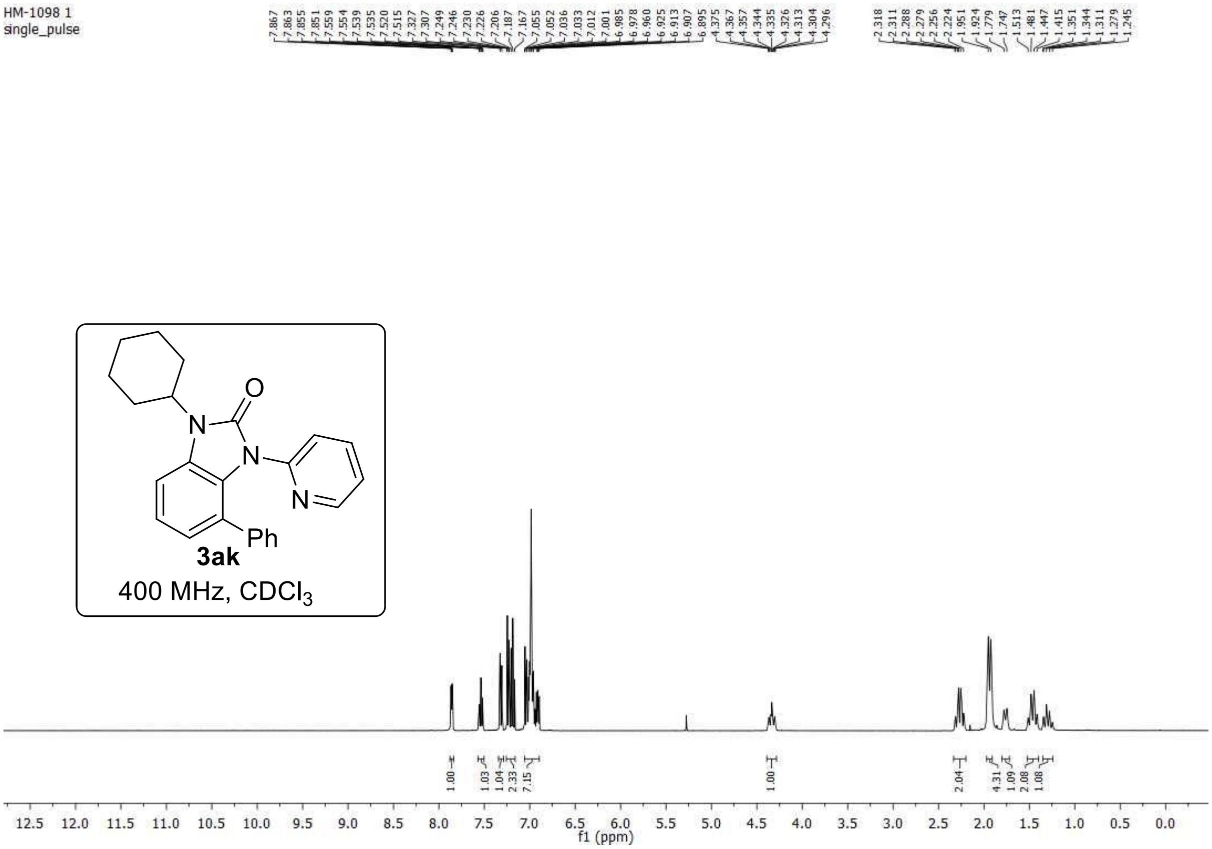

HM-1098 1
single_pulse
7.867 7.863 7.855 7.851 7.559 7.554 7.539 7.535 7.520 7.515 7.327 7.307 7.249 7.246 7.230 7.226 7.206 7.187 7.167 7.055 7.052 7.036 7.033 7.012 7.001 6.985 6.978 6.960 6.925 6.913 6.907 6.895 4.375 4.367 4.357 4.344 4.335 4.326 4.313 4.304 4.296 2.318 2.311 2.288 2.279 2.256 2.224 1.951 1.924 1.779 1.747 1.513 1.481 1.447 1.415 1.351 1.344 1.311 1.279 1.245
O
N
N
N
Ph
3ak
400 MHz, CDCl3
1.00 1.03 1.04 2.33 7.15 1.00 2.04 4.31 1.09 2.08 1.08
12.5 12.0 11.5 11.0 10.5 10.0 9.5 9.0 8.5 8.0 7.5 7.0 6.5 6.0 5.5 5.0 4.5 4.0 3.5 3.0 2.5 2.0 1.5 1.0 0.5 0.0
f1 (ppm)

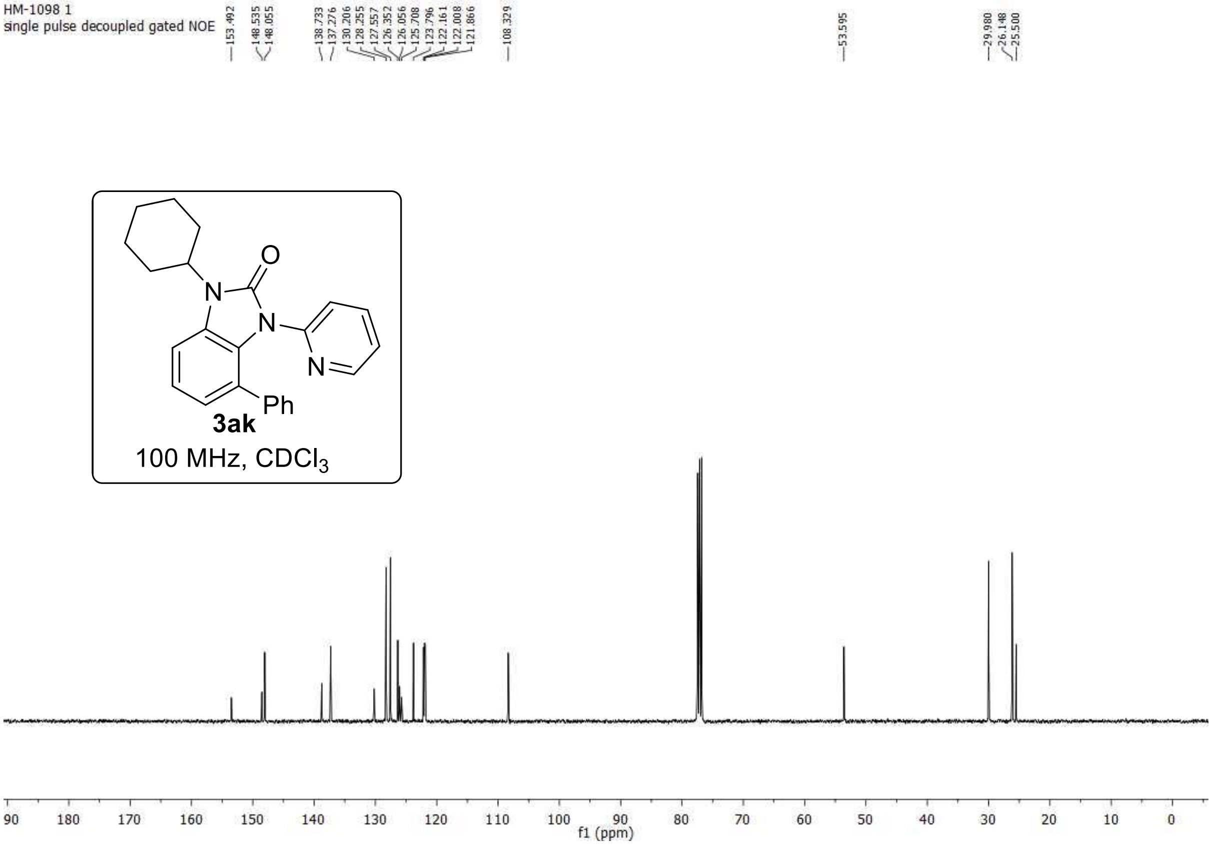

HM-1098 1
single pulse decoupled gated NOE
153.492 148.535 148.055 138.733 137.276 130.206 128.255 127.557 126.352 126.056 125.708 123.796 122.161 122.008 121.866 108.329 53.595 29.980 26.148 25.500
O
N
N
N
Ph
3ak
100 MHz, CDCl3
190 180 170 160 150 140 130 120 110 100 90 80 70 60 50 40 30 20 10 0
f1 (ppm)

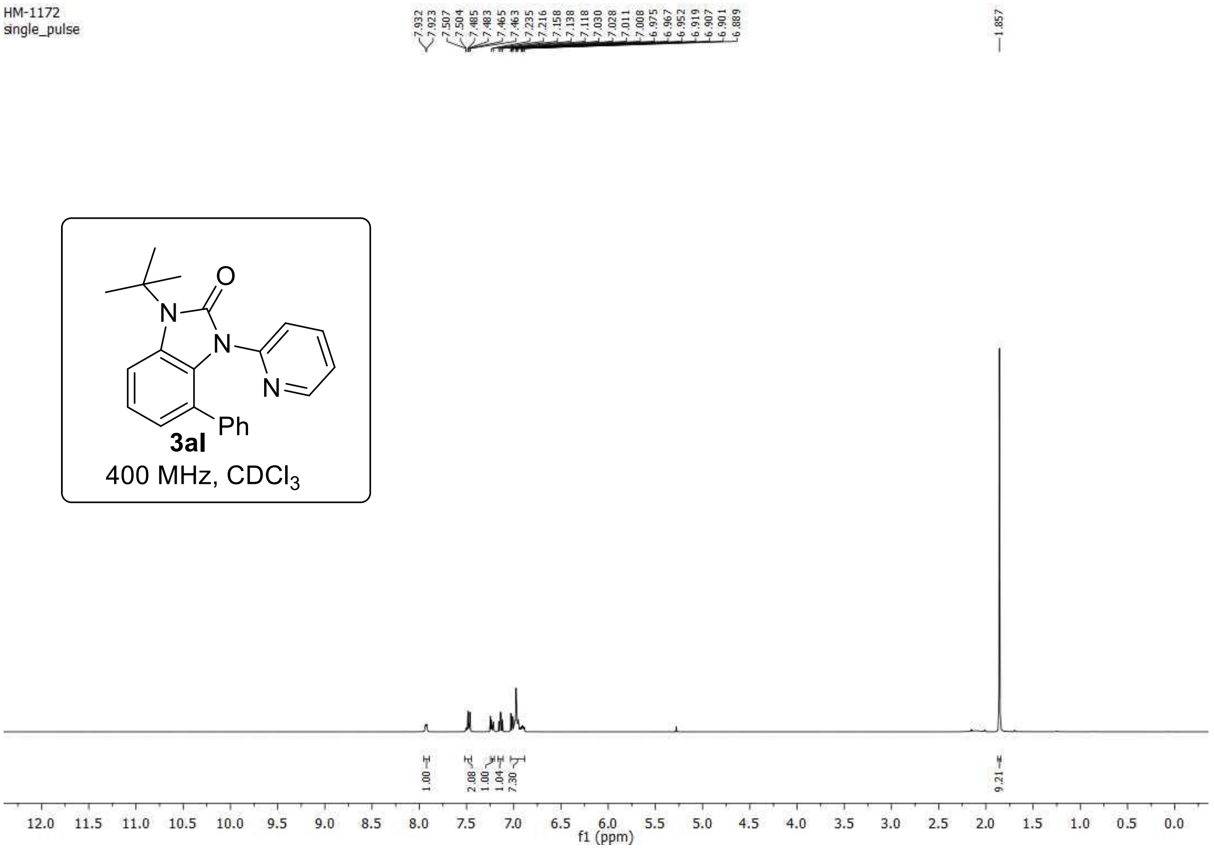
HM-1172
single_pulse
7.932
7.923
7.507
7.504
7.485
7.483
7.465
7.463
7.235
7.216
7.158
7.138
7.118
7.030
7.028
7.011
7.008
6.975
6.967
6.952
6.919
6.907
6.901
6.889
1.857
3al
400 MHz, CDCl3
1.00
2.08
1.00
1.04
7.30
9.21
12.0
11.5
11.0
10.5
10.0
9.5
9.0
8.5
8.0
7.5
7.0
6.5
6.0
5.5
5.0
4.5
4.0
3.5
3.0
2.5
2.0
1.5
1.0
0.5
0.0
f1 (ppm)

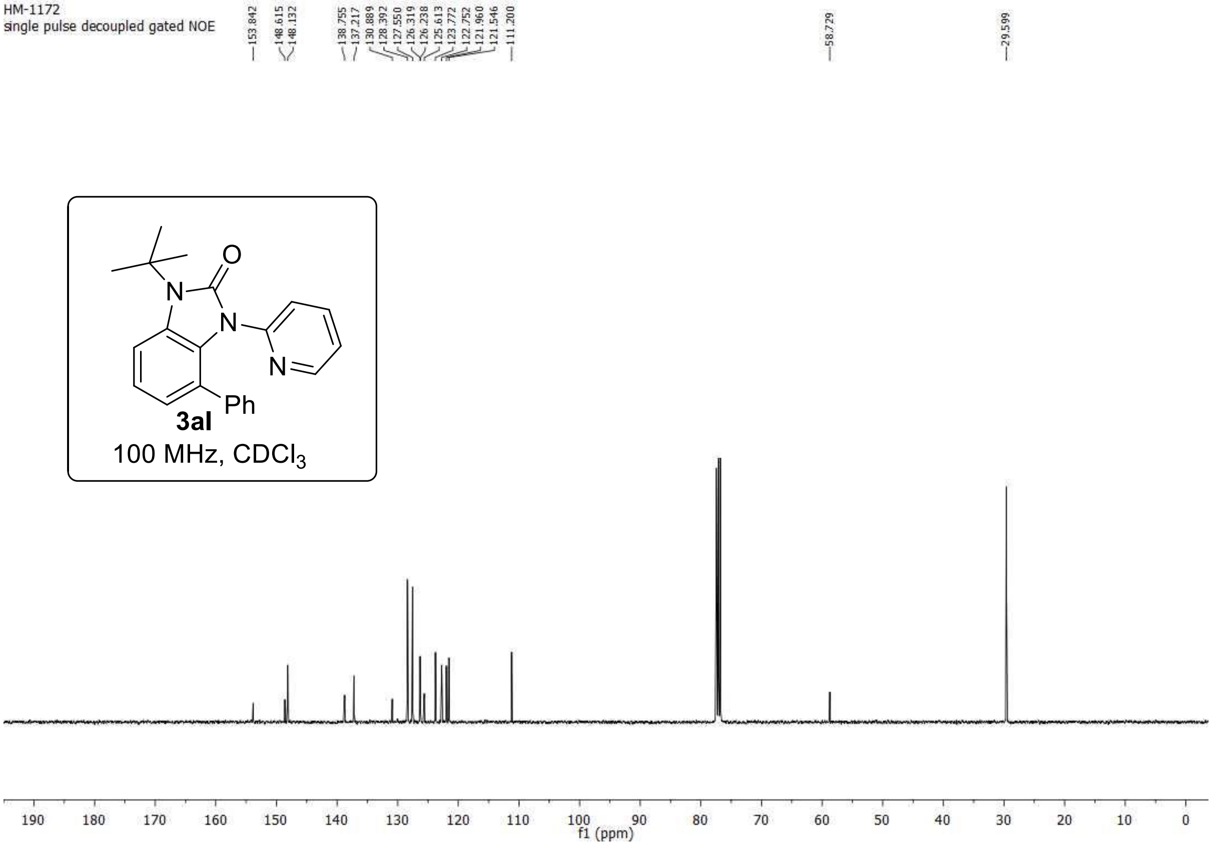
HM-1172
single pulse decoupled gated NOE
153.842
148.615
148.132
138.755
137.217
130.889
128.392
127.550
126.319
126.238
125.613
123.772
122.752
121.960
121.546
111.200
58.729
29.599
3al
100 MHz, CDCl3
190
180
170
160
150
140
130
120
110
100
90
80
70
60
50
40
30
20
10
0
f1 (ppm)

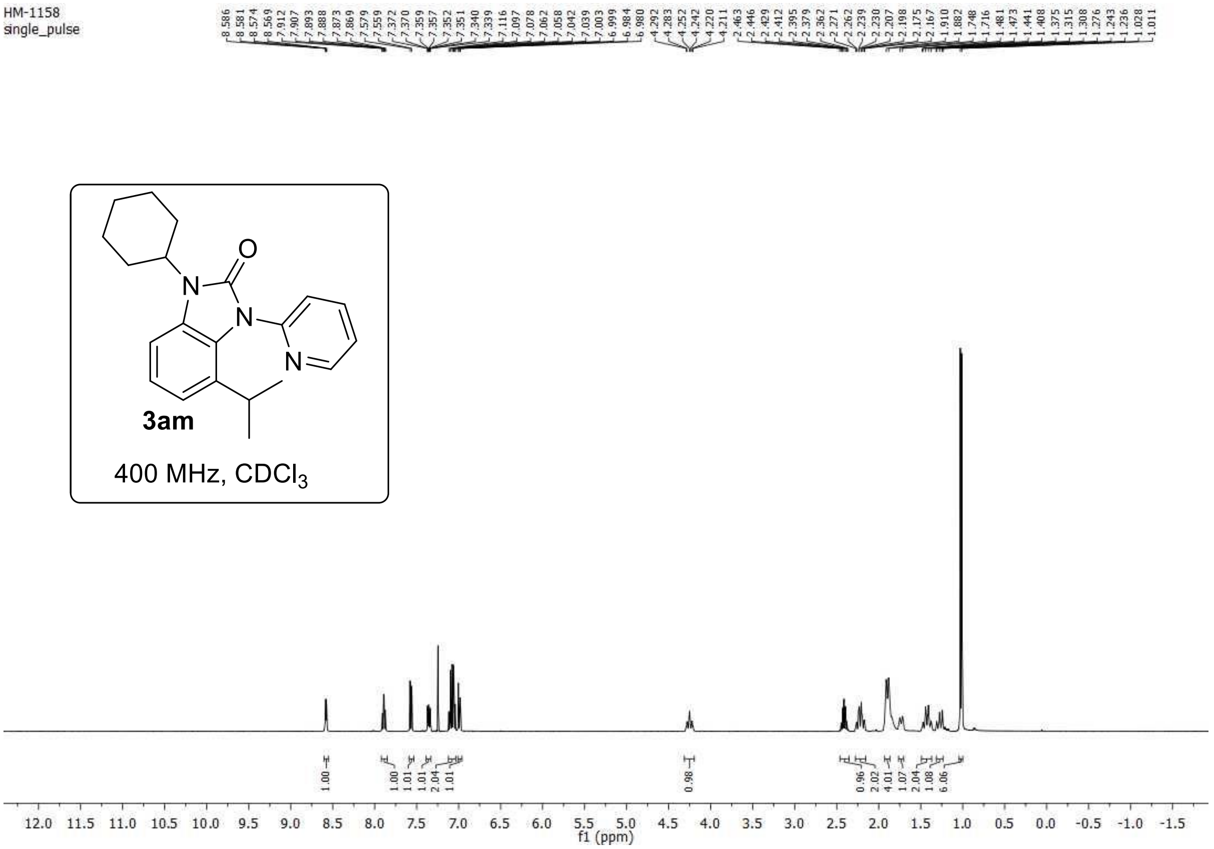
HM-1158
single_pulse
8.586 8.581 8.574 8.569 7.912 7.907 7.893 7.888 7.873 7.869 7.579 7.559 7.372 7.370 7.359 7.357 7.352 7.351 7.340 7.339 7.116 7.097 7.078 7.062 7.058 7.042 7.039 7.003 6.999 6.984 6.980 4.292 4.283 4.252 4.242 4.220 4.211 2.463 2.446 2.429 2.412 2.395 2.379 2.362 2.271 2.262 2.239 2.230 2.207 2.198 2.175 2.167 1.910 1.882 1.748 1.716 1.481 1.473 1.441 1.408 1.375 1.315 1.308 1.276 1.243 1.236 1.028 1.011
3am
400 MHz, CDCl$_3$
1.00 1.00 1.01 1.01 2.04 1.01 0.98 0.96 2.02 4.01 1.07 2.04 1.08 6.06
f1 (ppm)

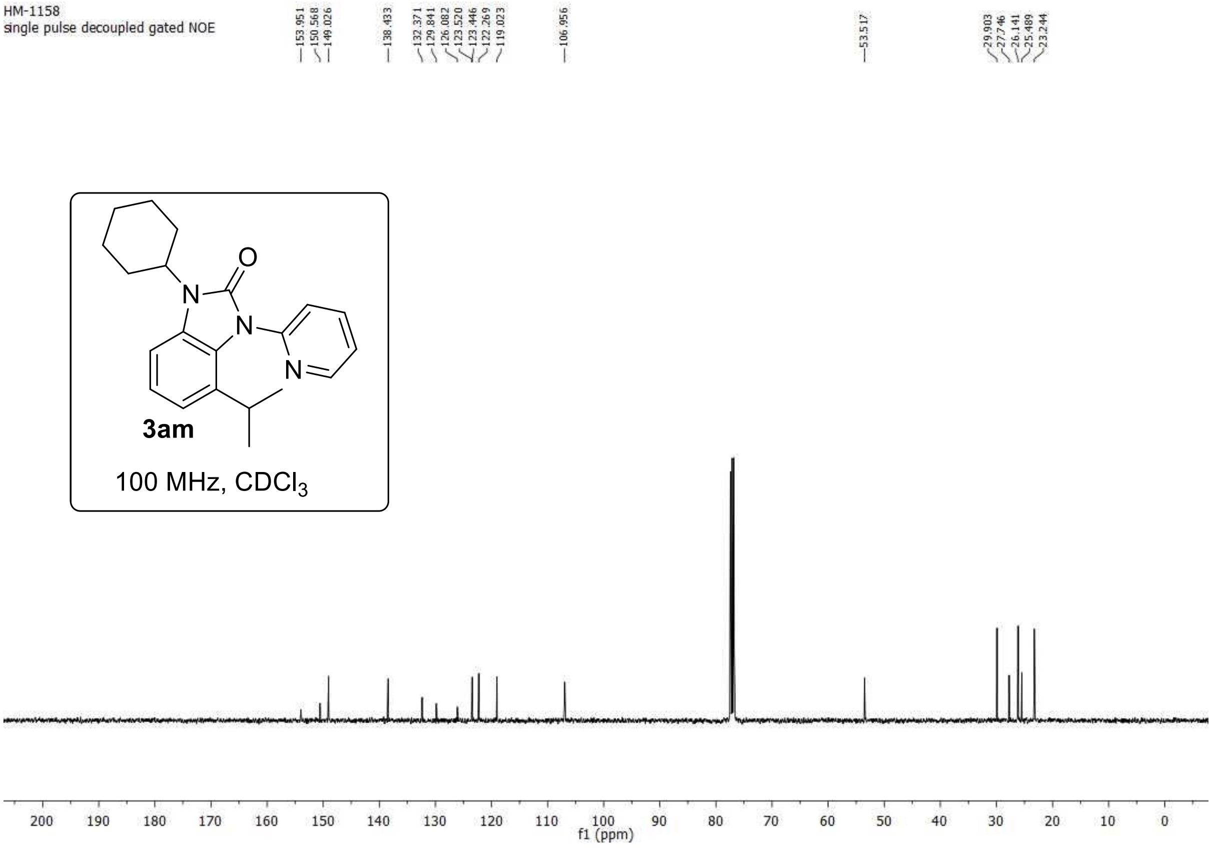
HM-1158
single pulse decoupled gated NOE
153.951 150.568 149.026 138.433 132.371 129.841 126.082 123.520 123.446 122.269 119.023 106.956 53.517 29.903 27.746 26.141 25.489 23.244
3am
100 MHz, CDCl$_3$
f1 (ppm)

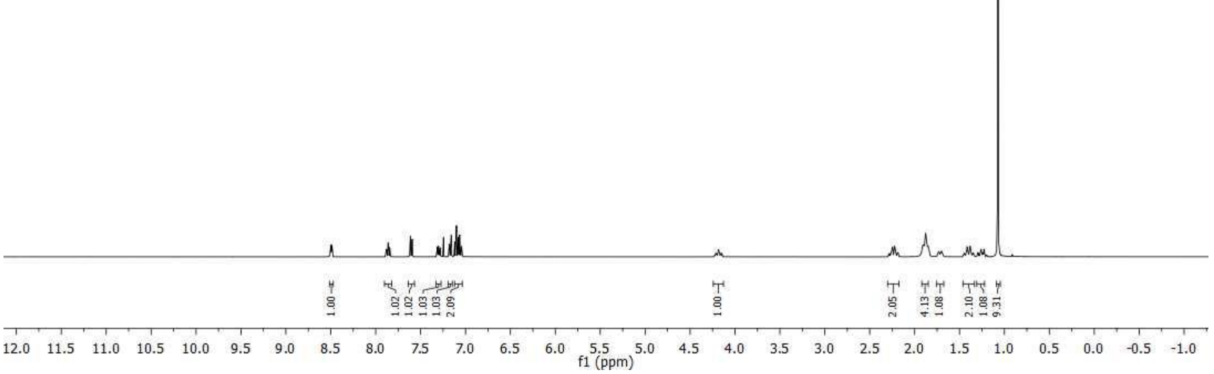

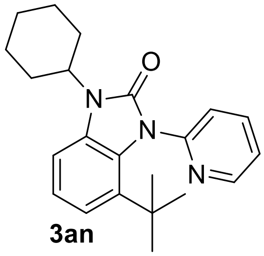

3an

400 MHz, $CDCl_3$

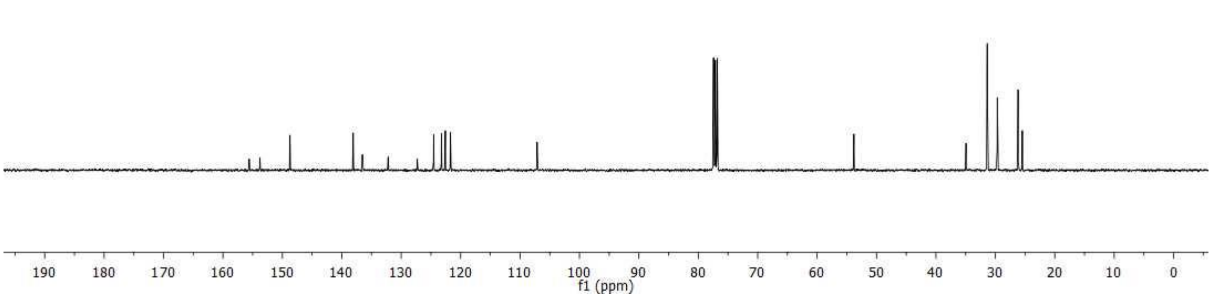

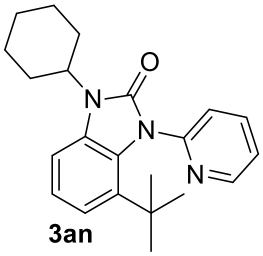

3an

100 MHz, $CDCl_3$

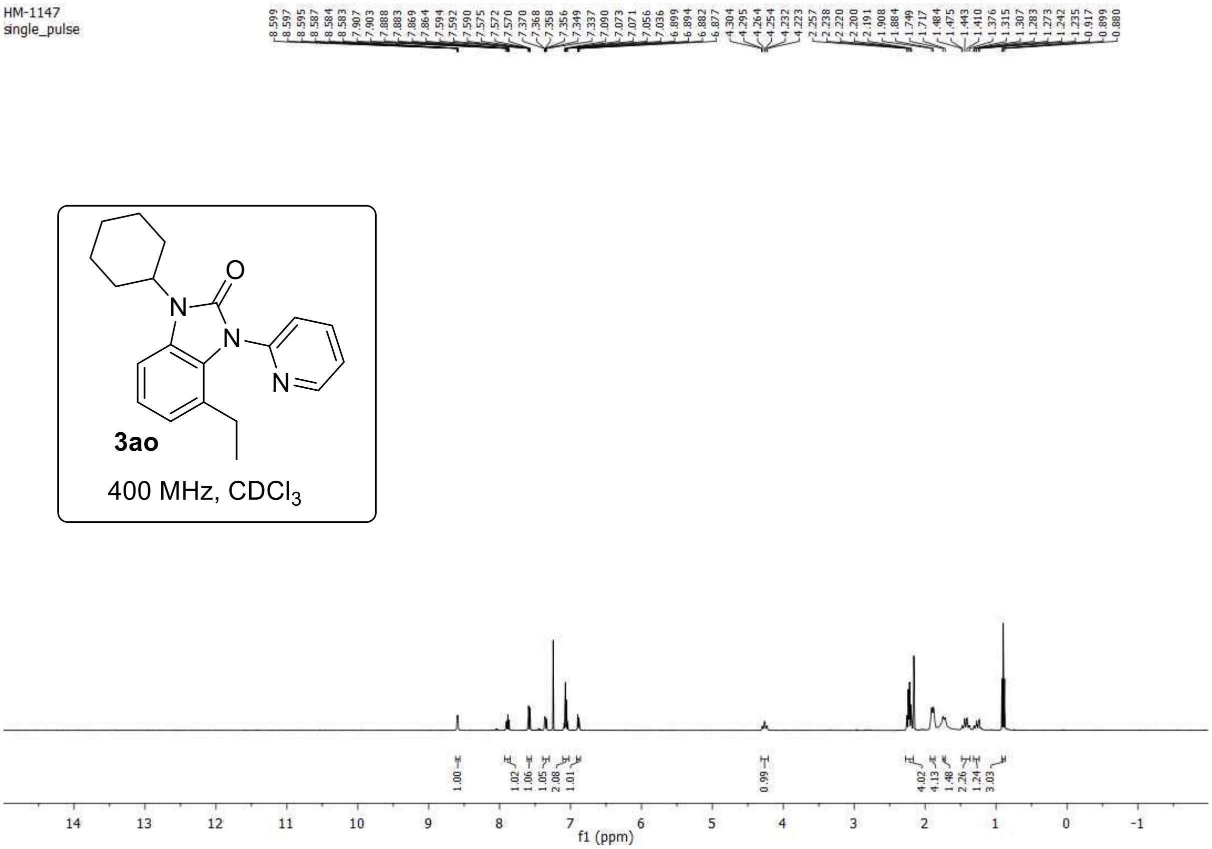

HM-1147
single_pulse
8.599 8.597 8.595 8.587 8.584 8.583 7.907 7.903 7.888 7.883 7.869 7.864 7.594 7.592 7.590 7.575 7.572 7.570 7.370 7.368 7.358 7.356 7.349 7.337 7.090 7.073 7.071 7.056 7.036 6.899 6.894 6.882 6.877 4.304 4.295 4.264 4.254 4.232 4.223 2.257 2.238 2.220 2.200 2.191 1.908 1.884 1.749 1.717 1.484 1.475 1.443 1.410 1.376 1.315 1.307 1.283 1.273 1.242 1.235 0.917 0.899 0.880
3ao
400 MHz, CDCl3
1.00 1.02 1.06 1.05 2.08 1.01 0.99 4.02 4.13 1.48 2.26 1.24 3.03
f1 (ppm)

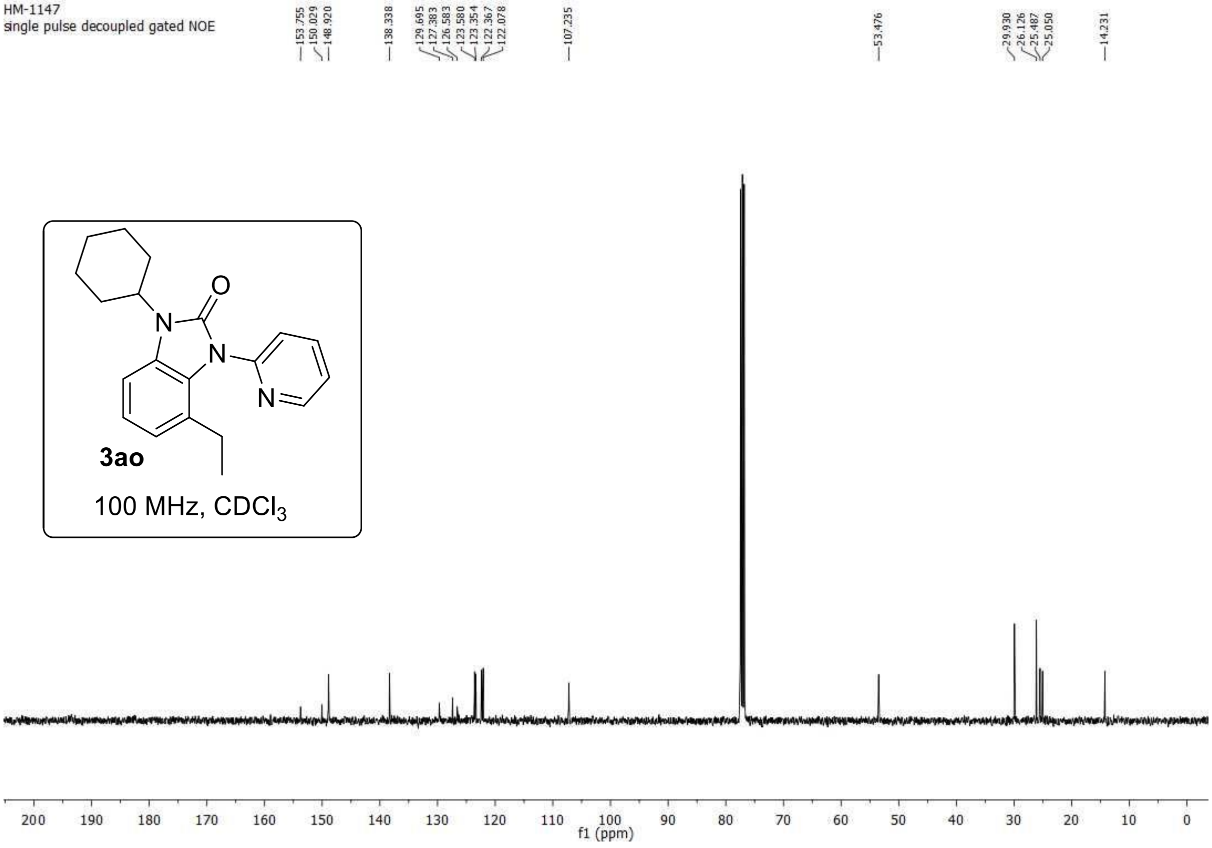

HM-1147
single pulse decoupled gated NOE
153.755 150.029 148.920 138.338 129.695 127.383 126.583 123.580 123.354 122.367 122.078 107.235 53.476 29.930 26.126 25.487 25.050 14.231
3ao
100 MHz, CDCl3
f1 (ppm)

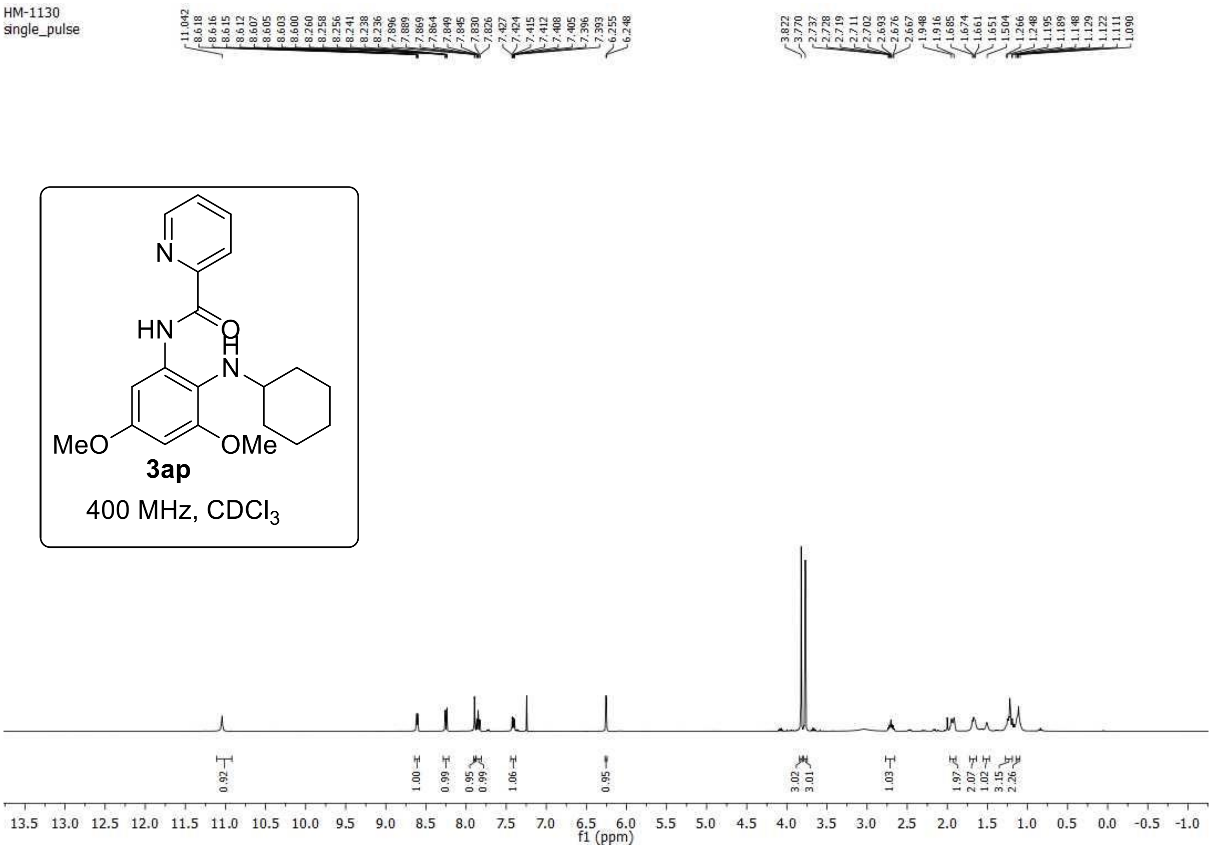
HM-1130
single_pulse
11.042
8.618
8.616
8.615
8.612
8.607
8.605
8.603
8.600
8.260
8.258
8.256
8.241
8.238
8.236
7.896
7.889
7.869
7.864
7.849
7.845
7.830
7.826
7.427
7.424
7.415
7.412
7.408
7.405
7.396
7.393
6.255
6.248
3.822
3.770
2.737
2.728
2.719
2.711
2.702
2.693
2.676
2.667
1.948
1.916
1.685
1.674
1.661
1.651
1.504
1.266
1.248
1.195
1.189
1.148
1.129
1.122
1.111
1.090
N
HN
O
H
N
MeO
OMe
3ap
400 MHz, CDCl3
0.92
1.00
0.99
0.95
0.99
1.06
0.95
3.02
3.01
1.03
1.97
2.07
1.02
3.15
2.26
13.5 13.0 12.5 12.0 11.5 11.0 10.5 10.0 9.5 9.0 8.5 8.0 7.5 7.0 6.5 6.0 5.5 5.0 4.5 4.0 3.5 3.0 2.5 2.0 1.5 1.0 0.5 0.0 -0.5 -1.0
f1 (ppm)

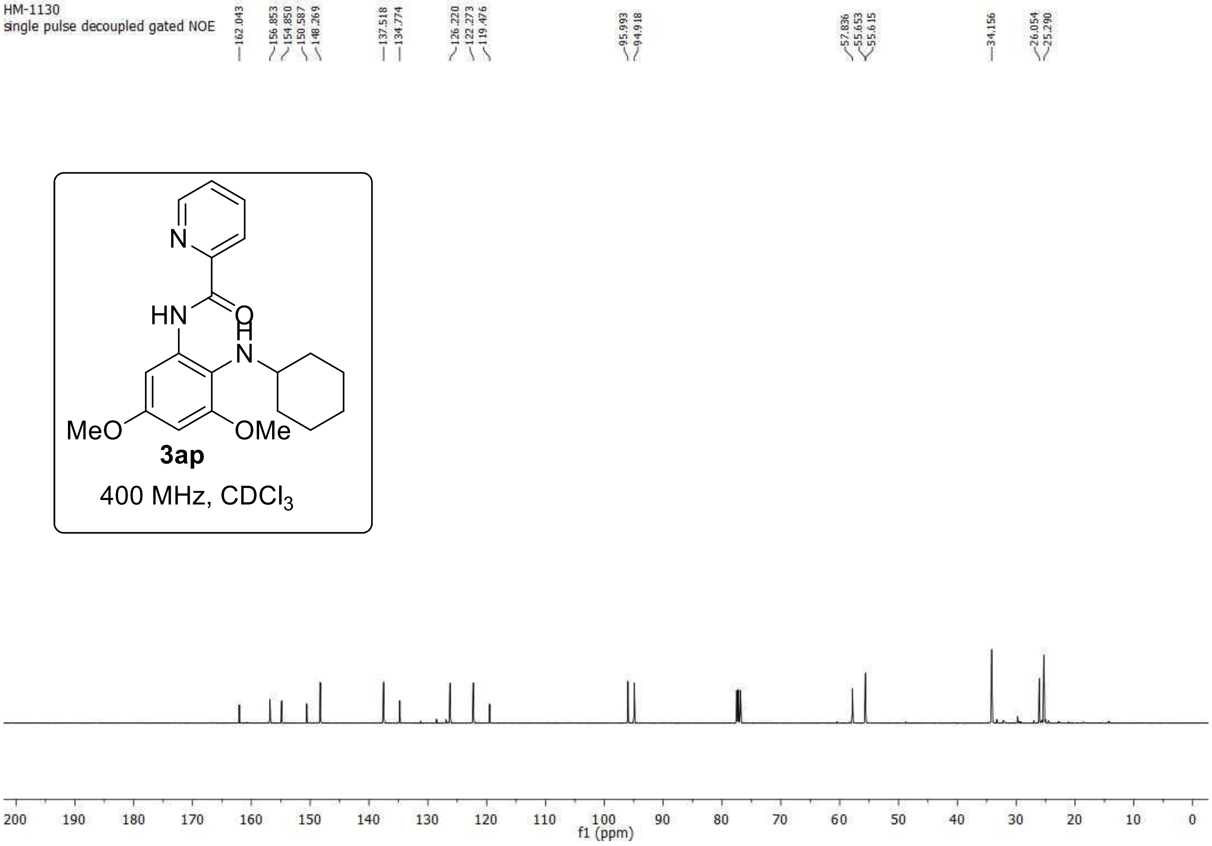
HM-1130
single pulse decoupled gated NOE
162.043
156.853
154.850
150.587
148.269
137.518
134.774
126.220
122.273
119.476
95.993
94.918
57.836
55.653
55.615
34.156
26.054
25.290
N
HN
O
H
N
MeO
OMe
3ap
400 MHz, CDCl3
200 190 180 170 160 150 140 130 120 110 100 90 80 70 60 50 40 30 20 10 0
f1 (ppm)

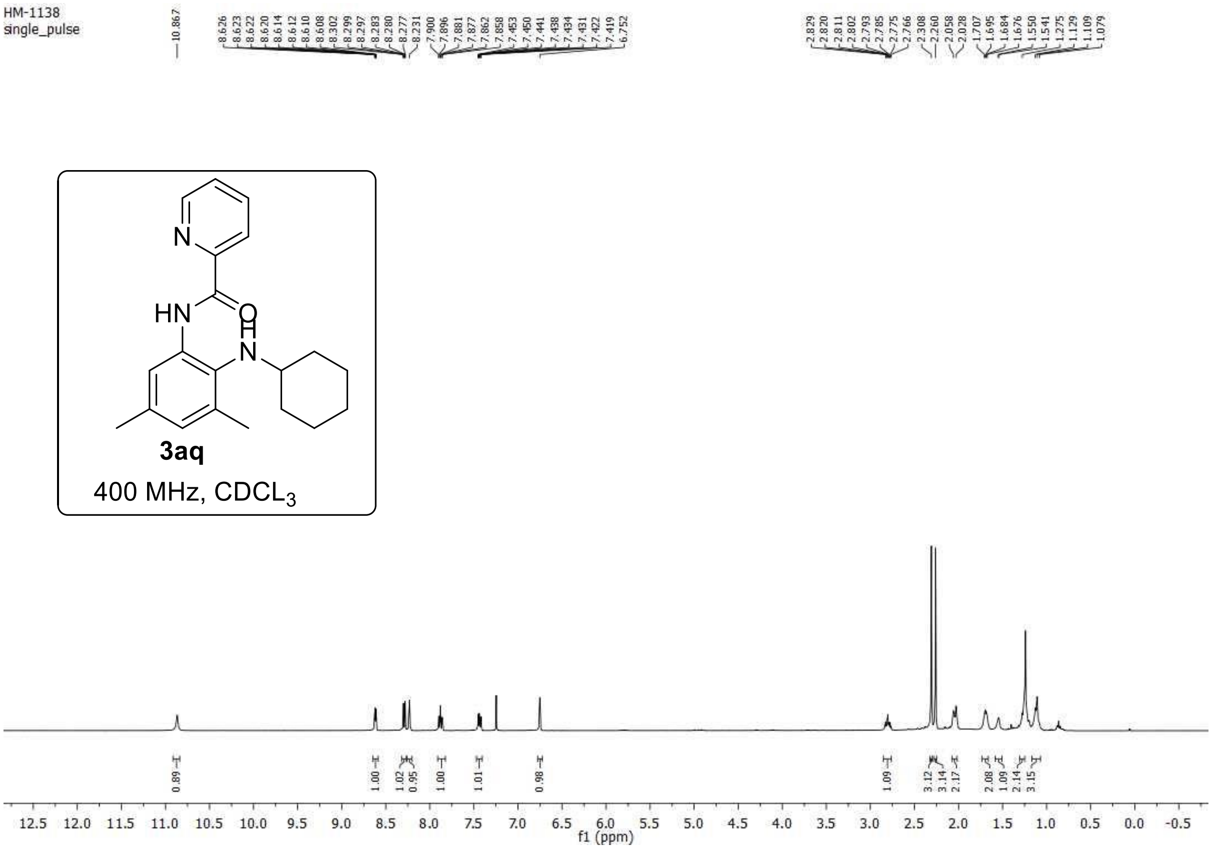
HM-1138
single_pulse
10.867
8.626 8.623 8.622 8.620 8.614 8.612 8.610 8.608 8.302 8.299 8.297 8.283 8.280 8.277 8.231 7.900 7.896 7.881 7.877 7.862 7.858 7.453 7.450 7.441 7.438 7.434 7.431 7.422 7.419 6.752
2.829 2.820 2.811 2.802 2.793 2.785 2.775 2.766 2.308 2.260 2.058 2.028 1.707 1.695 1.684 1.676 1.550 1.541 1.275 1.129 1.109 1.079
N
HN
O
H
N
3aq
400 MHz, CDCL3
0.89 1.00 1.02 0.95 1.00 1.01 0.98 1.09 3.12 3.14 2.17 2.08 1.09 2.14 3.15
12.5 12.0 11.5 11.0 10.5 10.0 9.5 9.0 8.5 8.0 7.5 7.0 6.5 6.0 5.5 5.0 4.5 4.0 3.5 3.0 2.5 2.0 1.5 1.0 0.5 0.0 -0.5
f1 (ppm)

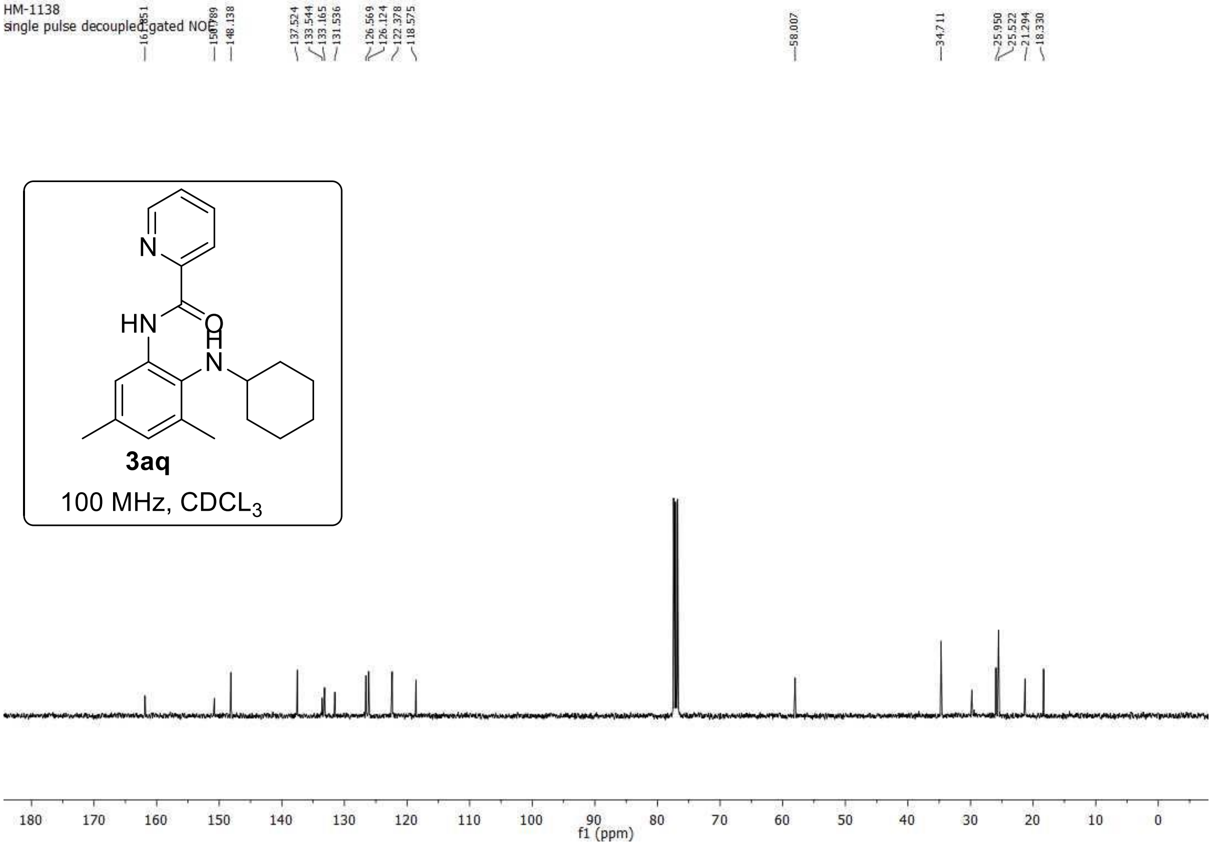
HM-1138
single pulse decoupled gated NOE
161.851 150.789 148.138 137.524 133.544 133.165 131.536 126.569 126.124 122.378 118.575 58.007 34.711 25.950 25.522 21.294 18.330
N
HN
O
H
N
3aq
100 MHz, CDCL3
180 170 160 150 140 130 120 110 100 90 80 70 60 50 40 30 20 10 0
f1 (ppm)

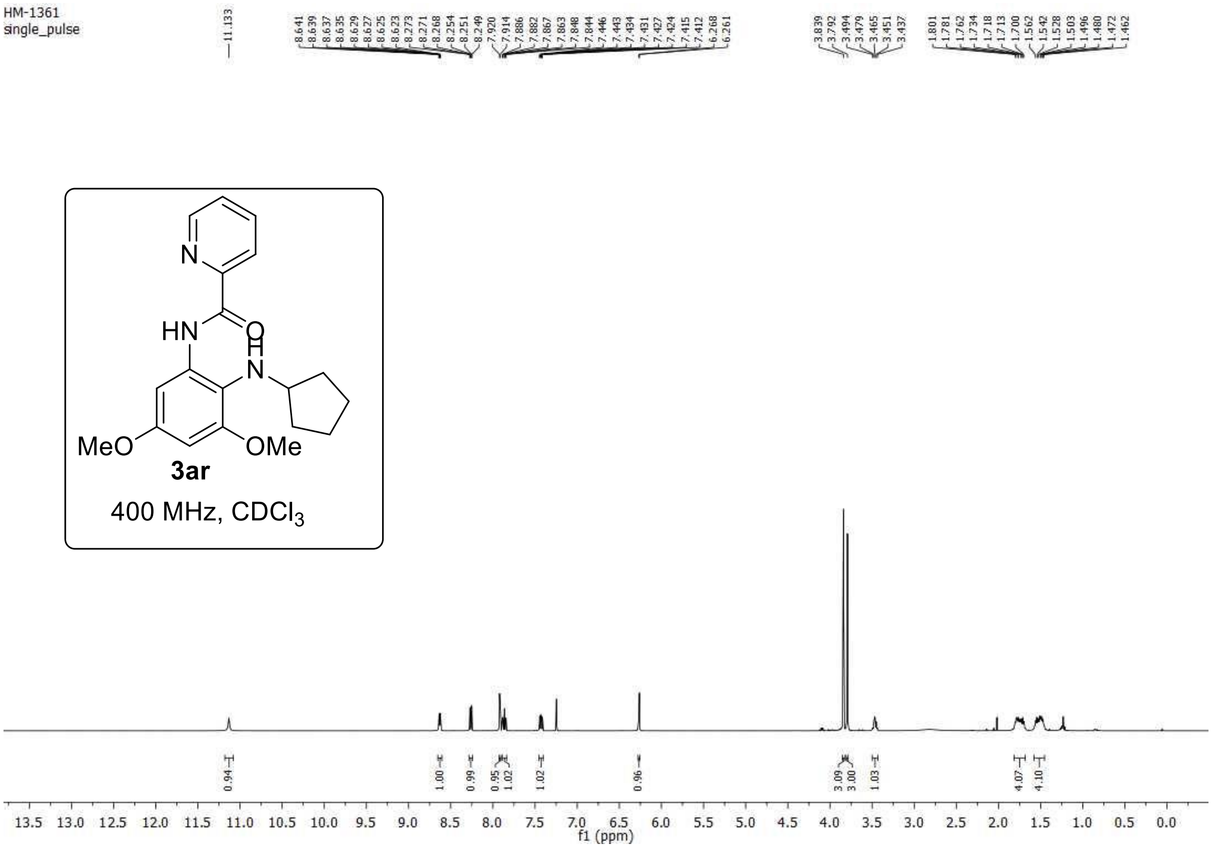
HM-1361
single_pulse
11.133
8.641 8.639 8.637 8.635 8.629 8.627 8.625 8.623 8.273 8.271 8.268 8.254 8.251 8.249 7.920 7.914 7.886 7.882 7.867 7.863 7.848 7.844 7.446 7.443 7.434 7.431 7.427 7.424 7.415 7.412 6.268 6.261
3.839 3.792 3.494 3.479 3.465 3.451 3.437
1.801 1.781 1.762 1.734 1.718 1.713 1.700 1.562 1.542 1.528 1.503 1.496 1.480 1.472 1.462
N
HN
O
H
N
MeO
OMe
3ar
400 MHz, CDCl3
0.94 1.00 0.99 0.95 1.02 1.02 0.96 3.09 3.00 1.03 4.07 4.10
f1 (ppm)

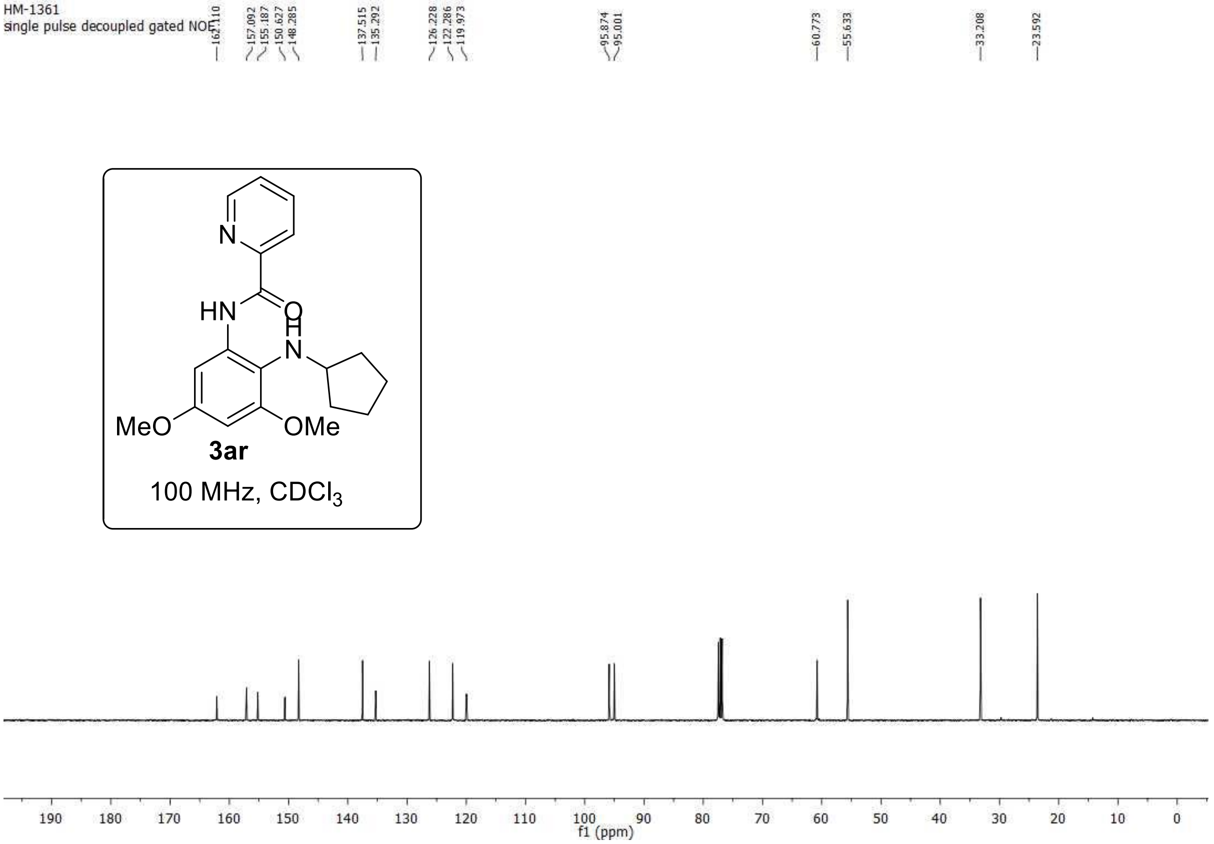
HM-1361
single pulse decoupled gated NOE
162.110 157.092 155.187 150.627 148.285 137.515 135.292 126.228 122.286 119.973 95.874 95.001 60.773 55.633 33.208 23.592
N
HN
O
H
N
MeO
OMe
3ar
100 MHz, CDCl3
f1 (ppm)

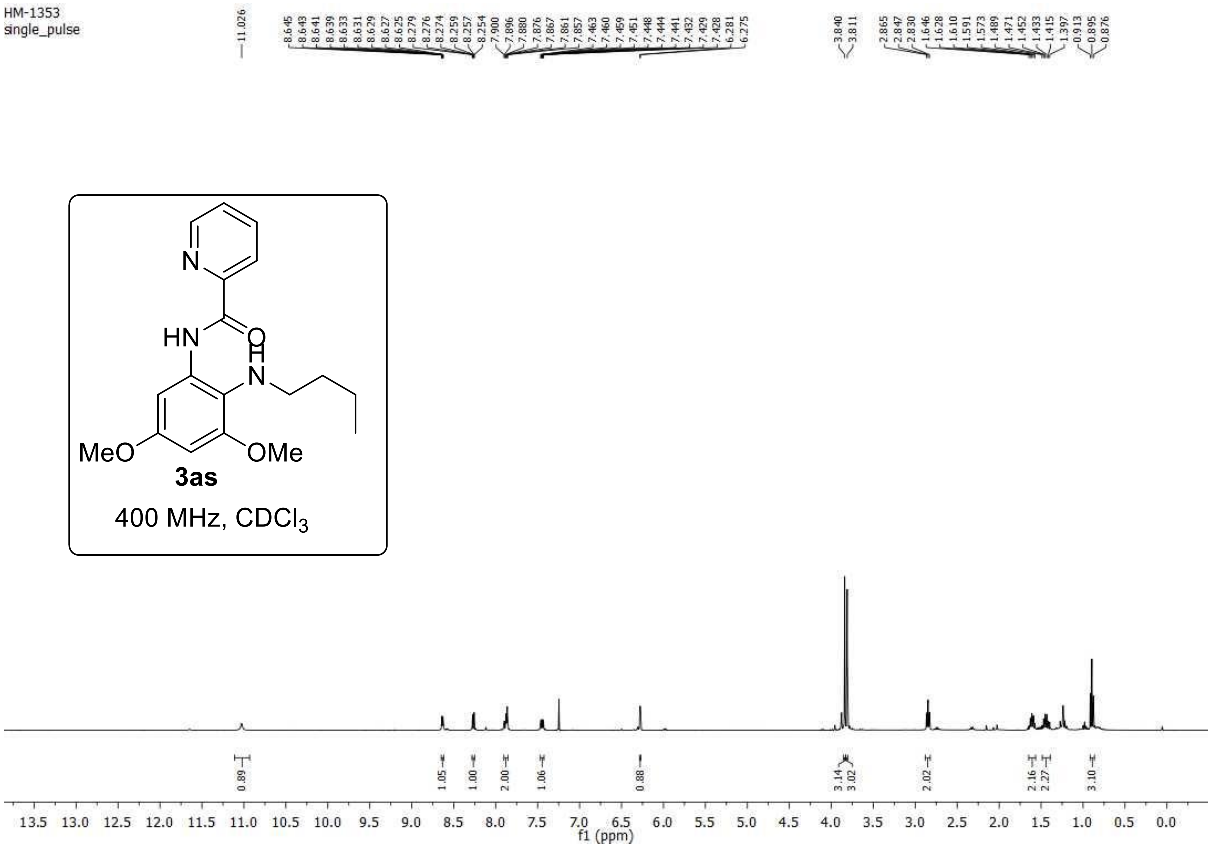

HM-1353
single_pulse
3as
400 MHz, CDCl3
f1 (ppm)

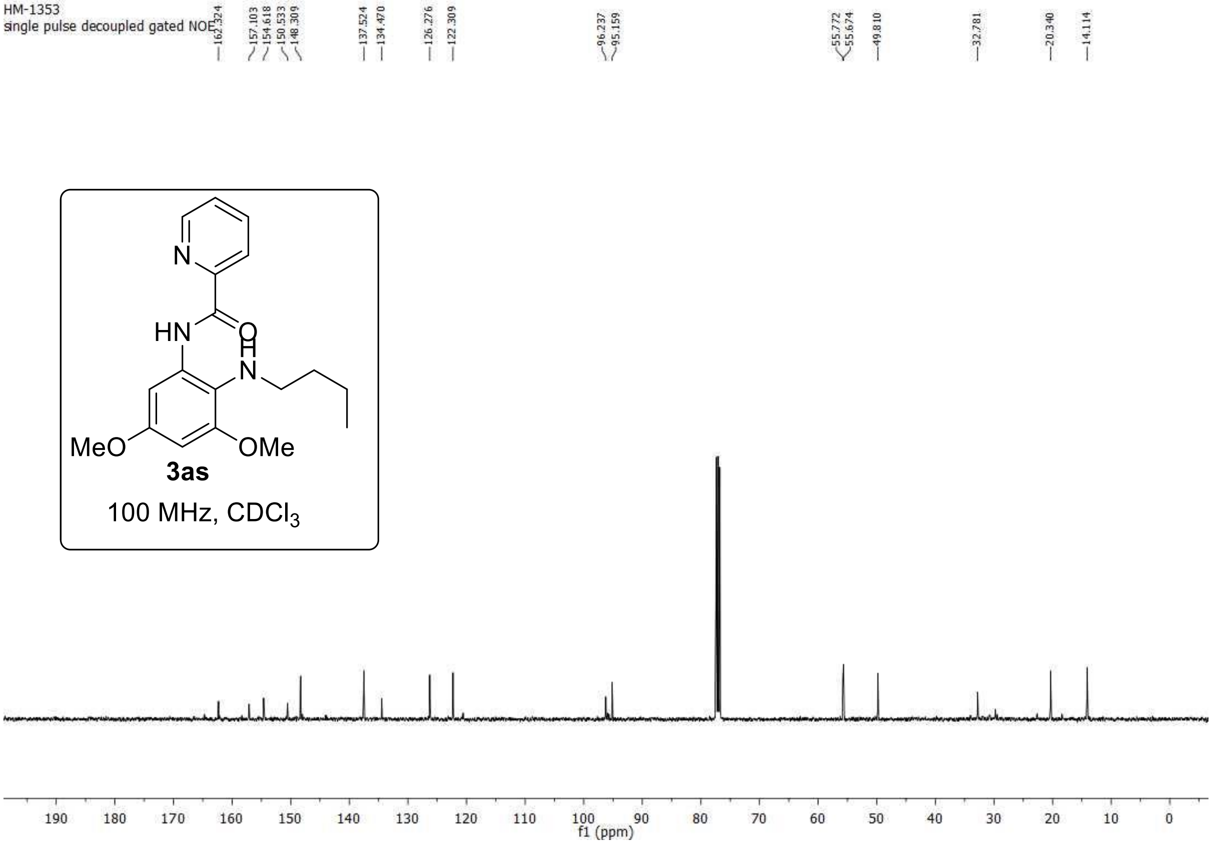

HM-1353
single pulse decoupled gated NOE
3as
100 MHz, CDCl3
f1 (ppm)

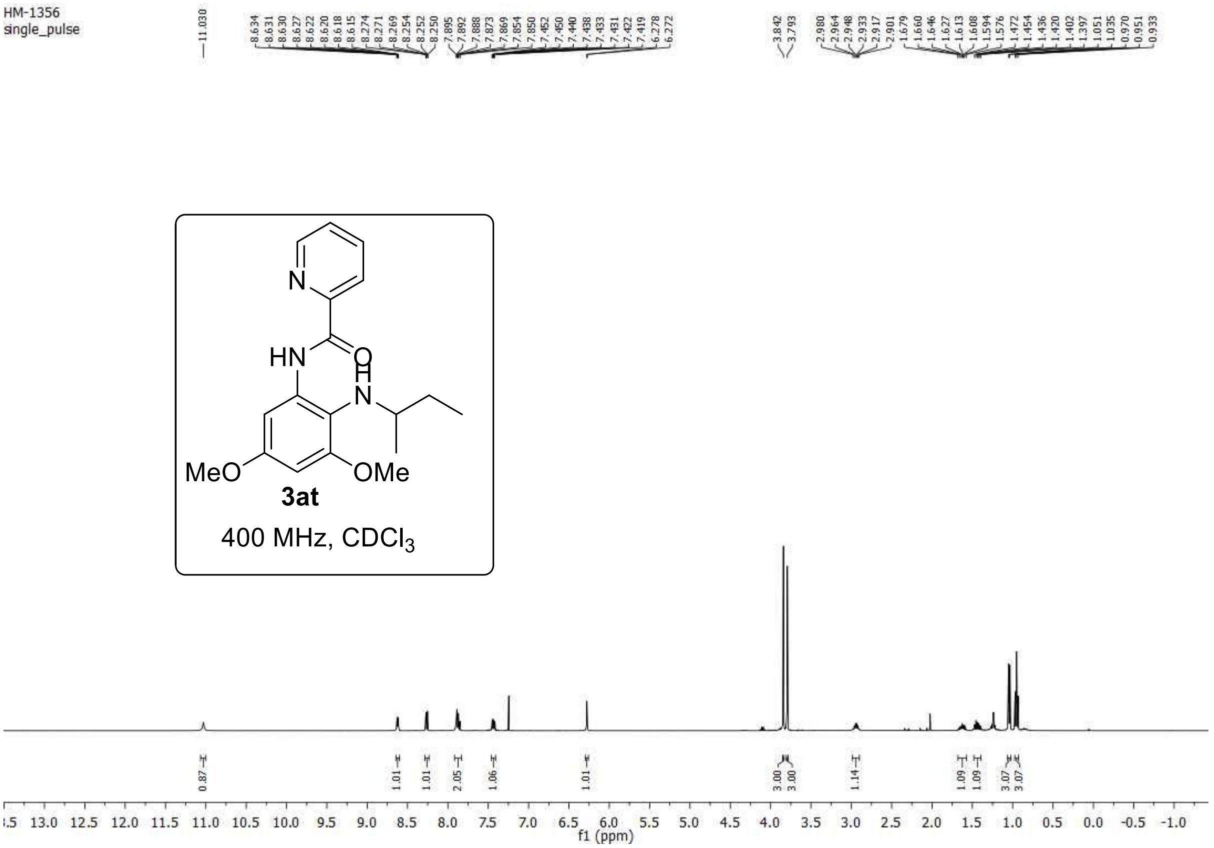

HM-1356
single_pulse
11.030
8.634 8.631 8.630 8.627 8.622 8.620 8.618 8.615 8.274 8.271 8.269 8.254 8.252 8.250 7.895 7.892 7.888 7.873 7.869 7.854 7.850 7.452 7.450 7.440 7.438 7.431 7.422 7.419 6.278 6.272
3.842 3.793
2.980 2.964 2.948 2.933 2.917 2.901 1.679 1.660 1.646 1.627 1.613 1.608 1.594 1.576 1.472 1.454 1.436 1.420 1.402 1.397 1.051 1.035 0.970 0.951 0.933
N
HN
O
H
N
MeO
OMe
3at
400 MHz, CDCl3
0.87 1.01 1.01 2.05 1.06 1.01 3.00 3.00 1.14 1.09 1.09 3.07 3.07
13.0 12.5 12.0 11.5 11.0 10.5 10.0 9.5 9.0 8.5 8.0 7.5 7.0 6.5 6.0 5.5 5.0 4.5 4.0 3.5 3.0 2.5 2.0 1.5 1.0 0.5 0.0 -0.5 -1.0
f1 (ppm)

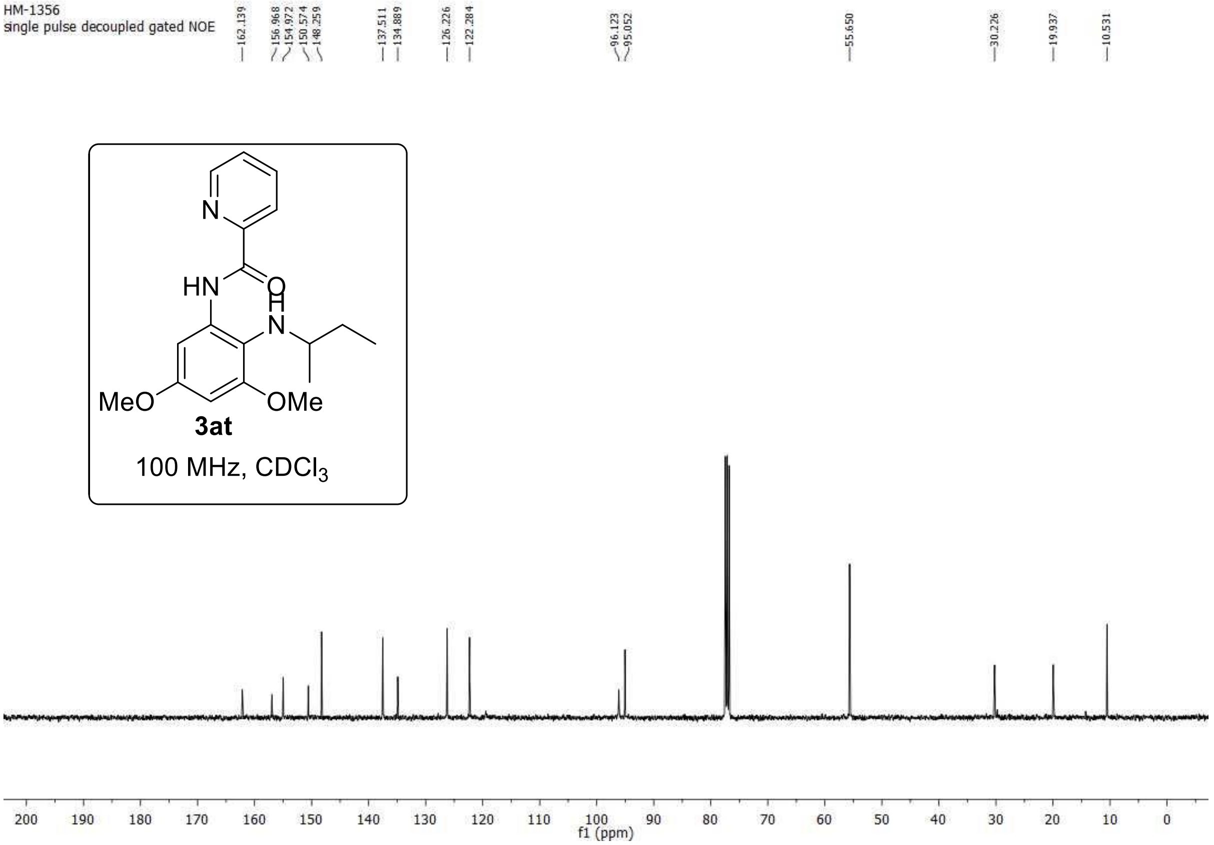

HM-1356
single pulse decoupled gated NOE
162.139 156.968 154.972 150.574 148.259 137.511 134.889 126.226 122.284 96.123 95.052 55.650 30.226 19.937 10.531
N
HN
O
H
N
MeO
OMe
3at
100 MHz, CDCl3
200 190 180 170 160 150 140 130 120 110 100 90 80 70 60 50 40 30 20 10 0
f1 (ppm)

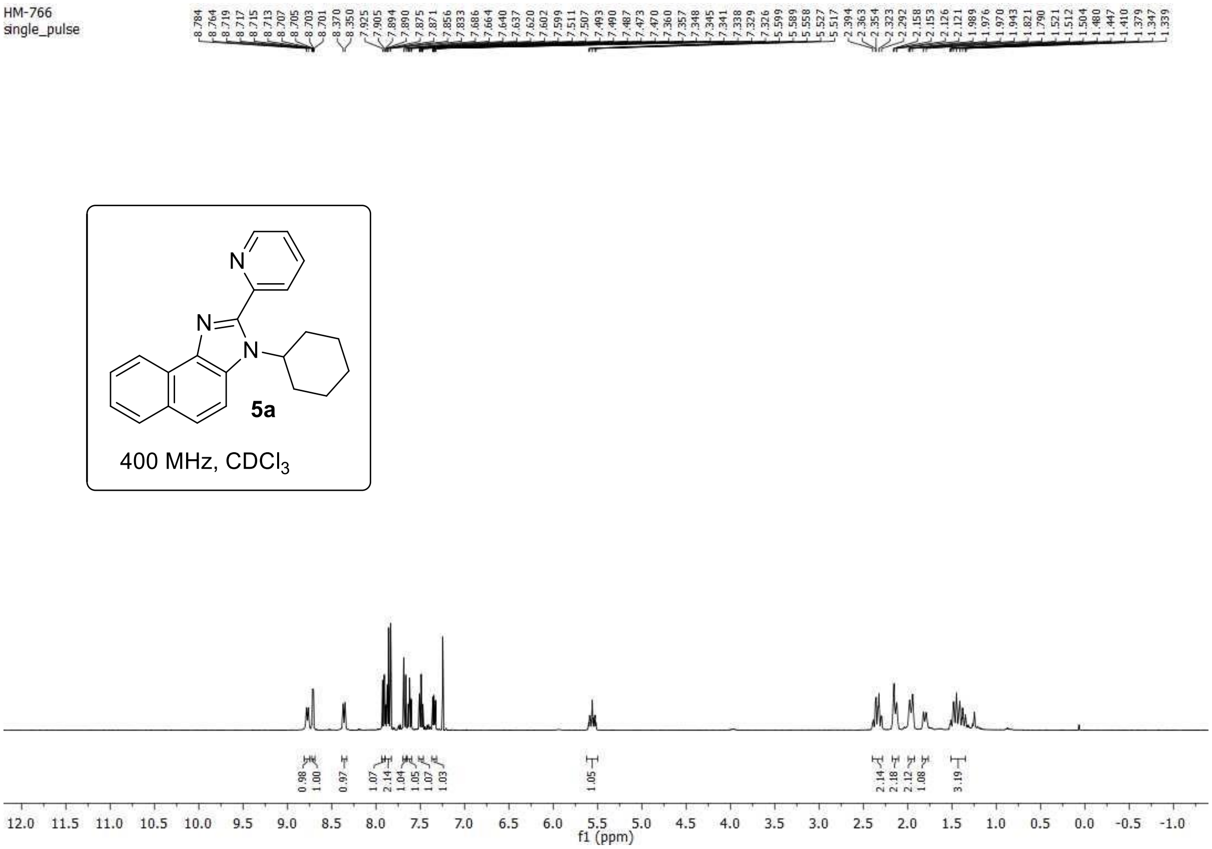
HM-766
single_pulse
8.784 8.764 8.719 8.717 8.715 8.713 8.707 8.705 8.703 8.701 8.370 8.350 7.925 7.905 7.894 7.890 7.875 7.871 7.856 7.833 7.686 7.664 7.640 7.637 7.620 7.602 7.599 7.511 7.507 7.493 7.490 7.487 7.473 7.470 7.360 7.357 7.348 7.345 7.341 7.338 7.329 7.326 5.599 5.589 5.558 5.527 5.517 2.394 2.363 2.354 2.323 2.292 2.158 2.153 2.126 2.121 1.989 1.976 1.970 1.943 1.821 1.790 1.521 1.512 1.504 1.480 1.447 1.410 1.379 1.347 1.339
N
N
N
5a
400 MHz, CDCl3
0.98 1.00 0.97 1.07 2.14 1.04 1.05 1.07 1.03 1.05 2.14 2.18 2.12 1.08 3.19
12.0 11.5 11.0 10.5 10.0 9.5 9.0 8.5 8.0 7.5 7.0 6.5 6.0 5.5 5.0 4.5 4.0 3.5 3.0 2.5 2.0 1.5 1.0 0.5 0.0 -0.5 -1.0
f1 (ppm)

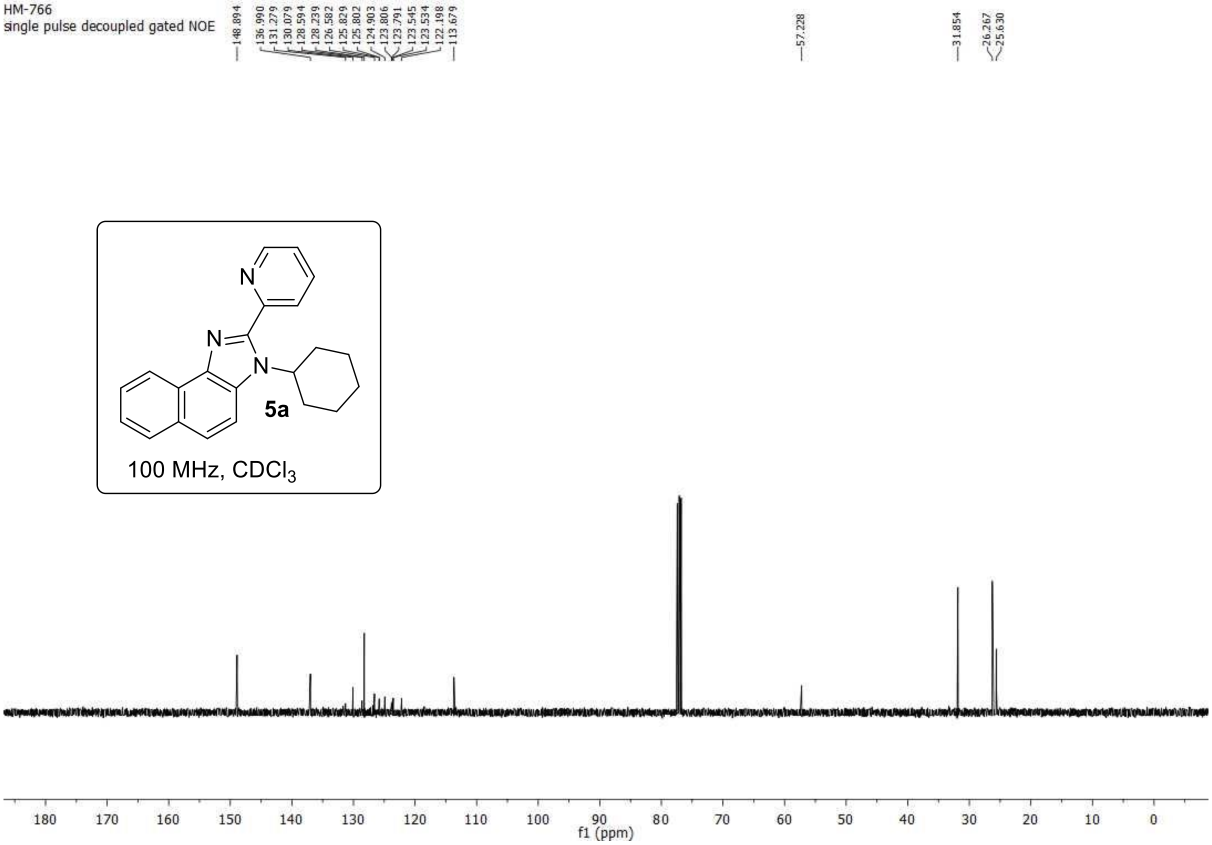
HM-766
single pulse decoupled gated NOE
148.894 136.990 131.279 130.079 128.594 128.239 126.582 125.829 125.802 124.903 123.806 123.791 123.545 123.534 122.198 113.679
57.228
31.854 26.267 25.630
N
N
N
5a
100 MHz, CDCl3
180 170 160 150 140 130 120 110 100 90 80 70 60 50 40 30 20 10 0
f1 (ppm)

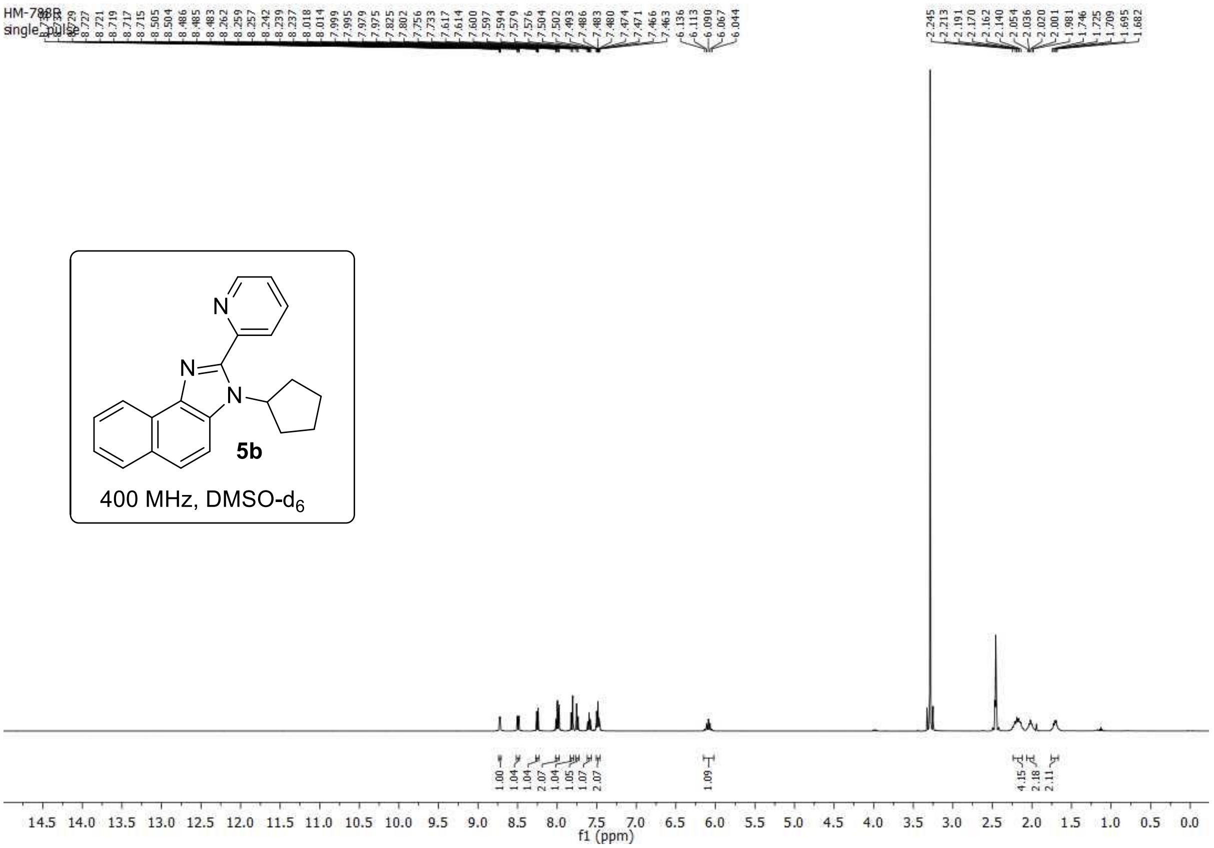
HM-788R
single_pulse
5b
400 MHz, DMSO-d$_6$
f1 (ppm)

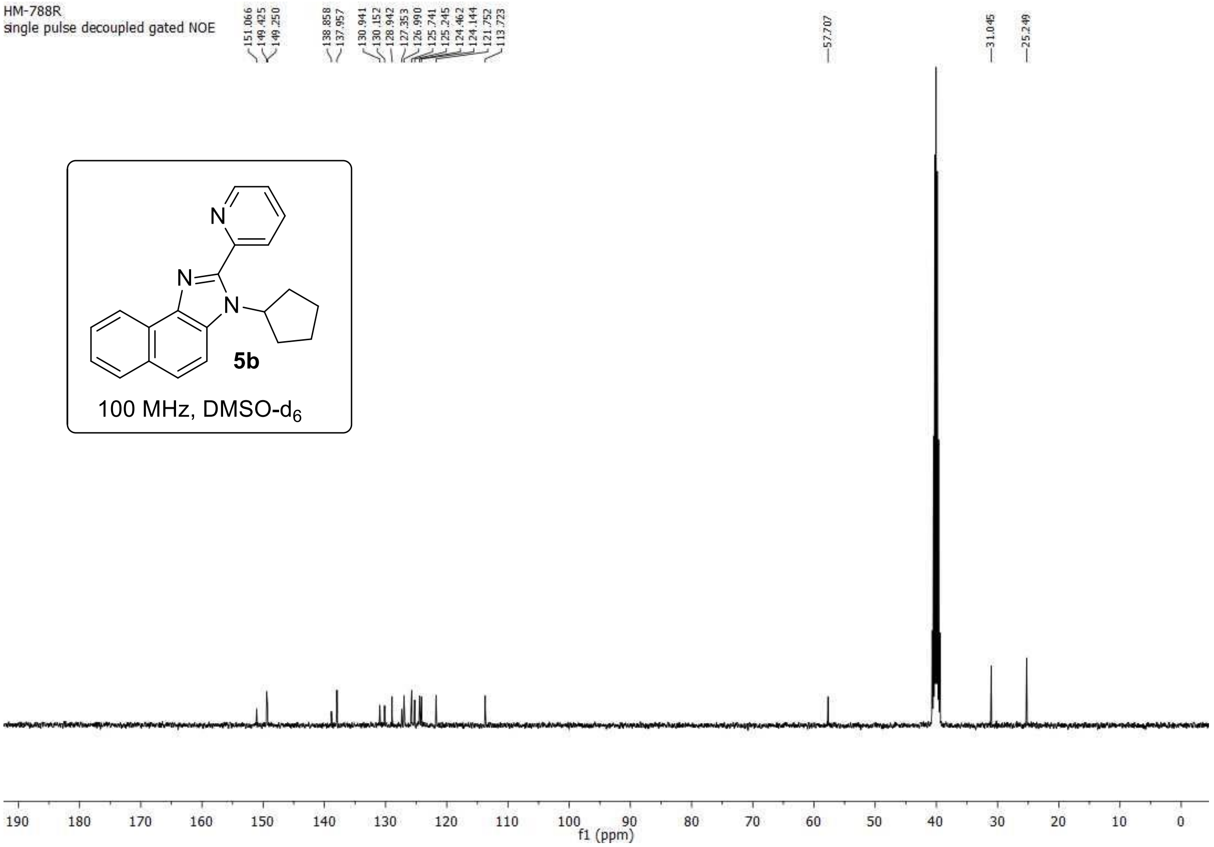
HM-788R
single pulse decoupled gated NOE
5b
100 MHz, DMSO-d$_6$
f1 (ppm)

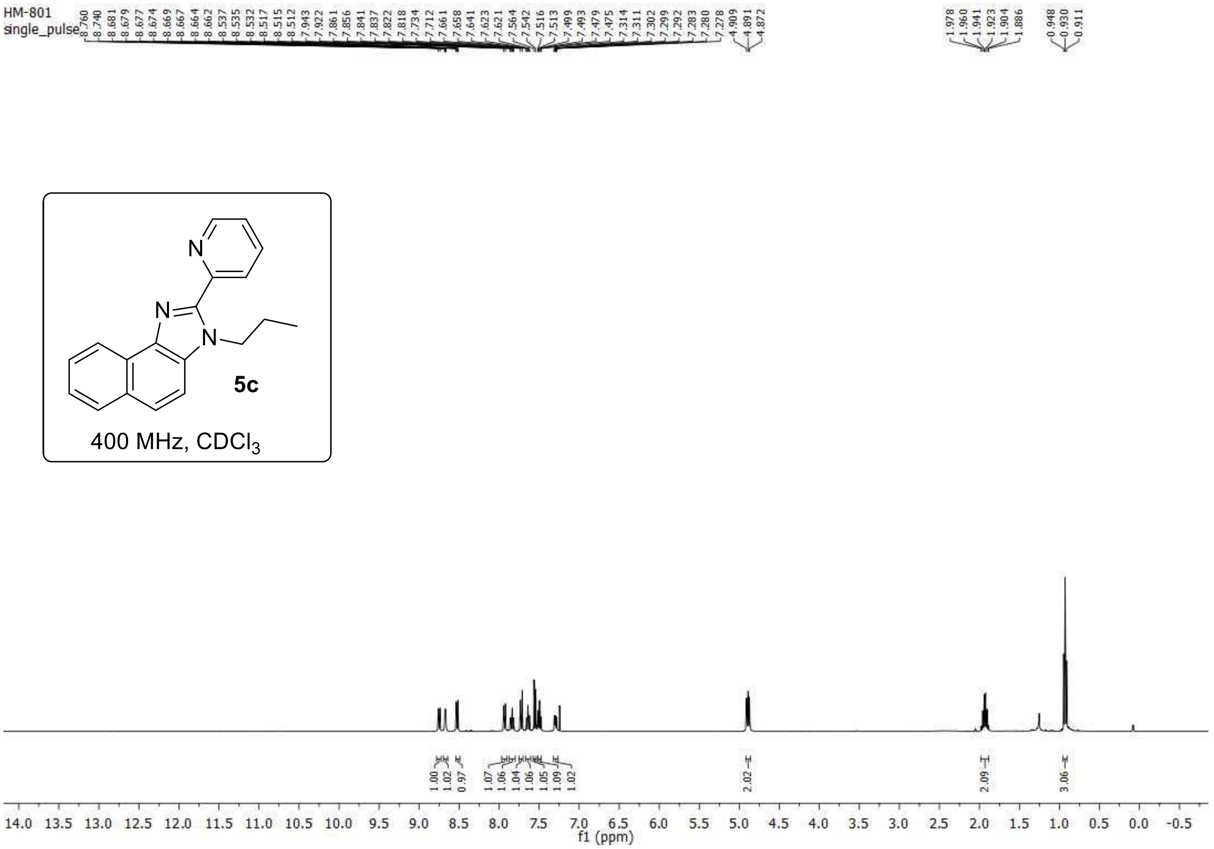
HM-801
single_pulse
8.760 8.740 8.681 8.679 8.677 8.674 8.669 8.667 8.664 8.662 8.537 8.535 8.532 8.517 8.515 8.512 7.943 7.922 7.861 7.856 7.841 7.837 7.822 7.818 7.734 7.712 7.661 7.658 7.641 7.623 7.621 7.564 7.542 7.516 7.513 7.499 7.493 7.479 7.475 7.314 7.311 7.302 7.299 7.292 7.283 7.280 7.278 4.909 4.891 4.872
1.978 1.960 1.941 1.923 1.904 1.886
0.948 0.930 0.911
N
N
N
5c
400 MHz, $CDCl_3$
1.00 1.02 0.97 1.07 1.06 1.04 1.06 1.05 1.09 1.02
2.02
2.09
3.06
14.0 13.5 13.0 12.5 12.0 11.5 11.0 10.5 10.0 9.5 9.0 8.5 8.0 7.5 7.0 6.5 6.0 5.5 5.0 4.5 4.0 3.5 3.0 2.5 2.0 1.5 1.0 0.5 0.0 -0.5
f1 (ppm)

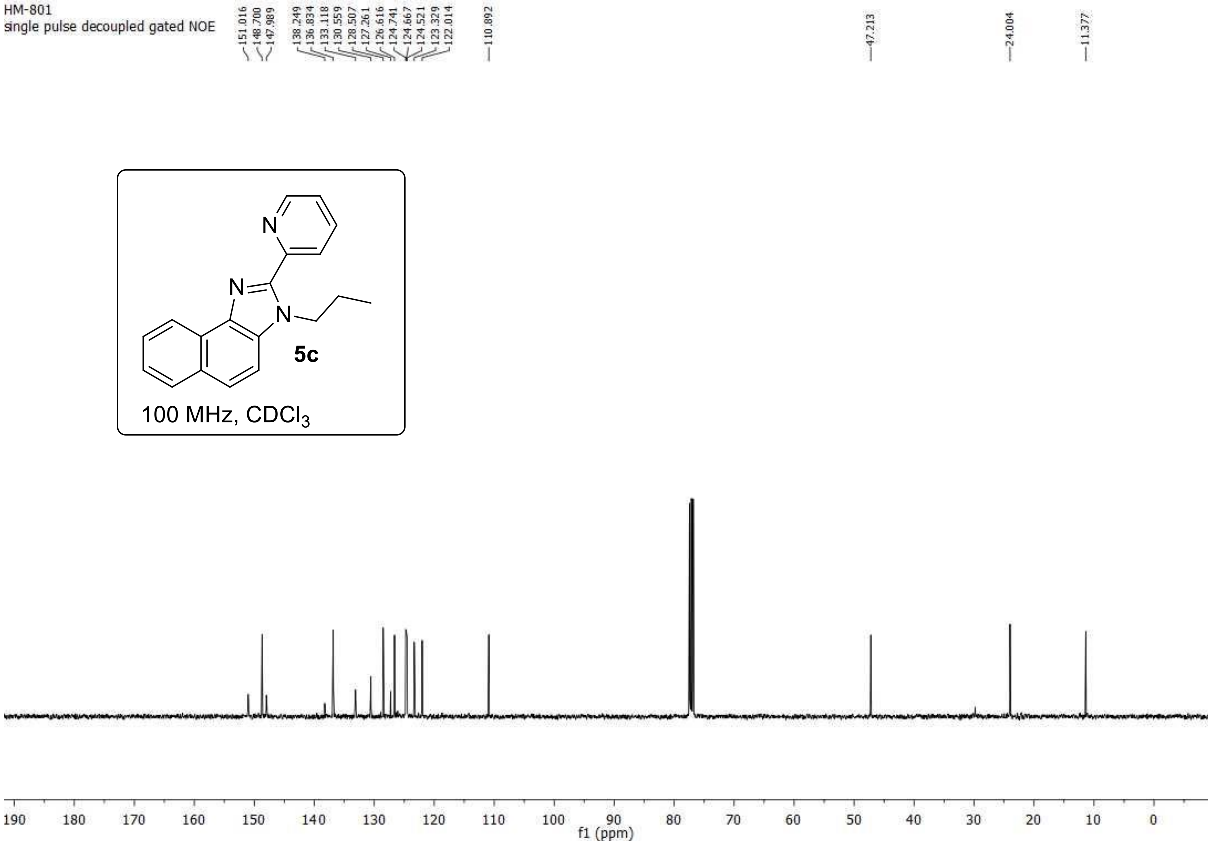
HM-801
single pulse decoupled gated NOE
151.016 148.700 147.989 138.249 136.834 133.118 130.559 128.507 127.261 126.616 124.741 124.667 124.521 123.329 122.014
110.892
47.213
24.004
11.377
N
N
N
5c
100 MHz, $CDCl_3$
190 180 170 160 150 140 130 120 110 100 90 80 70 60 50 40 30 20 10 0
f1 (ppm)

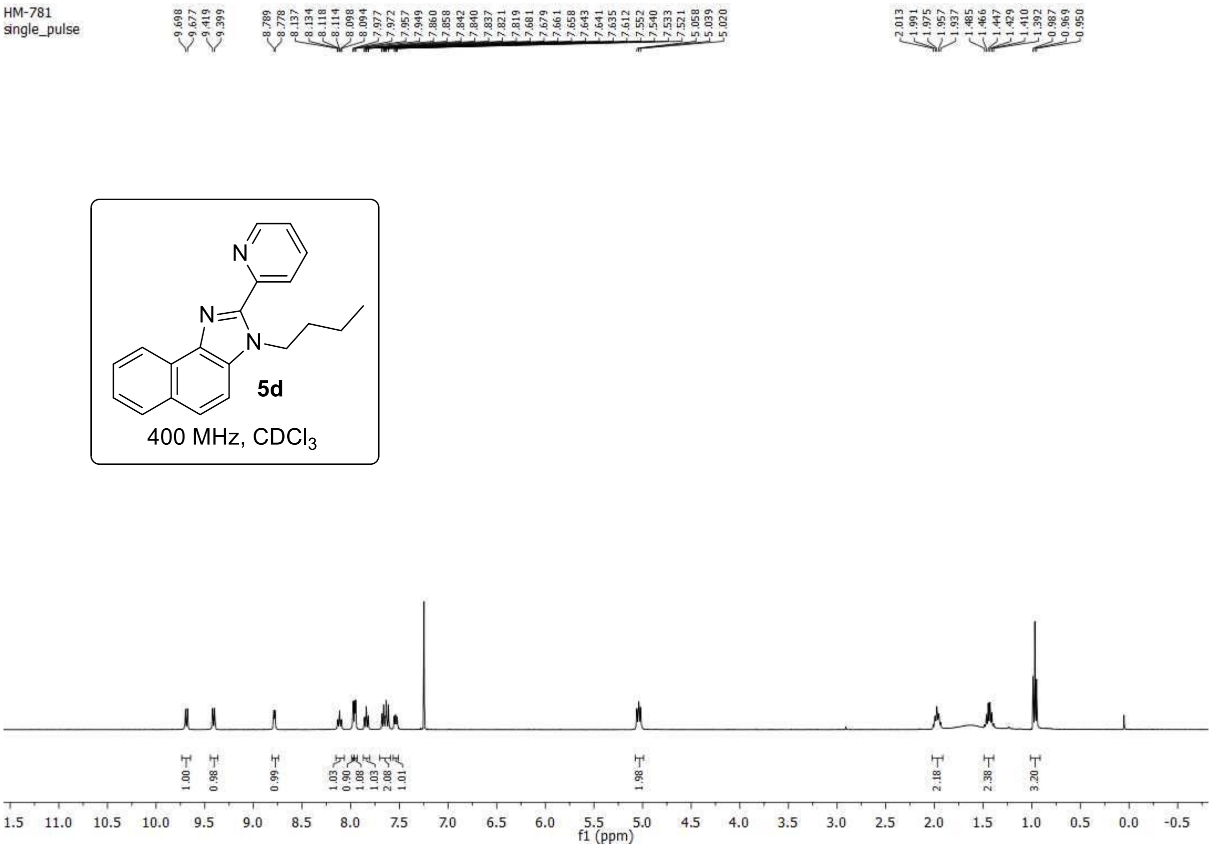

HM-781
single_pulse
9.698 9.677 9.419 9.399 8.789 8.778 8.137 8.134 8.118 8.114 8.098 8.094 7.977 7.972 7.957 7.949 7.860 7.858 7.842 7.840 7.837 7.821 7.819 7.681 7.679 7.661 7.658 7.643 7.641 7.635 7.612 7.552 7.540 7.533 7.521 5.058 5.039 5.020
2.013 1.991 1.975 1.957 1.937 1.485 1.466 1.447 1.429 1.410 1.392 0.987 0.969 0.950
N
N
N
5d
400 MHz, CDCl3
1.00 0.98 0.99 1.03 0.90 1.08 1.03 2.08 1.01 1.98 2.18 2.38 3.20
f1 (ppm)

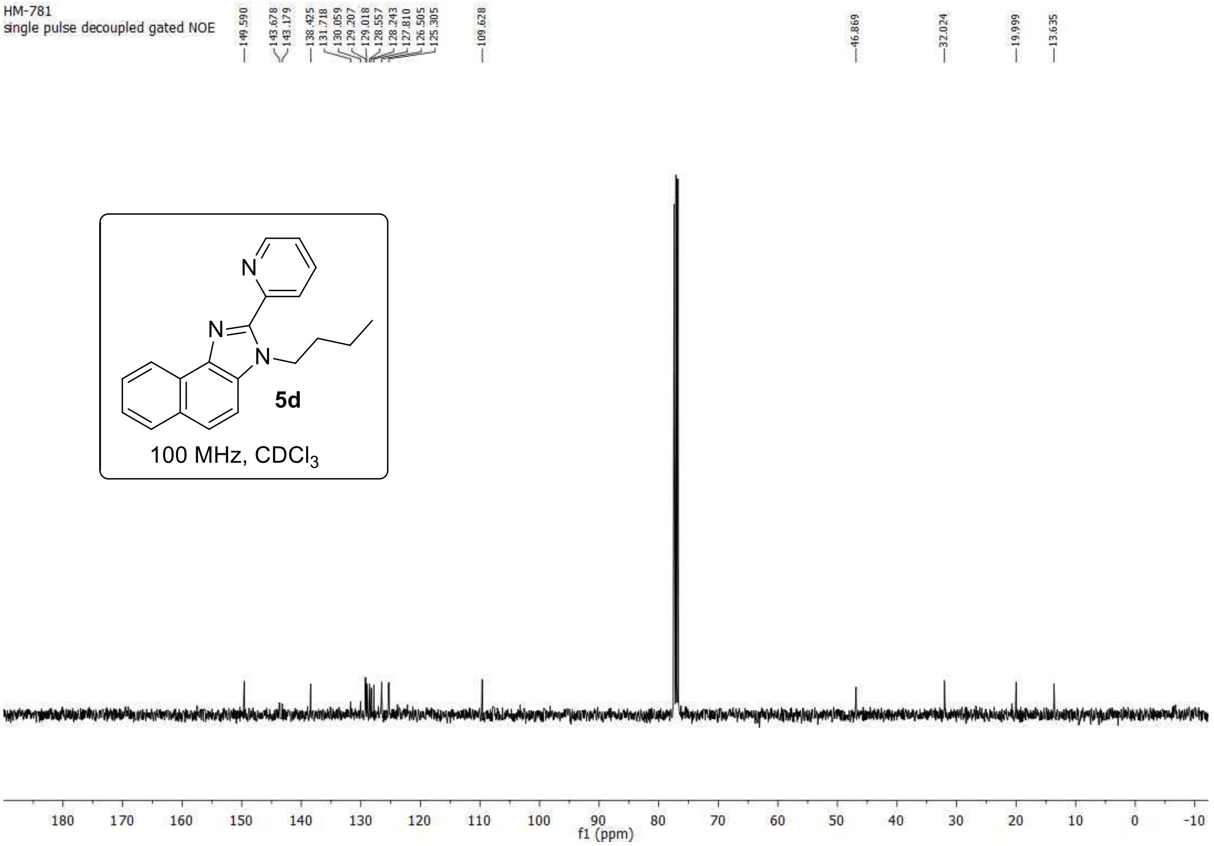

HM-781
single pulse decoupled gated NOE
149.590 143.678 143.179 138.425 131.718 130.059 129.207 129.018 128.557 128.243 127.810 126.505 125.305 109.628 46.869 32.024 19.999 13.635
N
N
N
5d
100 MHz, CDCl3
f1 (ppm)

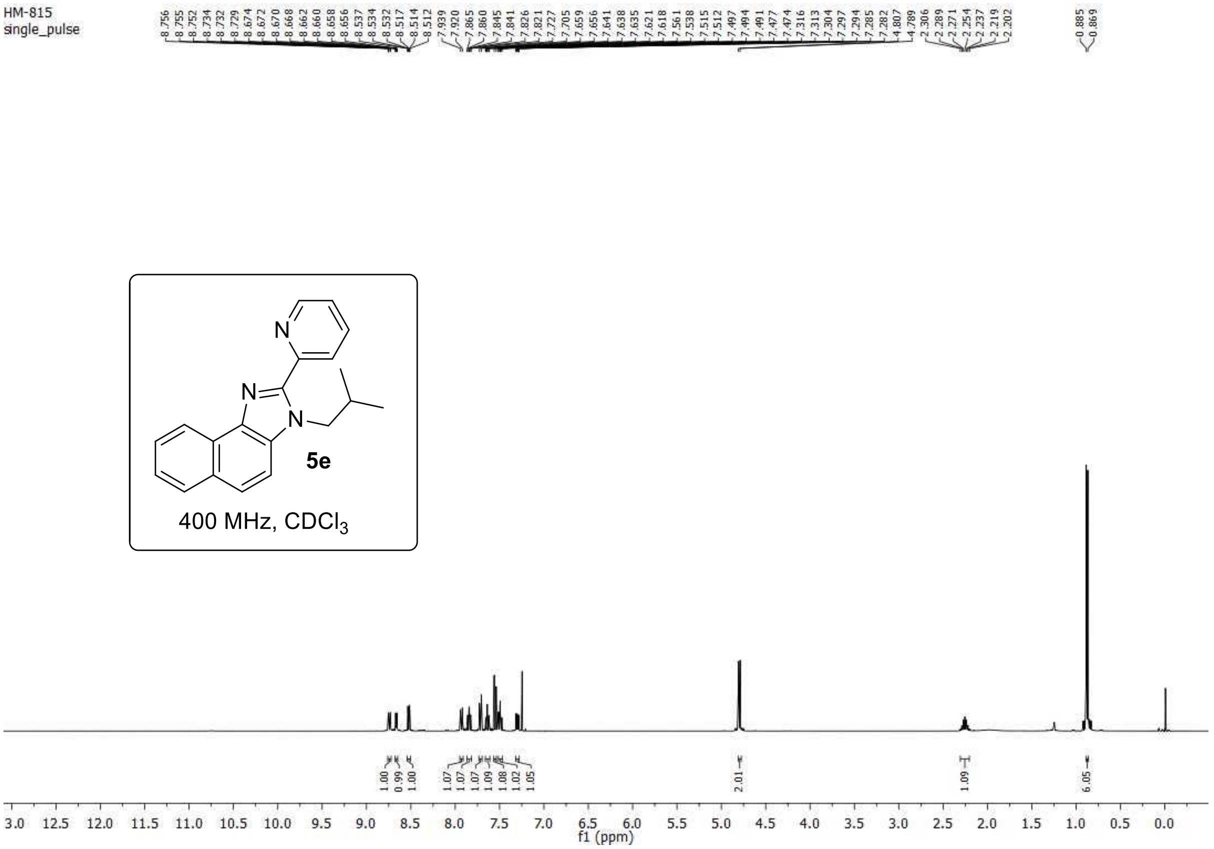
HM-815
single_pulse
8.756 8.755 8.752 8.734 8.732 8.729 8.674 8.672 8.670 8.668 8.662 8.660 8.658 8.656 8.537 8.534 8.532 8.517 8.514 8.512 7.939 7.920 7.865 7.860 7.845 7.841 7.826 7.821 7.727 7.705 7.659 7.656 7.641 7.638 7.635 7.621 7.618 7.561 7.538 7.515 7.512 7.497 7.494 7.491 7.477 7.474 7.316 7.313 7.304 7.297 7.294 7.285 7.282 4.807 4.789 2.306 2.289 2.271 2.254 2.237 2.219 2.202
0.885 0.869
N
N
N
5e
400 MHz, CDCl3
1.00 0.99 1.00 1.07 1.07 1.07 1.09 1.08 1.02 1.05 2.01 1.09 6.05
f1 (ppm)

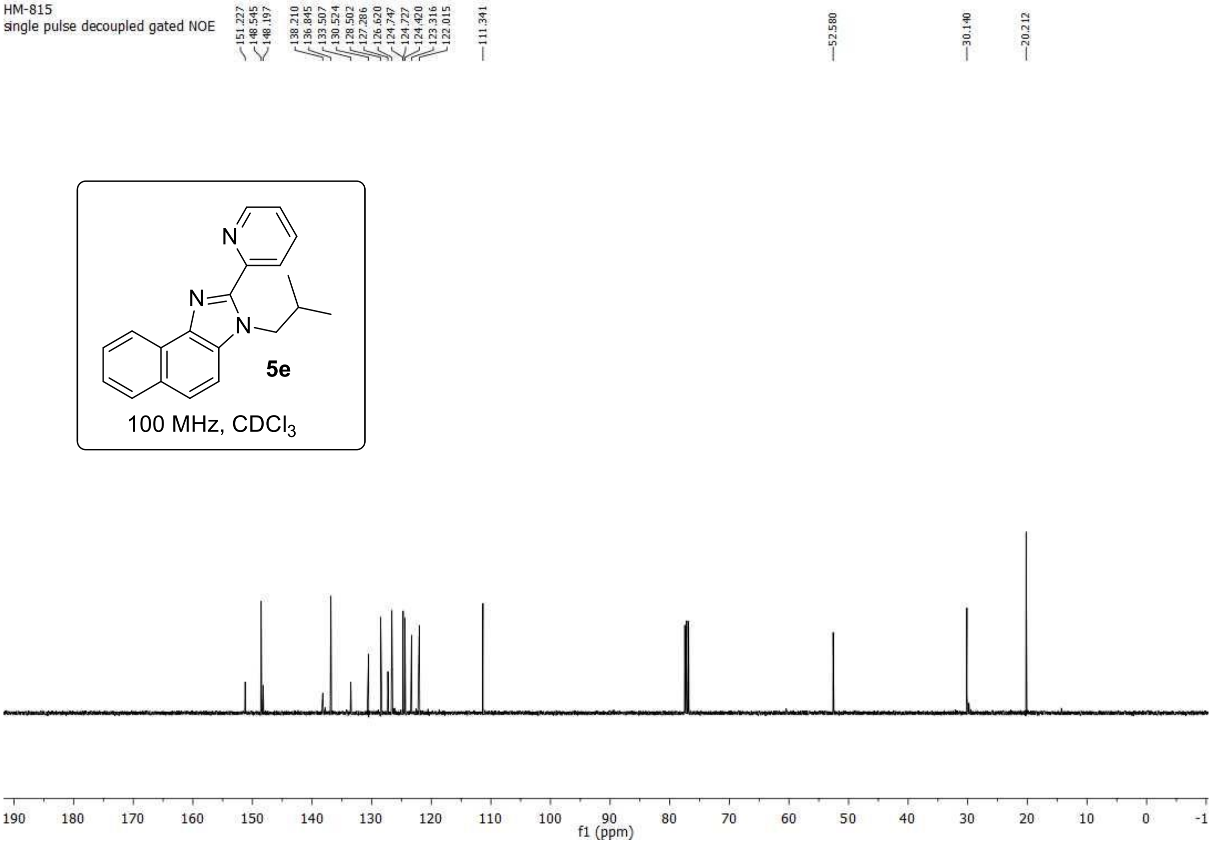
HM-815
single pulse decoupled gated NOE
151.227 148.545 148.197 138.210 136.845 133.507 130.524 128.502 127.286 126.620 124.747 124.727 124.420 123.316 122.015 111.341 52.580 30.140 20.212
N
N
N
5e
100 MHz, CDCl3
f1 (ppm)

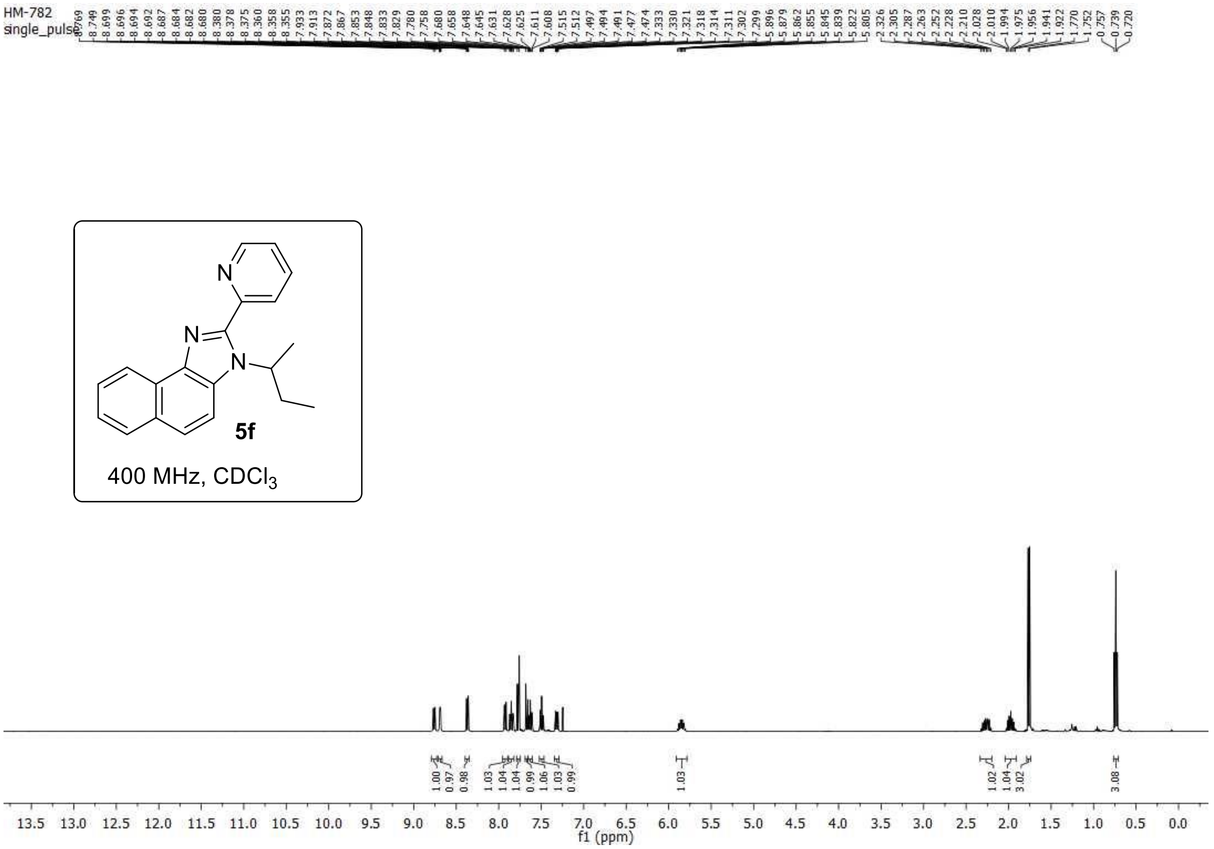
HM-782
single_pulse
5f
400 MHz, CDCl$_3$
f1 (ppm)

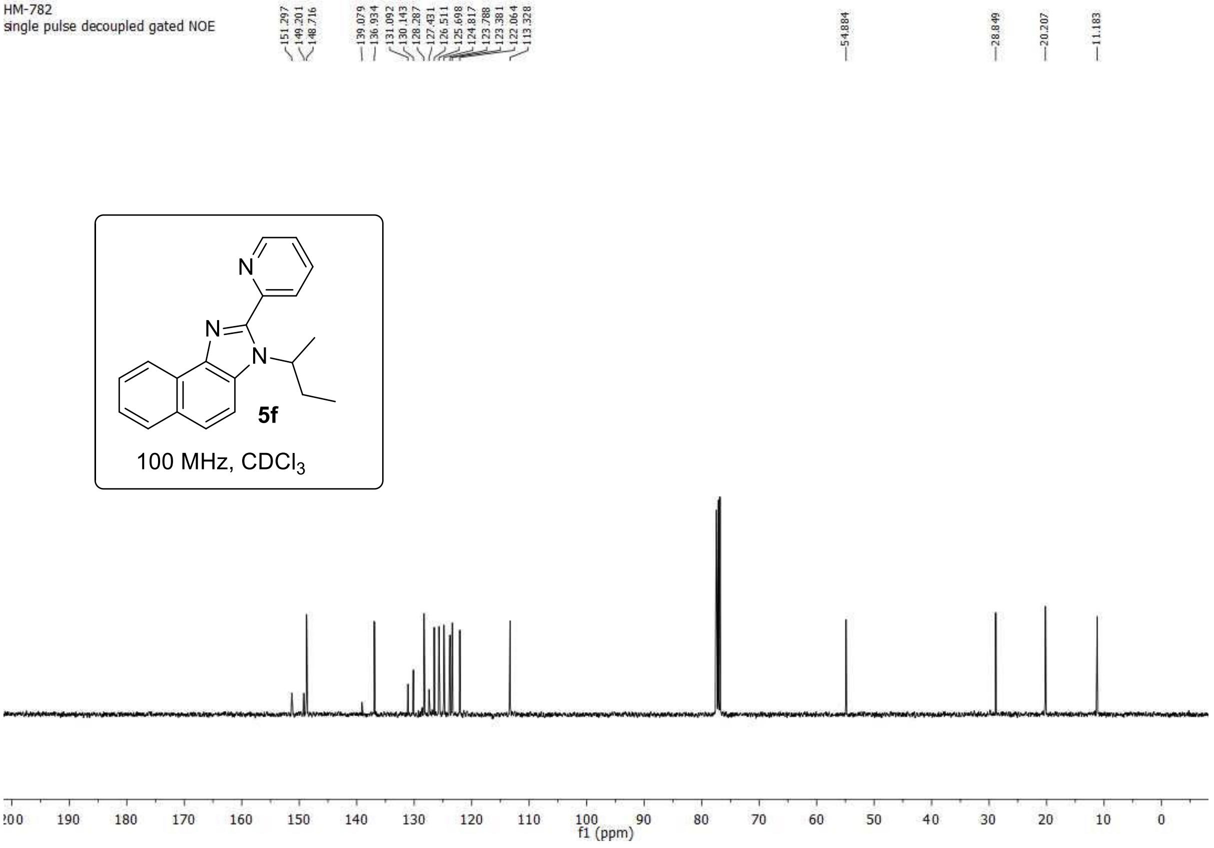
HM-782
single pulse decoupled gated NOE
151.297
149.201
148.716
139.079
136.934
131.092
130.143
128.287
127.431
126.511
125.698
124.817
123.788
123.381
122.064
113.328
54.884
28.849
20.207
11.183
5f
100 MHz, CDCl$_3$
f1 (ppm)

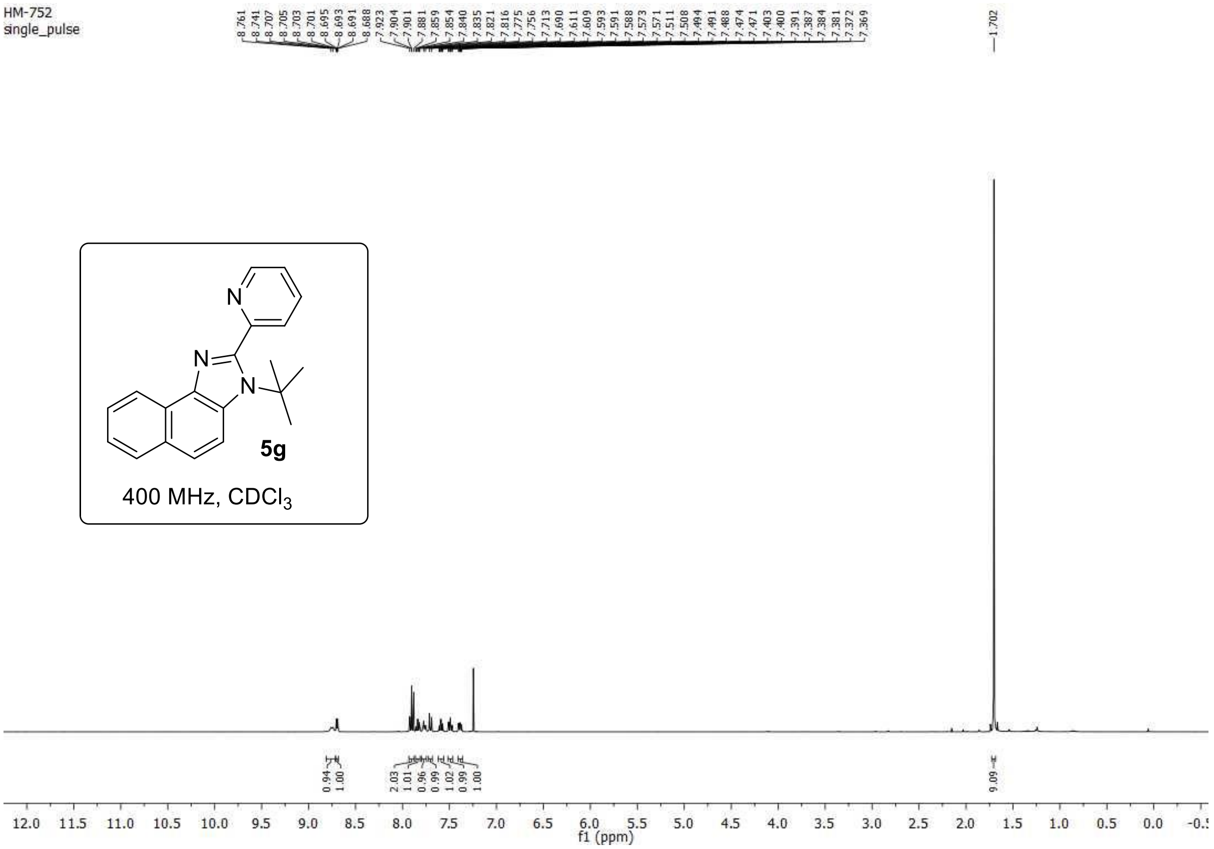

HM-752
single_pulse
8.761
8.741
8.707
8.705
8.703
8.701
8.695
8.693
8.691
8.688
7.923
7.904
7.901
7.881
7.859
7.854
7.840
7.835
7.821
7.816
7.775
7.756
7.713
7.690
7.611
7.609
7.593
7.591
7.588
7.573
7.571
7.511
7.508
7.494
7.491
7.488
7.474
7.471
7.403
7.400
7.391
7.387
7.384
7.381
7.372
7.369
1.702
N
N
N
5g
400 MHz, CDCl3
0.94
1.00
2.03
1.01
0.96
0.99
1.02
0.99
1.00
9.09
f1 (ppm)

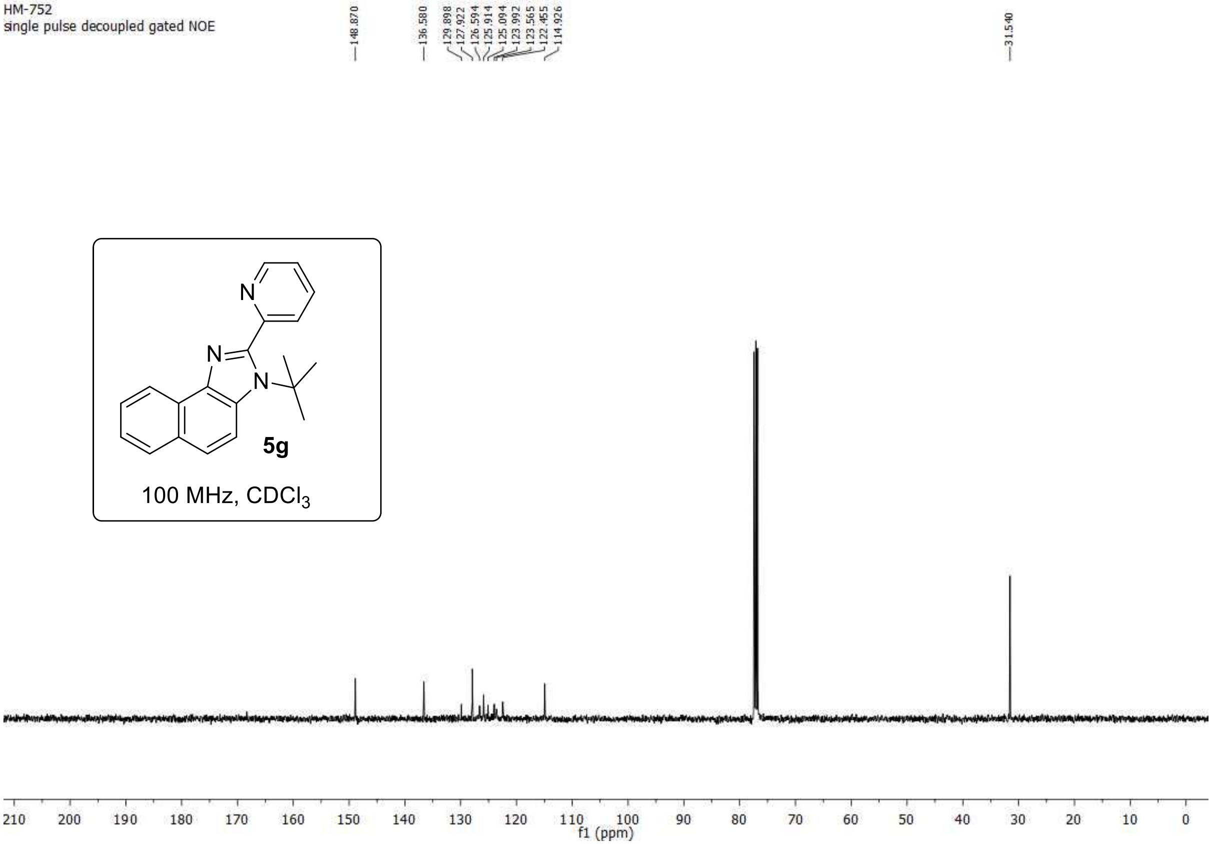

HM-752
single pulse decoupled gated NOE
148.870
136.580
129.898
127.922
126.594
125.914
125.094
123.992
123.565
122.455
114.926
31.540
N
N
N
5g
100 MHz, CDCl3
f1 (ppm)

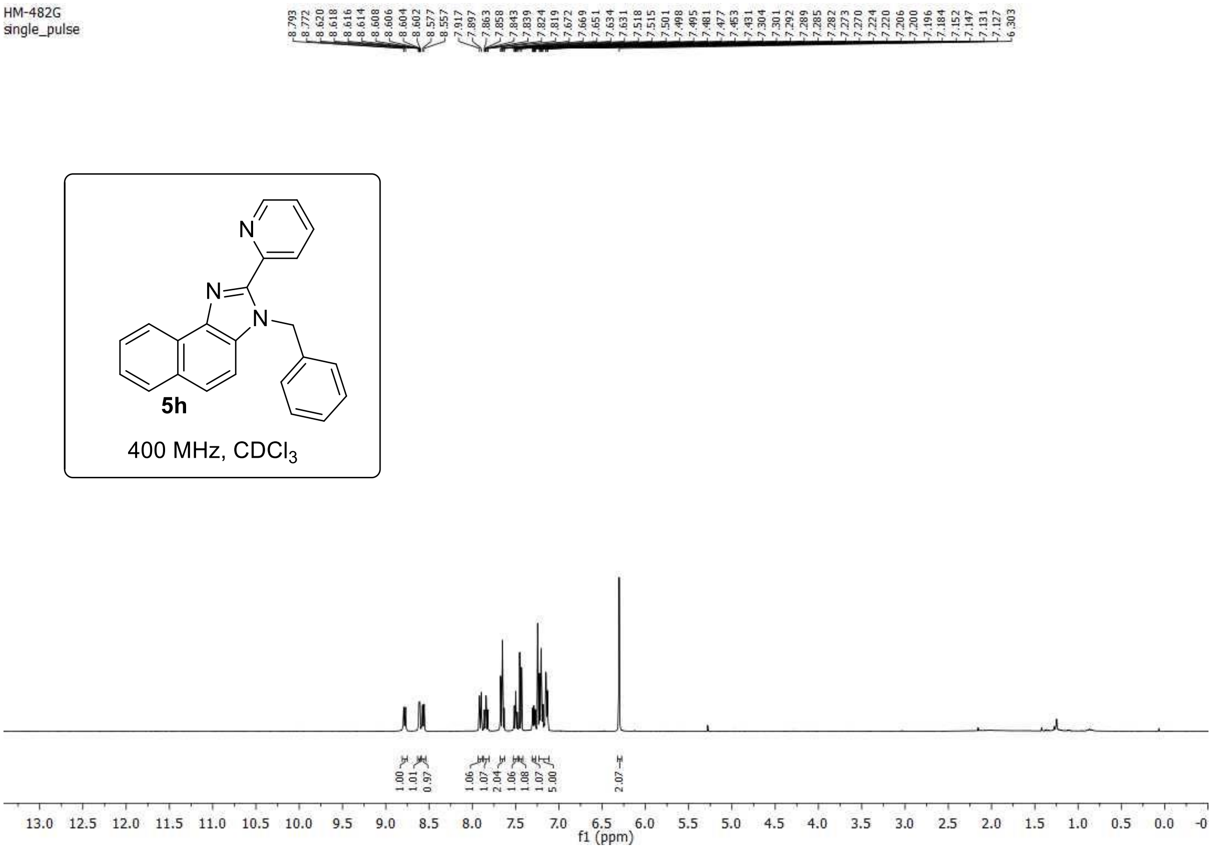
HM-482G
single_pulse
8.793
8.772
8.620
8.618
8.616
8.614
8.608
8.606
8.604
8.602
8.577
8.557
7.917
7.897
7.863
7.858
7.843
7.839
7.824
7.819
7.672
7.669
7.651
7.634
7.631
7.518
7.515
7.501
7.498
7.495
7.481
7.477
7.453
7.431
7.304
7.301
7.292
7.289
7.285
7.282
7.273
7.270
7.224
7.220
7.206
7.200
7.196
7.184
7.152
7.147
7.131
7.127
6.303
N
N
N
5h
400 MHz, $CDCl_3$
1.00
1.01
0.97
1.06
1.07
2.04
1.06
1.08
1.07
5.00
2.07
13.0 12.5 12.0 11.5 11.0 10.5 10.0 9.5 9.0 8.5 8.0 7.5 7.0 6.5 6.0 5.5 5.0 4.5 4.0 3.5 3.0 2.5 2.0 1.5 1.0 0.5 0.0
f1 (ppm)

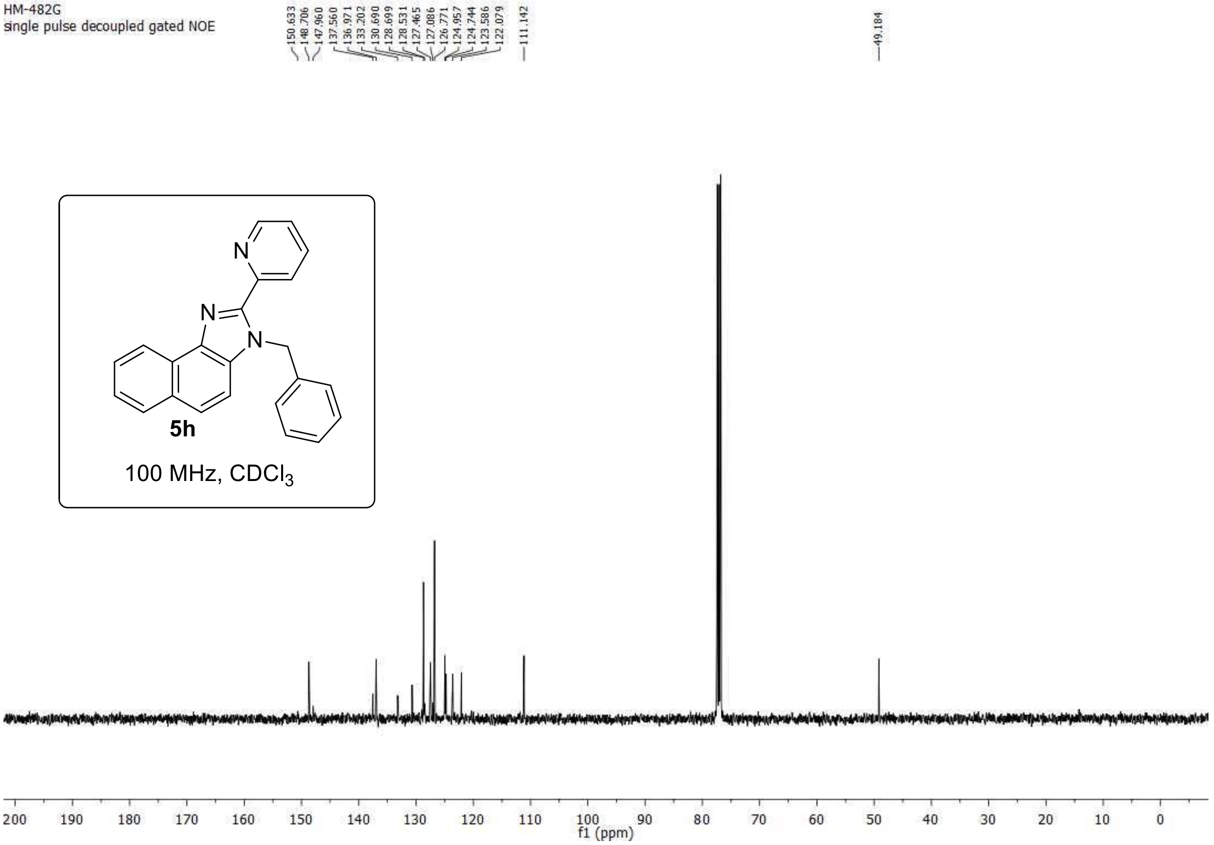
HM-482G
single pulse decoupled gated NOE
150.633
148.706
147.960
137.560
136.971
133.202
130.690
128.699
128.531
127.465
127.086
126.771
124.957
124.744
123.586
122.079
111.142
49.184
N
N
N
5h
100 MHz, $CDCl_3$
200 190 180 170 160 150 140 130 120 110 100 90 80 70 60 50 40 30 20 10 0
f1 (ppm)

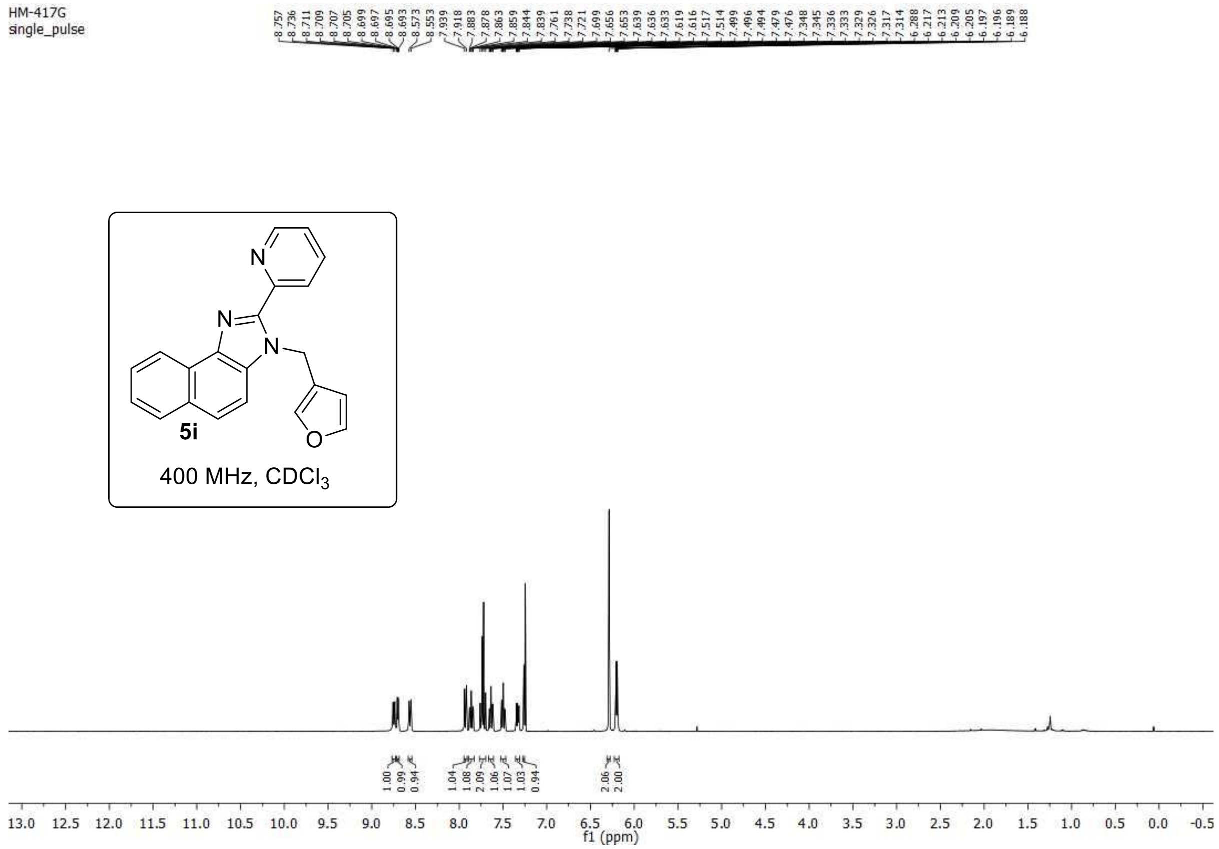
HM-417G
single_pulse
8.757 8.736 8.711 8.709 8.707 8.705 8.699 8.697 8.695 8.693 8.573 8.553 7.939 7.918 7.883 7.878 7.863 7.859 7.844 7.839 7.761 7.738 7.721 7.699 7.656 7.653 7.639 7.636 7.633 7.619 7.616 7.517 7.514 7.499 7.496 7.494 7.479 7.476 7.348 7.345 7.336 7.333 7.329 7.326 7.317 7.314 6.288 6.217 6.213 6.209 6.205 6.197 6.196 6.189 6.188
N
N
N
O
5i
400 MHz, CDCl$_3$
1.00 0.99 0.94 1.04 1.08 2.09 1.06 1.07 1.03 0.94 2.06 2.00
f1 (ppm)

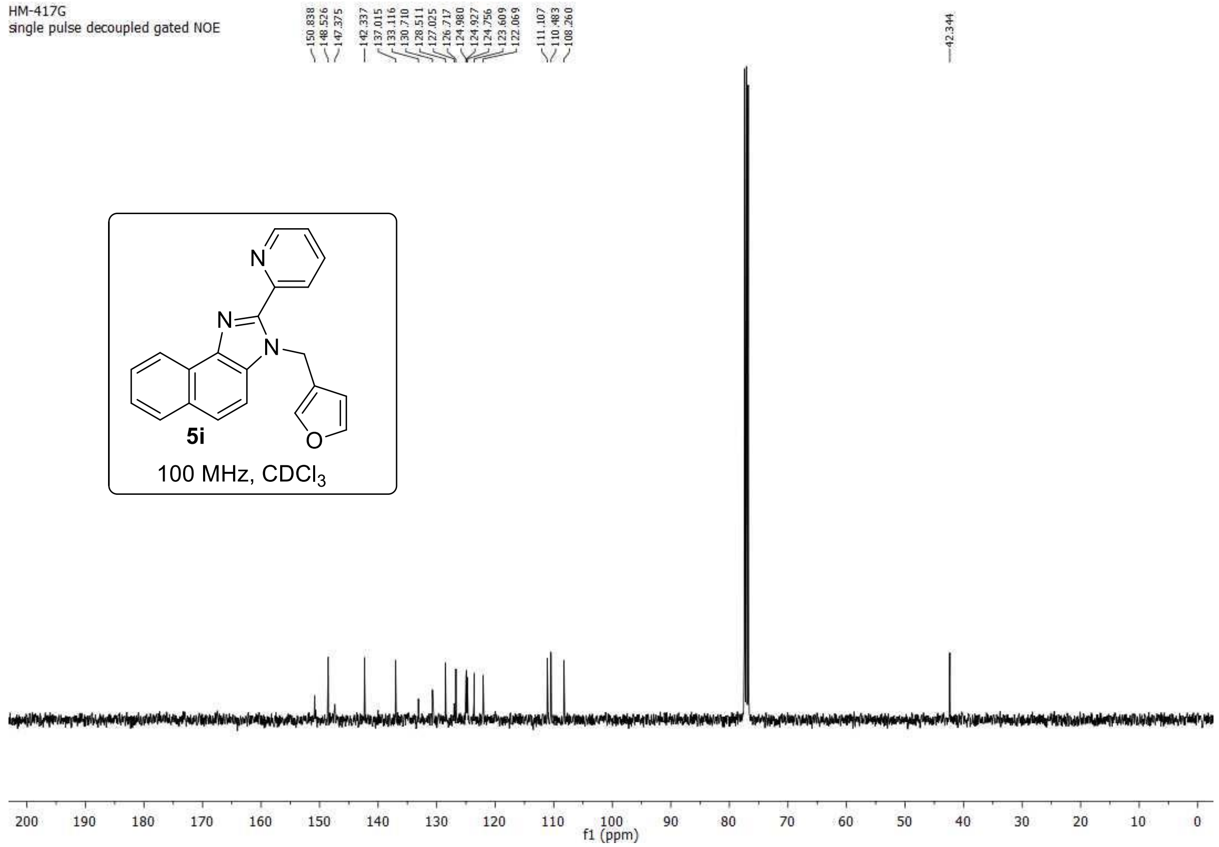
HM-417G
single pulse decoupled gated NOE
150.838 148.526 147.375 142.337 137.015 133.116 130.710 128.511 127.025 126.717 124.980 124.927 124.756 123.609 122.069 111.107 110.483 108.260 42.344
N
N
N
O
5i
100 MHz, CDCl$_3$
f1 (ppm)

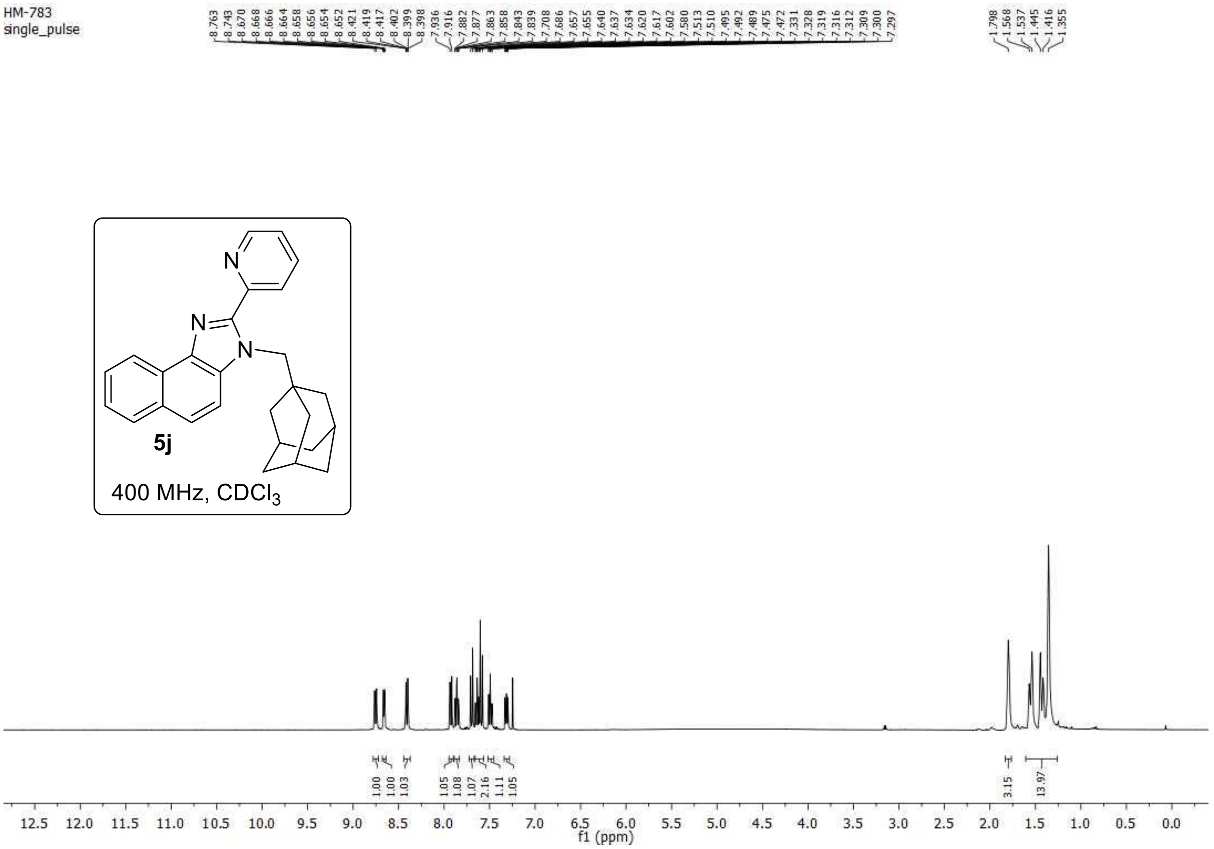
HM-783
single_pulse
8.763 8.743 8.670 8.668 8.666 8.664 8.658 8.656 8.654 8.652 8.421 8.419 8.417 8.402 8.399 8.398 7.936 7.916 7.882 7.877 7.863 7.858 7.843 7.839 7.708 7.686 7.657 7.655 7.640 7.637 7.634 7.620 7.617 7.602 7.580 7.513 7.510 7.495 7.492 7.489 7.475 7.472 7.331 7.328 7.319 7.316 7.312 7.309 7.300 7.297
1.798 1.568 1.537 1.445 1.416 1.355
N
N
N
5j
400 MHz, $CDCl_3$
1.00 1.00 1.03 1.05 1.08 1.07 2.16 1.11 1.05 3.15 13.97
f1 (ppm)

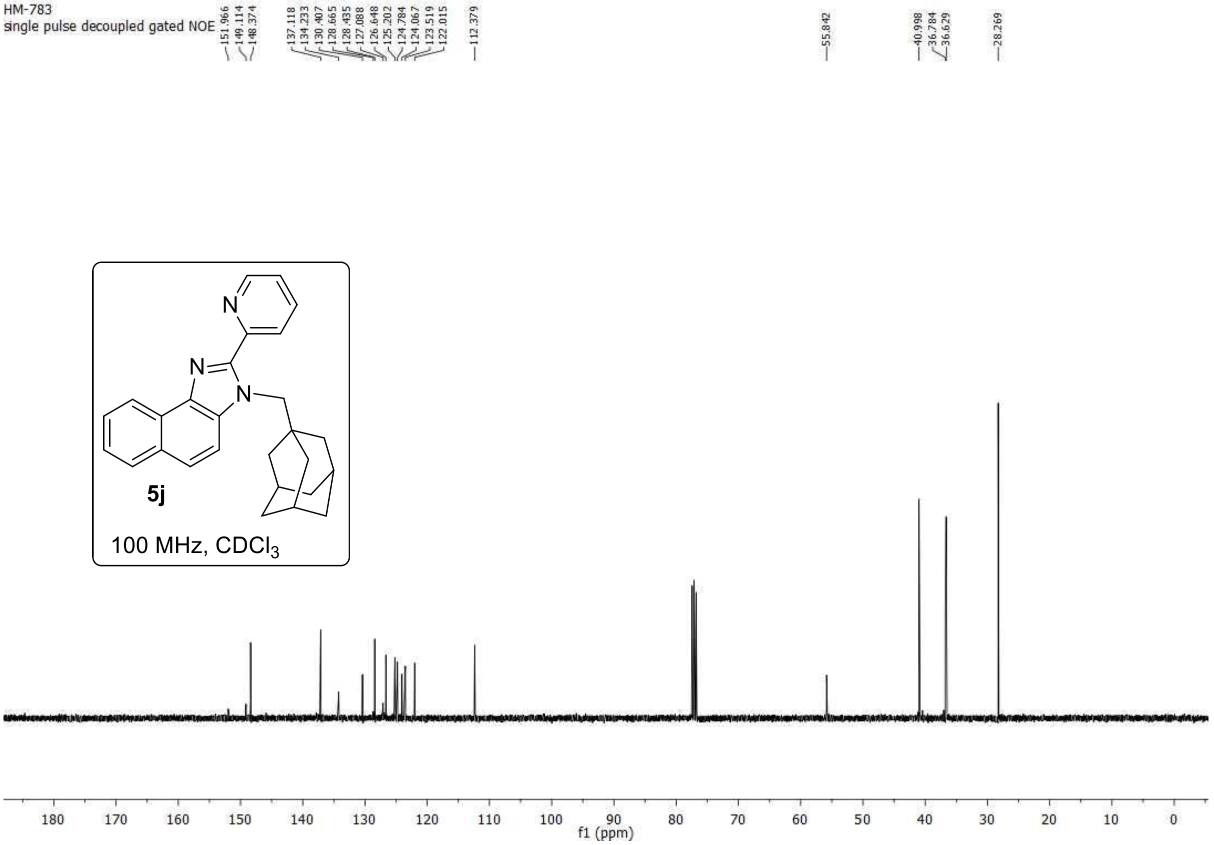
HM-783
single pulse decoupled gated NOE
151.966 149.114 148.374 137.118 134.233 130.407 128.665 128.435 127.088 126.648 125.202 124.784 124.067 123.519 122.015 112.379
55.842 40.998 36.784 36.629 28.269
N
N
N
5j
100 MHz, $CDCl_3$
f1 (ppm)

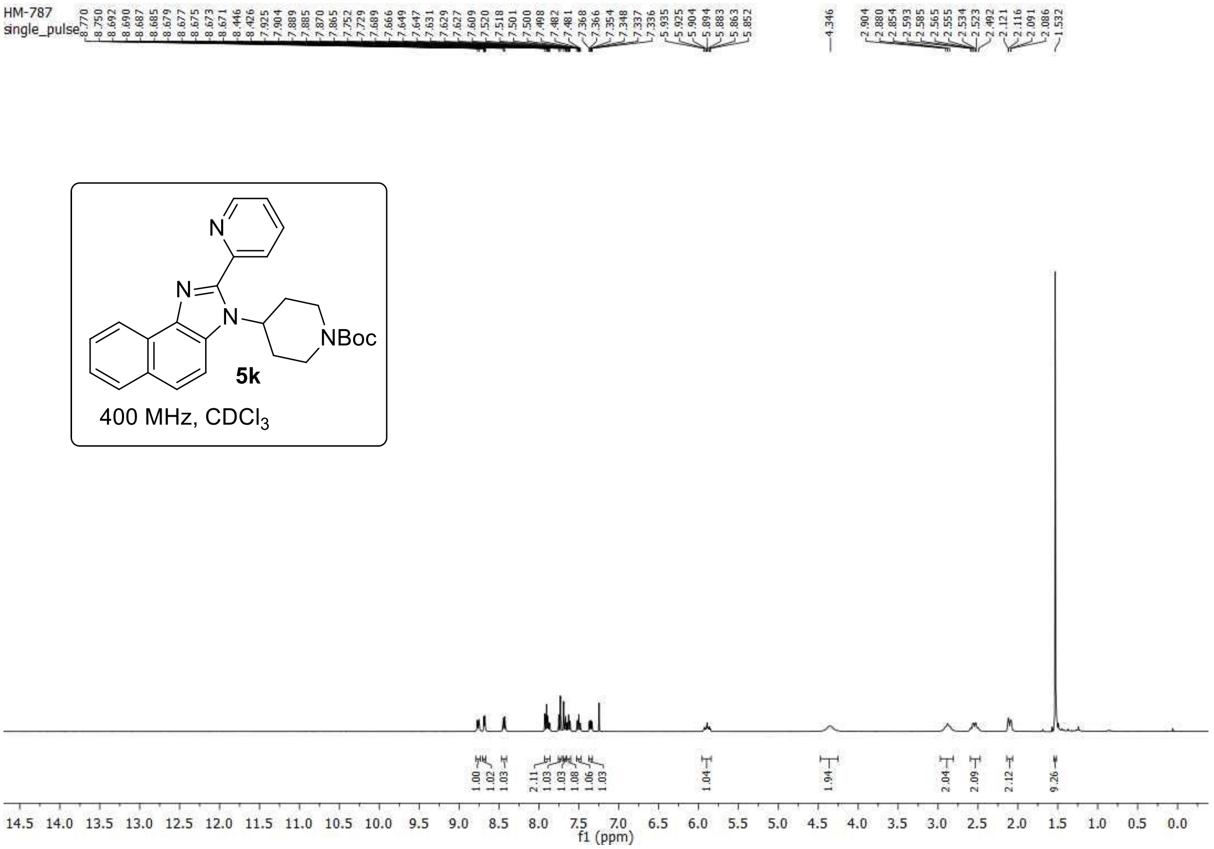
HM-787
single_pulse
8.770 8.750 8.692 8.690 8.687 8.685 8.679 8.677 8.675 8.673 8.671 8.446 8.426 7.925 7.904 7.889 7.885 7.870 7.865 7.752 7.729 7.689 7.666 7.649 7.647 7.631 7.629 7.627 7.609 7.520 7.518 7.501 7.500 7.498 7.482 7.481 7.368 7.366 7.354 7.348 7.337 7.336 5.935 5.925 5.904 5.894 5.883 5.863 5.852
4.346
2.904 2.880 2.854 2.593 2.585 2.565 2.555 2.534 2.523 2.492 2.121 2.116 2.091 2.086 1.532
N
N
N
NBoc
5k
400 MHz, CDCl$_3$
1.00 1.02 1.03 2.11 1.03 1.03 1.08 1.06 1.03 1.04 1.94 2.04 2.09 2.12 9.26
14.5 14.0 13.5 13.0 12.5 12.0 11.5 11.0 10.5 10.0 9.5 9.0 8.5 8.0 7.5 7.0 6.5 6.0 5.5 5.0 4.5 4.0 3.5 3.0 2.5 2.0 1.5 1.0 0.5 0.0
f1 (ppm)

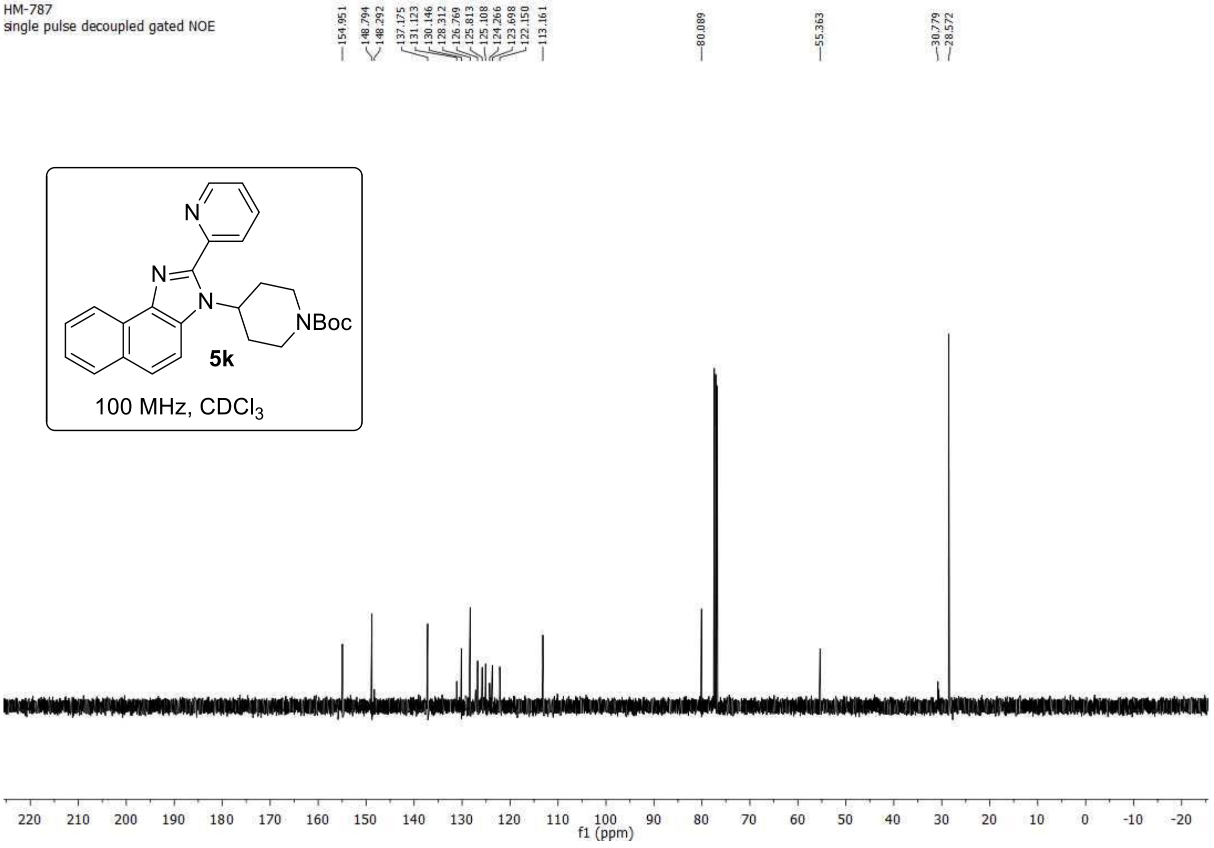
HM-787
single pulse decoupled gated NOE
154.951 148.794 148.292 137.175 131.123 130.146 128.312 126.769 125.813 125.108 124.266 123.698 122.150 113.161
80.089
55.363
30.779 28.572
N
N
N
NBoc
5k
100 MHz, CDCl$_3$
220 210 200 190 180 170 160 150 140 130 120 110 100 90 80 70 60 50 40 30 20 10 0 -10 -20
f1 (ppm)

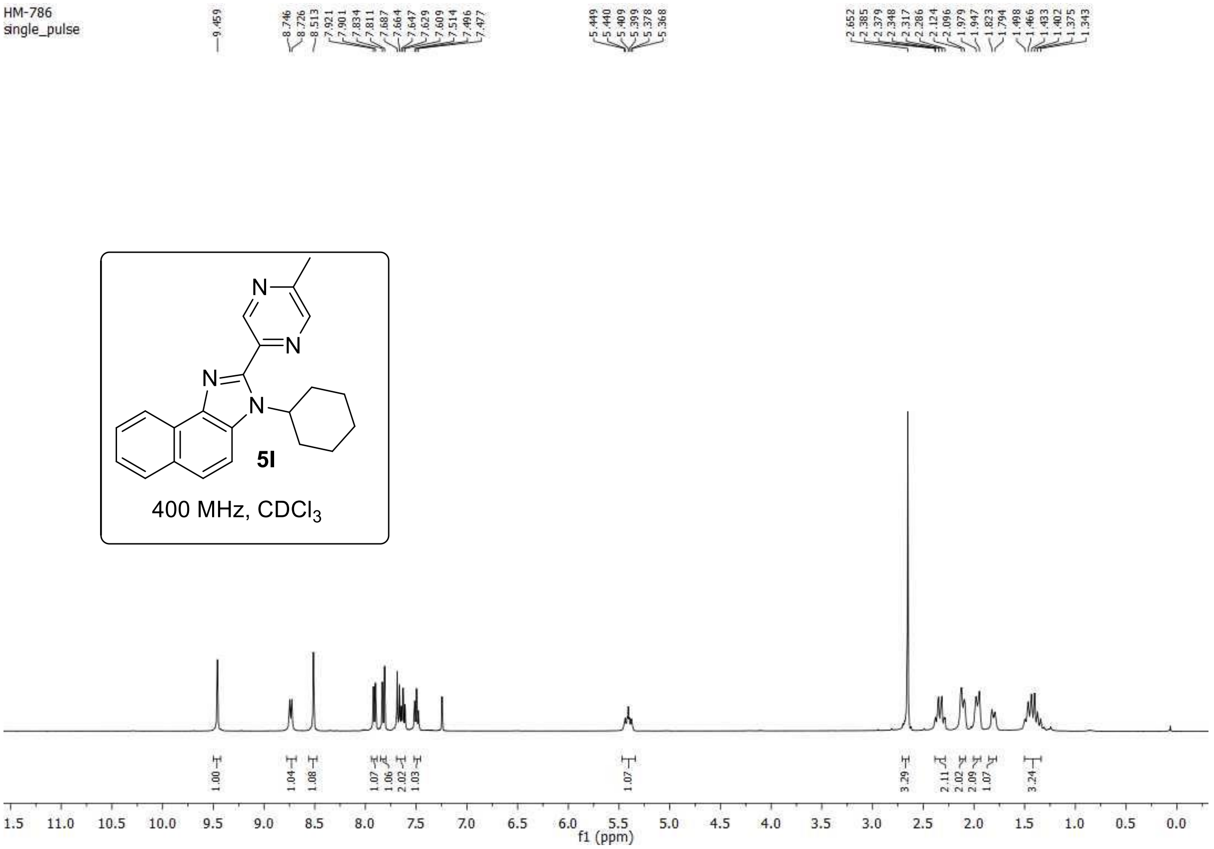
HM-786
single_pulse
9.459
8.746 8.726 8.513 7.921 7.901 7.834 7.811 7.687 7.664 7.647 7.629 7.609 7.514 7.496 7.477
5.449 5.440 5.409 5.399 5.378 5.368
2.652 2.385 2.379 2.348 2.317 2.286 2.124 2.096 1.979 1.947 1.823 1.794 1.498 1.466 1.433 1.402 1.375 1.343
5l
400 MHz, CDCl3
1.00 1.04 1.08 1.07 1.06 2.02 1.03 1.07 3.29 2.11 2.02 2.09 1.07 3.24
f1 (ppm)

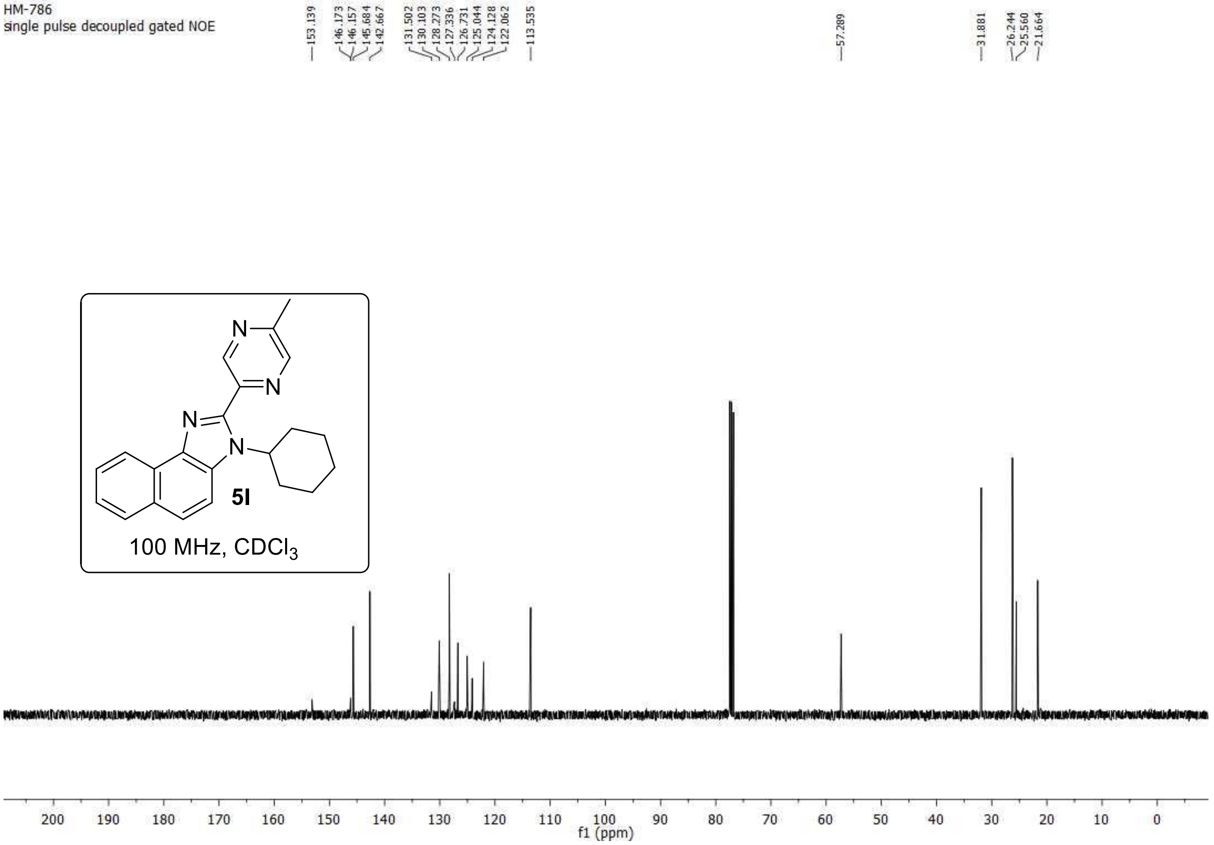
HM-786
single pulse decoupled gated NOE
153.139 146.173 146.157 145.684 142.667 131.502 130.103 128.273 127.336 126.731 125.044 124.128 122.062 113.535
57.289
31.881 26.244 25.560 21.664
5l
100 MHz, CDCl3
f1 (ppm)

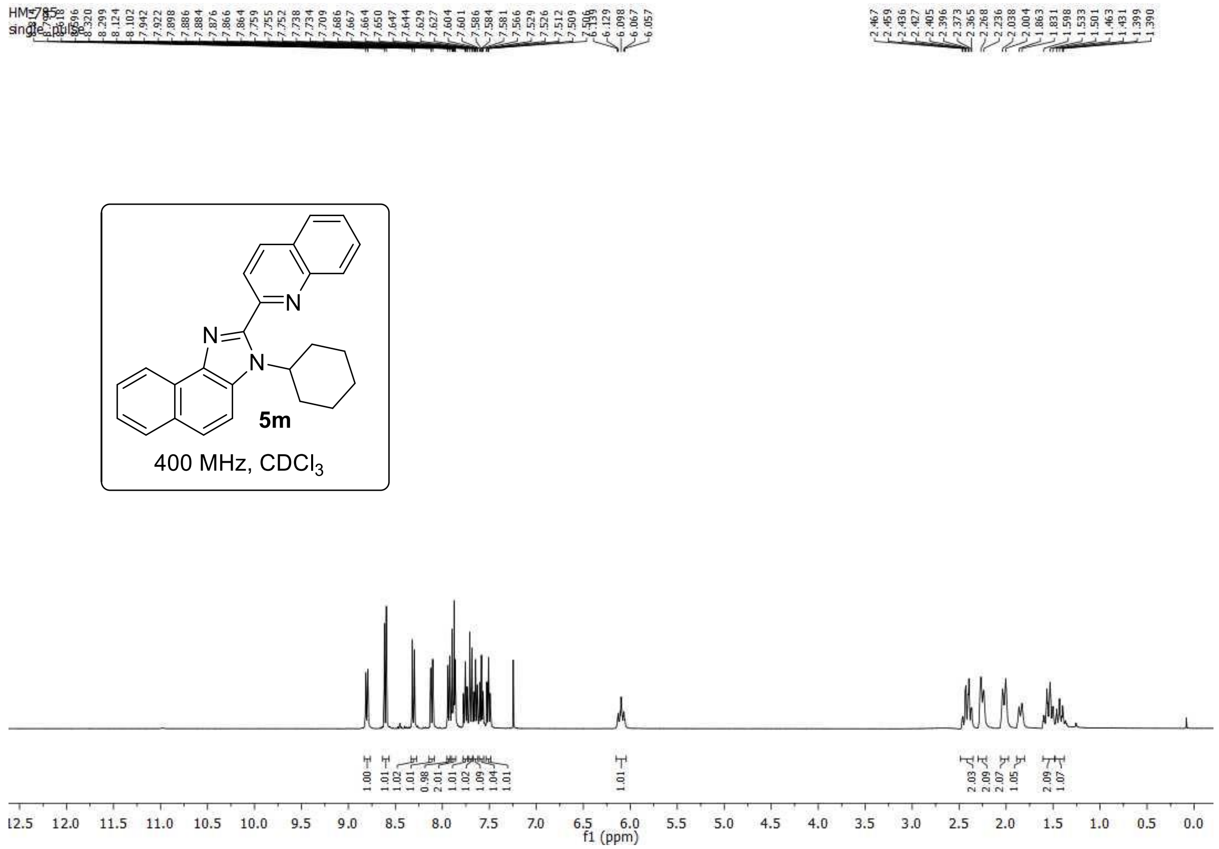
HM-785
single_pulse
N
N
N
5m
400 MHz, CDCl3
2.467
2.459
2.436
2.427
2.405
2.396
2.373
2.365
2.268
2.236
2.038
2.004
1.863
1.831
1.598
1.533
1.501
1.463
1.431
1.399
1.390
f1 (ppm)

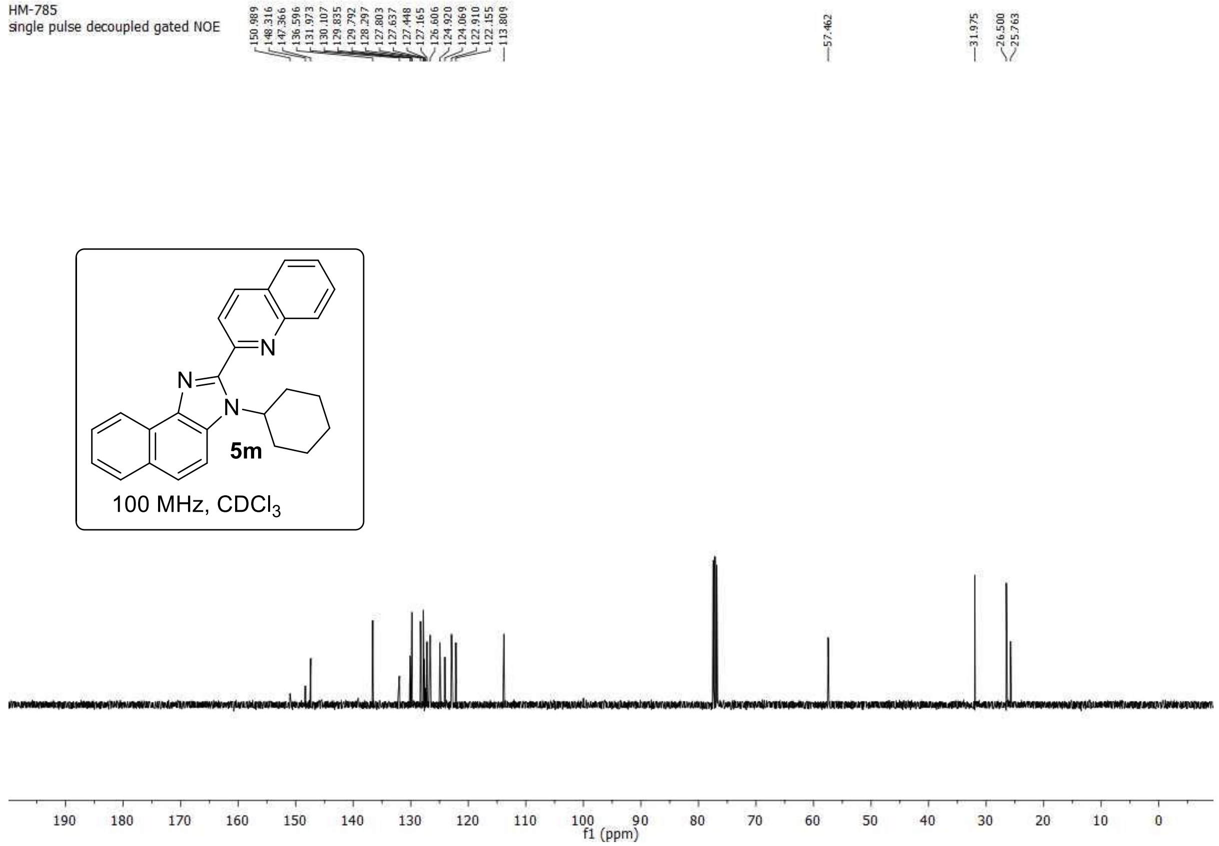
HM-785
single pulse decoupled gated NOE
150.989
148.316
147.366
136.596
131.973
130.107
129.835
129.792
128.297
127.803
127.637
127.448
127.165
126.606
124.920
124.069
122.910
122.155
113.809
57.462
31.975
26.500
25.763
N
N
N
5m
100 MHz, CDCl3
f1 (ppm)

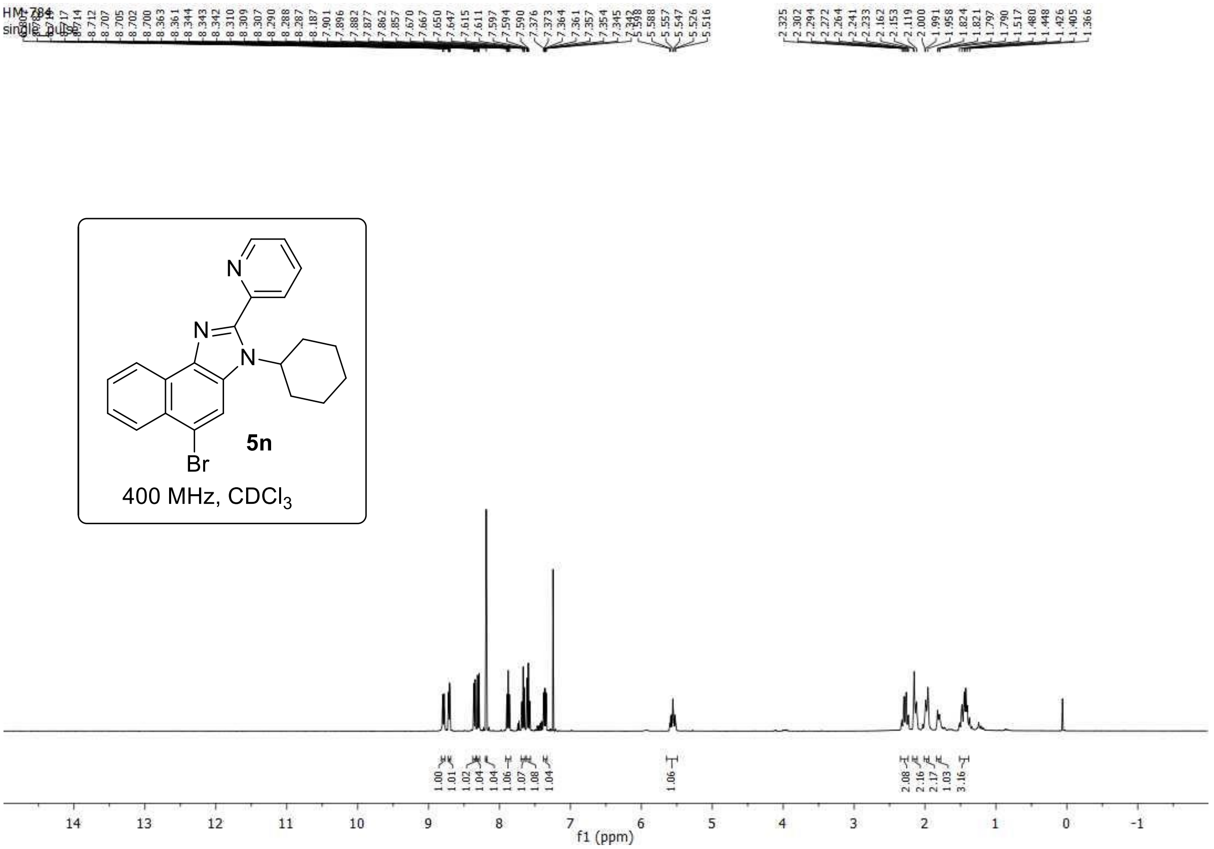
HM-784
single pulse
8.712
8.707
8.705
8.702
8.700
8.363
8.361
8.344
8.343
8.342
8.310
8.309
8.307
8.290
8.288
8.287
8.187
7.901
7.896
7.882
7.877
7.862
7.857
7.670
7.667
7.650
7.647
7.615
7.611
7.597
7.594
7.590
7.376
7.373
7.364
7.361
7.357
7.354
7.345
5.588
5.557
5.547
5.526
5.516
2.325
2.302
2.294
2.272
2.264
2.241
2.233
2.162
2.153
2.119
2.000
1.991
1.958
1.824
1.821
1.797
1.790
1.517
1.480
1.448
1.426
1.405
1.366
N
N
N
5n
Br
400 MHz, CDCl3
1.00
1.01
1.02
1.04
1.04
1.06
1.07
1.08
1.04
1.06
2.08
2.16
2.17
1.03
3.16
14
13
12
11
10
9
8
7
6
5
4
3
2
1
0
-1
f1 (ppm)

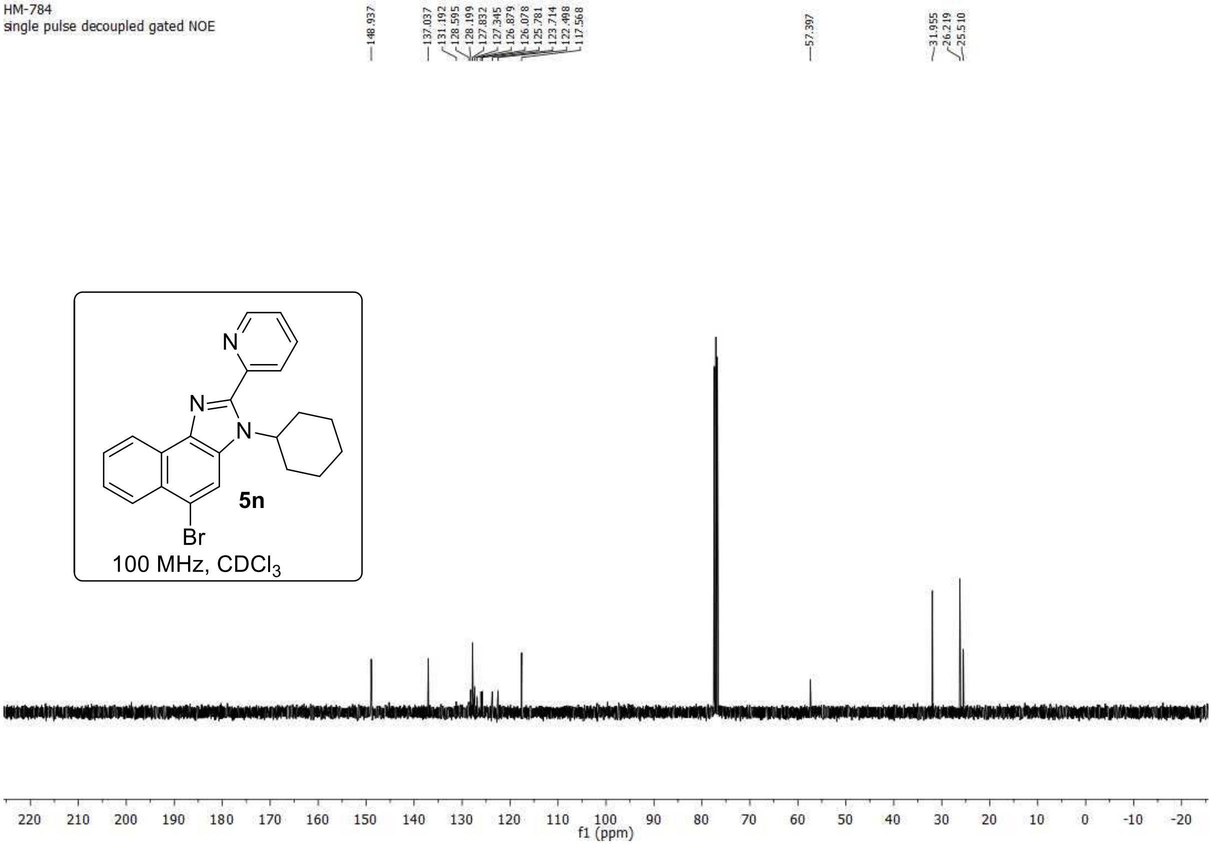
HM-784
single pulse decoupled gated NOE
148.937
137.037
131.192
128.595
128.199
127.832
127.345
126.879
126.078
125.781
123.714
122.498
117.568
57.397
31.955
26.219
25.510
N
N
N
5n
Br
100 MHz, CDCl3
220
210
200
190
180
170
160
150
140
130
120
110
100
90
80
70
60
50
40
30
20
10
0
-10
-20
f1 (ppm)

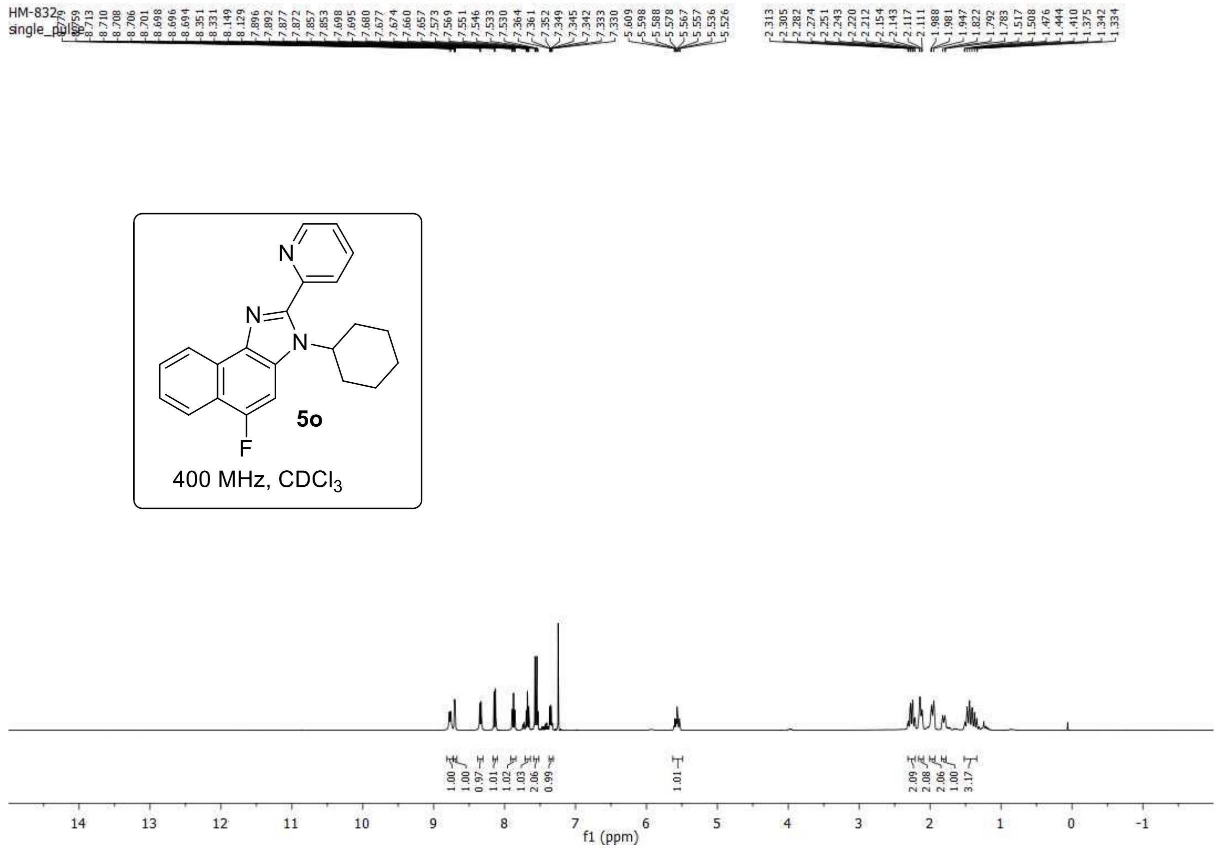
HM-832
single_pulse
8.779 8.759 8.713 8.710 8.708 8.706 8.701 8.698 8.696 8.694 8.351 8.331 8.149 8.129 7.896 7.892 7.877 7.872 7.857 7.853 7.698 7.695 7.680 7.677 7.674 7.660 7.657 7.573 7.569 7.551 7.546 7.533 7.530 7.364 7.361 7.352 7.349 7.345 7.342 7.333 7.330 5.609 5.598 5.588 5.578 5.567 5.557 5.536 5.526
2.313 2.305 2.282 2.274 2.251 2.243 2.220 2.212 2.154 2.143 2.117 2.111 1.988 1.981 1.947 1.822 1.792 1.783 1.517 1.508 1.476 1.444 1.410 1.375 1.342 1.334
N
N
N
5o
F
400 MHz, CDCl$_3$
1.00 1.00 0.97 1.01 1.02 1.03 2.06 0.99
1.01
2.09 2.08 2.06 1.00 3.17
14 13 12 11 10 9 8 7 6 5 4 3 2 1 0 -1
f1 (ppm)

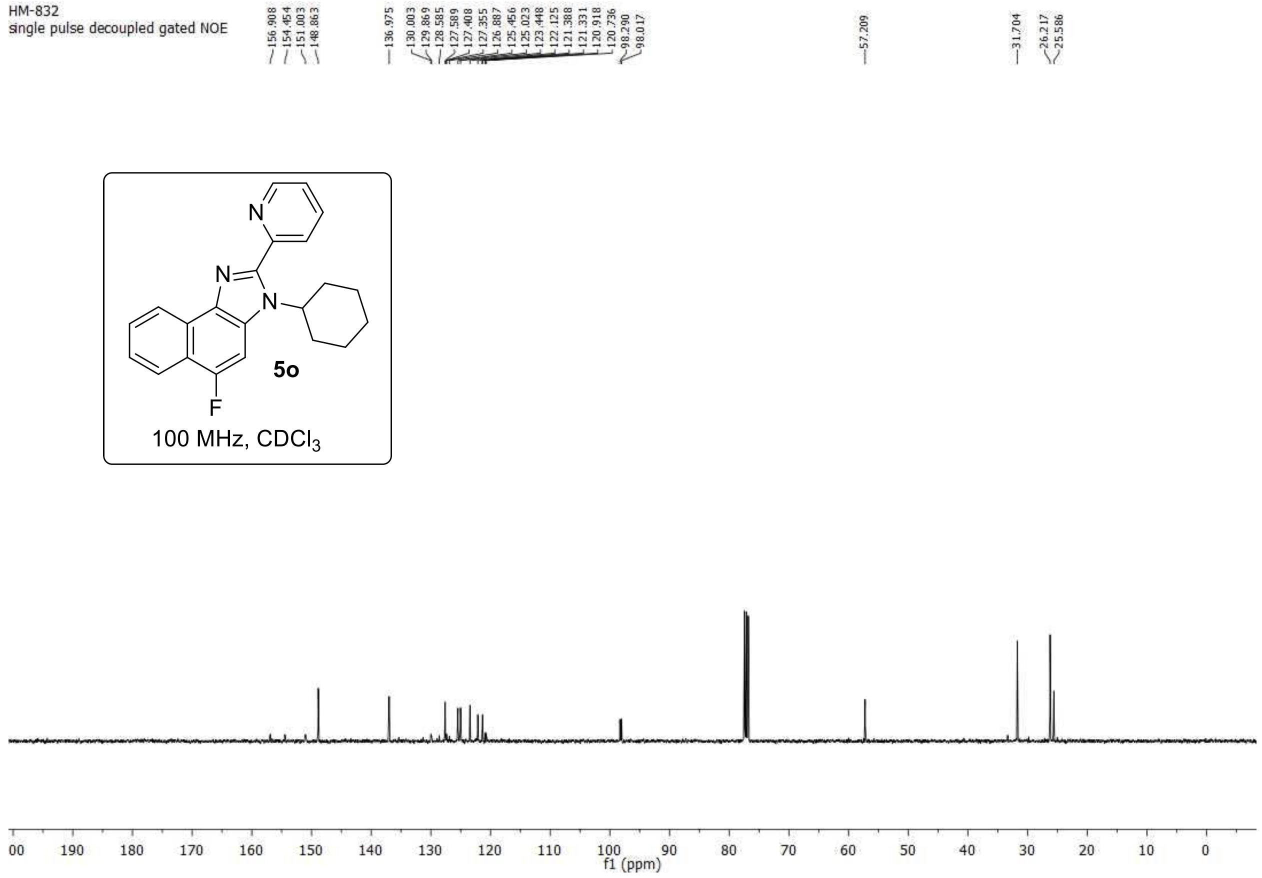
HM-832
single pulse decoupled gated NOE
156.908 154.454 151.003 148.863
136.975
130.003 129.869 128.585 127.589 127.408 127.355 126.887 125.456 125.023 123.448 122.125 121.388 121.331 120.918 120.736 98.290 98.017
57.209
31.704
26.217 25.586
N
N
N
5o
F
100 MHz, CDCl$_3$
00 190 180 170 160 150 140 130 120 110 100 90 80 70 60 50 40 30 20 10 0
f1 (ppm)

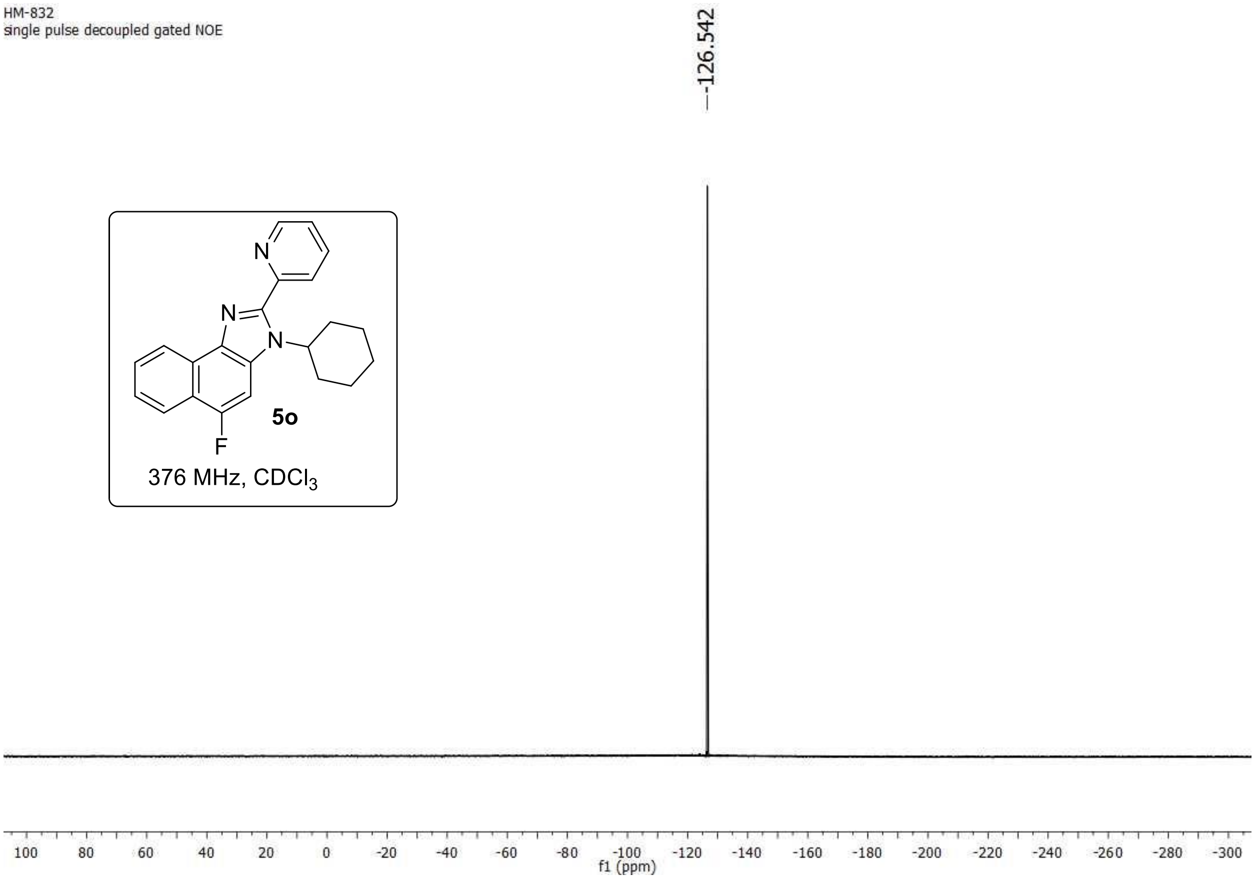
HM-832
single pulse decoupled gated NOE
-126.542
N
N
N
F
5o
376 MHz, CDCl$_3$
100 80 60 40 20 0 -20 -40 -60 -80 -100 -120 -140 -160 -180 -200 -220 -240 -260 -280 -300
f1 (ppm)

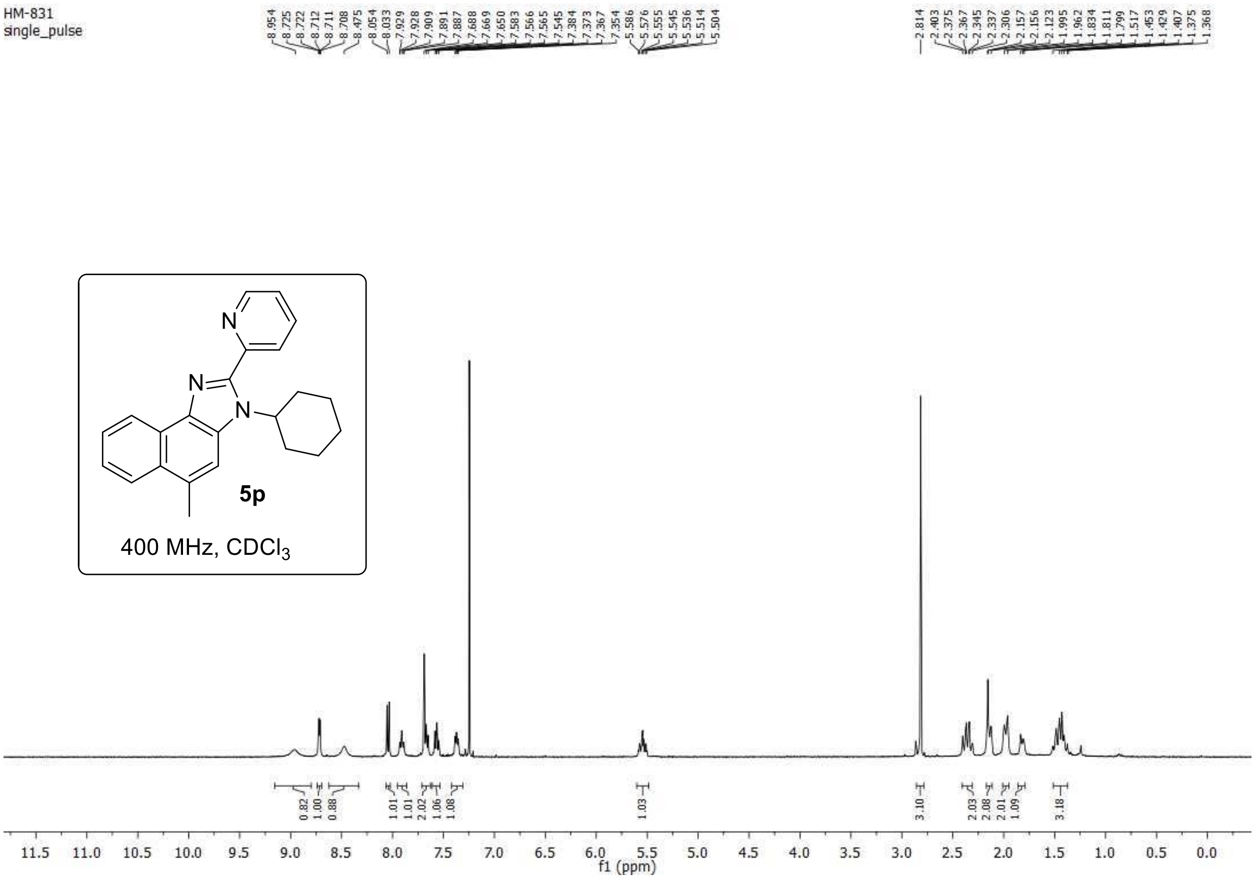
HM-831
single_pulse
8.954
8.725
8.722
8.712
8.711
8.708
8.475
8.054
8.033
7.929
7.928
7.909
7.891
7.887
7.688
7.669
7.650
7.583
7.566
7.565
7.545
7.384
7.373
7.367
7.354
5.586
5.576
5.555
5.545
5.536
5.514
5.504
2.814
2.403
2.375
2.367
2.345
2.337
2.306
2.157
2.156
2.123
1.995
1.962
1.834
1.811
1.799
1.517
1.453
1.429
1.407
1.375
1.368
N
N
N
5p
400 MHz, $CDCl_3$
0.82
1.00
0.88
1.01
1.01
2.02
1.06
1.08
1.03
3.10
2.03
2.08
2.01
1.09
3.18
f1 (ppm)

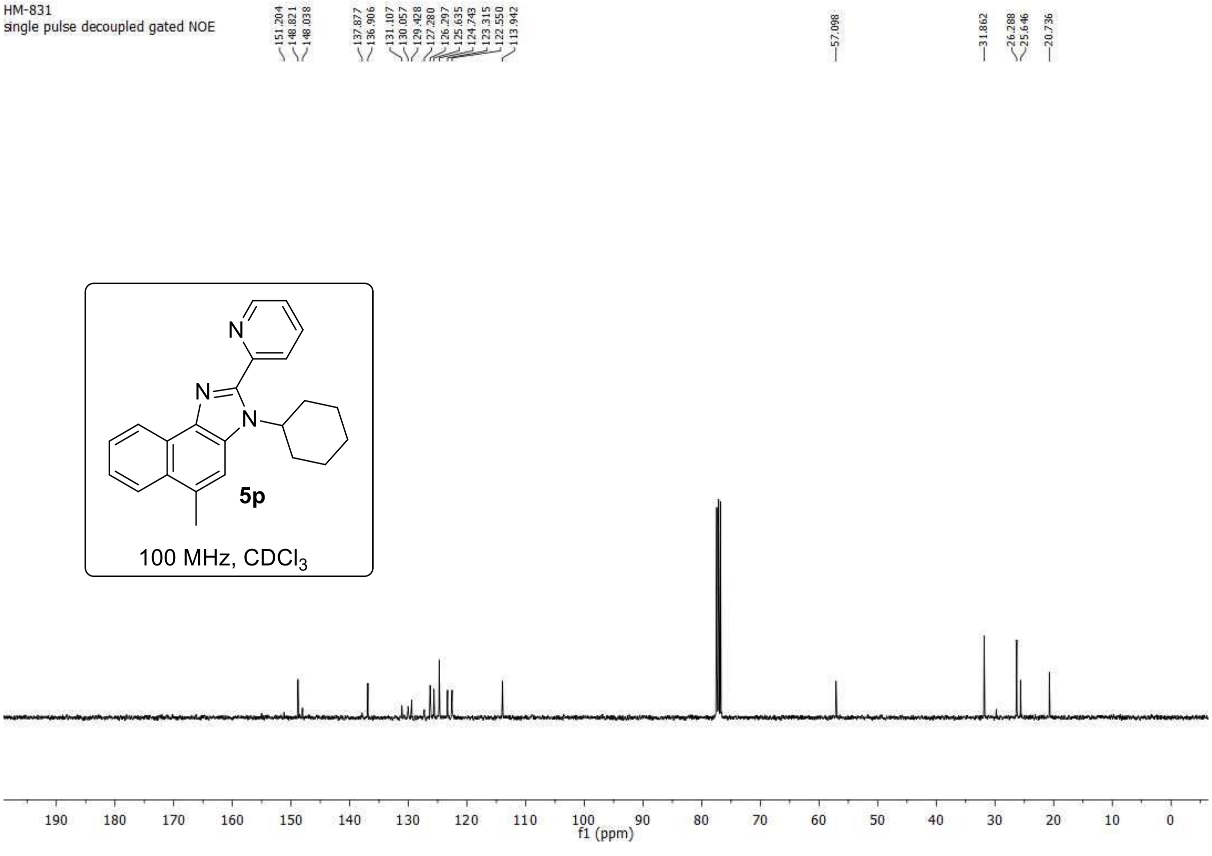
HM-831
single pulse decoupled gated NOE
151.204
148.821
148.038
137.877
136.906
131.107
130.057
129.428
127.280
126.297
125.635
124.743
123.315
122.550
113.942
57.098
31.862
26.288
25.646
20.736
N
N
N
5p
100 MHz, $CDCl_3$
f1 (ppm)

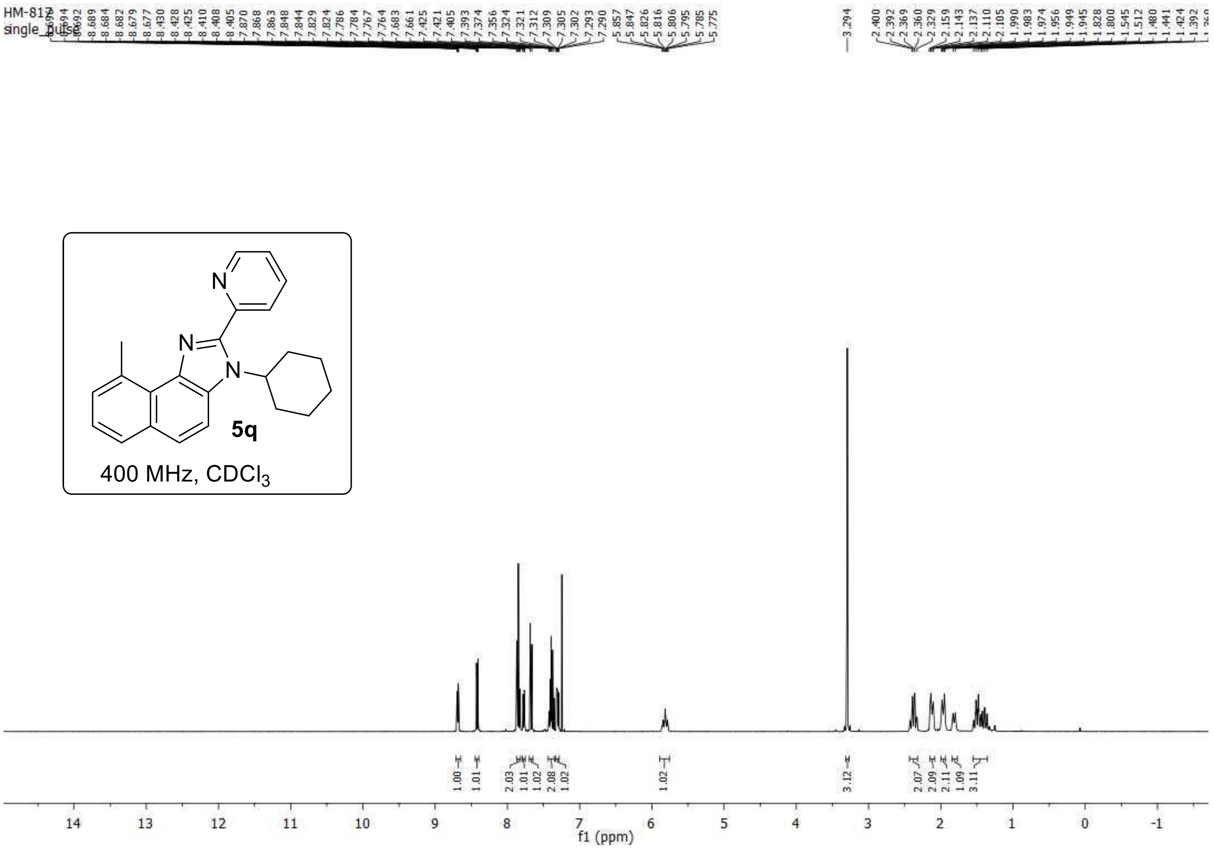
HM-817
single_pulse
8.694
8.692
8.689
8.684
8.682
8.679
8.677
8.430
8.428
8.425
8.410
8.408
8.405
7.870
7.868
7.863
7.848
7.844
7.829
7.824
7.786
7.784
7.767
7.764
7.683
7.661
7.425
7.421
7.405
7.393
7.374
7.356
7.324
7.321
7.312
7.309
7.305
7.302
7.293
7.290
5.857
5.847
5.826
5.816
5.806
5.795
5.785
5.775
3.294
2.400
2.392
2.369
2.360
2.329
2.159
2.143
2.137
2.110
2.105
1.990
1.983
1.974
1.956
1.949
1.945
1.828
1.800
1.545
1.512
1.480
1.441
1.424
1.392
N
N
N
5q
400 MHz, CDCl3
1.00
1.01
2.03
1.01
1.02
2.08
1.02
1.02
3.12
2.07
2.09
2.11
1.09
3.11
14
13
12
11
10
9
8
7
6
5
4
3
2
1
0
-1
f1 (ppm)

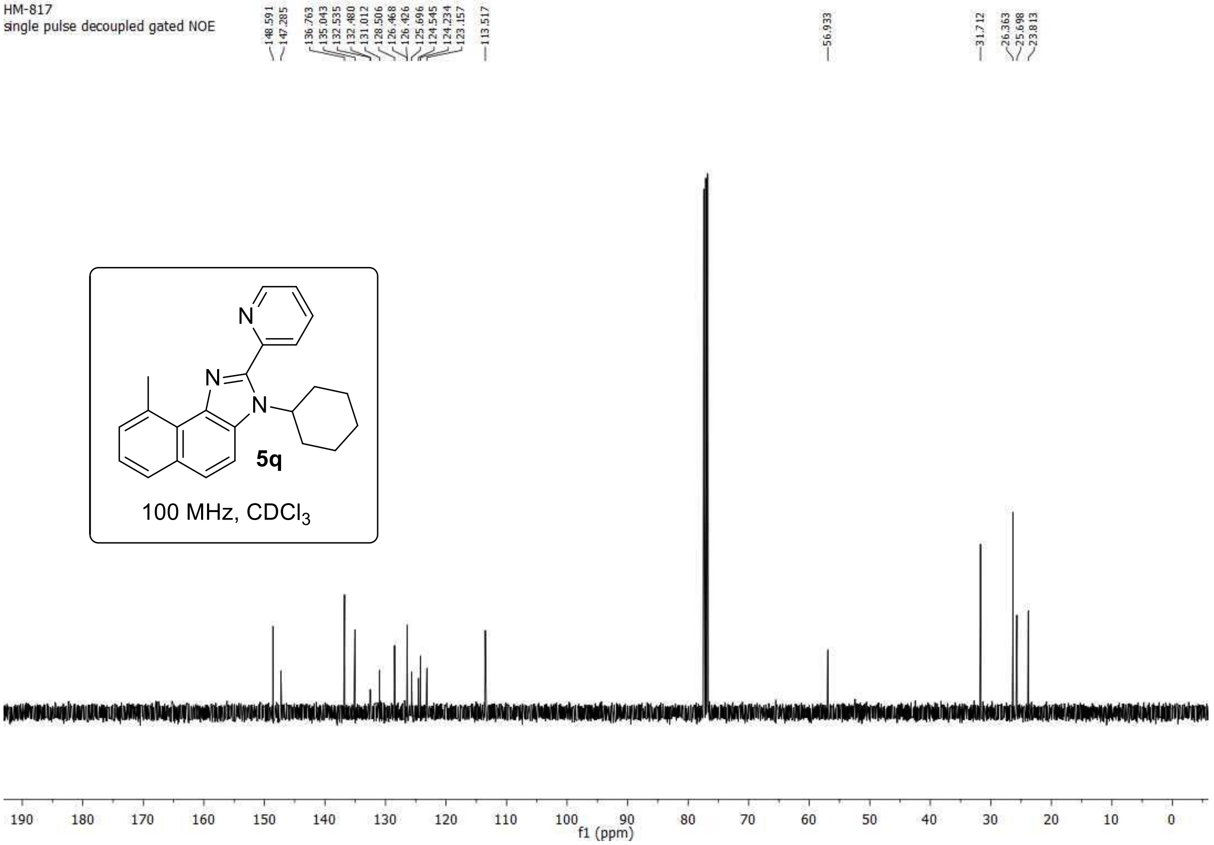
HM-817
single pulse decoupled gated NOE
148.591
147.285
136.763
135.043
132.535
132.480
131.012
128.506
126.468
126.426
125.696
124.545
124.234
123.157
113.517
56.933
31.712
26.363
25.698
23.813
N
N
N
5q
100 MHz, CDCl3
190
180
170
160
150
140
130
120
110
100
90
80
70
60
50
40
30
20
10
0
f1 (ppm)

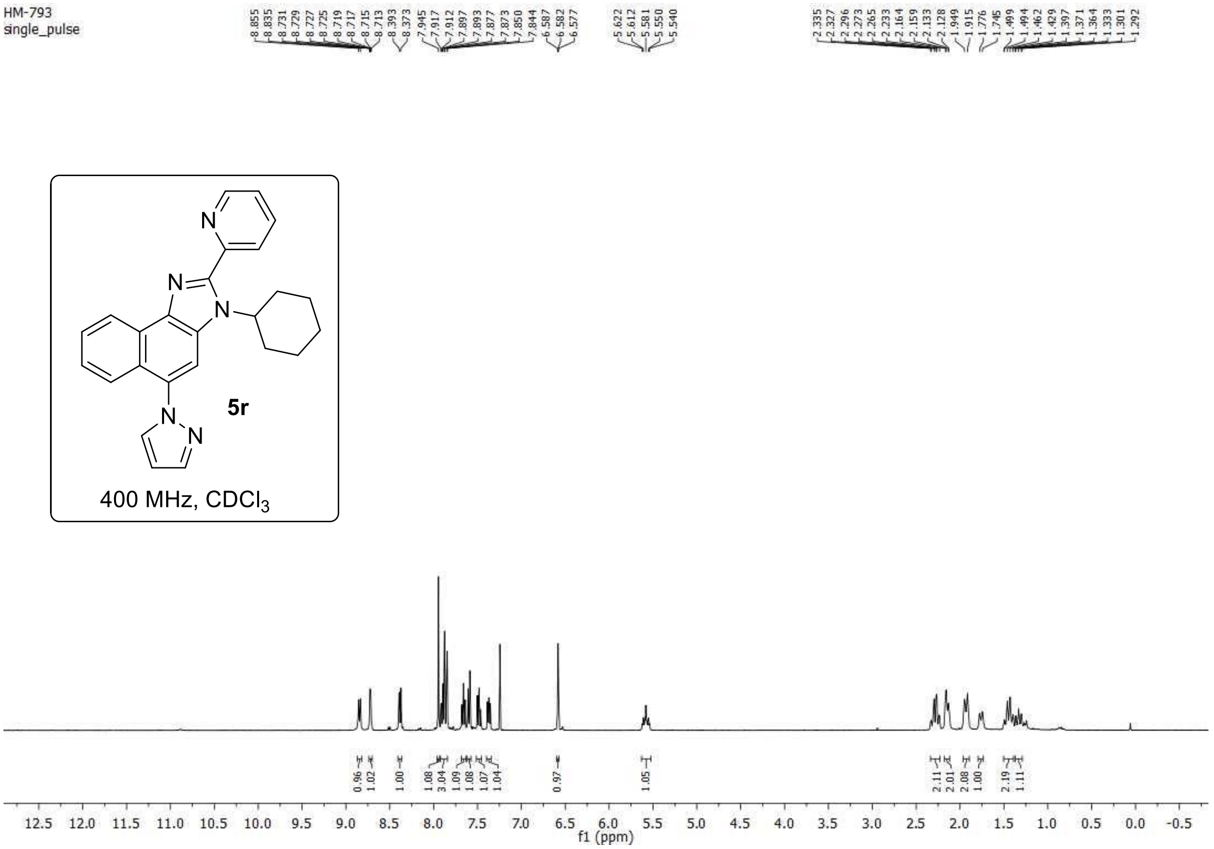
HM-793
single_pulse
8.855 8.835 8.731 8.729 8.727 8.725 8.719 8.717 8.715 8.713 8.393 8.373 7.945 7.917 7.912 7.897 7.893 7.877 7.873 7.850 7.844 6.587 6.582 6.577
5.622 5.612 5.581 5.550 5.540
2.335 2.327 2.296 2.273 2.265 2.233 2.164 2.159 2.133 2.128 1.949 1.915 1.776 1.745 1.499 1.494 1.462 1.429 1.397 1.371 1.364 1.333 1.301 1.292
N
N
N
5r
N
N
400 MHz, CDCl3
0.96 1.02 1.00 1.08 3.04 1.09 1.08 1.07 1.04 0.97 1.05 2.11 2.01 2.08 1.00 2.19 1.11
12.5 12.0 11.5 11.0 10.5 10.0 9.5 9.0 8.5 8.0 7.5 7.0 6.5 6.0 5.5 5.0 4.5 4.0 3.5 3.0 2.5 2.0 1.5 1.0 0.5 0.0 -0.5
f1 (ppm)

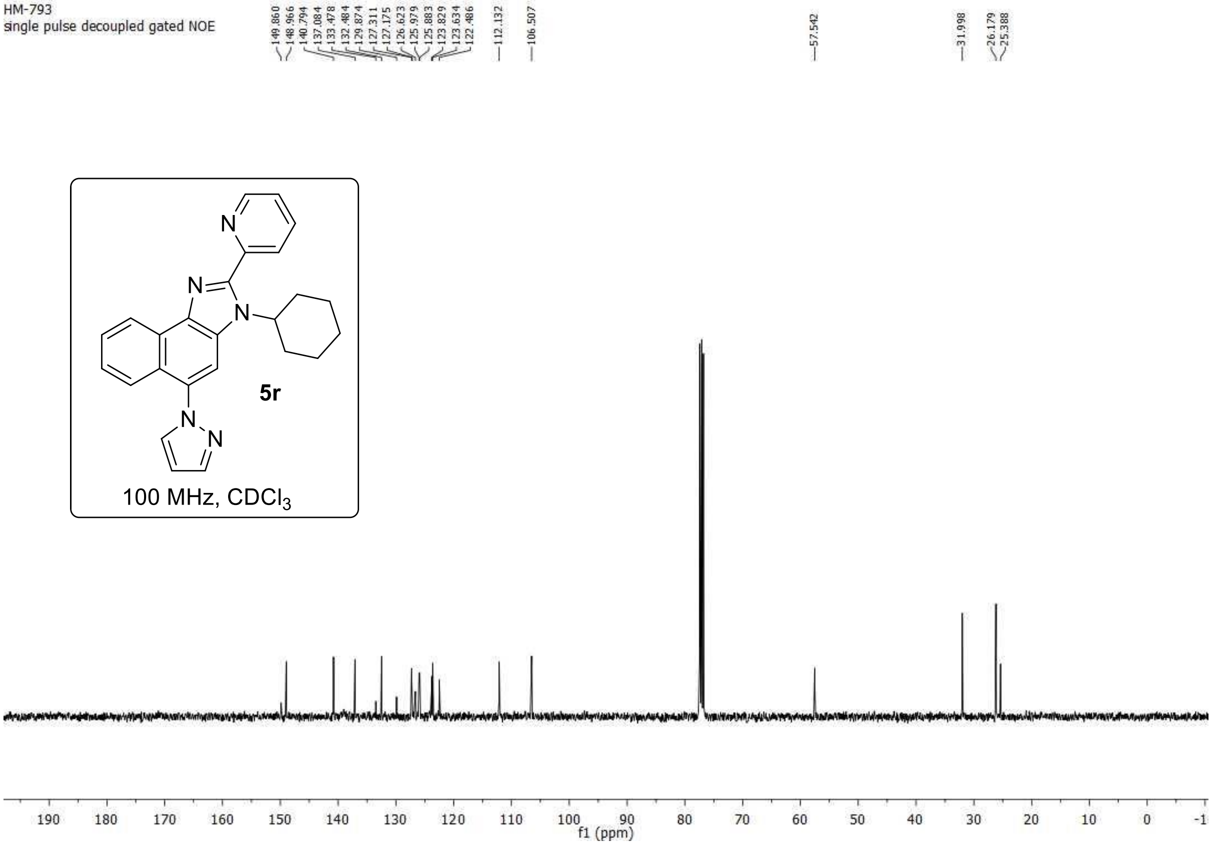
HM-793
single pulse decoupled gated NOE
149.860 148.966 140.794 137.084 133.478 132.484 129.874 127.311 127.175 126.623 125.979 125.883 123.829 123.634 122.486 112.132 106.507
57.542
31.998 26.179 25.388
N
N
N
5r
N
N
100 MHz, CDCl3
190 180 170 160 150 140 130 120 110 100 90 80 70 60 50 40 30 20 10 0 -1
f1 (ppm)

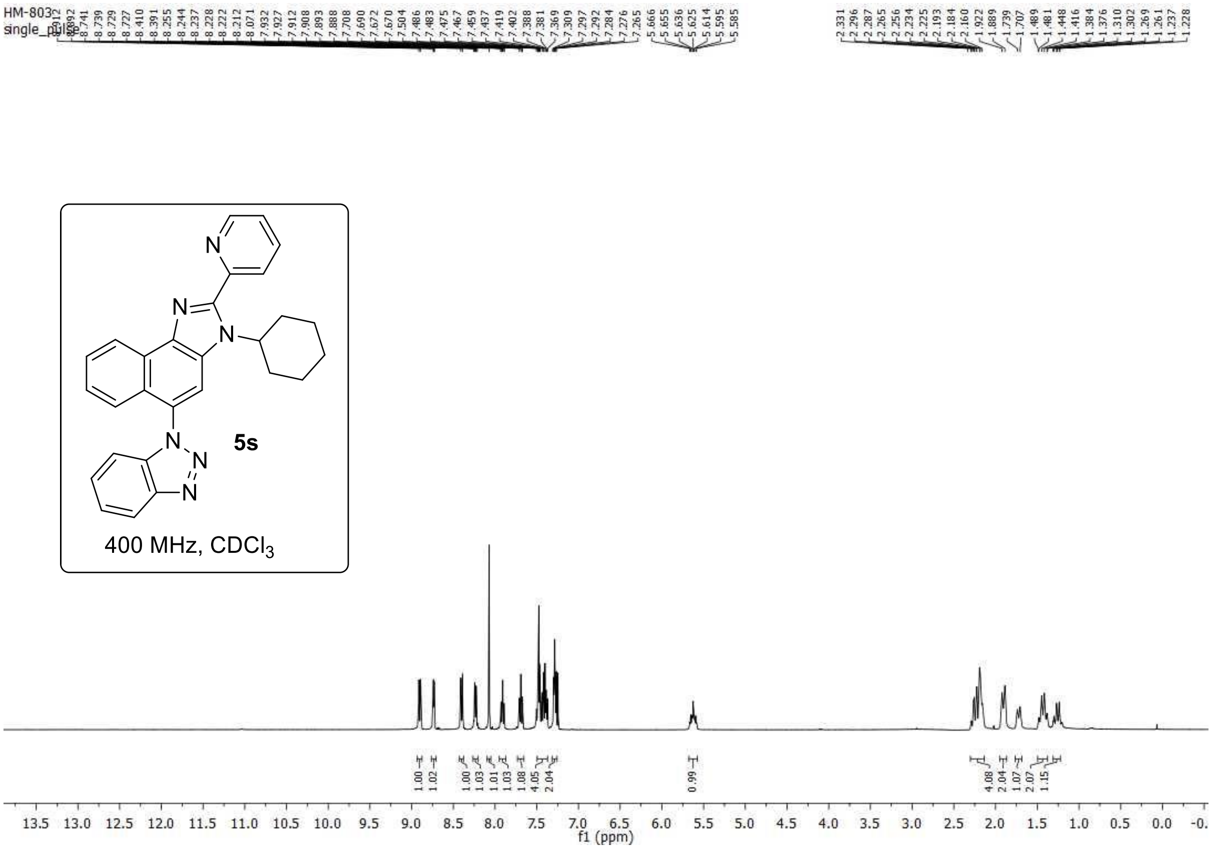
HM-803
single_pulse
8.912 8.892 8.741 8.739 8.729 8.727 8.410 8.391 8.255 8.244 8.237 8.228 8.222 8.212 8.071 7.932 7.927 7.912 7.908 7.893 7.888 7.708 7.690 7.672 7.670 7.504 7.486 7.483 7.475 7.467 7.459 7.437 7.419 7.402 7.388 7.381 7.369 7.309 7.297 7.292 7.284 7.276 7.265 5.666 5.655 5.636 5.625 5.614 5.595 5.585
2.331 2.296 2.287 2.265 2.256 2.234 2.225 2.193 2.184 2.160 1.922 1.889 1.739 1.707 1.489 1.481 1.448 1.416 1.384 1.376 1.310 1.302 1.269 1.261 1.237 1.228
5s
400 MHz, CDCl3
1.00 1.02 1.00 1.03 1.01 1.03 1.08 4.05 2.04 0.99 4.08 2.04 1.07 2.07 1.15
f1 (ppm)

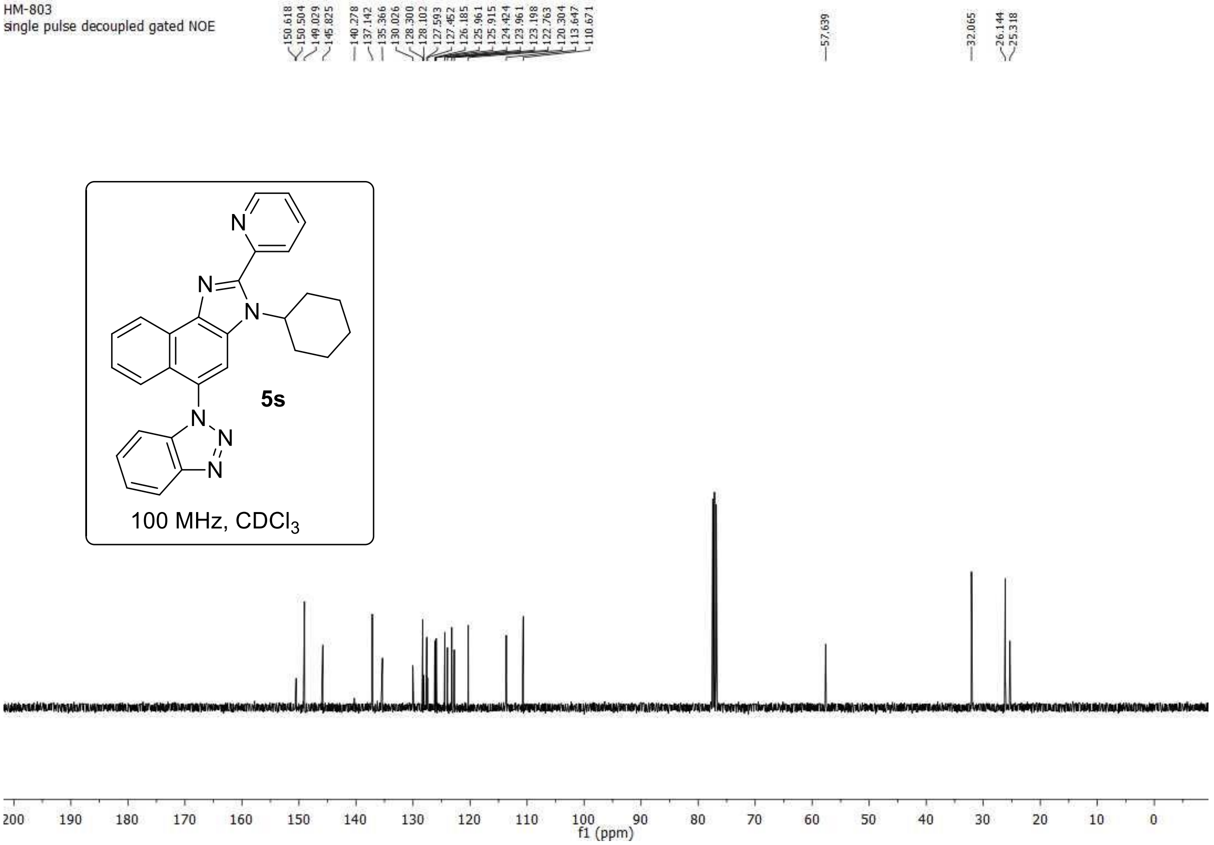
HM-803
single pulse decoupled gated NOE
150.618 150.504 149.029 145.825 140.278 137.142 135.366 130.026 128.300 128.102 127.593 127.452 126.185 125.961 125.915 124.424 123.961 123.198 122.763 120.304 113.647 110.671
57.639
32.065 26.144 25.318
5s
100 MHz, CDCl3
f1 (ppm)

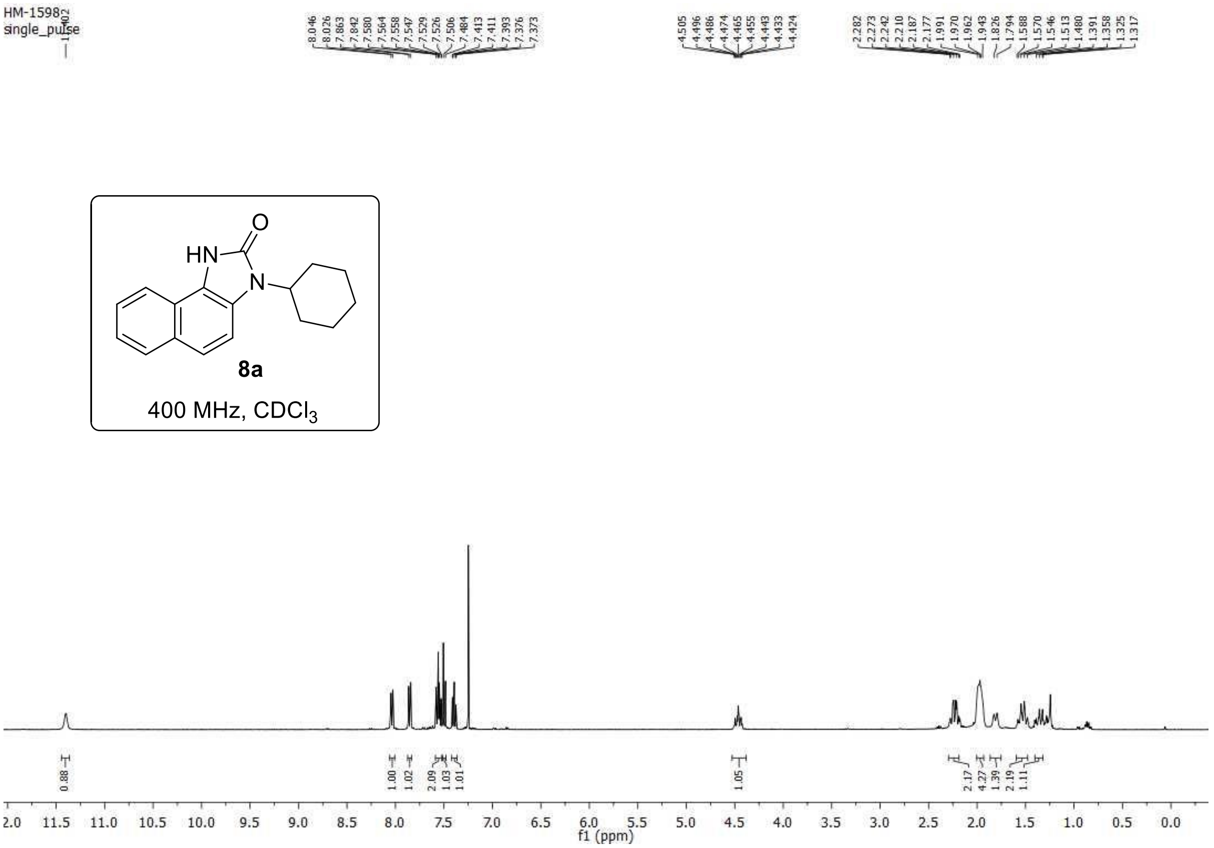
HM-1598
single_pulse
11.402
8.046 8.026 7.863 7.842 7.580 7.564 7.558 7.547 7.529 7.526 7.506 7.484 7.413 7.411 7.393 7.376 7.373
4.505 4.496 4.486 4.474 4.465 4.455 4.443 4.433 4.424
2.282 2.273 2.242 2.210 2.187 2.177 1.991 1.970 1.962 1.943 1.826 1.794 1.588 1.570 1.546 1.513 1.480 1.391 1.358 1.325 1.317
HN
O
N
8a
400 MHz, CDCl3
0.88
1.00
1.02
2.09
1.03
1.01
1.05
2.17
4.27
1.39
2.19
1.11
f1 (ppm)

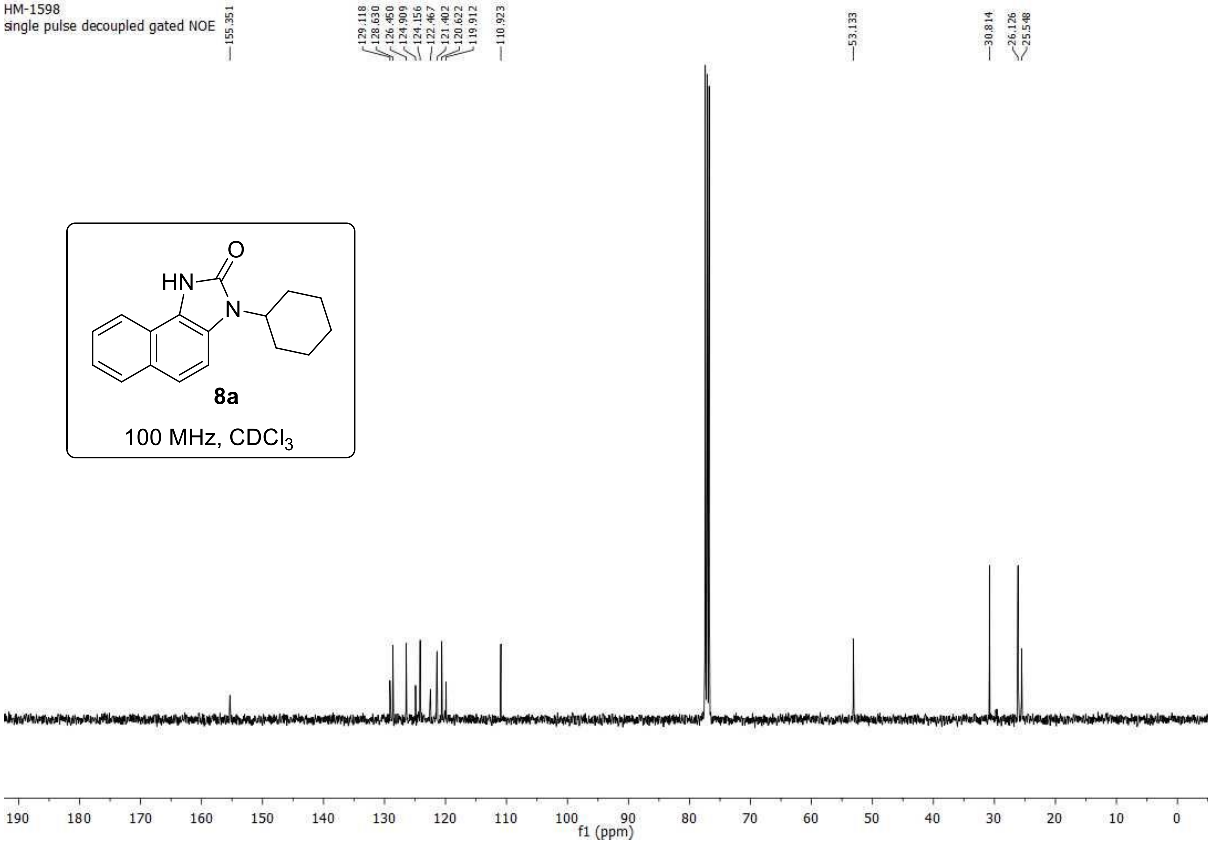
HM-1598
single pulse decoupled gated NOE
155.351
129.118 128.630 126.450 124.909 124.156 122.467 121.402 120.622 119.912
110.923
53.133
30.814
26.126 25.548
HN
O
N
8a
100 MHz, CDCl3
f1 (ppm)

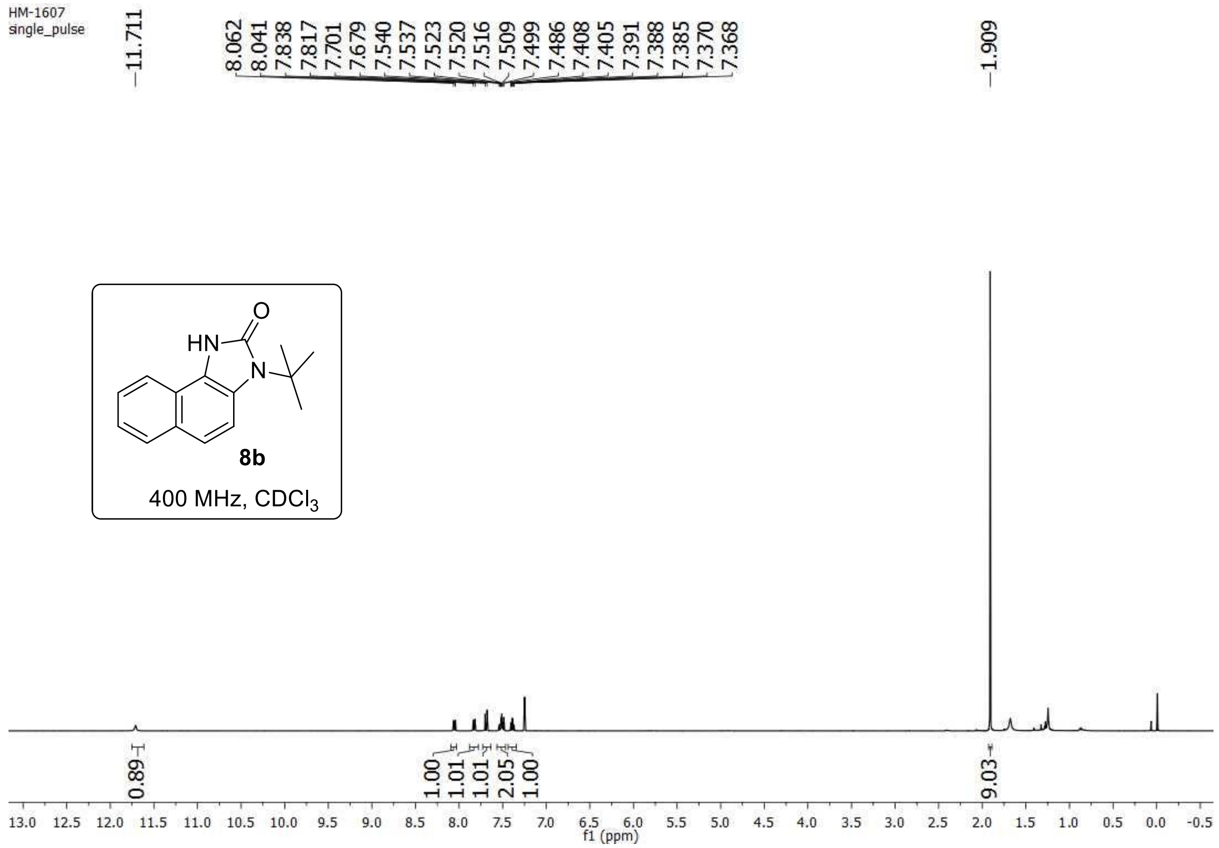
HM-1607
single_pulse
11.711
8.062
8.041
7.838
7.817
7.701
7.679
7.540
7.537
7.523
7.520
7.516
7.509
7.499
7.486
7.408
7.405
7.391
7.388
7.385
7.370
7.368
1.909
HN
O
N
8b
400 MHz, $CDCl_3$
0.89
1.00
1.01
1.01
2.05
1.00
9.03
13.0 12.5 12.0 11.5 11.0 10.5 10.0 9.5 9.0 8.5 8.0 7.5 7.0 6.5 6.0 5.5 5.0 4.5 4.0 3.5 3.0 2.5 2.0 1.5 1.0 0.5 0.0 -0.5
f1 (ppm)

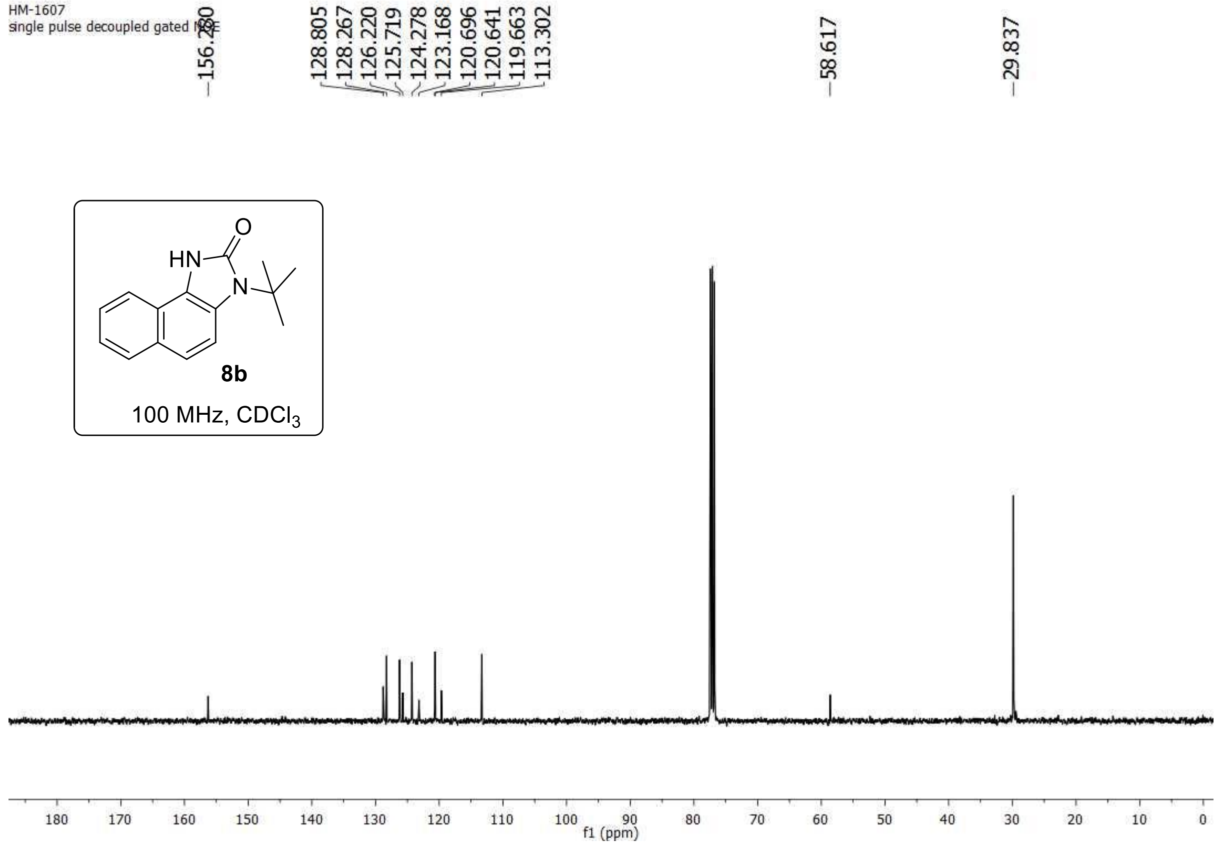
HM-1607
single pulse decoupled gated NOE
156.280
128.805
128.267
126.220
125.719
124.278
123.168
120.696
120.641
119.663
113.302
58.617
29.837
HN
O
N
8b
100 MHz, $CDCl_3$
180 170 160 150 140 130 120 110 100 90 80 70 60 50 40 30 20 10 0
f1 (ppm)
